# Investigating the relationship between non-occupational pesticide exposure and metabolomic biomarkers

**DOI:** 10.3389/fpubh.2023.1248609

**Published:** 2023-10-11

**Authors:** Saranya Palaniswamy, Khaled Abass, Jaana Rysä, Joan O. Grimalt, Jon Øyvind Odland, Arja Rautio, Marjo-Riitta Järvelin

**Affiliations:** ^1^Center for Life Course Health Research, Faculty of Medicine, University of Oulu, Oulu, Finland; ^2^Department of Epidemiology and Biostatistics, School of Public Health, Imperial College London, London, United Kingdom; ^3^Arctic Health, Faculty of Medicine, University of Oulu, Oulu, Finland; ^4^Department of Environmental Health Sciences, College of Health Sciences, University of Sharjah, Sharjah, United Arab Emirates; ^5^School of Pharmacy, University of Eastern Finland, Kuopio, Finland; ^6^Institute of Environmental Assessment and Water Research (IDAEA), Spanish Council for Scientific Research (CSIC), Barcelona, Spain; ^7^The Norwegian University of Science and Technology, Trondheim, Norway; ^8^School of Health Systems and Public Health, Faculty of Health Sciences, University of Pretoria, Pretoria, South Africa; ^9^Thule Institute, University of Arctic, University of Oulu, Oulu, Finland; ^10^Unit of Primary Care, Oulu University Hospital, Oulu, Finland; ^11^MRC-PHE Centre for Environment and Health, School of Public Health, Imperial College London, London, United Kingdom; ^12^Department of Life Sciences, College of Health and Life Sciences, Brunel University London, London, United Kingdom

**Keywords:** pesticides, metabolomics, Finland, general population, non-occupational exposure, non-communicable diseases

## Abstract

The relationship between pesticide exposures and metabolomics biomarkers is not well understood. We examined the changes in the serum metabolome (early biomarkers) and the metabolic pathways associated with various pesticide exposure scenarios (OPE: overall exposure, PEM: exposure in months, PEY: exposure in years, and PEU: reported specific pesticides use) using data from the Northern Finland Birth Cohort 1966 31-year cross-sectional examination. We utilized questionnaire data on pesticide exposures and serum samples for nuclear magnetic resonance (NMR)-based metabolomics analyses. For exposures and metabolites associations, participants size varied between 2,361 and 5,035. To investigate associations between metabolomics biomarkers and exposure to pesticide scenarios compared to those who reported no exposures multivariable regression analyses stratified by sex and adjustment with covariates (season of pesticide use, socioeconomic position (SEP), alcohol consumption, BMI, and latitude of residence) were performed. Multiple testing by Benjamini–Hochberg false discovery rate (FDR) correction applied. Pesticide exposures differed by sex, season of pesticide use, alcohol, SEP, latitude of residence. Our results showed that all pesticide exposure scenarios were negatively associated with decreased HDL concentrations across all lipoprotein subclasses in women. OPE, PEY, and PEU were associated with decreased branched-chain amino acid concentrations in men and decreased albumin concentrations in women. OPE, PEY and PEU were also associated with changes in glycolysis metabolites and ketone bodies in both sexes. Specific pesticides exposure was negatively associated with sphingolipids and inflammatory biomarkers in men. In women, OPE, PEM, and PEU were associated with decreased apolipoprotein A1 and increased apolipoprotein B/apolipoprotein A1 ratio. Our findings suggest that identification of early biomarkers of disease risk related to pesticide exposures can inform strategies to reduce exposure and investigate causal pathways. Women may be more susceptible to non-occupational pesticide exposures when compared to men, and future sex-specific studies are warranted.

## Introduction

The use of pesticides has rapidly expanded on a global scale in recent years (1). Pesticides encompass a broad range of chemicals, such as fungicides, herbicides, insecticides, and rodenticides, among others (1). People can be exposed to pesticides through various sources, including occupational and non-occupational routes such as consuming contaminated food and water, or through direct pesticide exposure (2). Despite their significant benefits to agriculture, long-term exposure to pesticides has been linked to the development of chronic diseases including neurodegenerative diseases (3, 4), type 2 diabetes (T2D), all-cause mortality (5–8) and other non-communicable diseases (NCDs). The mechanisms underlying how pesticides interact with biological pathways and contribute to the development of NCDs remain poorly understood.

Identification of biomarkers for exposure and early disease risk is crucial. Metabolomics is a valuable tool for discovering new biomarkers of pesticide exposure in epidemiological studies (9). This technique can directly observe metabolic changes in biological fluids, enabling the identification of early biomarkers of complex low-dose pesticide exposures in the general population (10, 11). Intermediate biomarkers can help explain the molecular and cellular mechanisms of pesticide toxicity (12). However, metabolomics only reveals changes in normal or altered metabolic functions and does not identify the underlying etiology of diseases. Some experimental studies on rats (13–16) and humans, such as epidemiological studies on the older adults (17, 18) and pregnant women (19–21), have reported metabolic perturbations associated with pesticide exposures. Nevertheless, over the past few years, a growing body of evidence suggests that even low-dose exposures to pesticides commonly found in the general population could produce a variety of biochemical changes and potentially lead to adverse health outcomes in humans. Moreover, men and women respond differently to the absorption rate, metabolism, and bioavailability of chemicals in relation to pesticide exposure (22–24). More research on sex-specific associations in relation to different pesticide exposure scenarios and low-dose exposures scenarios is needed to properly address exposures in women and related health outcomes (25). Non-targeted metabolomics can provide information about mechanisms, pathways, and biomarkers after pesticide exposures (26, 27). Additionally, metabolomics can also be employed to uncover the impact of low-dose exposure to pesticides on biochemical processes. Moreover, diagnostic biomarkers are not currently available for early detection of metabolic diseases, which makes metabolomics important in biomarker search.

In a previous study, clinical lipid parameters were measured in blood samples from the Northern Finland Birth cohort 1966 (n = 5,037) and the results were examined in relation to pesticide exposure, allowing different associations by sex to be identified. Now, in the present study, we applied for the first time, Nuclear Magnetic Resonance (NMR)-based metabolomics profiling in a large general population based setting to analyze the relationship between different non-occupational pesticide exposure scenarios (OPE: overall pesticide exposure, length of exposures (PEM: pesticide exposure in months; PEY: pesticide exposure in years), PEU: specific pesticides use reported), and changes in human systemic metabolism measured by circulating metabolomics biomarkers which provides a very detailed description of the lipid composition involved in health alterations/disturbances due to pesticide exposure. Metabolomics measurements provide a more comprehensive picture of an individual’s lipids and lipoprotein levels which cannot be determined by routine clinical lipid tests. Additionally, they can differentiate between different sizes of lipoproteins and other metabolites such as aminoacids. Results from the present study may advance our understanding of pesticide exposure perturbations at the metabolome-wide level and low-dose exposure scenarios. In addition, comparison with available diagnostic markers may provide information on their usefulness for early detection of metabolic diseases.

## Methods

### Study population

The current study is based on Northern Finland Birth Cohort 1966 (NFBC1966) 31-years participants. The detailed cohort information has been published previously (28) and as well included in the Supplementary information (29). Participants who took part in the study provided their written consent to utilize their data. All activities were performed in compliance with the 1964 Declaration of Helsinki. The Northern Ostrobothnia Hospital District’s ethics committee gave the NFBC1966 study their approval. A flowchart outlining the study population is presented in Supplementary Figure S1.

### Exposure assessment

The participants received a postal questionnaire which included questions regarding health in general, lifestyle, environment, and exposure to chemicals including pesticides.

The question enquired about “the duration of exposure to pesticides and plant protection products, asking respondents to indicate their length of exposure in both months (1–12 months) and years (one to multiple years).” In addition, “what type of pesticide and plant protection products (to specify the name of the pesticide used) were used” was asked simultaneously. The categorization of pesticide exposure scenarios was described previously in (23) and in the Supplementary information (Supplementary Tables S1, S2).

### Blood sample measurements

Participants in the NFBC1966 study were asked for a clinical examination after completing a postal questionnaire at 31-years. The blood samples were collected after an overnight fasting interval of 8 to 11 h, centrifuged, and stored initially at −20°C and then at −80°C. The samples were handled at a testing laboratory (T113) accredited by the Finnish Accreditation Service (FINAS) (EN ISO 15189), NordLab Oulu (former name Oulu University Hospital, Laboratory).

### Outcome assessment (metabolomics biomarkers)

Serum metabolomics biomarkers were measured using Proton Nuclear Magnetic Resonance (Nightingale Health Ltd., 2016 quantification version). Analytical methodology described in (30) and the metabolomics platform is based on three molecular windows for each sample: lipid, low-molecular-weight metabolite data, and lipoprotein. We assessed the lipid and lipoprotein traits that are related to low-density lipoprotein (LDL), high-density lipoprotein (HDL), very low-density lipoprotein (VLDL) and intermediate-density lipoprotein (IDL) and apolipoproteins, amino acids, ketone bodies, sphingolipids, metabolites related to glycolysis pathway, fatty acids, inflammation, and fluid balance. The subfraction traits are denoted using a three-part naming convention, separated by hyphens. The first component signifies the size classification (XS, S, M, L, XL, XXL), the second component represents the lipoprotein density fraction (VLDL, LDL, IDL, HDL), and the third component indicates the specific measurements, such as triglycerides (TG), free cholesterol (FC), phospholipids (PL), total lipids (L), cholesterol esters (CE), total cholesterol (C). For instance, the term S-HDL-P refers to the concentration measurement of small-sized high-density lipoprotein particles. A detailed list of names and abbreviations of circulating metabolites is given in the online Supplementary material (Appendices 3, 4).

### Covariates

Demographic characteristics such as sex, socioeconomic position (SEP), lifestyle variables, season of pesticide use, anthropometry (body mass index (BMI)) and latitude of residence were accounted for in the study as covariates as reported from previous literature. Regression analyses were conducted separately for men and women, with adjustments made for covariates such as season of pesticide use, BMI, alcohol consumption, socioeconomic position, and latitude of residence. More details can be found in the online Supplementary Appendix 1.

### Statistical analyses

Descriptive statistics calculated for all explanatory, confounding, and outcome measures, with normally distributed variables presented as mean (95% CI), non-parametric variables as median (IQR), and categorical variables as n (%). To compare the differences between participants with and without exposure to OPE, PEM, PEY, and PEU, chi-square test for categorical variables, independent-sample Student’s t test for normally distributed data, and the Wilcoxon–Mann–Whitney U test for non-parametric data were used. The distribution of metabolites was examined, and natural logarithm-transformation of all measured metabolite concentrations was performed to mitigate skewness. Standardization (*z*-scores) of transformed variables was performed so that the magnitude of effects is comparable across different pesticide exposure scenarios in the regression analyses.

To determine the associations between various pesticide exposure scenarios (OPE, PEM, PEY, and PEU) and metabolomics biomarkers, multivariable regression analysis was carried out. The models were stratified by sex and adjusted for BMI, socioeconomic position, season of pesticide use, alcohol consumption, and latitude of residence in subsequent steps. To account for multiple testing, the false discovery rate (FDR) was corrected using the Benjamini-Hochberg procedure. The resulting regression coefficients can be interpreted as the change in the category (yes/no) of OPE, PEM, PEY, and PEU per 1-SD change in the concentration of the metabolomic biomarkers. All statistical analyses were performed using SAS version 9.4 (SAS Institute Inc.) and R version 3.6.3 (R Project for Statistical Computing) with a significance threshold of *p* < 0.05 for 2-sided tests.

## Results

### Study population (NFBC1966) characteristics

Characteristics of pesticide exposure scenarios with anthropometric (BMI), lifestyle (alcohol, smoking), socioeconomic position, environmental and demographic covariates are shown in Supplementary Tables S3, S4. Pesticide exposure scenarios differed with socioeconomic position and sex. Alcohol consumption and season of pesticide use differed with years of pesticide exposure. Latitude-related differences existed between PEY and PEU.

The spearman’s correlation coefficients between pesticide exposure scenarios and circulating metabolites are shown in Supplementary Table S5. Correlations between pesticide exposures and lipoprotein subclasses were in the positive direction, except for HDL family components including very-large, large, medium, and small HDL (*p* > 0.05). In addition, all pesticide exposures were positively correlated with the apolipoprotein (apo) B/apo A1 ratio.

### Pesticide exposure scenarios with lipoprotein contents in men – multivariable regression results

Regression results of pesticide exposure scenarios (OPE, PEM, PEY and PEU) with circulating metabolites after adjustment with multiple covariates and FDR multiple testing correction are presented in Tables 1–4. The detailed results of the unadjusted and adjusted models with BMI, socioeconomic position, season of pesticide use, alcohol consumption and latitude of residence separately for men and women are presented in Supplementary Tables S6–S9.

**Table 1:** Multiple linear regression analyses on the association between overall pesticide exposure (OPE) with standardized (*z*-score) metabolomics biomarkers.

Metabolomics clusters	β (95% confidence intervals)^1^^,^^2^	*p*-value	*p*-value correction (Separately for males and females) ^1^
*Males*			
Cholesterol esters			
XXL_VLDL_CE	0.166 (0.014, 0.319)	0.0325	0.032
Branched-chain amino acids			
Valine [μmol/L]	−0.170 (−0.315, −0.026)	0.0208	0.031
Ketone bodies			
Acetate [μmol/L]	0.222 (0.053, 0.391)	0.0099	0.031
*Glycolysis related metabolites*			
Lactate	−0.189 (−0.352, −0.026)	0.0231	0.031
*Females*			
Total lipoprotein			
L_HDL_P	−0.274 (−0.473, −0.075)	0.0069	0.014
M_HDL_P	−0.331 (−0.566, −0.096)	0.0058	0.014
S_HDL_P	−0.322 (−0.566, −0.078)	0.0096	0.015
Triglycerides			
L_HDL_TG	−0.278 (−0.509, −0.047)	0.0183	0.021
Phospholipids			
L_HDL_PL	−0.287 (−0.488, −0.086)	0.0052	0.014
M_HDL_PL	−0.339 (−0.578, −0.100)	0.0054	0.014
S_HDL_PL	−0.452 (−0.691, −0.213)	0.0002	0.002
Cholesterol esters			
L_HDL_CE	−0.230 (−0.413, −0.048)	0.0134	0.017
M_HDL_CE	−0.306 (−0.534, −0.078)	0.0085	0.014
Free cholesterol			
L_HDL_FC	−0.179 (−0.354, −0.0042)	0.0448	0.045
M_HDL_FC	−0.305 (−0.531, −0.079)	0.0080	0.014
S_HDL_FC	−0.484 (−0.733, −0.234)	0.0001	0.002
Total lipids			
L_HDL_L	−0.267 (−0.465, −0.070)	0.0079	0.014
M_HDL_L	−0.327 (−0.559, −0.096)	0.0057	0.014
S_HDL_L	−0.332 (−0.579, −0.086)	0.0082	0.014
Total cholesterol			
L_HDL_C	−0.222 (−0.406, −0.039)	0.0174	0.021
M_HDL_C	−0.309 (−0.535, −0.084)	0.0072	0.014
HDL_C	−0.273 (−0.485, −0.061)	0.0116	0.017
Apolipoproteins			
Apolipoprotein A1 [g/L]	−0.303 (−0.539, −0.066)	0.0122	0.017
Apo B/Apo A1 ratio	0.281 (0.079, 0.483)	0.0065	0.014
Ketone bodies			
Acetoacetate [μmol/L]	−0.257 (−0.498, −0.015)	0.0373	0.041
Glycolysis related metabolites			
Glycerol [mmol/L]	0.246 (0.010, 0.482)	0.0410	0.043
Fluid balance			
Albumin	−0.355 (−0.566, −0.144)	0.0010	0.008

1 Model adjusted for BMI, socioeconomic position, season of pesticide use, alcohol consumption and latitude of residence. Multiple testing by Benjamin-Hochberg False discovery rate correction separately for men and women and each cluster.

2 The *z*-score standardized regression coefficients (β coefficients, 95% CI) represent the change in biomarker concentrations per 1 standard deviation change in overall pesticide exposure (OPE) category.

**Table 2:** Multiple linear regression analyses on the association between pesticide exposure in months (PEM) with standardized (*z*-score) metabolomics biomarkers.

Metabolomics clusters	β (95% confidence intervals)^1^^,^^2^	*p*-value	*p*-value correction (Separately for males and females)^1^
*Females*			
Total lipoprotein			
L_HDL_P	−0.318 (−0.563, −0.074)	0.0108	0.023
M_HDL_P	−0.372 (−0.661, −0.083)	0.0117	0.023
S_HDL_P	−0.340 (−0.641, −0.040)	0.0262	0.031
Phospholipids			
L_HDL_PL	−0.339 (−0.586, −0.091)	0.0073	0.023
M_HDL_PL	−0.388 (−0.682, −0.094)	0.0097	0.023
S_HDL_PL	−0.532 (−0.826, −0.238)	0.0004	0.004
Cholesterol esters			
L_HDL_CE	−0.278 (−0.503, −0.053)	0.0153	0.024
M_HDL_CE	−0.354 (−0.634, −0.073)	0.0135	0.023
Free cholesterol			
L_HDL_FC	−0.229 (−0.445, −0.0144)	0.0366	0.038
M_HDL_FC	−0.349 (−0.627, −0.071)	0.0139	0.023
S_HDL_FC	−0.576 (−0.883, −0.269)	0.0002	0.004
Total lipids			
L_HDL_L	−0.313 (−0.556, −0.070)	0.0115	0.023
M_HDL_L	−0.370 (−0.656, −0.085)	0.0110	0.023
S_HDL_L	−0.353 (−0.656, −0.050)	0.0224	0.028
Total cholesterol			
L_HDL_C	−0.270 (−0.496, −0.045)	0.0189	0.025
M_HDL_C	−0.356 (−0.633, −0.078)	0.0120	0.023
HDL_C	−0.315 (−0.576, −0.054)	0.0181	0.025
Apolipoproteins			
Apolipoprotein A1 [g/L]	−0.323 (−0.614, −0.031)	0.0301	0.033
Apo B/Apo A1 ratio	0.367 (0.118, 0.616)	0.0038	0.023
Glycolysis related metabolites			
Citrate [μmol/L]	0.298 (0.016, 0.579)	0.0385	0.038

1 Model adjusted for BMI, socioeconomic position, season of pesticide use, alcohol consumption and latitude of residence. Multiple testing by Benjamin-Hochberg False discovery rate correction separately for men and women and each cluster.

2 The *z*-score standardized regression coefficients (β coefficients, 95% CI) represent the change in biomarker concentrations per 1 standard deviation change in pesticide exposures in months (PEM) category.

**Table 3:** Multiple linear regression analyses on the association between pesticide exposure in years (PEY) with standardized (*z*-score) metabolomics biomarkers.

Metabolomics clusters	β (95% confidence intervals)^1^^,^^2^	*p*-value	*p*-value correction (Separately for males and females)^1^
*Males*			
Branched-chain amino acids			
Isoleucine [μmol/L]	−0.208 (−0.400, −0.016)	0.0336	0.034
Valine [μmol/L]	−0.259 (−0.443, −0.074)	0.0061	0.018
Ketone bodies			
Acetate [μmol/L]	0.259 (0.043, 0.476)	0.0186	0.028
*Females*			
Phospholipids			
S_HDL_PL	−0.488 (−0.903, −0.073)	0.0213	0.05
Free cholesterol			
S_HDL_FC	−0.442 (−0.876, −0.0087)	0.0456	0.05
Ketone bodies			
Acetoacetate [μmol/L]	−0.425 (−0.850, −0.00039)	0.0498	0.05
beta-hydroxybutyrate [μmol/L]	−0.452 (−0.889, −0.015)	0.0425	0.05
Fluid balance			
Albumin	−0.453 (−0.819, −0.087)	0.0154	0.05

1 Model adjusted for BMI, socioeconomic position, season of pesticide use, alcohol consumption and latitude of residence. Multiple testing by Benjamin-Hochberg False discovery rate correction separately for men and women and each cluster.

2 The *z*-score standardized regression coefficients (β coefficients, 95% CI) represent the change in biomarker concentrations per 1 standard deviation change in pesticide exposures in years (PEY) category.

**Table 4:** Multiple linear regression analyses on the association between specific pesticides use (PEU) with standardized (*z*-score) metabolomics biomarkers.

Metabolomics clusters	β (95% confidence intervals)^1^^,^^2^	*p*-value	*p*-value correction (Separately for males and females)^1^
*Males*			
Ketone bodies			
Acetoacetate [μmol/L]	−0.246 (−0.489, −0.0015)	0.0486	0.049
Sphingolipids			
Sphingomyelin	−0.263 (−0.515, −0.012)	0.0404	0.049
Inflammation			
Alpha-1-acid glycoprotein	−0.262 (−0.511, −0.013)	0.0391	0.049
*Females*			
Total lipoprotein			
XXL_VLDL_P	0.276 (0.0062, 0.546)	0.0450	0.047
L_HDL_P	−0.365 (−0.628, −0.103)	0.0064	0.016
M_HDL_P	−0.437 (−0.747, −0.127)	0.0058	0.016
S_HDL_P	−0.432 (−0.754, −0.111)	0.0085	0.017
Triglycerides			
XXL_VLDL_TG	0.279 (0.010, 0.548)	0.0418	0.047
L_HDL_TG	−0.337 (−0.642, −0.032)	0.0304	0.041
Phospholipids			
L_HDL_PL	−0.387 (−0.653, −0.122)	0.0042	0.016
M_HDL_PL	−0.453 (−0.768, −0.138)	0.0049	0.016
S_HDL_PL	−0.643 (−0.959, −0.328)	<0.0001	0.002
Cholesterol esters			
XXL_VLDL_CE	0.372 (0.068, 0.676)	0.0166	0.025
XL_VLDL_CE	0.318 (0.0068, 0.629)	0.0452	0.047
L_VLDL_CE	0.385 (0.070, 0.699)	0.0166	0.025
M_VLDL_CE	0.311 (0.031, 0.592)	0.0295	0.041
L_HDL_CE	−0.314 (−0.555, −0.074)	0.0106	0.020
M_HDL_CE	−0.432 (−0.733, −0.131)	0.0050	0.016
Free cholesterol			
L_HDL_FC	−0.247 (−0.479, −0.016)	0.0360	0.046
M_HDL_FC	−0.416 (−0.714, −0.118)	0.0062	0.016
S_HDL_FC	−0.691 (-1.019, −0.362)	<0.0001	0.002
Total lipids			
XXL_VLDL_L	0.269 (0.00021, 0.538)	0.0498	0.050
L_HDL_L	−0.358 (−0.619, −0.097)	0.0071	0.016
M_HDL_L	−0.437 (−0.743, −0.131)	0.0052	0.016
S_HDL_L	−0.447 (−0.772, −0.122)	0.0071	0.016
Total cholesterol			
XXL_VLDL_C	0.295 (0.0074, 0.583)	0.0444	0.047
L_VLDL_C	0.313 (0.011, 0.615)	0.0425	0.047
L_HDL_C	−0.304 (−0.546, −0.062)	0.0138	0.023
M_HDL_C	−0.431 (−0.729, −0.133)	0.0045	0.016
HDL_C_	−0.387 (−0.667, −0.108)	0.0067	0.016
Apolipoproteins			
Apolipoprotein A1 [g/L]	−0.393 (−0.706, −0.080)	0.0138	0.023
Apo B/Apo A1 ratio	0.428 (0.161, 0.695)	0.0017	0.016
Ketone bodies			
Acetoacetate [μmol/L]	−0.362 (−0.678, −0.045)	0.0251	0.037
Glycolysis related metabolites			
Glycerol [mmol/L]	0.326 (0.019, 0.632)	0.0373	0.046
Fluid balance			
Albumin	−0.374 (−0.653, −0.095)	0.0086	0.017

1 Model adjusted for BMI, socioeconomic position, season of pesticide use, alcohol consumption and latitude of residence. Multiple testing by Benjamin-Hochberg False discovery rate correction separately for men and women and each cluster.

2 The *z*-score standardized regression coefficients (β coefficients, 95% CI) represent the change in biomarker concentrations per 1 standard deviation change in reported specific pesticide use (PEU) category.

In Table 1, overall exposure was positively associated with XXL_VLDL_CE (β = 0.166; 95% CI: 0.014, 0.319; FDR corrected value of *p* = 0.032) after adjustment with multiple covariates. However, there were no associations observed between pesticide exposure scenarios and any other lipoprotein subclasses in men.

### Pesticide exposure scenarios with lipoproteins in women – multivariable regression results

Overall pesticide exposure was negatively associated with the HDL component in total lipoprotein (L_HDL_P, M_HDL_P, S_HDL_P), triglycerides (L_HDL_TG), phospholipids (L_HDL_PL, M_HDL_PL, S_HDL_PL), cholesterol esters (L_HDL_CE, M_HDL_CE), free cholesterol (L_HDL_FC, M_HDL_FC, S_HDL_FC), total lipids (L_HDL_L, M_HDL_L, S_HDL_L), and total cholesterol (L_HDL_C, M_HDL_C, HDL_C; Table 1). Similarly, PEM was negatively associated with HDL components in all clusters, except for triglycerides (Table 2). However, years of pesticide exposures was only negatively associated with the HDL component in phospholipids (S_HDL_PL) and free cholesterol (S_HDL_FC; Table 3). Similar to OPE, PEU was associated with decreased HDL concentrations in all lipoprotein profiles (Table 4). In addition, specific pesticide use was associated with increased VLDL concentrations in total lipoprotein (XXL_VLDL_P), triglycerides (XXL_VLDL_TG), cholesterol esters (XXL_VLDL_CE, XL_VLDL_CE, L_VLDL_CE, M_VLDL_CE), total lipids (XXL_VLDL_L) and total cholesterol (XXL_VLDL_C, L_VLDL_C; Table 4).

### Pesticide exposure scenarios with aminoacids, ketone bodies, glycolysis-related metabolites, sphingolipids in men – multivariable regression results

Overall exposure (Table 1) and pesticide exposure in years (Table 3) was negatively associated with branched-chain amino acid valine (OPE, β = −0.170; 95% CI: −0.315, −0.026; FDR value of *p* = 0.031; PEM, β = −0.259; 95% CI: −0.443, −0.074; FDR value of *p* = 0.018). Moreover, both OPE and PEY was positively associated with acetate (OPE, β = 0.222; 95% CI: 0.053, 0.391; FDR value of *p* = 0.031; PEY, β = 0.259; 95% CI: 0.043, 0.476; FDR value of *p* = 0.028). Specific pesticide use was negatively associated with branched-chain amino acid, isoleucine (β = −0.208; 95% CI: −0.400, −0.016; FDR value of *p* = 0.034), ketone body, acetoacetate (β = −0.246; 95% CI: −0.489, −0.0015; FDR value of *p* = 0.049) and sphingomyelin (β = −0.263; 95% CI: −0.515, −0.012; FDR value of p = 0.049). In addition, overall pesticide exposure was associated with decreased lactate concentrations (β = −0.189; 95% CI: −0.352, −0.026; FDR value of p = 0.031).

### Pesticide exposure scenarios with apolipoproteins, ketone bodies and glycolysis-related metabolites in women – multivariable regression results

Overall pesticide exposure (OPE, β = −0.303; 95% CI: −0.539, −0.066; FDR value of *p* = 0.017; Table 1), pesticide exposure in months (PEM, β = −0.323; 95% CI: −0.614, −0.031; FDR value of *p* = 0.033; Table 2), and specific pesticides use (PEU, β = −0.393; 95% CI: −0.706, −0.080; FDR value of *p* = 0.023; Table 4) associated with decreased apolipoprotein A1. Similar pesticide exposure scenarios were associated with increased Apo B/Apo A1 ratio (OPE, β = 0.281; 95% CI: 0.079, 0.483; FDR value of *p* = 0.014; Table 1; PEM, β = 0.367; 95% CI: 0.118, 0.616; FDR value of p = 0.023; PEU, β = 0.428; 95% CI: 0.161, 0.695; FDR value of *p* = 0.016; Table 4). OPE (β = −0.257; 95% CI: −0.498, −0.015; FDR value of *p* = 0.041; Table 1) and PEU (β = −0.362; 95% CI: −0.678, −0.045; FDR value of *p* = 0.037; Table 4) was negatively associated with ketone body acetoacetate. Decreased concentrations of ketone bodies, acetoacetate (β = −0.425; 95% CI: −0.850, −0.00039; FDR value of *p* = 0.05) and beta-hydroxybutyrate (β = −0.452; 95% CI: −0.889, −0.015; FDR value of *p* = 0.05; Table 3) were associated with years of pesticide exposure. Different pesticide exposure scenarios were also associated with changes in glycolysis-related metabolites, overall pesticide exposure and reported pesticides use with glycerol (OPE, β = 0.246; 95% CI: 0.010, 0.482; FDR value of *p* = 0.043; Table 1; PEU, β = 0.326; 95% CI: 0.019, 0.632; FDR value of *p* = 0.046; Table 4) and pesticide exposure in months with citrate (β = 0.298; 95% CI: 0.016, 0.579; FDR value of *p* = 0.038; Table 2).

### Pesticide exposure scenarios with fluid balance, inflammation in men and women – multivariable regression results

**Figure 1:**
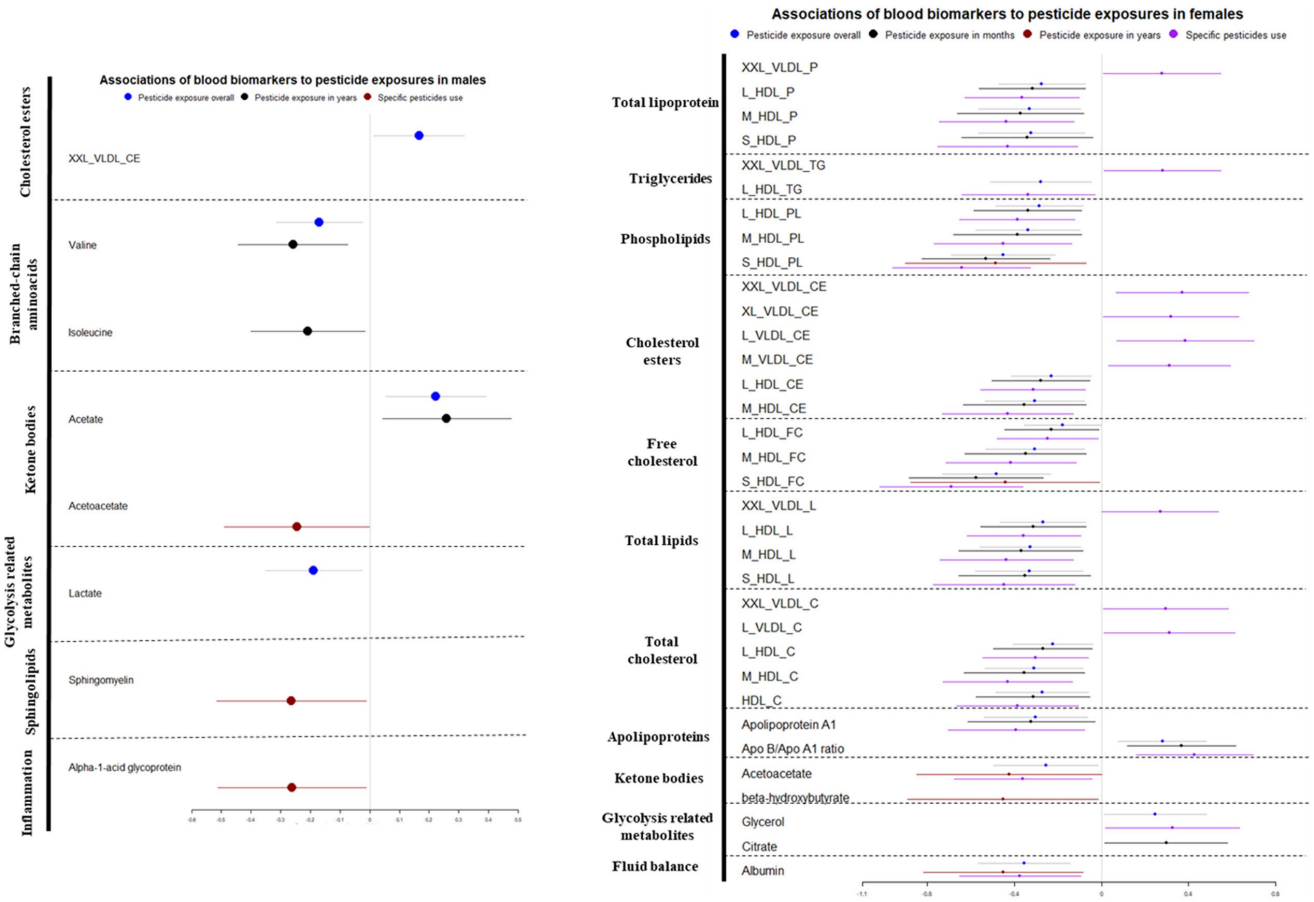
Circulating metabolomics biomarkers and its associations with different pesticide exposure scenarios in men and women (category change in pesticide exposure per 1-SD change in biomarker concentration, β coefficients and 95% CI). Final model adjusted for BMI, socioeconomic position, season of pesticide use, alcohol consumption and latitude of residence).

Pesticide exposure scenarios (overall exposure, years of pesticide exposure, reported pesticides use) was associated with decreased fluid balance marker albumin (OPE, β = −0.355; 95% CI: −0.566, −0.144; FDR value of *p* = 0.008; Table 1; PEY, β = −0.453; 95% CI: −0.819, −0.087; FDR value of *p* = 0.05; Table 3; PEU, β = −0.374; 95%CI: −0.653, −0.095; FDR value of *p* = 0.017; Table 4) in women. Specific pesticide use was also negatively associated with the inflammatory biomarker alpha-1-acid glycoprotein (β = −0.262; 95% CI: −0.511, −0.013; FDR value of *p* = 0.049; Table 4) in men. Figure 1 depicts the regression results of the associations of metabolomics biomarkers with different pesticide scenarios and across the pesticide exposure scenarios stratified by sex after multiple testing corrections.

## Discussion

The study was conducted to examine the impact of various non-occupational pesticide exposure scenarios on metabolomics biomarkers in young adults from Finland. The study also stratified the analyses by sex and adjusted for several covariates, including BMI, socioeconomic position, season of pesticide use, alcohol consumption, and latitude of residence. Additionally, a correction for multiple testing was carried out in the study’s analyses.

### Lipoprotein subclasses

In women, all pesticide exposure scenarios were associated with decreased high-density lipoprotein concentrations of different size in all lipoprotein subclasses (Figure 1).

Concerning phospholipids (PL), women showed negative associations between overall exposure, pesticide exposure in months and reported specific pesticides use with high-density lipoproteins of different size, such as S_HDL_PL, M_HDL_PL and L_HDL_PL; except, years of pesticide exposure was only associated with decreased S_HDL_PL concentrations. Furthermore, in women negative associations with high-density lipoproteins of total lipoproteins (P), free cholesterol (FC), total lipids (L), triglycerides (TG) and total cholesterol (C), involving compounds of small (S), medium (M) or large size (L) and pesticide exposure scenarios (overall, months of exposure and specific pesticides reported) were observed. According to the findings of our study, all the pesticide exposure scenarios examined were found to have a negative association with several measures of HDL, including most sizes of HDL in total lipoproteins (P), as well as with phospholipids (PL), total cholesterol (C), free cholesterol (FC), cholesterol esters (CE), and total lipids (L; Figure 1).

Concerning cholesterol ester (CE), a positive association of the extremely large very low-density lipoprotein (XXL_VLDL_CE) and overall pesticide exposure was found in men. Similar positive associations were also observed in women who reported specific pesticides exposure and cholesterol esters in very low-density lipoproteins of different sizes, XXL_VLDL_CE, XL_VDL_CE, L_VLDL_CE and M_VLDL_CE. In contrast, a negative association between pesticide scenarios (overall, months of exposure and specific pesticides use) and cholesterol esters in varying sizes of high-density lipoprotein (L_HDL_CE and M_HDL_CE) concentrations was observed in women. Our study observations align with previous research that has reported positive associations between total cholesterol and LDL cholesterol in women (23). However, our study offers a more comprehensive description of the specific types of lipids that are involved in these associations.

There have been only a limited number of experimental and epidemiological studies that have examined the potential link between exposure to pesticides and changes in blood lipid markers (23, 31–34). Common methods of assessing lipids in the bloodstream, such as measuring levels of LDL and HDL, do not differentiate between the size, density, concentration, and composition of lipoproteins. These factors may have varying levels of importance when it comes to determining the risk of cardiovascular disease (30). The current method of clinically measuring HDL levels involves only quantifying the total cholesterol content of the HDL particles, while disregarding important factors such as their composition (including TG and phospholipids), particle size, and subclass concentration. However, the present study has been able to provide information of changes in individual subclasses of lipoprotein particles and lipid-related characteristics resulting from exposure to pesticides. These findings can serve as early indicators of an individual’s susceptibility to cardiovascular diseases (35, 36). Prior studies have demonstrated that VLDL, LDL, and the associated lipids are predictive of both cardiovascular disease (CVD) and the development of T2D (37, 38). In addition, our study’s results confirm previous findings indicating that cholesterol esters comprise the majority of total cholesterol in lipoproteins (39). Study on older adults participants investigating associations between pesticide p, p’-DDE and circulating metabolites, has reported the interference of p,p’-DDE in alterations of lipoprotein metabolites (17).

In both women and men, increased concentrations of extremely large size VLDL lipoproteins in cholesterol esters with pesticide exposures were found. Moreover, this association was seen for several different sizes of the very-low-density lipoprotein in women, in particular. However, in women, pesticide exposure was related to decreased concentrations of HDL in cholesterol esters. These differences are consistent with higher disturbances of cholesterol metabolism in women than those in men upon exposure to pesticides. Other lipoprotein categories, such as total lipoproteins (P), free cholesterol (FC), total lipids (L), triglycerides (TG), and total cholesterol (C), also show this gender difference, with negative relationships between non-occupational pesticide exposure and HDL found in women but not in males. Our study results show low doses of pesticide exposures affect multiple subclasses of circulating metabolites, which could contribute to identification of early biomarkers of T2D and CVD, and that women are at higher risk than men. Furthermore, it is possible that pesticide exposures may contribute to the exacerbation of metabolic disorders through their positive association with specific lipoprotein groups and the related pathways, with these associations differing by sex.

### Apolipoprotein

Our study found that various pesticide exposure scenarios, including overall exposure, months of exposure, and use of specific pesticides, were associated with decreased concentrations of apolipoprotein A1 and an increased apo B/apo A1 ratio in women (Figure 1). The apo B/apo A1 ratio reflects the balance between atherogenic and antiatherogenic particles and serves as a marker of CVD risk (40–42). An increase in the apo B/apo A1 ratio with exposure to pesticides could indicate a potential biomarker for future cardiometabolic risk and other clinical outcomes.

### Glycolysis-related metabolites

In men, overall pesticide exposure was associated with decreased concentrations of lactate. Different pesticide exposure scenarios were associated with increased glycerol (overall pesticide exposure, specific pesticides use) and citrate concentrations (years of pesticide exposure) in women. Changes in multiple intermediary metabolites (lactate, citrate, glycerol) related to glycolysis-related metabolism in relation to different pesticide exposure scenarios were observed (Figure 1). The Krebs cycle, also known as the tricarboxylic acid (TCA) cycle, is a fundamental metabolic pathway that governs numerous cellular functions and influences cell fate in humans. The metabolites and intermediates generated during the Krebs cycle are critical building blocks for the production of macromolecules, including lipids, nucleotides, and proteins (43). The changes in citrate concentrations due to exposure to pesticides may indicate a disruption in the Krebs cycle and a potential impact on the consumption of citrate, a precursor for the synthesis of macromolecules such as lipids, nucleotides, and proteins. Furthermore, decrease in lactate concentrations also suggests that pesticides may alter the energy metabolism and interfere with glucose homeostasis, potentially contributing to the development of T2D (44, 45) and obesity (46). In addition, some pesticides are reported to inhibit the enzyme lactate dehydrogenase (47).

### Ketone bodies

Overall pesticide exposure and years of pesticide exposure was associated with change in acetate concentrations in men. Different pesticide categories were associated with decreased concentrations of acetoacetate (overall, years of exposure and specific pesticides use) and beta-hydroxy butyrate (specific pesticides use; Figure 1). Experimental investigations show pesticide exposures to be associated with different adverse mechanisms (i) an increase in intermediary metabolites of TCA cycle, and (ii) elevated concentrations of ketone bodies, which indicate inhibition of acetyl-CoA, ultimately leading to disruptions in liver energy and fatty acid metabolism (48, 49). In addition, the observed alteration in beta-hydroxybutyrate concentrations, a product of liver fatty acid oxidation, may suggest impaired liver function (48, 50).

### Branched chain amino acids

Overall pesticide exposure and years of pesticide exposure was negatively associated with valine and isoleucine in men (Figure 1). Dietary intake is a major determinant of circulating levels of branched-chain amino acids (BCAAs). BCAAs play a crucial role in providing nitrogen for the synthesis of glutamate, which is the primary excitatory neurotransmitter in the brain. Alterations in BCAA metabolism have been suggested to accompany the development of Alzheimer’s disease and incident dementia (51–53). Furthermore, experimental studies in mice exposed to organophosphorus pesticides have shown links between long-term pesticide exposure and disrupted amino acid metabolism (54). The present study included participants who reported use of herbicides and pesticides including glyphosate, malathion, deltamethrin, permethrin, cypermethrin, triadimefon which are reported to inhibit the activity of enzymes in metabolic pathways and alterations in amino acid metabolism (55). A study on 22,632 participants including eight prospective cohorts has reported that these metabolites may be utilized as early markers of mild cognitive impairment resulting in incident dementia and future risk of Alzheimer’s disease (53). Moreover, lifelong cumulative pesticide exposure is reported to be associated with nervous system disorders and development of Alzheimer’s disease (4). In a systematic review conducted on studies published between 1963 and 2010 that examined the effects of organophosphate and carbamate pesticides, it was found that these pesticides can affect enzymatic pathways involved in the metabolism of proteins, fats and carbohydrates within mitochondria, peroxisomes, and cytoplasm (56). Disturbances in amino acid metabolism may serve as a useful marker of low-level pesticide exposure in the general population, based on these findings.

### Fluid balance and inflammation

Our study found that various pesticide exposure scenarios, including overall exposure, years of exposure, and use of specific pesticides, were associated with decreased albumin concentrations in women (Figure 1). A significant proportion of the circulating proteins in our body is composed of serum albumin. A decrease in its concentration has been reported to have an independent association with the risk of cardiovascular diseases (57, 58). Furthermore, the reported use of specific pesticides also included organophosphorus and other pesticides, and albumin levels could potentially serve as a biomarker for monitoring exposure to multiple pesticides at low levels (23, 59). In addition, a negative association was observed between specific pesticides exposure and alpha-1-acid glycoprotein, a novel biomarker of systemic inflammation and cardiovascular disease risk, in men (60).

The associations observed between multiple metabolomics biomarkers and short-duration (in months, PEM, Table 2) pesticide exposure in women, as well as the associations with a few metabolites and long-duration (in years, PEY, Table 3) pesticide exposure in both men and women, highlight the complex relationship between low-dose pesticide exposures and health effects. Low-dose exposure to pesticides, even below regulatory safety thresholds, has been a subject of concern due to the potential for cumulative effects and the disruption of biological processes. Some studies suggest that chronic low-dose exposures to pesticides may contribute to adverse health outcomes, including developmental, reproductive, and neurocognitive effects, as well as an increased risk of certain chronic diseases (61–63).

Sex differences in low-dose pesticide exposure effects are an emerging area of research. Biological and physiological differences between men and women can lead to varying responses to pesticide exposure (63, 64). Hormonal fluctuations, genetic variations, and metabolic differences may contribute to the sex-specific effects of pesticides (64–66). Few studies suggest that women may be more vulnerable to the effects of certain pesticides due to hormonal interactions and potential disruption of the endocrine system (63–66). However, the research on sex differences in low-dose pesticide exposure effects is still evolving, and more studies are needed to better understand the mechanisms underlying these differences and their implications for health outcomes.

### Strengths and limitations

The strengths of the study are, first, we investigated different non-occupational pesticide exposure scenarios and performed extensive characterization of lipoproteins, amino acids, and other circulating metabolites using the metabolomics approach. Secondly, we conducted a sex-stratified analysis as pesticides may affect men differently than women and some pesticides may not affect women at all (67). Thirdly, despite a relatively small sample size in our cohort for the pesticide exposure categories compared to the unexposed group, we observed independent associations between all pesticide exposure scenarios and multiple circulating metabolites. Fourth, we performed adjustment for multiple potential confounders including environmental, anthropometric, lifestyle and socioeconomic position. Despite these considerations, our study does have some limitations. Although the specific pesticide that each participant was exposed to is known, the sample size did not allow for pesticide chemical and class-specific analyses. However, the pesticides commonly reported by the exposed participants were insecticides, herbicides, and pyrethroids, which were typically used in agricultural work at the time. Our reported use of specific pesticide exposure variable allowed us to distinguish the effects of exposure to a complex mixture of pesticides in our study. To identify early biomarkers of disease risk specific to different pesticide classes, future research on larger samples is needed. Additionally, our study may have limited generalizability since it only included individuals of Finnish ethnicity.

## Conclusion

We observed that non-occupational pesticide exposure scenarios led to alterations and disturbances in the serum metabolomic biomarkers in the Finnish adult population. In women, all types of pesticide exposure were associated with decreased HDL concentrations in all lipoprotein subclasses, albumin, apo A1 and increased apo B/apoA1 ratio. In men, all categories of pesticide exposure were associated with decreases in branched-chain amino acid concentrations and specific pesticide exposure was negatively associated with sphingolipids and inflammatory biomarkers. Both sexes showed significant changes in glycolysis-related metabolites and ketone bodies in relation to pesticide exposures. The observed changes in the serum metabolome could potentially provide insight into the underlying biological mechanisms or pathways that contribute to the development of non-communicable diseases. According to these results, non-occupational exposure to pesticide implies a higher risk in women.

## Data availability statement

The datasets presented in this article are not readily available because NFBC data is available from the University of Oulu, Infrastructure for Population Studies. Permission to use the data can be applied for research purposes *via* an electronic material request portal. In the use of data, we follow the EU general data protection regulation (679/2016) and Finnish Data Protection Act. The use of personal data is based on cohort participant’s written informed consent at his/her latest follow-up study, which may cause limitations to its use. Please, contact the NFBC project center (NFBCprojectcenter@oulu.fi) and visit the cohort website (www.oulu.fi/nfbc) or Fairdata.fi (http://urn.fi/urn:nbn:fi:att:bc1e5408-980e-4a62-b899-43bec3755243) for additional information. Requests to access the datasets should be directed to NFBC project center (NFBCprojectcenter@oulu.fi).

## Ethics statement

The studies involving humans were approved by Participants who took part in the study provided their written consent to utilize their data. All activities were performed in compliance with the 1964 Declaration of Helsinki. The Northern Ostrobothnia Hospital District’s ethics committee gave the NFBC1966 study their approval. The studies were conducted in accordance with the local legislation and institutional requirements. The participants provided their written informed consent to participate in this study.

## Author contributions

SP: conceptualization, data curation, methodology, statistical analysis, validation, writing – original draft, and writing – review and editing. AR and M-RJ: conceptualization and writing – review and editing. KA, JR, JO, and JG: writing – review and editing. All authors contributed to the article and approved the submitted version.

## Abbreviations

Apo, apolipoprotein
BMI, body mass index
C, total cholesterol
CE, cholesterol ester
CVD, cardiovascular disease
EDCs, endocrine-disrupting chemicals
FC, free cholesterol
HDL, high-density lipoprotein
IDL, intermediate-density lipoprotein
L, large size of lipoproteins
LDL, low-density lipoprotein
L, total lipids component
M, medium size of lipoprotein
NFBC1966, Northern Finland Birth Cohort 1966
NMR, nuclear magnetic resonance
OPE, overall pesticide exposure
P, total lipoproteins
PEM, pesticide exposure in months
PEU, specific pesticides
PEY, pesticide exposure in years
PL, phospholipids
SEP, socioeconomic position
S, small
TG, triglycerides
T2D, type 2 diabetes
VLDL, very low-density lipoprotein
XS, very-small size of lipoprotein
XL, very-large size of lipoprotein
XXL, extremely large size of lipoprotein
