# Supplementary material for: Investigating the relationship between non-occupational pesticide exposure and metabolomic biomarkers
Source: Front Public Health. 2023 Oct 11;11:1248609. doi: 10.3389/fpubh.2023.1248609 (PMC10602903; doi:10.3389/fpubh.2023.1248609)
Supplement: Supplementary file 1 [file Data_Sheet_1.docx]

**Online Supplementary material**

**Investigating the relationship between non-occupational pesticide exposure and metabolomic biomarkers**

Saranya Palaniswamy^1,2,3^, Khaled Abass^3,4^, Jaana Rysä^5^, Joan O. Grimalt^6^, Jon Øyvind Odland^7,8^, Arja Rautio^4,9*,^ Marjo-Riitta Järvelin^1,2,10,11,12*^

*Shared last authors

**Affiliations:**

1. Center for Life Course Health Research, Faculty of Medicine, University of Oulu, Oulu, Finland.

2. Department of Epidemiology and Biostatistics, School of Public Health, Imperial College London, London, United Kingdom.

3. Arctic Health, Faculty of Medicine, University of Oulu, Oulu, Finland.

4. Department of Environmental Health Sciences, College of Health Sciences, University of Sharjah, UAE.

5. School of Pharmacy, University of Eastern Finland, Kuopio, Finland.

6. Institute of Environmental Assessment and Water Research (IDAEA), Spanish Council for Scientific Research (CSIC), Barcelona, Catalonia, Spain.

7. NTNU The Norwegian University of Science and Technology, Trondheim, Norway.

8. School of Health Systems and Public Health, Faculty of Health Sciences, University of Pretoria, Pretoria, South Africa.

9. Thule Institute, University of Arctic, University of Oulu, Oulu, Finland.

10. Unit of Primary Care, Oulu University Hospital, Oulu, Finland.

11. MRC-PHE Centre for Environment and Health, School of Public Health, Imperial College London, London, United Kingdom.

12. Department of Life Sciences, College of Health and Life Sciences, Brunel University London, London, United Kingdom.

**Corresponding author:**

Dr. Saranya Palaniswamy, Center for Life Course Health Research, Faculty of Medicine, University of Oulu, Oulu, Finland. Email: [saranya.palaniswamy@oulu.fi](mailto:saranya.palaniswamy@oulu.fi);

**Supplementary methods:**

**Appendix 1.** Detailed description of the cohort and covariates from the NFBC1966

**Appendix 2.** Exposure assessment

**Appendix 3.** Outcome assessment (metabolomics biomarkers)

**Appendix 4.** Abbreviation of the metabolites measured in the NFBC1966 study

**Supplementary figures:**

**Figure S1.** A flowchart outlining the study population (NFBC1966)

**Supplementary tables:**

**Table S1.** Reported pesticides and plant protection products used by the NFBC1966 study population.

**Table S2.** Current use of different groups of pesticides in the NFBC1966 study population

**Table S3.** Descriptive statistics of the study population in NFBC1966 with overall pesticide exposure

**Table S4.** Descriptive statistics of the study population with pesticide exposure in months, in years and specific pesticides use in NFBC1966.

**Table S5.** Spearman correlation analyses on the association between pesticide exposure in months, years, specific pesticides, and pesticide exposure overall with circulating metabolites

**Table S6.** Multivariable regression analyses of pesticide exposure overall with total lipoprotein subclasses with standardised metabolomic biomarkers in men

**Table S7.** Multivariable regression analyses of pesticide exposure overall with total lipoprotein subclasses with standardised metabolomic biomarkers in women

**Table S8.** Multivariable regression analyses of pesticide exposure overall with triglycerides subclasses with standardised metabolomic biomarkers in men

**Table S9.** Multivariable regression analyses of pesticide exposure overall with triglycerides subclasses with standardised metabolomic biomarkers in women

**Table S10.** Multivariable regression analyses of pesticide exposure overall with phospholipid subclasses with standardised metabolomic biomarkers in men

**Table S11.** Multivariable regression analyses of pesticide exposure overall with phospholipid subclasses with standardised metabolomic biomarkers in women

**Table S12.** Multivariable regression analyses of pesticide exposure overall with cholesterol esters subclasses with standardised metabolomic biomarkers in men

**Table S13.** Multivariable regression analyses of pesticide exposure overall with cholesterol esters subclasses with standardised metabolomic biomarkers in women

**Table S14.** Multivariable regression analyses of pesticide exposure overall with free cholesterol subclasses with standardised metabolomic biomarkers in men

**Table S15.** Multivariable regression analyses of pesticide exposure overall with free cholesterol subclasses with standardised metabolomic biomarkers in women

**Table S16.** Multivariable regression analyses of pesticide exposure overall with total lipid subclasses with standardised metabolomic biomarkers in men

**Table S17.** Multivariable regression analyses of pesticide exposure overall with total lipid subclasses with standardised metabolomic biomarkers in women

**Table S18.** Multivariable regression analyses of pesticide exposure overall with total cholesterol subclasses with standardised metabolomic biomarkers in men

**Table S19.** Multivariable regression analyses of pesticide exposure overall with total cholesterol subclasses with standardised metabolomic biomarkers in women

**Table S20.** Multivariable regression analyses of pesticide exposure overall with apolipoproteins, aminoacids, sphingomyelins, glucose metabolism, fatty acids, inflammation, and fluid balance with standardised metabolomic biomarkers in men

**Table S21.** Multivariable regression analyses of pesticide exposure overall with apolipoproteins, aminoacids, sphingomyelins, glucose metabolism, fatty acids, inflammation, and fluid balance with standardised metabolomic biomarkers in women

**Table S22.** Multivariable regression analyses of pesticide exposure in months with total lipoprotein subclasses with standardised metabolomic biomarkers in men

**Table S23.** Multivariable regression analyses of pesticide exposure in months with total lipoprotein subclasses with standardised metabolomic biomarkers in women

**Table S24.** Multivariable regression analyses of pesticide exposure in months with triglycerides subclasses with standardised metabolomic biomarkers in men

**Table S25.** Multivariable regression analyses of pesticide exposure in months with triglycerides subclasses with standardised metabolomic biomarkers in women

**Table S26.** Multivariable regression analyses of pesticide exposure in months with phospholipid subclasses with standardised metabolomic biomarkers in men

**Table S27.** Multivariable regression analyses of pesticide exposure in months with phospholipid subclasses with standardised metabolomic biomarkers in women

**Table S28.** Multivariable regression analyses of pesticide exposure in months with cholesterol esters subclasses with standardised metabolomic biomarkers in men

**Table S29.** Multivariable regression analyses of pesticide exposure in months with cholesterol esters subclasses with standardised metabolomic biomarkers in women

**Table S30.** Multivariable regression analyses of pesticide exposure in months with free cholesterol subclasses with standardised metabolomic biomarkers in men

**Table S31.** Multivariable regression analyses of pesticide exposure in months with free cholesterol subclasses with standardised metabolomic biomarkers in women

**Table S32.** Multivariable regression analyses of pesticide exposure in months with total lipid subclasses with standardised metabolomic biomarkers in men

**Table S33.** Multivariable regression analyses of pesticide exposure in months with total lipid subclasses with standardised metabolomic biomarkers in women

**Table S34.** Multivariable regression analyses of pesticide exposure in months with total cholesterol subclasses with standardised metabolomic biomarkers in men

**Table S35.** Multivariable regression analyses of pesticide exposure in months with total cholesterol subclasses with standardised metabolomic biomarkers in women

**Table S36.** Multivariable regression analyses of pesticide exposure in months with apolipoproteins, aminoacids, sphingomyelins, glucose metabolism, fatty acids, inflammation, and fluid balance with standardised metabolomic biomarkers in men

**Table S37.** Multivariable regression analyses of pesticide exposure in months with apolipoproteins, aminoacids, sphingomyelins, glucose metabolism, fatty acids, inflammation, and fluid balance with standardised metabolomic biomarkers in women

**Table S38.** Multivariable regression analyses of pesticide exposure in years with total lipoprotein subclasses with standardised metabolomic biomarkers in men

**Table S39.** Multivariable regression analyses of pesticide exposure in years with total lipoprotein subclasses with standardised metabolomic biomarkers in women

**Table S40.** Multivariable regression analyses of pesticide exposure in years with triglycerides subclasses with standardised metabolomic biomarkers in men

**Table S41.** Multivariable regression analyses of pesticide exposure in year**s** with triglycerides subclasses with standardised metabolomic biomarkers in women

**Table S42.** Multivariable regression analyses of pesticide exposure in years with phospholipid subclasses with standardised metabolomic biomarkers in men

**Table S43.** Multivariable regression analyses of pesticide exposure in years with phospholipid subclasses with standardised metabolomic biomarkers in women

**Table S44.** Multivariable regression analyses of pesticide exposure in years with cholesterol esters subclasses with standardised metabolomic biomarkers in men

**Table S45.** Multivariable regression analyses of pesticide exposure in years with cholesterol esters subclasses with standardised metabolomic biomarkers in women

**Table S46.** Multivariable regression analyses of pesticide exposure in months with free cholesterol subclasses with standardised metabolomic biomarkers in men

**Table S47.** Multivariable regression analyses of pesticide exposure in years with free cholesterol subclasses with standardised metabolomic biomarkers in women

**Table S48.** Multivariable regression analyses of pesticide exposure in years with total lipid subclasses with standardised metabolomic biomarkers in men

**Table S49.** Multivariable regression analyses of pesticide exposure in years with total lipid subclasses with standardised metabolomic biomarkers in women

**Table S50.** Multivariable regression analyses of pesticide exposure in years with total cholesterol subclasses with standardised metabolomic biomarkers in men

**Table S51.** Multivariable regression analyses of pesticide exposure in years with total cholesterol subclasses with standardised metabolomic biomarkers in women

**Table S52.** Multivariable regression analyses of pesticide exposure in years with apolipoproteins, aminoacids, sphingomyelins, glucose metabolism, fatty acids, inflammation, and fluid balance with standardised metabolomic biomarkers in men

**Table S53.** Multivariable regression analyses of pesticide exposure in years with apolipoproteins, aminoacids, sphingomyelins, glucose metabolism, fatty acids, inflammation, and fluid balance with standardised metabolomic biomarkers in women

**Table S54.** Multivariable regression analyses of specific pesticides use with total lipoprotein subclasses with standardised metabolomic biomarkers in men

**Table S55.** Multivariable regression analyses of specific pesticides use with total lipoprotein subclasses with standardised metabolomic biomarkers in women

**Table S56.** Multivariable regression analyses of specific pesticides use with triglycerides subclasses with standardised metabolomic biomarkers in men

**Table S57.** Multivariable regression analyses of specific pesticides use with triglycerides subclasses with standardised metabolomic biomarkers in women

**Table S58.** Multivariable regression analyses of specific pesticides use with phospholipid subclasses with standardised metabolomic biomarkers in men

**Table S59.** Multivariable regression analyses of specific pesticides use with phospholipid subclasses with standardised metabolomic biomarkers in women

**Table S60.** Multivariable regression analyses of specific pesticides use with cholesterol esters subclasses with standardised metabolomic biomarkers in men

**Table S61.** Multivariable regression analyses of specific pesticides use with cholesterol esters subclasses with standardised metabolomic biomarkers in women

**Table S62.** Multivariable regression analyses of specific pesticides use with free cholesterol subclasses with standardised metabolomic biomarkers in men

**Table S63.** Multivariable regression analyses of specific pesticides use with free cholesterol subclasses with standardised metabolomic biomarkers in women

**Table S64.** Multivariable regression analyses of specific pesticides use with total lipid subclasses with standardised metabolomic biomarkers in men

**Table S65.** Multivariable regression analyses of specific pesticides use with total lipid subclasses with standardised metabolomic biomarkers in women

**Table S66.** Multivariable regression analyses of specific pesticides use with total cholesterol subclasses with standardised metabolomic biomarkers in men

**Table S67.** Multivariable regression analyses of specific pesticides use with total cholesterol subclasses with standardised metabolomic biomarkers in women

**Table S68.** Multivariable regression analyses of specific pesticides use with apolipoproteins, aminoacids, sphingomyelins, glucose metabolism, fatty acids, inflammation, and fluid balance with standardised metabolomic biomarkers in men

**Table S69.** Multivariable regression analyses of specific pesticides use with apolipoproteins, aminoacids, sphingomyelins, glucose metabolism, fatty acids, inflammation, and fluid balance with standardised metabolomic biomarkers in women

**Appendix 1. Detailed description of the cohort and covariates from the NFBC1966**

**Northern Finland Birth Cohort 1966 (NFBC1966)**: The NFBC1966 is a population-based, homogeneous, longitudinal birth cohort study comprised of offspring of pregnant women (n=12,055) with expected delivery dates during 1966, residing in two of the northernmost provinces of Finland (Oulu and Lapland)^1^. There were 12,058 live births, and these children were followed-up until the age of 46 years (Nordström et al., 2021). In 1997, at 31-years of age, participants’ health, lifestyle, and occupation were assessed by postal questionnaires (n=11,541; 97% of the birth cohort alive and traced) (University of Oulu). Participants who responded (n=8,463) and resided in Northern Finland or in the Helsinki area were invited for a clinical examination (n=6,033). The current study is based on 31-year-old NFBC1966 participants. Study participants gave written informed consent for their data usage. All procedures performed were in accordance with the 1964 Declaration of Helsinki. The Ethics Committee of the Northern Ostrobothnia Hospital District has approved the NFBC1966 study. The flowchart of the study population is shown in **Figure S1**.

**Body mass index (BMI):** During clinical examination, height and weight measurements were used to calculate BMI (kg/m^2^). BMI was then categorized as a dichotomous variable as ≤ 24.99 (underweight and normal weight) and > 24.99 (overweight and obese).

**Season of blood sampling:** The season of participant attendance at the clinical assessment was categorised according to the Finnish Meteorological Institute standard as high pesticide use season (summer (1 June–30 August) autumn (1 September–31 October)) and low pesticide use season (winter (1 November–31 March) spring (1 April–31 May)) ^2^.

The remaining covariates latitude of residence, socioeconomic position, educational status, lifestyle covariates (smoking, alcohol, physical activity) and dietary index were assessed by postal questionnaire responses at 31-years.

**Latitude of residence**: The residence of the participants at age 31 years was collected from the population register office. They were categorised as residing in Helsinki (60°N); the city of Oulu (65°N) and elsewhere in northernmost provinces of Oulu and Lapland (>65°N).

**Socioeconomic position (SEP):** SEP was categorised as I and II (professional), III (skilled worker), IV (unskilled worker), V (farmer) and VI (others-pensioner, student, long-term unemployed or not defined).

**Educational status:** Level of education was categorised into three options as i) Under 9 years of basic school education, ii) Basic school from 7-16 years old, and iii) Matriculation examination attended at 18 years old.

**Smoking:** The postal questionnaire asked participants about their smoking history, frequency, duration, and type of products smoked. Current smoking was then categorised as non-smokers (non-smoker and former/occasional) and smokers (active smoker) in the current study.

**Alcohol consumption**: Alcohol consumption during the 6 months prior to the questionnaire was calculated as grams per day (g/day) and has been described elsewhere ^3^. It was further categorised according to WHO sex-specific classification as abstainer, low-risk drinker (≤20 and ≤40 g/day for women and men, respectively) or at-risk drinker (>20 and >40 g/day for women and men, respectively) ^4^.

**Physical activity:** The reported frequency and duration of leisure time and brisk physical activity were used to calculate the metabolic equivalent of task (MET) scores in hours per week, and these were ordered into quartiles. An intensity value of 3 METs is considered as light physical activity, and 5 METs as brisk physical activity ^5^.

**Habitual dietary index:** Diet score was calculated based on the consumption of various food in the previous 6 months and was reported on a structured six-point scale (from never/<once per month to several times per day) and has been described previously. The food frequency question included 32 products categorised under grain products, milk products, vegetables, meat, fruits and others (chocolates, sweets and packaged meals). An unhealthy diet included daily or frequent consumption of red meat and less frequent consumption of rye or crisp bread, berries or fruit, salads and vegetables. The score ranged from 0–5 and was categorised as healthy diet (<3 points) and unhealthy diet (4–5 points) ^2^.

**References:**

1. University of Oulu. Northern Finland Birth Cohort 1966. University of Oulu. http://urn.fi/urn:nbn:fi:att:bc1e5408-980e-4a62-b899-43bec3755243
2. Finnish Meteorological Institute. Seasons in Finland. 2016.

<http://en.ilmatieteenlaitos.fi/seasons-in-finland> (accessed 20 October 2020).

1. Fawehinmi TO, Ilomäki J, Voutilainen S, Kauhanen J. Alcohol Consumption and Dietary Patterns: The FinDrink Study. *PLoS ONE*. 2012;7(6):e38607. doi:10.1371/journal.pone.0038607
2. Laitinen J, Pietiläinen K, Wadsworth M, Sovio U, Järvelin MR. Predictors of abdominal obesity among 31-y-old men and women born in Northern Finland in 1966. *European Journal of Clinical Nutrition*. Published online 2004. doi:10.1038/sj.ejcn.1601765
3. Nordström, T., Miettunen, J., Auvinen, J., Ala-Mursula, L., Keinänen-Kiukaanniemi, S., Veijola, J., Järvelin, M.-R., Sebert, S., Männikkö, M., 2021. Cohort Profile: 46 years of follow-up of the Northern Finland Birth Cohort 1966 (NFBC1966). Int J Epidemiol. https://doi.org/10.1093/ije/dyab109
4. Suija K, Timonen M, Suviola M, Jokelainen J, Järvelin MR, Tammelin T. The association between physical fitness and depressive symptoms among young adults: Results of the Northern Finland 1966 birth cohort study. *BMC Public Health*. Published online 2013. doi:10.1186/1471-2458-13-535

**Appendix 2. Exposure assessment**

**Pesticide exposure scenarios – OPE: overall pesticide exposure; PEM: pesticide exposure in months; PEY: pesticide exposure in years; PEU: specific pesticides use reported.**

Participants received a postal questionnaire at 31-years which included general health questions on lifestyle, environment, exposure to chemicals including pesticides. The questions included “How long have you been exposed to the following substance - pesticides and plant protection products in months (1-12 months) and in years (one to multiple years)?”. In addition, “what type of pesticide and plant protection products (to specify the name of the pesticide used) were used” was asked simultaneously. Participants who reported no pesticide exposure at all were categorized as “no” (non-exposed), and those who reported pesticides exposures from 1 to 12 months were grouped as pesticide exposure in months (PEM); or from one to multiple years were grouped as pesticide exposure in years (PEY) as “yes” (exposed). The name of pesticides and plant protection products used by NFBC1966 participants is shown in **Supplementary Table S1**. Participants responses were categorized as pyrethroids, organophosphates, insect repellents, herbicides, fungicides, and plant growth regulators (Table S2). Due to the limited number, participants who reported no use of any pesticide were categorized as “no” (non-exposed), and those who reported specific name of pesticide used by them in **Supplementary Table S2** were merged together as “yes” (exposed). The overall pesticide exposure (OPE) variable combines the participants who reported pesticide exposures during months or in years or reporting the name of pesticides used. So, in NFBC1966, four pesticide exposure metrics [overall pesticide exposure (OPE), months of pesticide exposure (PEM), years of pesticide exposure (PEY) and specific pesticides (PEU)] were used to model exposure-metabolomics biomarkers associations. PEM and PEY were explored separately from overall exposure to assess the severity of exposure, and PEU was also evaluated for accuracy of reporting. People who reported using specific pesticides (PEY) was highly positively correlated with people reporting pesticide exposure in months PEM (r = 0.488; p < 0.0001) and/or in years PEY (r = 0.415; p < 0.0001). The correlation coefficients show the reliability of using different pesticide exposures in the current study.

**Blood sample measurements:** After completion of postal questionnaire, NFBC1966 participants were invited for a clinical examination and blood samples were taken after an overnight fasting period from 8 to 11 hours, centrifuged immediately, and stored first at −20°C and later at −80°C. Blood samples were analyzed at NordLab Oulu (former name Oulu University Hospital, Laboratory), a testing laboratory (T113) accredited by the Finnish Accreditation Service (FINAS) (EN ISO 15189).

**Appendix 3. Outcome assessment (metabolomics biomarkers):** The quantification of the serum metabolomics biomarkers was performed by Proton Nuclear Magnetic Resonance (NMR) as described in Würtz et al., 2015. The metabolomics platform is based on three molecular windows (lipoprotein, low-molecular-weight metabolite data, and lipid) for each sample (Würtz et al., 2015). Subclasses of lipoproteins were defined as follows: six very large (XXL) very low-density lipoprotein (VLDL) subclasses greater than 75 nm – 31.3 nm in diameter, intermediate-density lipoprotein (IDL) at a diameter of 28.6 nm, three low-density lipoprotein (LDL) subclasses between 25.5 nm – 18.7 nm in diameter, and five high-density lipoprotein (HDL) subclasses between 14.3 nm – 8.7 nm in diameter. Lipoprotein components including triglycerides (TG), phospholipids (PL), cholesterol esters (CE), free cholesterol (FC), total lipids (L), and total cholesterol (C) were included in the quantification. In addition, apolipoproteins, amino acids including branched-chain and aromatic amino acids, ketone bodies, sphingolipids, glycolysis-related metabolites, fatty acids, inflammation, and fluid balance were assessed. The detailed list of names and abbreviations of circulating metabolites is given in the online supplementary material.

**References:**

1. Würtz, P., Havulinna, A.S., Soininen, P., Tynkkynen, T., Prieto-Merino, D., Tillin, T., Ghorbani, A., Artati, A., Wang, Q., Tiainen, M., Kangas, A.J., Kettunen, J., Kaikkonen, J., Mikkilä, V., Jula, A., Kähönen, M., Lehtimäki, T., Lawlor, D.A., Gaunt, T.R., Hughes, A.D., Sattar, N., Illig, T., Adamski, J., Wang, T.J., Perola, M., Ripatti, S., Vasan, R.S., Raitakari, O.T., Gerszten, R.E., Casas, J.-P., Chaturvedi, N., Ala-Korpela, M., Salomaa, V., 2015a. Metabolite Profiling and Cardiovascular Event Risk. Circulation 131, 774–785. https://doi.org/10.1161/CIRCULATIONAHA.114.013116

**Appendix 4. Abbreviation of the metabolites measured in the NFBC1966 study**

| **Total lipoproteins (P)** | **Total lipids (L)** |
| --- | --- |
| Concentration of chylomicrons and extremely large VLDL particles [mol/L], XXL_VLDL_P | Total lipids in chylomicrons and extremely large VLDL [mmol/L], XXL_VLDL_L |
| Very-large VLDL [mol/L], XL_VLDL_P | Very-large VLDL [mmol/L], XL_VLDL_L |
| Large VLDL [mol/L], L_VLDL_P | Large VLDL [mmol/L], L_VLDL_L |
| Medium VLDL [mol/L], M_VLDL_P | Medium VLDL [mmol/L], M_VLDL_L |
| Small VLDL [mol/L], S_VLDL_P | Small VLDL [mmol/L], S_VLDL_L |
| Very-small VLDL [mol/L], XS_VLDL_P | Very-small VLDL [mmol/L], XS_VLDL_L |
| IDL [mol/L], IDL_P | IDL [mmol/L], IDL_L |
| Large LDL [mol/L], L_LDL_P | Large LDL [mmol/L], L_LDL_L |
| Medium LDL [mol/L], M_LDL_P | Medium LDL [mmol/L], M_LDL_L |
| Small LDL [mol/L], S_LDL_P | Small LDL [mmol/L], S_LDL_L |
| Very-large HDL [mol/L], XL_HDL_P | Very-large HDL [mmol/L], XL_HDL_L |
| Large HDL [mol/L], L_HDL_P | Large HDL [mmol/L], L_HDL_L |
| Medium HDL [mol/L], M_HDL_P | Medium HDL [mmol/L], M_HDL_L |
| Small HDL [mol/L], S_HDL_P | Small HDL [mmol/L], S_HDL_L |
| **Triglycerides (TG)** | **Total cholesterol (C)** |
| Triglycerides in chylomicrons and extremely large VLDL [mmol/L], XXL_VLDL_TG | Total cholesterol in chylomicrons and extremely large VLDL [mmol/L], XXL_VLDL_C |
| Very-large VLDL [mmol/L], XL_VLDL_TG | Very-large VLDL [mmol/L], XL_VLDL_C |
| Large VLDL [mmol/L], L_VLDL_TG | Large VLDL [mmol/L], L_VLDL_C |
| Medium VLDL [mmol/L], M_VLDL_TG | Medium VLDL [mmol/L], M_VLDL_C |
| Small VLDL [mmol/L], S_VLDL_TG | Small VLDL [mmol/L], S_VLDL_C |
| Very-small VLDL [mmol/L], XS_VLDL_TG | Very-small VLDL [mmol/L], XS_VLDL_C |
| Total VLDL [mmol/L], VLDL_TG | IDL [mmol/L], IDL_C |
| IDL [mmol/L], IDL_TG | Large LDL [mmol/L], L_LDL_C |
| Large LDL [mmol/L], L_LDL_TG | Medium LDL [mmol/L], M_LDL_C |
| Medium LDL [mmol/L], M_LDL_TG | Small LDL [mmol/L], S_LDL_C |
| Small LDL [mmol/L], S_LDL_TG | Total LDL [mmol/L], HDL_C |
| Total LDL [mmol/L], LDL_TG | Very-large HDL [mmol/L], XL_HDL_C |
| Very-large HDL [mmol/L], XL_HDL_TG | Large HDL [mmol/L], L_HDL_C |
| Large HDL [mmol/L], L_HDL_TG | Medium HDL [mmol/L], M_HDL_C |
| Medium HDL [mmol/L], M_HDL_TG | Small HDL [mmol/L], S_HDL_C |
| Small HDL [mmol/L], S_HDL_TG | Total HDL [mmol/L], HDL_C |
| Total HDL [mmol/L], HDL_TG | Serum cholesterol [mmol/L] |
| Serum TG [mmol/L], Serum TG | Remnant cholesterol [mmol/L] |
| **Phospholipids (PL)** |  |
| Phospholipids in chylomicrons and extremely large VLDL [mmol/L], XXL_VLDL_PL | **Apolipoproteins** |
| Very-large VLDL [mmol/L], XL_VLDL_PL | Apolipoprotein A1 [g/L] |
| Large VLDL [mmol/L], L_VLDL_PL | Apolipoprotein B [g/L] |
| Medium VLDL [mmol/L], M_VLDL_PL | Ratio of Apolipoprotein B/Apolipoprotein A1 (Apo B/ Apo A1 ratio) |
| Small VLDL [mmol/L], S_VLDL_PL |  |
| Very-small VLDL [mmol/L], XS_VLDL_PL | **Amino acids** |
| IDL [mmol/L], IDL_PL | Alanine [µmol/L] |
| Large LDL [mmol/L], L_LDL_PL | Glutamine [µmol/L] |
| Medium LDL [mmol/L], M_LDL_PL | Glycine [µmol/L] |
| Small LDL [mmol/L], S_LDL_PL | Histidine [µmol/L] |
| Very-large HDL [mmol/L], XL_HDL_PL | **Branched-chain amino acids** |
| Large HDL [mmol/L], L_HDL_PL | Isoleucine [µmol/L] |
| Medium HDL [mmol/L], M_HDL_PL | Leucine [µmol/L] |
| Small HDL [mmol/L], S_HDL_PL | Valine [µmol/L] |
|  |  |
|  |  |
| **Cholesterol Esters (CE)** | **Aromatic amino acids** |
| Cholesterol esters in chylomicrons and extremely large VLDL [mmol/L], XXL_VLDL_CE | Phenylalanine [µmol/L] |
| Very-large VLDL [mmol/L], XL_VLDL_CE | Tyrosine [µmol/L] |
| Large VLDL [mmol/L], L_VLDL_CE | **Ketone bodies** |
| Medium VLDL [mmol/L], M_VLDL_CE | Acetoacetate [µmol/L] |
| Small VLDL [mmol/L], S_VLDL_CE | Acetate [µmol/L] |
| Very-small VLDL [mmol/L], XS_VLDL_CE | beta-hydroxybutyrate [µmol/L] |
| IDL [mmol/L], IDL_CE |  |
| Large LDL [mmol/L], L_LDL_CE | **Sphingolipids** |
| Medium LDL [mmol/L], M_LDL_CE | Sphingomyelin |
| Small LDL [mmol/L], S_LDL_CE | Total choline |
| Very-large HDL [mmol/L], XL_HDL_CE |  |
| Large HDL [mmol/L], L_HDL_CE | **Glycolysis related metabolites** |
| Medium HDL [mmol/L], M_HDL_CE | Citrate [µmol/L] |
| Small HDL [mmol/L], S_HDL_CE | Glucose [mmol/L] |
| Esterified cholesterol [mmol/L], EstC | Glycerol [mmol/L] |
| **Free cholesterol (FC)** | Lactate [mmol/L] |
| Free cholesterol in chylomicrons and extremely large VLDL [mmol/L], XXL_VLDL_FC | Pyruvate [mmol/L] |
| Very-large VLDL [mmol/L], XL_VLDL_FC |  |
| Large VLDL [mmol/L], L_VLDL_FC | **Fatty acids** |
| Medium VLDL [mmol/L], M_VLDL_FC | Total fatty acids [mmol/L] |
| Small VLDL [mmol/L], S_VLDL_FC | Monounsaturated fatty acids [mmol/L] |
| Very-small VLDL [mmol/L], XS_VLDL_FC | Saturated fatty acids [mmol/L] |
| IDL [mmol/L], IDL_FC |  |
| Large LDL [mmol/L], L_LDL_FC | **Inflammation** |
| Medium LDL [mmol/L], M_LDL_FC | Alpha-1-acid glycoprotein [mmol/L] |
| Small LDL [mmol/L], S_LDL_FC |  |
| Very-large HDL [mmol/L], XL_HDL_FC | **Fluid balance** |
| Large HDL [mmol/L], L_HDL_FC | Creatinine [µmol/L] |
| Medium HDL [mmol/L], M_HDL_FC | Albumin [standardized concentration units] |
| Small HDL [mmol/L], S_HDL_FC |  |
| Free cholesterol [mmol/L] |  |
|  |  |

**Supplementary Figures:**

**Figure S1. Flowchart of the study population in the NFBC1966**

**
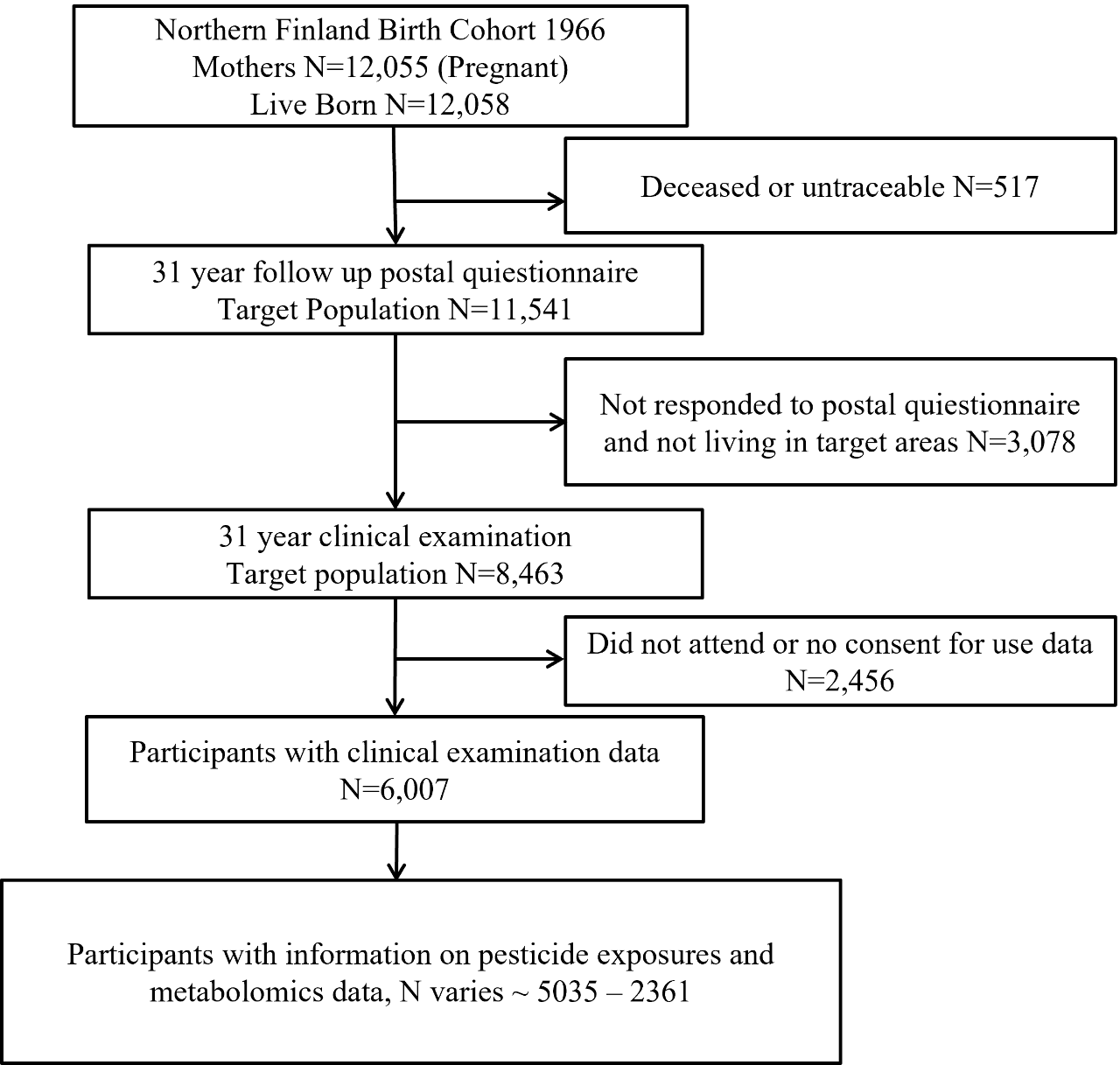
**

**Table S1. Reported pesticides and plant protection products used by the NFBC1966 study population.**

| **Pesticides common name** | **CAS** | **Type** | **Chemical group** |
| --- | --- | --- | --- |
| Cypermethrin | 52315-07-8‎ | Insecticide | Pyrethroids |
| Deltamethrin | 52918-63-5 | Insecticide | Pyrethroids |
| Dichlorvos | ‎62-73-7 | Insecticide | Organophosphate |
| Dimethoate | 60-51-5 | Insecticide | Organophosphate |
| Daminozide | ‎1596-84-5 | Plant growth regulator | *N*-(Dimethylamino)succinamic acid |
| Glyphosate | 1071-83-6 | Herbicides | N-(Phosphonomethyl)glycine |
| Lambda-cyhalothrin | 91465-08-6 | Insecticide | Pyrethroids |
| Malathion | 121-75-5 | Insecticide | Organophosphate |
| Mancozeb | 8018-01-7 | Fungicide | Dithiocarbamates |
| MCPA (2-methyl-4-chlorophenoxyacetic acid) | 94-74-6 | Herbicide | Phenoxy herbicide |
| N,N-Diethyl-meta-toluamide | 134-62-3 | Insecticide | Diethyltoluamide |
| Permethrin | 52645-53-1 | Insecticide | Pyrethroids |
| Pirimicarb | 23103-98-2 | Insecticide | Aminopyrimidine |
| Pyrethrins | 8003-34-7 | Insecticide | Pyrethroids |
| Thiram | 137-26-8 | Fungicide | Tetramethylthiuram disulfide |
| Triadimefon | 43121-43-3 | Fungicide | Triazoles |
| Triforine | 26644-46-2 | Fungicide | N-alkylpiperazines |
| Miscellaneous plant protection products |  |  |  |

**Table S2. Current use of different groups of pesticides and plant protection products in the NFBC1966 study population**

| **All pesticides** | **N (%)** |
| --- | --- |
| Pyrethroid | 46 (40.71) |
| Organophosphates | 5 (4.42) |
| Insect repellent | 32 (28.32) |
| Fungicides | 5 (4.42) |
| Herbicides | 23 (20.35) |
| Plant growth regulators | 2 (1.78) |
| **PEU (exposed) (yes)*** | **N= 113** |
| **PEU (non-exposed) (no)*** | **N= 4912** |

*Participants who reported no use of any pesticide was categorized as “no” (non-exposed) and those who reported pesticide use in Table S1 and categorized in Table S2 merged together as “yes” (exposed).

**Table S3. Descriptive statistics of the study population in NFBC1966 with overall pesticide exposure**

|  |  | **Overall pesticide exposure^**^ (OPE)** | |  |
| --- | --- | --- | --- | --- |
|  |  | No (N=4773) | Yes (N=264) | **P-value** |
| **Age** |  | 31 |  |  |
| **Sex, n %** | 5037 |  |  |  |
| Male |  | 2242 (46.97) | 180 (68.18) | <.0001 |
| Female |  | 2531 (53.03) | 84 (31.82) |  |
| **BMI (kg/m^2^), n %** | 5000 |  |  |  |
| Normal weight |  | 2857 (60.29) | 145 (55.56) | 0.129 |
| Overweight and Obese |  | 1882 (39.71) | 116 (44.44) |  |
| **Smoking status, n %** | 4966 |  |  |  |
| No smoker |  | 2099 (44.57) | 110 (42.80) | 0.578 |
| Smokers |  | 2610 (55.43) | 147 (57.20) |  |
| **Alcohol consumption (g/day), n %^a^** | 4878 |  |  |  |
| Abstainer |  | 423 (9.15) | 38 (14.96) | 0.007 |
| Low risk drinker |  | 3934 (85.08) | 205 (80.71) |  |
| At-risk drinker |  | 267 (5.77) | 11 (4.33) |  |
| **Physical activity, n %** | 4981 |  |  |  |
| Low |  | 1558 (33.00) | 99 (38.08) | 0.235 |
| Medium |  | 1626 (34.44) | 84 (32.30) |  |
| High |  | 1537 (32.56) | 77 (29.62) |  |
| **Socioeconomic position, n%** | 4972 |  |  |  |
| I + II (Professional) |  | 1175 (24.92) | 49 (18.92) | <.0001 |
| III (Skilled worker) |  | 1474 (31.26) | 50 (19.31) |  |
| IV (Unskilled worker) |  | 1202 (25.49) | 56 (21.62) |  |
| V (Farmer) |  | 112 (2.38) | 68 (26.25) |  |
| VI (Other)^b^ |  | 752 (15.95) | 36 (13.90) |  |
| **Season of pesticide use^c^, n %** | 4996 |  |  |  |
| High pesticide use season |  | 3611 (76.26) | 219 (83.91) | 0.005 |
| Low pesticide use season |  | 1124 (23.74) | 42 (16.09) |  |
| **Latitude of residence, n %** | 5037 |  |  |  |
| Oulu |  | 925 (19.38) | 27 (10.23) | <.0001 |
| Other provinces of Oulu and Lapland |  | 2998 (62.81) | 211 (79.92) |  |
| Helsinki |  | 850 (17.81) | 26 (9.85) |  |

** N varies due to missing data for some of the variables from N=5037 to N=4878.

Data are presented as n %.

P-value for differences between overall pesticide exposure with demographic, environmental, anthropometric, socioeconomic position, lifestyle (smoking, physical activity, alcohol consumption) and season of pesticide use covariates was analyzed by chi-square test and fisher test for categorical variables.

^a^Alcohol classification according to WHO sex-specific classification as abstainer, low risk drinker (≤20 g/day and ≤40 g/day for women and men, respectively) or at-risk drinker (> 20 g/day and>40 g/day for women and men, respectively).

^b^Includes students, pensioners, long-term unemployed, or not defined.

^c^High pesticide use season [summer (1 June–30 August) autumn (1 September–31 October)] and low pesticide use season [winter (1 November–31 March) spring (1 April–31 May)].

**Table S4. Descriptive statistics of the study population with pesticide exposure in months, in years and specific pesticides use in NFBC1966**

|  | **Pesticide exposure months^**^**  **(PEM)** | | **P-value** | **Pesticide exposure years^**^**  **(PEY)** | | **P-value** | **Specific pesticides****  **(PEU)** | | **P-value** |
| --- | --- | --- | --- | --- | --- | --- | --- | --- | --- |
|  | N=5017 | |  | N=5025 | |  | N=5025 | |  |
|  | No  (N=4887) | Yes  (N=130) |  | No  (N=4890) | Yes  (N=135) |  | No  (N=4912) | Yes  (N=113) |  |
| **Age** | 31 |  |  |  |  |  | 31 |  |  |
| **Sex, n %** | N=5017 |  |  | N=5025 |  |  | N=5025 |  |  |
| Male | 2327 (47.62) | 79 (60.77) | **0.0030** | 2308 (47.20) | 104 (77.04) | **<.0001** | 2344 (47.72) | 68 (60.18) | **0.0099** |
| Female | 2560 (52.38) | 51 (39.23) |  | 2582 (52.80) | 31 (22.96) |  | 2568 (52.28) | 45 (39.82) |  |
| **BMI (kg/m^2^), n (%)** | N=4980 |  |  | N=4988 |  |  | N=4988 |  |  |
| Normal weight | 2917 (60.11) | 72 (56.69) | 0.4381 | 2923 (60.23) | 71 (52.59) | 0.0739 | 2929 (60.07) | 65 (58.04) | 0.6967 |
| Overweight and Obese | 1936 (39.89) | 55 (43.31) |  | 1930 (39.77) | 64 (47.41) |  | 1947 (39.93) | 47 (41.96) |  |
| **Smoking status, n (%)** | N=4946 |  |  | N=4954 |  |  | N=4954 |  |  |
| No smoker | 2136 (44.31) | 65 (52.00) | 0.0875 | 2159 (44.78) | 46 (34.59) | **0.0196** | 2157 (44.54) | 48 (43.24) | 0.8470 |
| Smokers | 2685 (55.69) | 60 (48.00) |  | 2662 (55.22) | 87 (65.41) |  | 2686 (55.46) | 63 (56.76) |  |
| **Alcohol consumption (g/day), n (%)^a^** | N=4858 |  |  | N=4867 |  |  | N=4867 |  |  |
| Abstainer | 444 (9.38) | 15 (11.90) | 0.6337 | 438 (9.24) | 21 (16.28) | **0.0147** | 442 (9.29) | 17 (15.45) | 0.0906 |
| Low risk drinker | 4018 (84.91) | 104 (82.54) |  | 4027 (84.99) | 104 (80.62) |  | 4042 (84.97) | 89 (80.91) |  |
| At-risk drinker | 270 (5.71) | 7 (5.56) |  | 273 (5.76) | 4 (3.10) |  | 273 (5.74) | 4 (3.64) |  |
| **Physical activity, n (%)** | N=4961 |  |  | N=4969 |  |  | N=4969 |  |  |
| Low | 1605 (33.20) | 47 (37.01) | 0.6570 | 1599 (33.07) | 55 (41.04) | 0.1146 | 1614 (33.24) | 40 (35.40) | 0.8838 |
| Medium | 1661 (34.36) | 42 (33.07) |  | 1667 (34.48) | 37 (27.61) |  | 1667 (34.33) | 37 (32.74) |  |
| High | 1568 (32.44) | 38 (29.92) |  | 1569 (32.45) | 42 (31.34) |  | 1575 (32.43) | 36 (31.86) |  |
| **Socioeconomic position, n (%)** | N=4954 |  |  | N=4962 |  |  | N=4962 |  |  |
| I + II (Professional) | 1188 (24.61) | 31 (24.41) | **<.0001** | 1202 (24.89) | 20 (15.04) | **<.0001** | 1231 (25.39) | 22 (19.47) | **<.0001** |
| III (Skilled worker) | 1491 (30.89) | 32 (25.20) |  | 1504 (31.15) | 18 (13.53) |  | 1201 (24.77) | 21 (18.58) |  |
| IV (Unskilled worker) | 1231 (25.50) | 18 (14.17) |  | 1219 (25.24) | 34 (25.56) |  | 1494 (30.81) | 28 (24.78) |  |
| V (Farmer) | 148 (3.07) | 29 (22.83) |  | 136 (2.82) | 43 (32.33) |  | 152 (3.13) | 27 (23.89) |  |
| VI (Other)^b^ | 769 (15.93) | 17 (13.39) |  | 768 (15.90) | 18 (13.53) |  | 771 (15.90) | 15 (13.27) |  |
| **Season of pesticide use^c^, n (%)** | N=4976 |  |  | N=4984 |  |  | N=4984 |  |  |
| High pesticide use season | 3710 (76.51) | 104 (81.89) | 0.1683 | 3701 (76.33) | 120 (88.89) | **0.0004** | 3730 (76.54) | 91 (81.98) | 0.2117 |
| Low pesticide use season | 1139 (23.49) | 23 (18.11) |  | 1148 (23.67) | 15 (11.11) |  | 1143 (23.46) | 20 (18.02) |  |
| **Latitude of residence, n (%)** | N=5017 |  |  | N=5025 |  |  | N=5025 |  |  |
| Oulu | 930 (19.03) | 21 (16.15) | 0.2175 | 945 (19.33) | 7 (5.19) | **<.0001** | 943 (19.20) | 9 (7.96) | **0.0011** |
| Other provinces of Oulu and Lapland | 3101 (63.45) | 92 (70.77) |  | 3076 (62.90) | 123 (91.11) |  | 3109 (63.29) | 90 (79.65) |  |
| Helsinki | 856 (17.52) | 17 (13.08) |  | 869 (17.77) | 5 (3.70) |  | 860 (17.51) | 14 (12.39) |  |

** N varies due to missing data for some of the variables from N=5025 to N=4858.

Data are presented as n (%).

P-value for differences between PEM, PEY and PEU with demographic, environmental, anthropometric, socioeconomic position, lifestyle (smoking, physical activity, alcohol consumption) and season of pesticide use covariates was analyzed by chi-square/fisher’s test for categorical variables.

^a^ Alcohol classification according to WHO sex-specific classification as abstainer, low risk drinker (≤20 g/day and ≤40 g/day for women and men, respectively) or at-risk drinker (> 20 g/day and>40 g/day for women and men, respectively).

^b^Includes students, pensioners, long-term unemployed, or not defined.

^c^High pesticide use season (summer (1 June–30 August) autumn (1 September–31 October)) and low pesticide use season (winter (1 November–31 March) spring (1 April–31 May)).

**Table S5. Spearman correlation analyses on the association between pesticide exposure in months, years, specific pesticides use and pesticide exposure overall with circulating metabolites.**

|  | **Variable** | **Pesticide exposure in months (PEM)** | | **Pesticide exposure in years (PEY)** | | **Specific pesticides use (PEU)** | | **Pesticide exposure overall (OPE)** | |
| --- | --- | --- | --- | --- | --- | --- | --- | --- | --- |
|  |  | ***rho*** | ***P-value*** | ***rho*** | ***P-value*** | ***rho*** | ***P-value*** | ***rho*** | ***P-value*** |
|  | **Lipoprotein subclasses** |  |  |  |  |  |  |  |  |
|  | Concentration of chylomicrons and extremely large VLDL particles [mol/L] | 0.028 | 0.053 | 0.047 | 0.001 | 0.033 | 0.023 | 0.056 | 0.0001 |
|  | Very-large VLDL [mol/L] | 0.024 | 0.093 | 0.040 | 0.005 | 0.028 | 0.049 | 0.047 | 0.001 |
|  | Large VLDL [mol/L] | 0.027 | 0.062 | 0.037 | 0.010 | 0.028 | 0.052 | 0.046 | 0.001 |
|  | Medium VLDL [mol/L] | 0.026 | 0.076 | 0.038 | 0.009 | 0.027 | 0.059 | 0.045 | 0.002 |
|  | Small VLDL [mol/L] | 0.026 | 0.077 | 0.039 | 0.008 | 0.020 | 0.158 | 0.044 | 0.002 |
|  | Very-small VLDL [mol/L] | 0.018 | 0.215 | 0.032 | 0.026 | 0.012 | 0.400 | 0.032 | 0.026 |
|  | IDL [mol/L] | 0.007 | 0.637 | -0.007 | 0.641 | 0.002 | 0.871 | 0.002 | 0.894 |
|  | Large LDL [mol/L] | 0.016 | 0.278 | 0.029 | 0.043 | 0.008 | 0.586 | 0.027 | 0.061 |
|  | Medium LDL [mol/L] | 0.016 | 0.256 | 0.030 | 0.035 | 0.011 | 0.462 | 0.029 | 0.048 |
|  | Small LDL [mol/L] | 0.019 | 0.194 | 0.029 | 0.045 | 0.011 | 0.461 | 0.029 | 0.041 |
|  | Very-large HDL [mol/L] | -0.002 | 0.890 | -0.037 | 0.011 | -0.012 | 0.392 | -0.030 | 0.037 |
|  | Large HDL [mol/L] | -0.022 | 0.126 | -0.054 | 0.0002 | -0.042 | 0.004 | -0.063 | <.0001 |
|  | Medium HDL [mol/L] | -0.024 | 0.095 | -0.034 | 0.020 | -0.048 | 0.001 | -0.051 | 0.0004 |
|  | Small HDL [mol/L] | -0.012 | 0.421 | 0.004 | 0.803 | -0.026 | 0.069 | -0.016 | 0.261 |
|  | **Triglycerides** |  |  |  |  |  |  |  |  |
|  | Triglycerides in chylomicrons and extremely large VLDL [mmol/L] | 0.028 | 0.054 | 0.048 | 0.001 | 0.033 | 0.021 | 0.057 | <.0001 |
|  | Very-large VLDL [mmol/L] | 0.023 | 0.112 | 0.037 | 0.010 | 0.027 | 0.065 | 0.044 | 0.002 |
|  | Large VLDL [mmol/L] | 0.026 | 0.074 | 0.035 | 0.016 | 0.026 | 0.073 | 0.043 | 0.003 |
|  | Medium VLDL [mmol/L] | 0.023 | 0.109 | 0.034 | 0.017 | 0.025 | 0.084 | 0.041 | 0.004 |
|  | Small VLDL [mmol/L] | 0.027 | 0.067 | 0.033 | 0.022 | 0.023 | 0.106 | 0.042 | 0.004 |
|  | Very-small VLDL [mmol/L] | 0.025 | 0.089 | 0.026 | 0.077 | 0.017 | 0.251 | 0.034 | 0.019 |
|  | Total VLDL [mmol/L] | 0.025 | 0.084 | 0.034 | 0.018 | 0.024 | 0.098 | 0.042 | 0.004 |
|  | IDL [mmol/L] | 0.018 | 0.217 | 0.016 | 0.264 | 0.011 | 0.457 | 0.021 | 0.143 |
|  | Large LDL [mmol/L] | 0.015 | 0.299 | 0.017 | 0.230 | 0.007 | 0.633 | 0.019 | 0.174 |
|  | Medium LDL [mmol/L] | 0.016 | 0.268 | 0.015 | 0.285 | 0.009 | 0.533 | 0.019 | 0.179 |
|  | Small LDL [mmol/L] | 0.021 | 0.143 | 0.022 | 0.125 | 0.011 | 0.456 | 0.028 | 0.053 |
|  | Total LDL [mmol/L] | 0.016 | 0.266 | 0.018 | 0.215 | 0.008 | 0.588 | 0.021 | 0.150 |
|  | Very-large HDL [mmol/L] | 0.017 | 0.254 | 0.010 | 0.479 | 0.008 | 0.575 | 0.014 | 0.333 |
|  | Large HDL [mmol/L] | -0.002 | 0.882 | -0.014 | 0.322 | -0.010 | 0.484 | -0.019 | 0.183 |
|  | Medium HDL [mmol/L] | 0.013 | 0.377 | 0.016 | 0.267 | 0.005 | 0.734 | 0.017 | 0.228 |
|  | Small HDL [mmol/L] | 0.021 | 0.140 | 0.044 | 0.002 | 0.028 | 0.056 | 0.046 | 0.001 |
|  | Total HDL [mmol/L] | 0.014 | 0.334 | 0.016 | 0.258 | 0.008 | 0.582 | 0.015 | 0.297 |
|  | Serum TG [mmol/L] | 0.026 | 0.068 | 0.032 | 0.025 | 0.021 | 0.145 | 0.039 | 0.006 |
|  | **Phospholipids** |  |  |  |  |  |  |  |  |
|  | Phospholipids in chylomicrons and extremely large VLDL [mmol/L] | 0.024 | 0.095 | 0.035 | 0.015 | 0.020 | 0.160 | 0.043 | 0.003 |
|  | Very-large VLDL [mmol/L] | 0.028 | 0.057 | 0.041 | 0.005 | 0.026 | 0.069 | 0.049 | 0.001 |
|  | Large VLDL [mmol/L] | 0.030 | 0.037 | 0.038 | 0.009 | 0.029 | 0.048 | 0.048 | 0.001 |
|  | Medium VLDL [mmol/L] | 0.026 | 0.067 | 0.039 | 0.007 | 0.026 | 0.067 | 0.046 | 0.001 |
|  | Small VLDL [mmol/L] | 0.024 | 0.099 | 0.034 | 0.019 | 0.017 | 0.251 | 0.039 | 0.006 |
|  | Very-small VLDL [mmol/L] | 0.012 | 0.392 | 0.024 | 0.095 | 0.007 | 0.614 | 0.021 | 0.142 |
|  | IDL [mmol/L] | 0.011 | 0.429 | 0.026 | 0.068 | 0.004 | 0.768 | 0.022 | 0.132 |
|  | Large LDL [mmol/L] | 0.014 | 0.330 | 0.027 | 0.062 | 0.004 | 0.765 | 0.024 | 0.101 |
|  | Medium LDL [mmol/L] | 0.020 | 0.165 | 0.030 | 0.037 | 0.008 | 0.591 | 0.030 | 0.036 |
|  | Small LDL [mmol/L] | 0.023 | 0.118 | 0.026 | 0.072 | 0.008 | 0.594 | 0.029 | 0.041 |
|  | Very-large HDL [mmol/L] | -0.008 | 0.581 | -0.048 | 0.001 | -0.019 | 0.187 | -0.044 | 0.002 |
|  | Large HDL [mmol/L] | -0.025 | 0.088 | -0.053 | 0.0002 | -0.045 | 0.002 | -0.065 | <.0001 |
|  | Medium HDL [mmol/L] | -0.025 | 0.089 | -0.035 | 0.016 | -0.049 | 0.001 | -0.052 | 0.0003 |
|  | Small HDL [mmol/L] | -0.025 | 0.086 | -0.023 | 0.118 | -0.052 | 0.0003 | -0.040 | 0.006 |
|  | **Cholesterol Esters** |  |  |  |  |  |  |  |  |
|  | Cholesterol esters in chylomicrons and extremely large VLDL [mmol/L] | 0.031 | 0.030 | 0.049 | 0.0006 | 0.042 | 0.004 | 0.061 | <.0001 |
|  | Very-large VLDL [mmol/L] | 0.024 | 0.102 | 0.049 | 0.001 | 0.039 | 0.007 | 0.057 | <.0001 |
|  | Large VLDL [mmol/L] | 0.026 | 0.071 | 0.047 | 0.001 | 0.038 | 0.008 | 0.055 | 0.0001 |
|  | Medium VLDL [mmol/L] | 0.030 | 0.038 | 0.044 | 0.003 | 0.033 | 0.021 | 0.052 | 0.0003 |
|  | Small VLDL [mmol/L] | 0.019 | 0.172 | 0.045 | 0.002 | 0.015 | 0.293 | 0.043 | 0.003 |
|  | Very-small VLDL [mmol/L] | 0.016 | 0.259 | 0.036 | 0.012 | 0.011 | 0.458 | 0.035 | 0.016 |
|  | IDL [mmol/L] | 0.017 | 0.250 | 0.033 | 0.022 | 0.008 | 0.602 | 0.030 | 0.035 |
|  | Large LDL [mmol/L] | 0.017 | 0.241 | 0.032 | 0.027 | 0.009 | 0.501 | 0.030 | 0.038 |
|  | Medium LDL [mmol/L] | 0.015 | 0.305 | 0.031 | 0.029 | 0.012 | 0.410 | 0.028 | 0.050 |
|  | Small LDL [mmol/L] | 0.016 | 0.275 | 0.029 | 0.046 | 0.011 | 0.445 | 0.027 | 0.060 |
|  | Very-large HDL [mmol/L] | 0.006 | 0.683 | -0.017 | 0.236 | -0.004 | 0.807 | -0.007 | 0.626 |
|  | Large HDL [mmol/L] | -0.022 | 0.136 | -0.056 | <.0001 | -0.039 | 0.006 | -0.063 | <.0001 |
|  | Medium HDL [mmol/L] | -0.031 | 0.034 | -0.039 | 0.006 | -0.054 | 0.0002 | -0.058 | <.0001 |
|  | Small HDL [mmol/L] | -0.005 | 0.726 | 0.017 | 0.251 | -0.006 | 0.658 | -0.002 | 0.906 |
|  | Esterified cholesterol [mmol/L] | 0.013 | 0.368 | 0.012 | 0.409 | -0.008 | 0.573 | 0.011 | 0.430 |
|  | **Free cholesterol** |  |  |  |  |  |  |  |  |
|  | Free cholesterol in chylomicrons and extremely large VLDL [mmol/L] | 0.023 | 0.107 | 0.039 | 0.006 | 0.023 | 0.111 | 0.045 | 0.002 |
|  | Very-large VLDL [mmol/L] | 0.026 | 0.071 | 0.045 | 0.002 | 0.027 | 0.063 | 0.051 | 0.0004 |
|  | Large VLDL [mmol/L] | 0.028 | 0.055 | 0.038 | 0.008 | 0.026 | 0.071 | 0.047 | 0.001 |
|  | Medium VLDL [mmol/L] | 0.025 | 0.086 | 0.035 | 0.014 | 0.024 | 0.101 | 0.042 | 0.004 |
|  | Small VLDL [mmol/L] | 0.022 | 0.123 | 0.036 | 0.014 | 0.014 | 0.325 | 0.038 | 0.008 |
|  | Very-small VLDL [mmol/L] | 0.009 | 0.538 | 0.029 | 0.038 | 0.009 | 0.507 | 0.023 | 0.104 |
|  | IDL [mmol/L] | 0.011 | 0.435 | 0.024 | 0.103 | 0.004 | 0.807 | 0.019 | 0.172 |
|  | Large LDL [mmol/L] | 0.013 | 0.351 | 0.027 | 0.059 | 0.006 | 0.671 | 0.024 | 0.097 |
|  | Medium LDL [mmol/L] | 0.019 | 0.181 | 0.032 | 0.025 | 0.011 | 0.449 | 0.032 | 0.028 |
|  | Small LDL [mmol/L] | 0.023 | 0.106 | 0.035 | 0.016 | 0.014 | 0.329 | 0.037 | 0.010 |
|  | Very-large HDL [mmol/L] | 0.005 | 0.735 | -0.032 | 0.025 | -0.006 | 0.691 | -0.019 | 0.189 |
|  | Large HDL [mmol/L] | -0.021 | 0.153 | -0.055 | 0.0001 | -0.036 | 0.012 | -0.062 | <.0001 |
|  | Medium HDL [mmol/L] | -0.027 | 0.059 | -0.032 | 0.026 | -0.046 | 0.001 | -0.053 | 0.0003 |
|  | Small HDL [mmol/L] | -0.020 | 0.163 | -0.007 | 0.639 | -0.040 | 0.006 | -0.026 | 0.070 |
|  | Free cholesterol [mmol/L] | 0.009 | 0.535 | 0.015 | 0.299 | -0.004 | 0.763 | 0.011 | 0.449 |
|  | **Total lipids** |  |  |  |  |  |  |  |  |
|  | Total lipids in chylomicrons and extremely large VLDL [mmol/L] | 0.028 | 0.054 | 0.047 | 0.001 | 0.032 | 0.025 | 0.055 | 0.0001 |
|  | Very-large VLDL [mmol/L] | 0.025 | 0.089 | 0.041 | 0.005 | 0.029 | 0.047 | 0.048 | 0.001 |
|  | Large VLDL [mmol/L] | 0.027 | 0.059 | 0.038 | 0.009 | 0.028 | 0.049 | 0.047 | 0.001 |
|  | Medium VLDL [mmol/L] | 0.026 | 0.073 | 0.038 | 0.008 | 0.028 | 0.057 | 0.046 | 0.002 |
|  | Small VLDL [mmol/L] | 0.025 | 0.085 | 0.039 | 0.007 | 0.019 | 0.178 | 0.044 | 0.003 |
|  | Very-small VLDL [mmol/L] | 0.017 | 0.250 | 0.032 | 0.025 | 0.012 | 0.418 | 0.031 | 0.031 |
|  | IDL [mmol/L] | 0.014 | 0.330 | 0.029 | 0.045 | 0.007 | 0.652 | 0.026 | 0.077 |
|  | Large LDL [mmol/L] | 0.015 | 0.285 | 0.029 | 0.042 | 0.008 | 0.581 | 0.027 | 0.062 |
|  | Medium LDL [mmol/L] | 0.016 | 0.256 | 0.031 | 0.034 | 0.011 | 0.451 | 0.029 | 0.046 |
|  | Small LDL [mmol/L] | 0.019 | 0.195 | 0.029 | 0.040 | 0.011 | 0.443 | 0.029 | 0.039 |
|  | Very-large HDL [mmol/L] | -0.001 | 0.922 | -0.036 | 0.013 | -0.012 | 0.419 | -0.029 | 0.048 |
|  | Large HDL [mmol/L] | -0.022 | 0.127 | -0.054 | 0.0002 | -0.041 | 0.004 | -0.063 | <.0001 |
|  | Medium HDL [mmol/L] | -0.025 | 0.084 | -0.034 | 0.018 | -0.048 | 0.001 | -0.052 | 0.0003 |
|  | Small HDL [mmol/L] | -0.013 | 0.368 | 0.003 | 0.832 | -0.027 | 0.061 | -0.018 | 0.219 |
|  | **Total cholesterol** |  |  |  |  |  |  |  |  |
|  | Total cholesterol in chylomicrons and extremely large VLDL [mmol/L] | 0.028 | 0.050 | 0.047 | 0.001 | 0.036 | 0.013 | 0.056 | <.0001 |
|  | Very-large VLDL [mmol/L] | 0.025 | 0.079 | 0.049 | 0.001 | 0.034 | 0.018 | 0.055 | 0.0001 |
|  | Large VLDL [mmol/L] | 0.027 | 0.063 | 0.043 | 0.003 | 0.033 | 0.022 | 0.052 | 0.0004 |
|  | Medium VLDL [mmol/L] | 0.027 | 0.059 | 0.041 | 0.005 | 0.029 | 0.041 | 0.048 | 0.0009 |
|  | Small VLDL [mmol/L] | 0.021 | 0.149 | 0.043 | 0.003 | 0.015 | 0.304 | 0.042 | 0.004 |
|  | Very-small VLDL [mmol/L] | 0.013 | 0.351 | 0.035 | 0.016 | 0.010 | 0.483 | 0.031 | 0.032 |
|  | IDL [mmol/L] | 0.015 | 0.294 | 0.030 | 0.035 | 0.006 | 0.662 | 0.027 | 0.058 |
|  | Large LDL [mmol/L] | 0.016 | 0.263 | 0.031 | 0.033 | 0.009 | 0.547 | 0.029 | 0.047 |
|  | Medium LDL [mmol/L] | 0.016 | 0.277 | 0.032 | 0.029 | 0.012 | 0.416 | 0.029 | 0.045 |
|  | Small LDL [mmol/L] | 0.017 | 0.236 | 0.030 | 0.037 | 0.012 | 0.422 | 0.029 | 0.043 |
|  | Total LDL [mmol/L] | 0.016 | 0.263 | 0.031 | 0.033 | 0.010 | 0.484 | 0.029 | 0.046 |
|  | Very-large HDL [mmol/L] | 0.005 | 0.716 | -0.022 | 0.129 | -0.004 | 0.759 | -0.011 | 0.445 |
|  | Large HDL [mmol/L] | -0.021 | 0.143 | -0.056 | 0.0001 | -0.038 | 0.008 | -0.062 | <.0001 |
|  | Medium HDL [mmol/L] | -0.029 | 0.041 | -0.037 | 0.010 | -0.052 | 0.0003 | -0.057 | <.0001 |
|  | Small HDL [mmol/L] | -0.009 | 0.554 | 0.013 | 0.357 | -0.013 | 0.377 | -0.007 | 0.615 |
|  | Total HDL [mmol/L] | -0.019 | 0.184 | -0.045 | 0.002 | -0.046 | 0.002 | -0.056 | 0.0001 |
|  | Serum cholesterol [mmol/L] | 0.013 | 0.370 | 0.015 | 0.304 | -0.005 | 0.718 | 0.014 | 0.349 |
|  | Remnant cholesterol [mmol/L] | 0.023 | 0.119 | 0.039 | 0.007 | 0.015 | 0.309 | 0.040 | 0.005 |
|  | **Apolipoproteins** |  |  |  |  |  |  |  |  |
|  | Apolipoprotein A1 [g/L] | -0.014 | 0.331 | -0.035 | 0.015 | -0.043 | 0.003 | -0.046 | 0.002 |
|  | Apolipoprotein B [g/L] | 0.025 | 0.078 | 0.038 | 0.009 | 0.015 | 0.290 | 0.041 | 0.004 |
|  | Ratio of Apo B/Apo A1 | 0.034 | 0.019 | 0.062 | <.0001 | 0.042 | 0.003 | 0.069 | <.0001 |
|  | **Amino acids** |  |  |  |  |  |  |  |  |
|  | Alanine [µmol/L] | 0.002 | 0.864 | -0.009 | 0.532 | -0.011 | 0.447 | -0.011 | 0.455 |
|  | Glutamine [µmol/L] | -0.007 | 0.619 | 0.021 | 0.147 | -0.008 | 0.583 | 0.003 | 0.844 |
|  | Glycine [µmol/L] | -0.017 | 0.264 | -0.016 | 0.286 | -0.018 | 0.227 | -0.027 | 0.066 |
|  | Histidine [µmol/L] | 0.006 | 0.656 | -0.006 | 0.672 | -0.004 | 0.779 | -0.003 | 0.826 |
|  | **Branched-chain amino acids** |  |  |  |  |  |  |  |  |
|  | Isoleucine [µmol/L] | 0.022 | 0.131 | 0.012 | 0.389 | 0.003 | 0.821 | 0.017 | 0.233 |
|  | Leucine [µmol/L] | 0.026 | 0.072 | 0.026 | 0.078 | 0.003 | 0.845 | 0.027 | 0.059 |
|  | Valine [µmol/L] | 0.018 | 0.214 | 0.013 | 0.370 | -0.008 | 0.594 | 0.017 | 0.249 |
|  | **Aromatic amino acids** |  |  |  |  |  |  |  |  |
|  | Phenylalanine [µmol/L] | 0.028 | 0.054 | 0.025 | 0.079 | 0.026 | 0.076 | 0.027 | 0.058 |
|  | Tyrosine [µmol/L] | 0.001 | 0.959 | 0.018 | 0.225 | 0.002 | 0.916 | 0.004 | 0.792 |
|  | **Ketone bodies** |  |  |  |  |  |  |  |  |
|  | Acetoacetate [µmol/L] | 0.005 | 0.735 | -0.017 | 0.237 | -0.032 | 0.027 | -0.014 | 0.323 |
|  | Acetate [µmol/L] | 0.011 | 0.444 | 0.015 | 0.292 | -0.009 | 0.513 | 0.012 | 0.388 |
|  | beta-hydroxybutyrate [µmol/L] | 0.012 | 0.403 | -0.020 | 0.167 | -0.022 | 0.127 | -0.011 | 0.463 |
|  | **Sphingolipids** |  |  |  |  |  |  |  |  |
|  | Sphingomyelin | 0.001 | 0.948 | -0.018 | 0.206 | -0.031 | 0.0304 | -0.020 | 0.159 |
|  | Total choline | 0.002 | 0.895 | -0.010 | 0.473 | -0.027 | 0.0588 | -0.014 | 0.325 |
|  | **Glycolysis related metabolites** |  |  |  |  |  |  |  |  |
|  | Citrate [µmol/L] | 0.014 | 0.339 | 0.004 | 0.790 | 0.006 | 0.660 | 0.007 | 0.609 |
|  | Glucose [mmol/L] | -0.001 | 0.939 | 0.022 | 0.133 | -0.024 | 0.097 | 0.009 | 0.553 |
|  | Glycerol [mmol/L] | 0.0004 | 0.977 | 0.011 | 0.484 | 0.025 | 0.097 | 0.014 | 0.349 |
|  | Lactate [mmol/L] | -0.015 | 0.294 | -0.015 | 0.285 | -0.015 | 0.284 | -0.022 | 0.131 |
|  | Pyruvate [mmol/L] | 0.018 | 0.207 | 0.006 | 0.667 | 0.002 | 0.899 | 0.019 | 0.198 |
|  | **Fatty acids** |  |  |  |  |  |  |  |  |
|  | Total fatty acids [mmol/L] | 0.009 | 0.511 | 0.003 | 0.862 | -0.013 | 0.379 | 0.002 | 0.866 |
|  | Monounsaturated fatty acids [mmol/L] | 0.017 | 0.242 | 0.007 | 0.615 | -0.005 | 0.736 | 0.013 | 0.385 |
|  | Saturated fatty acids [mmol/L] | 0.012 | 0.422 | 0.016 | 0.265 | -0.001 | 0.965 | 0.014 | 0.344 |
|  | **Inflammation** |  |  |  |  |  |  |  |  |
|  | Alpha-1-acid glycoprotein | -0.008 | 0.603 | -0.006 | 0.667 | -0.021 | 0.155 | -0.016 | 0.268 |
|  | **Fluid balance** |  |  |  |  |  |  |  |  |
|  | Creatinine [µmol/L] | -0.011 | 0.436 | 0.030 | 0.037 | -0.011 | 0.467 | 0.008 | 0.587 |
|  | Albumin [standardized concentration units] | 0.009 | 0.524 | 0.002 | 0.891 | -0.023 | 0.108 | -0.002 | 0.895 |

**Pesticide exposure overall**

**Table S6. Multivariable regression analyses of pesticide exposure overall with total lipoprotein subclasses with standardised metabolomic biomarkers in men**

| **Pesticide exposure overall, β (95% CI), Males** | | | | | | |
| --- | --- | --- | --- | --- | --- | --- |
| **Total lipoproteins** | **Unadjusted** | **+ BMI** | **+ socioeconomic**  **position** | **+ season of pesticide use** | **+ alcohol consumption** | **+ latitude of residence** |
| XXL_VLDL_P | 0.088 (-0.068, 0.244) | 0.106 (-0.045, 0.258) | 0.095 (-.067, 0.258) | 0.102 (-.061, 0.265) | 0.103 (-.062, 0.267) | 0.102 (-.063, 0.267) |
| XL_VLDL_P | 0.010 (-0.145, 0.165) | 0.026 (-0.126, 0.177) | 0.014 (-.148, 0.176) | 0.013 (-.149, 0.175) | 0.019 (-.145, 0.184) | 0.021 (-.144, 0.185) |
| L_VLDL_P | -0.022 (-0.173, 0.129) | -0.007 (-0.154, 0.139) | 0.017 (-.139, 0.174) | 0.013 (-.145, 0.169) | 0.023 (-.136, 0.182) | 0.026 (-.133, 0.185) |
| M_VLDL_P | 0.0099 (-0.137, 0.157) | 0.026 (-0.116, 0.168) | 0.046 (-.107, 0.198) | 0.042 (-.112, 0.195) | 0.045 (-.110, 0.199) | 0.045 (-.110, 0.199) |
| S_VLDL_P | -0.000015 (-0.138, 0.138) | 0.018 (-0.115, 0.151) | 0.025 (-.118, 0.168) | 0.018 (-.126, 0.162) | 0.018 (-.128, 0.164) | 0.0089 (-.137, 0.155) |
| XS_VLDL_P | 0.0056 (-0.141, 0.152) | 0.025 (-0.119, 0.168) | -.020 (-.174, 0.134) | -.033 (-.188, 0.121) | -.036 (-.192, 0.121) | -.054 (-.209, 0.102) |
| IDL_P | -0.0068 (-0.151, 0.137) | -0.0048 (-0.149, 0.139) | 0.015 (-.142, 0.172) | 0.021 (-.136, 0.178) | 0.011 (-.149, 0.170) | 0.018 (-.142, 0.177) |
| L_LDL_P | 0.049 (-0.102, 0.202) | 0.062 (-0.089, 0.214) | -.017 (-.179, 0.145) | -.034 (-.196, 0.128) | -.029 (-.193, 0.135) | -.048 (-.210, 0.115) |
| M_LDL_P | 0.037 (-0.113, 0.187) | 0.047 (-0.102, 0.197) | -.025 (-.185, 0.135 | -.041 (-.201, 0.119) | -.035 (-.196, 0.127) | -.052 (-.213, 0.108) |
| S_LDL_P | 0.030 (-0.121, 0.181) | 0.039 (-0.110, 0.189) | -.032 (-.192, 0.129) | -.047 (-.208, 0.113) | -.038 (-.200, 0.125) | -.055 (-.216, 0.106) |
| XL_HDL_P | 0.047 (-0.102, 0.197) | 0.036 (-0.109, 0.182) | -.008 (-.165, 0.149) | -.00021 (-.158, 0.157) | 0.0066 (-.153, 0.166) | -.00063 (-.159, 0.159) |
| L_HDL_P | -0.0037 (-0.155, 0.147) | -0.019 (-0.165, 0.128) | -.052 (-.210, 0.105) | -.064 (-.222, 0.094) | -.045 (-.204, 0.114) | -.047 (-.206, 0.112) |
| M_HDL_P | -0.093 (-0.241, 0.055) | -0.106 (-0.254, 0.043) | -.102 (-.261, 0.057) | -.121 (-.279, 0.038) | -.088 (-.246, 0.069) | -.082 (-.237, 0.074) |
| S_HDL_P | -0.069 (-0.219, 0.081) | -0.077 (-0.228, 0.074) | -.112 (-.274, 0.049) | -.135 (-.296, 0.026) | -.105 (-.266, 0.055) | -.113 (-.272, 0.046) |

**Table S7. Multivariable regression analyses of pesticide exposure overall with total lipoprotein subclasses with standardised metabolomic biomarkers in women**

| **Pesticide exposure overall, β (95% CI), Females** | | | | | | |
| --- | --- | --- | --- | --- | --- | --- |
| **Total lipoprotein** | **Unadjusted** | **+ BMI** | **+ socioeconomic position** | **+ season of pesticide use** | **+ alcohol consumption** | **+ latitude of residence** |
| XXL_VLDL_P | 0.166 (-0.044, 0.377) | 0.141 (-0.061, 0.343) | 0.131 (-.075, 0.337) | 0.125 (-.082, 0.332) | 0.136 (-.073, 0.344) | 0.139 (-.069, 0.347) |
| XL_VLDL_P | 0.017 (-0.207, 0.241) | -0.0012 (-0.219, 0.216) | -.022 (-.243, 0.198) | -.038 (-.260, 0.184) | -.030 (-.253, 0.193) | -.019 (-.242, 0.204) |
| L_VLDL_P | 0.0083 (-0.212, 0.228) | -0.0074 (-0.219, 0.205) | -.014 (-.229, 0.202) | -.0278 (-.245, 0.189) | 0.018 (-.203, 0.238) | 0.029 (-.191, 0.249) |
| M_VLDL_P | 0.103 (-0.101, 0.307) | 0.080 (-0.116, 0.276) | 0.057 (-.142, 0.256) | 0.046 (-.154, 0.246) | 0.082 (-.121, 0.284) | 0.095 (-.107, 0.297) |
| S_VLDL_P | 0.135 (-0.082, 0.352) | 0.117 (-0.092, 0.325) | 0.077 (-.135, 0.288) | 0.053 (-.159, 0.265) | 0.083 (-.131, 0.297) | 0.083 (-.132, 0.297) |
| XS_VLDL_P | 0.172 (-0.052, 0.397) | 0.160 (-0.059, 0.380) | 0.097 (-.126, 0.319) | 0.074 (-.149, 0.297) | 0.099 (-.126, 0.325) | 0.096 (-.129, 0.321) |
| IDL_P | 0.025 (-0.235, 0.285) | 0.046 (-0.215, 0.308) | 0.058 (-.208, 0.324) | 0.058 (-.211, 0.326) | 0.045 (-.225, 0.316) | 0.045 (-.226, 0.315) |
| L_LDL_P | 0.130 (-0.095, 0.356) | 0.121 (-0.103, 0.345) | 0.046 (-.179, 0.273) | 0.022 (-.204, 0.248) | 0.054 (-.174, 0.282) | 0.053 (-.174, 0.281) |
| M_LDL_P | 0.137 (-0.088, 0.363) | 0.127 (-0.097, 0.351) | 0.057 (-.169, 0.283) | 0.033 (-.193, 0.258) | 0.074 (-.154, 0.302) | 0.073 (-.154, 0.300) |
| S_LDL_P | 0.141 (-0.086, 0.367) | 0.131 (-0.094, 0.356) | 0.065 (-.163, 0.292) | 0.039 (-.187, 0.266) | 0.082 (-.148, 0.311) | 0.079 (-.149, 0.309) |
| XL_HDL_P | 0.018 (-0.179, 0.216) | 0.051 (-0.144, 0.245) | 0.035 (-.163, 0.232) | 0.011 (-.188, 0.209) | -.017 (-.218, 0.184) | -.037 (-.236, 0.162) |
| L_HDL_P | -0.220 (-.418, -0.023) | -0.187 (-0.379, 0.0053) | -.211 (-.407, -.016) | -.236 (-.432, -.039) | -.274 (-.473, -.076) | -.274 (-.473, -.075) |
| M_HDL_P | -0.288 (-0.516, -0.059) | -0.280 (-0.509, -0.051) | -.308 (-.540, -.075) | -.322 (-.556, -.088) | -.349 (-.586, -.113) | -.331 (-.566, -.096) |
| S_HDL_P | -0.246 (-0.484, -0.0082) | -0.249 (-0.488, -0.0096) | -.304 (-.546, -.062) | -.323 (-.565, -.080) | -.331 (-.577, -.085) | -.322 (-.566, -.078) |

**Table S8. Multivariable regression analyses of pesticide exposure overall with triglycerides subclasses with standardised metabolomic biomarkers in men**

| **Pesticide exposure overall, β (95% CI), Males** | | | | | | |
| --- | --- | --- | --- | --- | --- | --- |
| **Triglycerides** | **Unadjusted** | **+ BMI** | **+ socioeconomic position** | **+ season of pesticide use** | **+ alcohol consumption** | **+ latitude of residence** |
| XXL_VLDL_TG | 0.084 (-0.070, 0.238) | 0.101 (-0.048, 0.250) | 0.089 (-.071, 0.248) | 0.096 (-.065, 0.256) | 0.098 (-.065, 0.259) | 0.097 (-.065, 0.259) |
| XL_VLDL_TG | -0.0069 (-0.157, 0.143) | 0.0059 (-0.141, 0.153) | -.0011 (-.158, 0.156) | -.0035 (-.161, 0.154) | 0.0052 (-.154, 0.165) | 0.0088 (-.151, 0.168) |
| L_VLDL_TG | -0.048 (-0.196, 0.100) | -0.035 (-0.179, 0.109) | -.0057 (-.160, 0.149) | -.011 (-.166, 0.143) | 0.0026 (-.154, 0.159) | 0.0068 (-.149, 0.163) |
| M_VLDL_TG | -0.010 (-0.152, 0.131) | 0.0025 (-0.134, 0.139) | 0.029 (-.118, 0.176) | 0.025 (-.122, 0.172) | 0.031 (-.118, 0.179) | 0.033 (-.116, 0.182) |
| S_VLDL_TG | -0.0087 (-0.143, 0.126) | 0.0064 (-0.123, 0.136) | 0.029 (-.110, 0.168) | 0.023 (-.117, 0.163) | 0.026 (-.116, 0.167) | 0.021 (-.121, 0.163) |
| XS_VLDL_TG | -0.0092 (-0.147, 0.128) | 0.0062 (-0.127, 0.139) | 0.0016 (-.141, 0.144) | -.0101 (-.153, 0.133) | -.0074 (-.152, 0.138) | -.022 (-.167, 0.122) |
| VLDL_TG | -0.000079 (-0.145, 0.145) | 0.014 (-0.126, 0.154) | 0.033 (-.117, 0.184) | 0.029 (-.122, 0.179) | 0.034 (-.118, 0.187) | 0.034 (-.119, 0.187) |
| IDL_TG | 0.014 (-0.128, 0.156) | 0.026 (-0.113, 0.164) | -.012 (-.161, 0.137) | -.028 (-.177, 0.120) | -.025 (-.176, 0.125) | -.048 (-.197, 0.101) |
| L_LDL_TG | 0.056 (-0.088, 0.201) | 0.066 (-0.076, 0.208) | 0.0087 (-.143, 0.161) | -.0079 (-.159, 0.144) | -.0046 (-.158, 0.149) | -.031 (-.182, 0.120) |
| M_LDL_TG | 0.076 (-0.069, 0.221) | 0.082 (-0.060, 0.225) | 0.027 (-.126, 0.181) | 0.012 (-.141, 0.165) | 0.016 (-.138, 0.171) | -.0093 (-.161, 0.143) |
| S_LDL_TG | 0.057 (-0.087, 0.200) | 0.066 (-0.075, 0.206) | 0.028 (-.122, 0.179) | 0.015 (-.136, 0.165) | 0.021 (-.131, 0.174) | 0.0015 (-.149, 0.153) |
| LDL_TG | 0.062 (-0.083, 0.206) | 0.070 (-0.072, 0.212) | 0.017 (-.136, 0.169) | 0.00043 (-.152, 0.152) | 0.0057 (-.148, 0.159) | -.0192 (-.171, 0.132) |
| XL_HDL_TG | 0.197 (0.046, 0.347) | 0.208 (0.057, 0.359) | 0.150 (-.012, 0.312) | 0.145 (-.017, 0.308) | 0.139 (-.025, 0.304) | 0.121 (-.043, 0.284) |
| L_HDL_TG | 0.136 (-0.0182, 0.289) | 0.135 (-0.019, 0.289) | 0.097 (-.069, 0.265) | 0.082 (-.085, 0.248) | 0.080 (-.088, 0.249) | 0.066 (-.101, 0.232) |
| M_HDL_TG | 0.057 (-0.092, 0.206) | 0.072 (-0.074, 0.218) | 0.077 (-.079, 0.234) | 0.073 (-.084, 0.231) | 0.075 (-.084, 0.234) | 0.068 (-.091, 0.227) |
| S_HDL_TG | 0.074 (-0.060, 0.209) | 0.088 (-0.042, 0.217) | 0.053 (-.086, 0.192) | 0.052 (-.088, 0.191) | 0.049 (-.092, 0.189) | 0.031 (-.109, 0.171) |
| HDL_TG | 0.108 (-0.039, 0.255) | 0.120 (-0.025, 0.266) | 0.086 (-.070, 0.243) | 0.076 (-.080, 0.233) | 0.076 (-.083, 0.234) | 0.058 (-.099, 0.216) |
| Serum_TG | 0.014 (-0.138, 0.165) | 0.029 (-0.117, 0.174) | 0.033 (-.123, 0.189) | 0.025 (-.132, 0.182) | 0.032 (-.127, 0.190) | 0.025 (-.133, 0.184) |

**Table S9. Multivariable regression analyses of pesticide exposure overall with triglycerides subclasses with standardised metabolomic biomarkers in women**

| **Pesticide exposure overall, β (95% CI), Females** | | | | | | |
| --- | --- | --- | --- | --- | --- | --- |
| **Triglycerides** | **Unadjusted** | **+ BMI** | **+ socioeconomic position** | **+ season of pesticide use** | **+ alcohol consumption** | **+ latitude of residence** |
| XXL_VLDL_TG | 0.169 (-0.040, 0.378) | 0.143 (-0.059, 0.344) | 0.135 (-.070, 0.339) | 0.131 (-.076, 0.337) | 0.143 (-.065, 0.350) | 0.146 (-.062, 0.353) |
| XL_VLDL_TG | -0.007 (-0.236, 0.222) | -0.026 (-0.249, 0.197) | -.044 (-.271, 0.182) | -.062 (-.289, 0.166) | -.056 (-.285, 0.173) | -.043 (-.271, 0.185) |
| L_VLDL_TG | -0.005 (-0.228, 0.217) | -0.022 (-0.237, 0.193) | -.025 (-.244, 0.193) | -.039 (-.259, 0.181) | 0.0063 (-.218, 0.230) | 0.021 (-.203, 0.244) |
| M_VLDL_TG | 0.086 (-0.127, 0.298) | 0.063 (-0.143, 0.268) | 0.049 (-.159, 0.258) | 0.041 (-.168, 0.250) | 0.082 (-.129, 0.295) | 0.098 (-.114, 0.309) |
| S_VLDL_TG | 0.163 (-0.067, 0.393) | 0.146 (-0.077, 0.368) | 0.119 (-.108, 0.346) | 0.099 (-.128, 0.327) | 0.124 (-.106, 0.354) | 0.126 (-.104, 0.356) |
| XS_VLDL_TG | 0.173 (-0.062, 0.407) | 0.164 (-0.064, 0.392) | 0.114 (-.117, 0.346) | 0.085 (-.147, 0.316) | 0.092 (-.143, 0.326) | 0.088 (-.146, 0.322) |
| VLDL_TG | 0.122 (-0.092, 0.335) | 0.099 (-0.105, 0.305) | 0.079 (-.129, 0.287) | 0.064 (-.145, 0.273) | 0.090 (-.122, 0.302) | 0.101 (-.110, 0.313) |
| IDL_TG | 0.212 (-0.024, 0.448) | 0.211 (-0.021, 0.444) | 0.148 (-.087, 0.383) | 0.113 (-.122, 0.349) | 0.104 (-.134, 0.343) | 0.095 (-.143, 0.333) |
| L_LDL_TG | 0.198 (-0.041, 0.438) | 0.200 (-0.037, 0.437) | 0.130 (-.109, 0.369) | 0.093 (-.146, 0.333) | 0.078 (-.164, 0.321) | 0.066 (-.176, 0.309) |
| M_LDL_TG | 0.213 (-0.022, 0.448) | 0.213 (-0.020, 0.446) | 0.156 (-.080, 0.392) | 0.122 (-.114, 0.359) | 0.109 (-.131, 0.348) | 0.096 (-.143, 0.335) |
| S_LDL_TG | 0.199 (-0.036, 0.434) | 0.192 (-0.039, 0.424) | 0.140 (-.095, 0.375) | 0.109 (-.126, 0.344) | 0.102 (-.136, 0.340) | 0.093 (-.145, 0.330) |
| LDL_TG | 0.204 (-0.034, 0.441) | 0.203 (-0.031, 0.438) | 0.137 (-.100, 0.375) | 0.102 (-.135, 0.339) | 0.088 (-.152, 0.329) | 0.077 (-.163, 0.317) |
| XL_HDL_TG | -0.002 (-0.232, 0.228) | 0.022 (-0.209, 0.253) | -.041 (-.274, 0.193) | -.073 (-.308, 0.162) | -.107 (-.345, 0.130) | -.112 (-.349, 0.126) |
| L_HDL_TG | -0.159 (-0.383, 0.066) | -0.133 (-0.357, 0.092) | -.199 (-.428, 0.028) | -.233 (-.461, -.0048) | -.274 (-.506, -.043) | -.278 (-.509, -.047) |
| M_HDL_TG | 0.077 (-0.149, 0.303) | 0.064 (-0.159, 0.289) | 0.017 (-.210, 0.244) | -.0081 (-.236, 0.220) | -.0277 (-.259, 0.205) | -.021 (-.253, 0.211) |
| S_HDL_TG | 0.229 (-0.012, 0.472) | 0.222 (-0.016, 0.459) | 0.169 (-.072, 0.410) | 0.137 (-.105, 0.379) | 0.124 (-.121, 0.370) | 0.111 (-.135, 0.356) |
| HDL_TG | 0.018 (-0.219, 0.256) | 0.026 (-0.211, 0.264) | -.045 (-.285, 0.195) | -.085 (-.325, 0.156) | -.120 (-.365, 0.124) | -.124 (-.368, 0.121) |
| Serum_TG | 0.142 (-0.069, 0.354) | 0.128 (-0.076, 0.332) | 0.089 (-.118, 0.296) | 0.065 (-.143, 0.272) | 0.070 (-.140, 0.280) | 0.075 (-.135, 0.286) |

**Table S10. Multivariable regression analyses of pesticide exposure overall with phospholipid subclasses with standardised metabolomic biomarkers in men**

| **Pesticide exposure overall, β (95% CI), Males** | | | | | | |
| --- | --- | --- | --- | --- | --- | --- |
| **Phospholipids** | **Unadjusted** | **+ BMI** | **+ socioeconomic position** | **+ season of pesticide use** | **+ alcohol consumption** | **+ latitude of residence** |
| XXL_VLDL_PL | 0.049 (-0.099, 0.199) | 0.064 (-0.081, 0.210) | 0.059 (-.096, 0.216) | 0.062 (-.095, 0.219) | 0.068 (-.091, 0.226) | 0.069 (-.090, 0.227) |
| XL_VLDL_PL | 0.049 (-0.105, 0.202) | 0.064 (-0.086, 0.214) | 0.049 (-.110, 0.210) | 0.049 (-.112, 0.211) | 0.055 (-.108, 0.218) | 0.054 (-.109, 0.217) |
| L_VLDL_PL | 0.0089 (-0.141, 0.159) | 0.025 (-0.121, 0.170) | 0.046 (-.109, 0.202) | 0.041 (-.115, 0.197) | 0.051 (-.1060, 0.209) | 0.053 (-.105, 0.211) |
| M_VLDL_PL | 0.019 (-0.130, 0.169) | 0.037 (-0.108, 0.181) | 0.049 (-.105, 0.205) | 0.044 (-.111, 0.200) | 0.047 (-.111, 0.205) | 0.045 (-.113, 0.203) |
| S_VLDL_PL | -0.014 (-0.154, 0.126) | 0.0042 (-0.131, 0.139) | 0.013 (-.133, 0.158) | 0.0051 (-.141, 0.152) | 0.0068 (-.141, 0.155) | -.0033 (-.151, 0.145) |
| XS_VLDL_PL | -0.0072 (-0.158, 0.144) | 0.0092 (-0.141, 0.159) | -.044 (-.205, 0.117) | -.061 (-.222, 0.100) | -.060 (-.223, 0.103) | -.078 (-.239, 0.084) |
| IDL_PL | 0.049 (-0.109, 0.206) | 0.062 (-0.095, 0.219) | -.023 (-.192, 0.145) | -.040 (-.208, 0.128) | -.039 (-.209, 0.131) | -.057 (-.226, 0.112) |
| L_LDL_PL | 0.047 (-0.106, 0.201) | 0.059 (-0.094, 0.213) | -.023 (-.187, 0.142) | -.040 (-.205, 0.124) | -.034 (-.200, 0.132) | -.051 (-.216, 0.114) |
| M_LDL_PL | 0.052 (-0.103, 0.208) | 0.064 (-0.089, 0.218) | -.016 (-.181, 0.149) | -.033 (-.198, 0.132) | -.022 (-.189, 0.144) | -.040 (-.206, 0.125) |
| S_LDL_PL | 0.051 (-0.102, 0.203) | 0.059 (-0.093, 0.210) | -.021 (-.183, 0.141) | -.037 (-.199, 0.126) | -.020 (-.184, 0.143) | -.038 (-.200, 0.125) |
| XL_HDL_PL | 0.019 (-0.143, 0.182) | 0.0048 (-0.151, 0.161) | -.006 (-.174, 0.162) | -.0025 (-.171, 0.166) | 0.000084 (-.169, 0.169) | -.003 (-.173, 0.167) |
| L_HDL_PL | -0.035 (-0.188, 0.118) | -0.051 (-0.199, 0.098) | -.079 (-.238, 0.082) | -.091 (-.252, 0.069) | -.069 (-.231, 0.092) | -.069 (-.229, 0.091) |
| M_HDL_PL | -0.089 (-0.237, 0.059) | -0.102 (-0.251, 0.046) | -.107 (-.266, 0.052) | -.126 (-.285, 0.033) | -.090 (-.248, 0.067) | -.085 (-.241, 0.070) |
| S_HDL_PL | -0.088 (-0.231, 0.056) | -0.098 (-0.243, 0.046) | -.106 (-.260, 0.048) | -.123 (-.278, 0.031) | -.093 (-.246, 0.060) | -.089 (-.241, 0.061) |

**Table S11. Multivariable regression analyses of pesticide exposure overall with phospholipid subclasses with standardised metabolomic biomarkers in women**

| **Pesticide exposure overall, β (95% CI), Females** | | | | | | |
| --- | --- | --- | --- | --- | --- | --- |
| **Phospholipids** | **Unadjusted** | **+ BMI** | **+ socioeconomic position** | **+ season of pesticide use** | **+ alcohol consumption** | **+ latitude of residence** |
| XXL_VLDL_PL | 0.087 (-0.129, 0.303) | 0.065 (-0.146, 0.276) | 0.063 (-.152, 0.278) | 0.053 (-.163, 0.269) | 0.055 (-.163, 0.273) | 0.065 (-.153, 0.283) |
| XL_VLDL_PL | 0.026 (-0.204, 0.257) | 0.0066 (-0.218, 0.232) | -.022 (-.249, 0.205) | -.038 (-.267, 0.192) | -.034 (-.265, 0.198) | -.024 (-.255, 0.208) |
| L_VLDL_PL | -0.025 (-0.245, 0.196) | -0.040 (-0.253, 0.173) | -.054 (-.271, 0.162) | -.071 (-.289, 0.147) | -.034 (-.256, 0.188) | -.024 (-.246, 0.198) |
| M_VLDL_PL | 0.112 (-0.095, 0.319) | 0.090 (-0.109, 0.289) | 0.059 (-.143, 0.262) | 0.045 (-.158, 0.248) | 0.076 (-.129, 0.282) | 0.088 (-.118, 0.293) |
| S_VLDL_PL | 0.149 (-0.078, 0.378) | 0.134 (-0.087, 0.354) | 0.093 (-.131, 0.317) | 0.066 (-.159, 0.290) | 0.087 (-.141, 0.314) | 0.084 (-.143, 0.312) |
| XS_VLDL_PL | 0.164 (-0.063, 0.390) | 0.153 (-0.071, 0.377) | 0.087 (-.139, 0.314) | 0.066 (-.160, 0.293) | 0.092 (-.137, 0.321) | 0.093 (-.136, 0.322) |
| IDL_PL | 0.119 (-0.107, 0.347) | 0.112 (-0.114, 0.339) | 0.035 (-.193, 0.264) | 0.011 (-.217, 0.239) | 0.035 (-.195, 0.265) | 0.036 (-.194, 0.266) |
| L_LDL_PL | 0.087 (-0.136, 0.309) | 0.078 (-0.144, 0.299) | 0.0025 (-.221, 0.226) | -.019 (-.243, 0.204) | 0.012 (-.214, 0.237) | 0.013 (-.212, 0.239) |
| M_LDL_PL | 0.106 (-0.120, 0.332) | 0.097 (-0.127, 0.321) | 0.017 (-.209, 0.243) | -.0089 (-.234, 0.216) | 0.024 (-.204, 0.252) | 0.023 (-.205, 0.251) |
| S_LDL_PL | 0.111 (-0.114, 0.335) | 0.106 (-0.117, 0.329) | 0.031 (-.194, 0.256) | 0.0019 (-.223, 0.227) | 0.031 (-.198, 0.259) | 0.029 (-.199, 0.258) |
| XL_HDL_PL | -0.0041 (-0.167, 0.159) | 0.025 (-0.134, 0.183) | 0.0154 (-.146, 0.177) | -.0016 (-.164, 0.161) | -.027 (-.190, 0.137) | -.037 (-.199, 0.126) |
| L_HDL_PL | -0.238 (-0.437, -0.039) | -0.207 (-0.401, -0.0125) | -.229 (-.426, -.032) | -.252 (-.451, -.054) | -.289 (-.490, -.089) | -.287 (-.488, -.086) |
| M_HDL_PL | -0.284 (-0.515, -0.052) | -0.273 (-0.506, -0.041) | -.304 (-.539, -.068) | -.325 (-.562, -.087) | -.356 (-.596, -.116) | -.339 (-.578, -.100) |
| S_HDL_PL | -0.416 (-0.647, -0.184) | -0.416 (-0.649, -0.183) | -.433 (-.669, -.196) | -.439 (-.678, -.202) | -.468 (-.708, -.227) | -.452 (-.691, -.213) |

**Table S12. Multivariable regression analyses of pesticide exposure overall with cholesterol esters subclasses with standardised metabolomic biomarkers in men**

| **Pesticide exposure overall, β (95% CI), Males** | | | | | | |
| --- | --- | --- | --- | --- | --- | --- |
| **Cholesterol esters** | **Unadjusted** | **+ BMI** | **+ socioeconomic position** | **+ season of pesticide use** | **+ alcohol consumption** | **+ latitude of residence** |
| XXL_VLDL_CE | 0.139 (-0.0042, 0.282) | 0.160 (0.020, 0.299) | 0.174 (0.025, 0.324) | 0.182 (0.031, 0.332) | 0.171 (0.019, 0.323) | 0.166 (0.014, 0.319) |
| XL_VLDL_CE | 0.049 (-0.098, 0.197) | 0.072 (-0.072, 0.216) | 0.058 (-.096, 0.212) | 0.066 (-.088, 0.220) | 0.057 (-.099, 0.213) | 0.053 (-.104, 0.209) |
| L_VLDL_CE | 0.017 (-0.132, 0.166) | 0.039 (-0.107, 0.185) | 0.079 (-.077, 0.236) | 0.082 (-.074, 0.238) | 0.075 (-.083, 0.232) | 0.073 (-.084, 0.231) |
| M_VLDL_CE | 0.069 (-0.083, 0.221) | 0.093 (-0.054, 0.240) | 0.092 (-.067, 0.249) | 0.089 (-.069, 0.249) | 0.081 (-.079, 0.242) | 0.073 (-.088, 0.234) |
| S_VLDL_CE | 0.038 (-0.104, 0.179) | 0.056 (-0.083, 0.195) | 0.024 (-.125, 0.173) | 0.017 (-.133, 0.167) | 0.0079 (-.144, 0.159) | -.0069 (-.158, 0.144) |
| XS_VLDL_CE | 0.041 (-0.109, 0.192) | 0.059 (-0.089, 0.209) | -.007 (-.167, 0.152) | -.012 (-.173, 0.148) | -.023 (-.184, 0.139) | -.038 (-.199, 0.122) |
| IDL_CE | 0.058 (-0.093, 0.208) | 0.072 (-0.077, 0.222) | -.012 (-.173, 0.148) | -.025 (-.186, 0.136) | -.023 (-.186, 0.139) | -.039 (-.201, 0.122) |
| L_LDL_CE | 0.046 (-0.106, 0.197) | 0.058 (-0.092, 0.209) | -.019 (-.181, 0.142) | -.034 (-.196, 0.128) | -.031 (-.194, 0.133) | -.047 (-.209, 0.116) |
| M_LDL_CE | 0.020 (-0.129, 0.170) | 0.029 (-0.120, 0.179) | -.038 (-.198, 0.123) | -.0523 (-.213, 0.109) | -.049 (-.212, 0.114) | -.063 (-.225, 0.099) |
| S_LDL_CE | 0.0075 (-0.147, 0.162) | 0.015 (-0.139, 0.169) | -.054 (-.220, 0.113) | -.067 (-.234, 0.099) | -.063 (-.232, 0.105) | -.077 (-.245, 0.091) |
| XL_HDL_CE | 0.055 (-0.099, 0.209) | 0.046 (-0.107, 0.199) | -.0114 (-.176, 0.153) | 0.0052 (-.160, 0.171) | 0.012 (-.156, 0.179) | 0.00023 (-.167, 0.167) |
| L_HDL_CE | 0.017 (-0.139, 0.173) | 0.0017 (-0.149, 0.152) | -.031 (-.194, 0.131) | -.040 (-.203, 0.123) | -.023 (-.187, 0.141) | -.024 (-.188, 0.139) |
| M_HDL_CE | -0.101 (-0.252, 0.049) | -0.117 (-0.266, 0.033) | -.100 (-.261, 0.060) | -.118 (-.279, 0.043) | -.086 (-.246, 0.074) | -.075 (-.234, 0.083) |
| S_HDL_CE | -0.069 (-0.236, 0.098) | -0.075 (-0.243, 0.092) | -.130 (-.310, 0.050) | -.149 (-.329, 0.030) | -.133 (-.316, 0.049) | -.147 (-.329, 0.034) |
| EstC | 0.018 (-0.135, 0.171) | 0.025 (-0.129, 0.178) | -.045 (-.210, 0.119) | -.061 (-.225, 0.104) | -.048 (-.215, 0.118) | -.064 (-.229, 0.101) |

**Table S13. Multivariable regression analyses of pesticide exposure overall with cholesterol esters subclasses with standardised metabolomic biomarkers in women**

| **Pesticide exposure overall, β (95% CI), Females** | | | | | | |
| --- | --- | --- | --- | --- | --- | --- |
| **Cholesterol esters** | **Unadjusted** | **Adjusted for BMI** | **+ socioeconomic position** | **+ season of pesticide use** | **+ alcohol consumption** | **+ latitude of residence** |
| XXL_VLDL_CE | 0.255 (0.022, 0.488) | 0.233 (0.0046, 0.461) | 0.212 (-.019, 0.444) | 0.205 (-.0291, 0.438) | 0.216 (-.019, 0.451) | 0.215 (-.0191, 0.449) |
| XL_VLDL_CE | 0.064 (-0.177, 0.304) | 0.047 (-0.189, 0.283) | 0.024 (-.214, 0.263) | 0.012 (-.227, 0.252) | 0.023 (-.217, 0.263) | 0.023 (-.216, 0.262) |
| L_VLDL_CE | 0.079 (-0.152, 0.312) | 0.065 (-0.161, 0.292) | 0.050 (-.180, 0.281) | 0.040 (-.192, 0.272) | 0.084 (-.151, 0.319) | 0.079 (-.156, 0.315) |
| M_VLDL_CE | 0.192 (-0.020, 0.404) | 0.171 (-0.035, 0.377) | 0.127 (-.082, 0.336) | 0.113 (-.097, 0.323) | 0.139 (-.073, 0.352) | 0.147 (-.066, 0.359) |
| S_VLDL_CE | 0.113 (-0.109, 0.334) | 0.093 (-0.123, 0.309) | 0.036 (-.183, 0.255) | 0.015 (-.204, 0.234) | 0.058 (-.162, 0.279) | 0.054 (-.167, 0.275) |
| XS_VLDL_CE | 0.164 (-0.053, 0.381) | 0.149 (-0.065, 0.365) | 0.096 (-.122, 0.314) | 0.088 (-.131, 0.307) | 0.120 (-.099, 0.340) | 0.114 (-.106, 0.334) |
| IDL_CE | 0.123 (-0.098, 0.345) | 0.112 (-0.108, 0.333) | 0.043 (-.179, 0.266) | 0.025 (-.197, 0.248) | 0.054 (-.171, 0.279) | 0.055 (-.169, 0.279) |
| L_LDL_CE | 0.126 (-0.102, 0.354) | 0.114 (-0.113, 0.341) | 0.044 (-.185, 0.274) | 0.024 (-.205, 0.253) | 0.071 (-.160, 0.302) | 0.072 (-.159, 0.302) |
| M_LDL_CE | 0.126 (-0.101, 0.353) | 0.114 (-0.112, 0.341) | 0.056 (-.173, 0.285) | 0.038 (-.190, 0.267) | 0.102 (-.128, 0.332) | 0.104 (-.125, 0.334) |
| S_LDL_CE | 0.127 (-0.094, 0.349) | 0.117 (-0.104, 0.337) | 0.065 (-.159, 0.288) | 0.047 (-.176, 0.270) | 0.109 (-.115, 0.335) | 0.111 (-.114, 0.336) |
| XL_HDL_CE | 0.085 (-0.131, 0.300) | 0.108 (-0.107, 0.324) | 0.094 (-.125, 0.313) | 0.073 (-.148, 0.293) | 0.055 (-.169, 0.279) | 0.023 (-.197, 0.242) |
| L_HDL_CE | -0.189 (-0.373, -0.007) | -0.156 (-0.333, 0.020) | -.172 (-.352, 0.0069) | -.194 (-.374, -.013) | -.228 (-.411, -.046) | -.230 (-.413, -.048) |
| M_HDL_CE | -0.301 (-0.523, -0.079) | -0.296 (-0.518, -0.073) | -.306 (-.532, -.080) | -.306 (-.534, -.079) | -.326 (-.556, -.096) | -.306 (-.534, -.078) |
| S_HDL_CE | -0.049 (-0.255, 0.156) | -0.055 (-0.262, 0.151) | -.113 (-.321, 0.095) | -.129 (-.336, 0.078) | -.098 (-.309, 0.112) | -.097 (-.307, 0.112) |
| EstC | 0.046 (-0.178, 0.270) | 0.045 (-0.179, 0.269) | -.026 (-.253, 0.201) | -.054 (-.281, 0.173) | -.036 (-.266, 0.194) | -.036 (-.267, 0.194) |

**Table S14. Multivariable regression analyses of pesticide exposure overall with free cholesterol subclasses with standardised metabolomic biomarkers in men**

| **Pesticide exposure overall, β (95% CI), Males** | | | | |  |  |
| --- | --- | --- | --- | --- | --- | --- |
| **Free cholesterol** | **Unadjusted** | **+ BMI** | **+ SEP** | **+ season of pesticide use** | **+ alcohol consumption** | **+ latitude of residence** |
| XXL_VLDL_FC | 0.086 (-0.064, 0.236) | 0.101 (-0.045, 0.246) | 0.088 (-.068, 0.245) | 0.091 (-.066, 0.248) | 0.092 (-.067, 0.251) | 0.090 (-.069, 0.249) |
| XL_VLDL_FC | 0.067 (-0.085, 0.219) | 0.085 (-0.064, 0.233) | 0.064 (-.095, 0.222) | 0.064 (-.095, 0.224) | 0.065 (-.097, 0.227) | 0.06 (-.102, 0.222) |
| L_VLDL_FC | -0.0094 (-0.155, 0.136) | 0.0054 (-0.136, 0.147) | 0.027 (-.125, 0.179) | 0.022 (-.130, 0.175) | 0.029 (-.126, 0.183) | 0.029 (-.125, 0.184) |
| M_VLDL_FC | 0.018 (-0.128, 0.164) | 0.034 (-0.107, 0.175) | 0.049 (-.102, 0.202) | 0.045 (-.108, 0.197) | 0.047 (-.108, 0.202) | 0.045 (-.109, 0.199) |
| S_VLDL_FC | -0.0038 (-0.141, 0.133) | 0.013 (-0.119, 0.147) | 0.012 (-.132, 0.155) | 0.002 (-.142, 0.146) | 0.0036 (-.142, 0.149) | -.0079 (-.153, 0.137) |
| XS_VLDL_FC | -0.018 (-0.169, 0.133) | -0.0016 (-0.152, 0.148) | -.051 (-.212, 0.111) | -.066 (-.227, 0.095) | -.069 (-.232, 0.095) | -.087 (-.249, 0.076) |
| IDL_FC | 0.033 (-0.126, 0.193) | 0.045 (-0.115, 0.205) | -.037 (-.209, 0.134) | -.053 (-.225, 0.118) | -.054 (-.227, 0.119) | -.071 (-.243, 0.101) |
| L_LDL_FC | 0.053 (-0.103, 0.209) | 0.064 (-0.093, 0.221) | -.027 (-.195, 0.141) | -.043 (-.211, 0.125) | -.041 (-.210, 0.129) | -.059 (-.227, 0.109) |
| M_LDL_FC | 0.055 (-0.095, 0.205) | 0.064 (-0.086, 0.215) | -.024 (-.185, 0.137) | -.040 (-.201, 0.121) | -.032 (-.195, 0.129) | -.052 (-.213, 0.109) |
| S_LDL_FC | 0.058 (-0.095, 0.211) | 0.068 (-0.086, 0.221) | -.022 (-.186, 0.142) | -.037 (-.201, 0.127) | -.027 (-.192, 0.139) | -.047 (-.212, 0.117) |
| XL_HDL_FC | 0.071 (-0.092, 0.235) | 0.062 (-0.097, 0.221) | 0.0099 (-.161, 0.181) | 0.018 (-.154, 0.189) | 0.024 (-.150, 0.198) | 0.023 (-.151, 0.197) |
| L_HDL_FC | -0.014 (-0.178, 0.149) | -0.029 (-0.186, 0.128) | -.059 (-.227, 0.110) | -.067 (-.236, 0.103) | -.049 (-.221, 0.122) | -.052 (-.223, 0.119) |
| M_HDL_FC | -0.109 (-0.264, 0.046) | -0.123 (-0.277, 0.032) | -.124 (-.289, 0.041) | -.144 (-.309, 0.021) | -.112 (-.278, 0.053) | -.107 (-.271, 0.056) |
| S_HDL_FC | -0.073 (-0.227, 0.080) | -0.083 (-0.237, 0.072) | -.117 (-.283, 0.048) | -.139 (-.3043, 0.026) | -.111 (-.276, 0.053) | -.118 (-.281, 0.045) |
| Free cholesterol | 0.021 (-0.134, 0.176) | 0.029 (-0.127, 0.185) | -.054 (-.221, 0.113) | -.073 (-.240, 0.094) | -.065 (-.234, 0.104) | -.081 (-.249, 0.086) |

**Table S15. Multivariable regression analyses of pesticide exposure overall with free cholesterol subclasses with standardised metabolomic biomarkers in women**

| **Pesticide exposure overall, β (95% CI), Females** | | | | |  |  |
| --- | --- | --- | --- | --- | --- | --- |
| **Free cholesterol** | **Unadjusted** | **+ BMI** | **+ SEP** | **+ season of pesticide use** | **+ alcohol consumption** | **+ latitude of residence** |
| XXL_VLDL_FC | 0.112 (-0.108, 0.332) | 0.093 (-0.122, 0.307) | 0.075 (-.143, 0.292) | 0.062 (-.157, 0.281) | 0.059 (-.162, 0.281) | 0.068 (-.153, 0.289) |
| XL_VLDL_FC | 0.028 (-0.201, 0.256) | 0.0099 (-0.214, 0.233) | -.023 (-.246, 0.201) | -.036 (-.262, 0.189) | -.031 (-.258, 0.197) | -.0254 (-.253, 0.202) |
| L_VLDL_FC | -0.035 (-0.266, 0.197) | -0.048 (-0.273, 0.177) | -.063 (-.291, 0.166) | -.082 (-.312, 0.148) | -.054 (-.288, 0.180) | -.044 (-.277, 0.190) |
| M_VLDL_FC | 0.105 (-0.109, 0.319) | 0.085 (-0.121, 0.291) | 0.053 (-.156, 0.262) | 0.035 (-.175, 0.245) | 0.057 (-.156, 0.269) | 0.069 (-.143, 0.282) |
| S_VLDL_FC | 0.111 (-0.115, 0.337) | 0.096 (-0.122, 0.315) | 0.048 (-.174, 0.269) | 0.0193 (-.202, 0.242) | 0.043 (-.181, 0.268) | 0.042 (-.183, 0.266) |
| XS_VLDL_FC | 0.135 (-0.087, 0.357) | 0.126 (-0.093, 0.346) | 0.066 (-.156, 0.289) | 0.046 (-.177, 0.269) | 0.074 (-.152, 0.299) | 0.072 (-.154, 0.297) |
| IDL_FC | 0.126 (-0.098, 0.349) | 0.120 (-0.103, 0.344) | 0.049 (-.176, 0.276) | 0.027 (-.198, 0.253) | 0.053 (-.175, 0.280) | 0.054 (-.174, 0.281) |
| L_LDL_FC | 0.122 (-0.099, 0.344) | 0.116 (-0.105, 0.337) | 0.042 (-.181, 0.266) | 0.019 (-.204, 0.242) | 0.052 (-.174, 0.277) | 0.050 (-.175, 0.275) |
| M_LDL_FC | 0.125 (-0.095, 0.345) | 0.119 (-0.100, 0.338) | 0.041 (-.179, 0.262) | 0.017 (-.204, 0.237) | 0.053 (-.169, 0.276) | 0.048 (-.174, 0.271) |
| S_LDL_FC | 0.142 (-0.083, 0.367) | 0.136 (-0.087, 0.359) | 0.059 (-.166, 0.285) | 0.035 (-.191, 0.260) | 0.074 (-.155, 0.302) | 0.066 (-.162, 0.294) |
| XL_HDL_FC | 0.092 (-0.103, 0.288) | 0.119 (-0.074, 0.313) | 0.107 (-.090, 0.303) | 0.087 (-.111, 0.285) | 0.067 (-.133, 0.268) | 0.053 (-.146, 0.251) |
| L_HDL_FC | -0.150 (-0.326, 0.026) | -0.117 (-0.287, 0.052) | -.129 (-.301, 0.043) | -.148 (-.321, 0.026) | -.177 (-.352, -.002) | -.179 (-.354, -.004) |
| M_HDL_FC | -0.260 (-0.479, -0.041) | -0.249 (-0.469, -0.030) | -.279 (-.502, -.056) | -.297 (-.521, -.072) | -.322 (-.548, -.095) | -.305 (-.531, -.079) |
| S_HDL_FC | -0.399 (-0.641, -0.158) | -0.393 (-0.637, -0.149) | -.438 (-.685, -.191) | -.465 (-.713, -.218) | -.491 (-.742, -.240) | -.484 (-.733, -.234) |
| Free cholesterol | 0.059 (-0.172, 0.291) | 0.059 (-0.172, 0.290) | -.018 (-.251, 0.216) | -.045 (-.279, 0.188) | -.033 (-.270, 0.203) | -.031 (-.268, 0.206) |

**Table S16. Multivariable regression analyses of pesticide exposure overall with total lipid subclasses with standardised metabolomic biomarkers in men**

| **Pesticide exposure overall, β (95% CI), Males** | | | | | | |
| --- | --- | --- | --- | --- | --- | --- |
| **Total lipids** | **Unadjusted** | **+ BMI** | **+ SEP** | **+ season of pesticide use** | **+ alcohol consumption** | **+ latitude of residence** |
| XXL_VLDL_L | 0.088 (-0.067, 0.244) | 0.107 (-0.044, 0.257) | 0.096 (-.065, 0.257) | 0.102 (-.059, 0.264) | 0.103 (-.061, 0.267) | 0.102 (-.062, 0.266) |
| XL_VLDL_L | 0.017 (-0.138, 0.172) | 0.033 (-0.118, 0.184) | 0.019 (-.142, 0.182) | 0.019 (-.143, 0.182) | 0.025 (-.139, 0.189) | 0.025 (-.139, 0.190) |
| L_VLDL_L | -0.019 (-0.169, 0.131) | -0.0034 (-0.149, 0.142) | 0.020 (-.135, 0.176) | 0.016 (-.140, 0.172) | 0.026 (-.132, 0.183) | 0.0286 (-.129, 0.186) |
| M_VLDL_L | 0.014 (-0.134, 0.162) | 0.030 (-0.112, 0.173) | 0.049 (-.104, 0.202) | 0.045 (-.109, 0.198) | 0.047 (-.108, 0.203) | 0.047 (-.109, 0.203) |
| S_VLDL_L | 0.0017 (-0.136, 0.139) | 0.019 (-0.114, 0.153) | 0.023 (-.119, 0.167) | 0.016 (-.128, 0.160) | 0.015 (-.130, 0.161) | 0.0057 (-.140, 0.151) |
| XS_VLDL_L | 0.0062 (-0.139, 0.151) | 0.025 (-0.118, 0.167) | -.023 (-.176, 0.130) | -.036 (-.189, 0.118) | -.039 (-.194, 0.117) | -.056 (-.210, 0.098) |
| IDL_L | 0.051 (-0.099, 0.203) | 0.065 (-0.085, 0.216) | -.016 (-.177, 0.146) | -.031 (-.192, 0.131) | -.029 (-.192, 0.134) | -.047 (-.209, 0.115) |
| L_LDL_L | 0.049 (-0.103, 0.202) | 0.062 (-0.089, 0.214) | -.019 (-.181, 0.144) | -.035 (-.197, 0.128) | -.031 (-.195, 0.134) | -.049 (-.212, 0.114) |
| M_LDL_L | 0.037 (-0.115, 0.189) | 0.048 (-0.104, 0.199) | -.027 (-.189, 0.136) | -.043 (-.206, 0.119) | -.037 (-.201, 0.127) | -.055 (-.218, 0.108) |
| S_LDL_L | 0.030 (-0.119, 0.179) | 0.039 (-0.109, 0.188) | -.033 (-.192, 0.127) | -.048 (-.208, 0.111) | -.039 (-.199, 0.122) | -.055 (-.215, 0.105) |
| XL_HDL_L | 0.048 (-0.104, 0.199) | 0.037 (-0.112, 0.185) | -.0089 (-.169, 0.151) | -.00056 (-.161, 0.159) | 0.0064 (-.156, 0.169) | -.00049 (-.163, 0.162) |
| L_HDL_L | -0.0051 (-0.157, 0.147) | -0.020 (-0.168, 0.127) | -.053 (-.212, 0.105) | -.065 (-.224, 0.094) | -.046 (-.206, 0.115) | -.047 (-.207, 0.113) |
| M_HDL_L | -0.095 (-0.243, 0.052) | -0.109 (-0.256, 0.039) | -.105 (-.263, 0.053) | -.124 (-.282, 0.035) | -.091 (-.248, 0.067) | -.084 (-.239, 0.071) |
| S_HDL_L | -0.073 (-0.226, 0.080) | -0.081 (-0.235, 0.073) | -.118 (-.283, 0.046) | -.141 (-.305, 0.023) | -.111 (-.274, 0.053) | -.119 (-.280, 0.043) |

**Table S17. Multivariable regression analyses of pesticide exposure overall with total lipid subclasses with standardised metabolomic biomarkers in women**

| **Pesticide exposure overall, β (95% CI), Females** | | | | | | |
| --- | --- | --- | --- | --- | --- | --- |
| **Total lipids** | **Unadjusted** | **+ BMI** | **+ SEP** | **+ season of pesticide use** | **+ alcohol consumption** | **+ latitude of residence** |
| XXL_VLDL_L | 0.163 (-0.047, 0.372) | 0.138 (-0.064, 0.339) | 0.127 (-.078, 0.332) | 0.120 (-.086, 0.326) | 0.130 (-.078, 0.338) | 0.133 (-.074, 0.341) |
| XL_VLDL_L | 0.019 (-0.204, 0.242) | 0.00075 (-0.216, 0.217) | -.0213 (-.241, 0.198) | -.037 (-.258, 0.184) | -.029 (-.251, 0.194) | -.018 (-.240, 0.204) |
| L_VLDL_L | 0.010 (-0.208, 0.228) | -0.005 (-0.215, 0.205) | -.013 (-.226, 0.201) | -.027 (-.242, 0.188) | 0.018 (-.201, 0.236) | 0.028 (-.190, 0.246) |
| M_VLDL_L | 0.108 (-0.097, 0.313) | 0.085 (-0.111, 0.282) | 0.060 (-.139, 0.259) | 0.049 (-.152, 0.249) | 0.082 (-.121, 0.285) | 0.095 (-.108, 0.298) |
| S_VLDL_L | 0.131 (-0.086, 0.349) | 0.113 (-0.096, 0.322) | 0.071 (-.141, 0.283) | 0.047 (-.166, 0.259) | 0.077 (-.138, 0.292) | 0.077 (-.138, 0.292) |
| XS_VLDL_L | 0.167 (-0.054, 0.388) | 0.155 (-0.062, 0.371) | 0.092 (-.127, 0.311) | 0.071 (-.149, 0.291) | 0.098 (-.124, 0.319) | 0.094 (-.127, 0.316) |
| IDL_L | 0.134 (-0.087, 0.355) | 0.126 (-0.094, 0.345) | 0.053 (-.169, 0.275) | 0.029 (-.193, 0.251) | 0.052 (-.172, 0.276) | 0.052 (-.172, 0.276) |
| L_LDL_L | 0.127 (-0.098, 0.352) | 0.118 (-0.106, 0.342) | 0.044 (-.182, 0.269) | 0.020 (-.206, 0.246) | 0.054 (-.173, 0.282) | 0.054 (-.174, 0.282) |
| M_LDL_L | 0.136 (-0.093, 0.365) | 0.125 (-0.102, 0.353) | 0.055 (-.175, 0.284) | 0.031 (-.198, 0.259) | 0.075 (-.157, 0.306) | 0.074 (-.157, 0.305) |
| S_LDL_L | 0.139 (-0.085, 0.363) | 0.129 (-0.093, 0.352) | 0.064 (-.161, 0.288) | 0.039 (-.185, 0.264) | 0.083 (-.144, 0.309) | 0.081 (-.146, 0.308) |
| XL_HDL_L | 0.027 (-0.173, 0.227) | 0.059 (-0.137, 0.256) | 0.044 (-.156, 0.243) | 0.020 (-.180, 0.221) | -.0077 (-.211, 0.195) | -.027 (-.228, 0.174) |
| L_HDL_L | -0.216 (-0.413, -0.0197) | -0.182 (-0.373, 0.0083) | -.206 (-.399, -.0119) | -.229 (-.425, -.034) | -.267 (-.465, -.070) | -.267 (-.465, -.070) |
| M_HDL_L | -0.287 (-0.512, -0.062) | -0.279 (-0.505, -0.054) | -.306 (-.535, -.076) | -.319 (-.550, -.089) | -.346 (-.579, -.113) | -.327 (-.559, -.096) |
| S_HDL_L | -0.257 (-0.497, -0.016) | -0.259 (-0.500, -0.017) | -.314 (-.559, -.069) | -.334 (-.578, -.089) | -.341 (-.589, -.093) | -.332 (-.579, -.086) |

**Table S18. Multivariable regression analyses of pesticide exposure overall with total cholesterol subclasses with standardised metabolomic biomarkers in men**

| **Pesticide exposure overall, β (95% CI), Males** | | | | | | |
| --- | --- | --- | --- | --- | --- | --- |
| **Total cholesterol** | **Unadjusted** | **+ BMI** | **+ SEP** | **+ season of pesticide use** | **+ alcohol consumption** | **+ latitude of residence** |
| XXL_VLDL_C | 0.118 (-0.031, 0.267) | 0.138 (-0.0069, 0.283) | 0.136 (-.019, 0.291) | 0.142 (-.014, 0.298) | 0.135 (-.023, 0.293) | 0.131 (-.027, 0.289) |
| XL_VLDL_C | 0.059 (-0.093, 0.211) | 0.079 (-0.069, 0.227) | 0.061 (-.097, 0.219) | 0.065 (-.094, 0.224) | 0.061 (-.100, 0.222) | 0.056 (-.105, 0.218) |
| L_VLDL_C | 0.0032 (-0.146, 0.152) | 0.023 (-0.122, 0.169) | 0.059 (-.096, 0.215) | 0.058 (-.098, 0.214) | 0.058 (-.100, 0.216) | 0.056 (-.102, 0.214) |
| M_VLDL_C | 0.048 (-0.102, 0.197) | 0.069 (-0.076, 0.213) | 0.075 (-.080, 0.231) | 0.072 (-.084, 0.228) | 0.068 (-.089, 0.226) | 0.063 (-.095, 0.221) |
| S_VLDL_C | 0.023 (-0.116, 0.162) | 0.041 (-0.094, 0.177) | 0.020 (-.125, 0.166) | 0.012 (-.134, 0.158) | 0.0068 (-.141, 0.155) | -.007 (-.155, 0.141) |
| XS_VLDL_C | 0.022 (-0.131, 0.175) | 0.041 (-0.111, 0.192) | -.023 (-.185, 0.139) | -.032 (-.195, 0.131) | -.041 (-.205, 0.124) | -.057 (-.221, 0.106) |
| IDL_C | 0.051 (-0.103, 0.205) | 0.065 (-0.088, 0.218) | -.020 (-.184, 0.144) | -.034 (-.199, 0.130) | -.033 (-.199, 0.133) | -.049 (-.214, 0.115) |
| L_LDL_C | 0.048 (-0.105, 0.201) | 0.060 (-0.092, 0.213) | -.021 (-.185, 0.143) | -.036 (-.200, 0.127) | -.033 (-.199, 0.132) | -.049 (-.214, 0.115) |
| M_LDL_C | 0.026 (-0.123, 0.175) | 0.036 (-0.113, 0.185) | -.036 (-.196, 0.124) | -.051 (-.212, 0.109) | -.047 (-.209, 0.115) | -.063 (-.223, 0.098) |
| S_LDL_C | 0.017 (-0.133, 0.166) | 0.025 (-0.124, 0.174) | -.047 (-.208, 0.113) | -.062 (-.222, 0.099) | -.055 (-.218, 0.107) | -.070 (-.232, 0.091) |
| LDL_C | 0.034 (-0.115, 0.184) | 0.045 (-0.104, 0.194) | -.031 (-.191, 0.129) | -.046 (-.206, 0.114) | -.042 (-.203, 0.119) | -.058 (-.218, 0.103) |
| XL_HDL_C | 0.058 (-0.097, 0.213) | 0.049 (-0.104, 0.202) | -.0091 (-.174, 0.155) | 0.0049 (-.160, 0.170) | 0.012 (-.155, 0.179) | 0.003 (-.164, 0.169) |
| L_HDL_C | 0.013 (-0.144, 0.170) | -0.0024 (-0.154, 0.149) | -.035 (-.198, 0.129) | -.043 (-.207, 0.121) | -.026 (-.191, 0.139) | -.027 (-.193, 0.138) |
| M_HDL_C | -0.105 (-0.256, 0.046) | -0.120 (-0.270, 0.029) | -.107 (-.268, 0.053) | -.125 (-.286, 0.036) | -.093 (-.253, 0.068) | -.083 (-.242, 0.075) |
| S_HDL_C | -0.062 (-0.221, 0.097) | -0.069 (-0.229, 0.091) | -.121 (-.293, 0.051) | -.143 (-.314, 0.028) | -.121 (-.294, 0.051) | -.134 (-.306, 0.037) |
| HDL_C_ | -0.031 (-0.176, 0.113) | -0.049 (-0.191, 0.091) | -.082 (-.233, 0.069) | -.096 (-.247, 0.056) | -.070 (-.2221, 0.082) | -.073 (-.225, 0.078) |
| Serum C | 0.032 (-0.125, 0.189) | 0.039 (-0.118, 0.197) | -.034 (-.203, 0.135) | -.052 (-.220, 0.117) | -.039 (-.210, 0.130) | -.056 (-.226, 0.113) |
| Remnant C | 0.048 (-0.101, 0.196) | 0.067 (-0.078, 0.212) | 0.017 (-.138, 0.173) | 0.0069 (-.149, 0.163) | 0.0053 (-.153, 0.163) | -.0091 (-.166, 0.148) |

**Table S19. Multivariable regression analyses of pesticide exposure overall with total cholesterol subclasses with standardised metabolomic biomarkers in women**

| **Pesticide exposure overall, β (95% CI), Females** | | | | | | |
| --- | --- | --- | --- | --- | --- | --- |
| **Total cholesterol** | **Unadjusted** | **+ BMI** | **+ SEP** | **+ season of pesticide use** | **+ alcohol consumption** | **+ latitude of residence** |
| XXL_VLDL_C | 0.196 (-0.026, 0.419) | 0.175 (-0.041, 0.391) | 0.154 (-.065, 0.374) | 0.144 (-.077, 0.365) | 0.151 (-.072, 0.373) | 0.153 (-.069, 0.375) |
| XL_VLDL_C | 0.047 (-0.180, 0.275) | 0.030 (-0.191, 0.252) | 0.0029 (-.219, 0.226) | -.010 (-.234, 0.214) | -.00069 (-.226, 0.225) | 0.0021 (-.223, 0.228) |
| L_VLDL_C | 0.011 (-0.214, 0.235) | -0.0035 (-0.221, 0.214) | -.019 (-.241, 0.201) | -.035 (-.257, 0.188) | 0.0086 (-.217, 0.234) | 0.011 (-.215, 0.237) |
| M_VLDL_C | 0.156 (-0.052, 0.365) | 0.136 (-0.066, 0.337) | 0.096 (-.108, 0.301) | 0.080 (-.125, 0.286) | 0.106 (-.102, 0.315) | 0.115 (-.093, 0.323) |
| S_VLDL_C | 0.111 (-0.108, 0.331) | 0.093 (-0.119, 0.306) | 0.039 (-.177, 0.254) | 0.015 (-.201, 0.231) | 0.052 (-.166, 0.269) | 0.048 (-.169, 0.266) |
| XS_VLDL_C | 0.159 (-0.063, 0.382) | 0.147 (-0.073, 0.366) | 0.088 (-.135, 0.311) | 0.075 (-.149, 0.299) | 0.107 (-.118, 0.332) | 0.102 (-.123, 0.328) |
| IDL_C | 0.126 (-0.099, 0.352) | 0.117 (-0.108, 0.341) | 0.046 (-.181, 0.273) | 0.026 (-.201, 0.253) | 0.055 (-.174, 0.284) | 0.056 (-.173, 0.285) |
| L_LDL_C | 0.125 (-0.102, 0.353) | 0.114 (-0.112, 0.341) | 0.043 (-.185, 0.272) | 0.022 (-.206, 0.251) | 0.065 (-.165, 0.296) | 0.066 (-.165, 0.296) |
| M_LDL_C | 0.129 (-0.099, 0.357) | 0.118 (-0.109, 0.344) | 0.054 (-.175, 0.283) | 0.034 (-.194, 0.263) | 0.089 (-.142, 0.319) | 0.089 (-.141, 0.319) |
| S_LDL_C | 0.135 (-0.089, 0.359) | 0.124 (-0.099, 0.347) | 0.065 (-.160, 0.291) | 0.046 (-.179, 0.271) | 0.101 (-.126, 0.328) | 0.100 (-.127, 0.327) |
| LDL_C | 0.127 (-0.097, 0.351) | 0.116 (-0.107, 0.339) | 0.050 (-.175, 0.276) | 0.0307 (-.195, 0.256) | 0.078 (-.149, 0.305) | 0.078 (-.149, 0.305) |
| XL_HDL_C | 0.085 (-0.123, 0.293) | 0.111 (-0.097, 0.318) | 0.096 (-.115, 0.307) | 0.074 (-.138, 0.286) | 0.055 (-.160, 0.269) | 0.027 (-.184, 0.238) |
| L_HDL_C | -0.184 (-0.367, 0.00031) | -0.149 (-0.327, 0.028) | -.165 (-.345, 0.015) | -.186 (-.368, -.0047) | -.220 (-.404, -.037) | -.222 (-.406, -.039) |
| M_HDL_C | -0.295 (-0.514, -.075) | -0.288 (-0.508, -0.068) | -.303 (-.526, -.079) | -.308 (-.532, -.083) | -.329 (-.555, -.101) | -.309 (-.535, -.084) |
| S_HDL_C | -0.116 (-0.336, 0.103) | -0.119 (-0.340, 0.100) | -.183 (-.405, 0.039) | -.203 (-.424, 0.019) | -.183 (-.408, 0.042) | -.181 (-.405, 0.043) |
| HDL_C_ | -0.213 (-0.421, -.0045) | -0.184 (-0.389, 0.020) | -.216 (-.424, -.0085) | -.244 (-.453, -.035) | -.274 (-.486, -.062) | -.273 (-.485, -.061) |
| Serum C | 0.051 (-0.180, 0.283) | 0.050 (-0.181, 0.282) | -.025 (-.259, 0.209) | -.054 (-.288, 0.180) | -.036 (-.274, 0.201) | -.036 (-.273, 0.202) |
| Remnant C | 0.147 (-0.069, 0.364) | 0.131 (-0.081, 0.343) | 0.068 (-.146, 0.283) | 0.049 (-.166, 0.264) | 0.082 (-.135, 0.299) | 0.083 (-.134, 0.300) |

**Table S20. Multivariable regression analyses of pesticide exposure overall with apolipoproteins, aminoacids, sphingomyelins, glucose metabolism, fatty acids, inflammation, and fluid balance with standardised metabolomic biomarkers in men**

| **Pesticide exposure overall, β (95% CI), Males** | | | | | | |
| --- | --- | --- | --- | --- | --- | --- |
|  | **Unadjusted** | **+ BMI** | **+ SEP** | **+ season of pesticide use** | **+ alcohol consumption** | **+ latitude of residence** |
| **Apolipoproteins** |  |  |  |  |  |  |
| Apolipoprotein A1 [g/L] | -0.035 (-0.177, 0.107) | -0.046 (-0.188, 0.095) | -.076 (-.228, 0.076) | -.095 (-.247, 0.057) | -.069 (-.221, 0.083) | -.074 (-.225, 0.077) |
| Apolipoprotein B [g/L] | 0.033 (-0.116, 0.182) | 0.051 (-0.095, 0.197) | 0.0023 (-.154, 0.159) | -.011 (-.168, 0.146) | -.0069 (-.166, 0.152) | -.020 (-.178, 0.138) |
| Ratio of Apo B/Apo A1 | 0.054 (-0.087, 0.195) | 0.079 (-0.054, 0.213) | 0.052 (-.092, 0.195) | 0.052 (-.092, 0.197) | 0.038 (-.107, 0.184) | 0.029 (-.117, 0.174) |
| **Amino acids** |  |  |  |  |  |  |
| Alanine [µmol/L] | -0.143 (-0.296, 0.011) | -0.137 (-0.290, 0.015) | -.042 (-.205, 0.122) | -.064 (-.228, 0.099) | -.061 (-.227, 0.104) | -.075 (-.239, 0.089) |
| Glutamine [µmol/L] | -0.058 (-0.219, 0.102) | -0.061 (-0.221, 0.099) | -.145 (-.317, 0.027) | -.100 (-.270, 0.069) | -.092 (-.262, 0.079) | -.087 (-.258, 0.083) |
| Glycine [µmol/L] | -0.059 (-0.191, 0.073) | -0.054 (-0.187, 0.078) | -.057 (-.198, 0.085) | -.081 (-.222, 0.059) | -.109 (-.251, 0.032) | -.135 (-.274, 0.0038) |
| Histidine [µmol/L] | 0.099 (-0.068, 0.268) | 0.098 (-0.071, 0.266) | 0.133 (-.048, 0.314) | 0.164 (-.0180, 0.345) | 0.154 (-.029, 0.336) | 0.162 (-.020, 0.345) |
| **Branched-chain amino acids** |  |  |  |  |  |  |
| Isoleucine [µmol/L] | -0.148 (-0.289, -0.006) | -0.137 (-0.275, 0.0018) | -.107 (-.255, 0.041) | -.122 (-.271, 0.027) | -.119 (-.269, 0.032) | -.127 (-.277, 0.023) |
| Leucine [µmol/L] | -0.120 (-0.259, 0.019) | -0.123 (-0.259, 0.013) | -.086 (-.232, 0.060) | -.106 (-.252, 0.040) | -.100 (-.249, 0.048) | -.115 (-.262, 0.033) |
| Valine [µmol/L] | -0.173 (-0.308, -0.038) | -0.177 (-0.311, -0.043) | -.144 (-.287, -.00032) | -.159 (-.303, -.0154) | -.158 (-.303, -.0127) | -.170 (-.315, -.026) |
| **Aromatic amino acids** |  |  |  |  |  |  |
| Phenylalanine [µmol/L] | 0.081 (-0.065, 0.227) | 0.085 (-0.057, 0.226) | 0.043 (-.109, 0.195) | 0.021 (-.1301, 0.172) | 0.0034 (-.149, 0.155) | -.029 (-.177, 0.119) |
| Tyrosine [µmol/L] | -0.165 (-0.314, -0.017) | -0.151 (-0.297, -0.004) | -.132 (-.289, 0.026) | -.094 (-.251, 0.063) | -.091 (-.250, 0.068) | -.094 (-.253, 0.065) |
| **Ketone bodies** |  |  |  |  |  |  |
| Acetoacetate [µmol/L] | -0.048 (-0.198, 0.103) | -0.064 (-0.215, 0.087) | -.128 (-.289, 0.034) | -.133 (-.295, 0.029) | -.114 (-.278, 0.049) | -.115 (-.279, 0.048) |
| Acetate [µmol/L] | 0.145 (-0.010, 0.300) | 0.154 (-0.001, 0.309) | 0.232 (0.065, 0.398) | 0.217 (0.051, 0.384) | 0.231 (0.062, 0.399) | 0.222 (0.053, 0.391) |
| beta-hydroxybutyrate [µmol/L] | 0.0063 (-0.139, 0.152) | -0.014 (-0.161, 0.132) | -.087 (-.243, 0.069) | -.096 (-.252, 0.061) | -.082 (-.239, 0.074) | -.097 (-.252, 0.059) |
| **Sphingolipids** |  |  |  |  |  |  |
| Sphingomyelin | -0.016 (-.173, 0.141) | -0.017 (-0.175, 0.141) | -.089 (-.259, 0.079) | -.111 (-.279, 0.059) | -.097 (-.268, 0.073) | -.112 (-.281, 0.057) |
| Total choline | -0.017 (-0.163, 0.129) | -0.016 (-0.163, 0.131) | -.067 (-.224, 0.091) | -.083 (-.239, 0.075) | -.061 (-.219, 0.097) | -.072 (-.229, 0.085) |
| **Glycolysis related metabolites** |  |  |  |  |  |  |
| Citrate [µmol/L] | 0.089 (-0.068, 0.247) | 0.081 (-0.077, 0.239) | 0.056 (-.114, 0.226) | 0.039 (-.131, 0.208) | 0.047 (-.124, 0.218) | 0.033 (-.137, 0.203) |
| Glucose [mmol/L] | -0.021 (-0.174, 0.132) | -0.019 (-0.173, 0.135) | -.070 (-.235, 0.095) | -.096 (-.260, 0.068) | -.083 (-.248, 0.082) | -.090 (-.255, 0.074) |
| Glycerol [mmol/L] | -0.022 (-0.184, 0.139) | -0.018 (-0.181, 0.144) | -.0079 (-.183, 0.167) | 0.021 (-.154, 0.196) | 0.014 (-.163, 0.191) | 0.026 (-.150, 0.202) |
| Lactate [mmol/L] | -0.229 (-0.383, -0.077) | -0.228 (-0.381, -0.075) | -.159 (-.323, 0.0056) | -.169 (-.335, -.0049) | -.164 (-.330, 0.0019) | -.189 (-.352, -.026) |
| Pyruvate [mmol/L] | 0.097 (-0.058, 0.253) | 0.102 (-0.055, 0.258) | 0.062 (-.1049, 0.229) | 0.081 (-.087, 0.248) | 0.068 (-.101, 0.238) | 0.070 (-.099, 0.239) |
| **Fatty acids** |  |  |  |  |  |  |
| Total fatty acids [mmol/L] | -0.035 (-0.187, 0.117) | -0.028 (-0.179, 0.123) | -.054 (-.215, 0.108) | -.068 (-.229, 0.094) | -.049 (-.212, 0.114) | -.059 (-.222, 0.102) |
| Monounsaturated fatty acids [mmol/L] | -0.059 (-0.208, 0.091) | -0.052 (-0.199, 0.095) | -.056 (-.214, 0.102) | -.067 (-.226, 0.091) | -.049 (-.207, 0.110) | -.056 (-.215, 0.102) |
| Saturated fatty acids [mmol/L] | 0.054 (-0.097, 0.204) | 0.061 (-0.088, 0.209) | -.0011 (-.1601, 0.159) | -.015 (-.175, 0.145) | 0.0038 (-.157, 0.165) | -.0095 (-.169, 0.151) |
| **Inflammation** |  |  |  |  |  |  |
| Alpha-1-acid glycoprotein [mmol/L] | -0.105 (-0.263, 0.052) | -0.105 (-.259, 0.049) | -.105 (-.271, 0.061) | -.132 (-.298, 0.034) | -.138 (-.306, 0.029) | -.149 (-.315, 0.018) |
| **Fluid balance** |  |  |  |  |  |  |
| Creatinine [µmol/L] | -0.039 (-0.241, 0.162) | -0.041 (-0.243, 0.161) | 0.031 (-.186, 0.248) | 0.069 (-.147, 0.286) | 0.069 (-.149, 0.289) | 0.077 (-.143, 0.297) |
| Albumin [standardized concentration units] | -0.059 (-0.229, 0.112) | -0.061 (-0.233, 0.110) | -.103 (-.288, 0.081) | -.129 (-.313, 0.055) | -.121 (-.307, 0.065) | -.131 (-.316, 0.054) |

**Table S21. Multivariable regression analyses of pesticide exposure overall with apolipoproteins, aminoacids, sphingomyelins, glucose metabolism, fatty acids, inflammation, and fluid balance with standardised metabolomic biomarkers in women**

| **Pesticide exposure overall, β (95% CI), Females** | | | | | | |
| --- | --- | --- | --- | --- | --- | --- |
|  | **Unadjusted** | **+ BMI** | **+ SEP** | **+ season of pesticide use** | **+ alcohol consumption** | **+ latitude of residence** |
| **Apolipoproteins** |  |  |  |  |  |  |
| Apolipoprotein A1 [g/L] | -0.223 (-0.452, 0.0068) | -0.201 (-0.429, 0.028) | -.250 (-.482, -.018) | -.283 (-.516, -.049) | -.308 (-.545, -.071) | -.303 (-.539, -.066) |
| Apolipoprotein B [g/L] | 0.143 (-0.072, 0.357) | 0.127 (-0.082, 0.337) | 0.063 (-.149, 0.275) | 0.042 (-.170, 0.254) | 0.079 (-.135, 0.293) | 0.082 (-.133, 0.296) |
| Ratio of Apo B/Apo A1 | 0.286 (0.079, 0.492) | 0.256 (0.058, 0.453) | 0.227 (0.027, 0.428) | 0.229 (0.027, 0.430) | 0.282 (0.079, 0.484) | 0.281 (0.079, 0.483) |
| **Amino acids** |  |  |  |  |  |  |
| Alanine [µmol/L] | 0.088 (-0.134, 0.311) | 0.098 (-0.124, 0.319) | 0.109 (-.115, 0.334) | 0.091 (-.135, 0.316) | 0.089 (-.140, 0.318) | 0.087 (-.141, 0.316) |
| Glutamine [µmol/L] | -0.226 (-0.444, -0.008) | -0.209 (-0.428, 0.010) | -.237 (-.459, -.0145) | -.231 (-.454, -.0078) | -.224 (-.451, 0.0023) | -.205 (-.428, 0.019) |
| Glycine [µmol/L] | -0.167 (-0.428, 0.094) | -0.168 (-0.431, 0.094) | -.245 (-.511, 0.020) | -.240 (-.506, 0.026) | -.212 (-.483, 0.059) | -.219 (-.4881, 0.049) |
| Histidine [µmol/L] | -0.075 (-0.296, 0.147) | -0.072 (-0.294, 0.149) | -.026 (-.251, 0.198) | -.031 (-.257, 0.196) | -.051 (-.282, 0.179) | -.048 (-.278, 0.182) |
| **Branched-chain amino acids** |  |  |  |  |  |  |
| Isoleucine [µmol/L] | -0.048 (-0.249, 0.153) | -0.064 (-0.259, 0.132) | -.071 (-.269, 0.128) | -.060 (-.260, 0.139) | -.032 (-.235, 0.172) | -.021 (-.223, 0.181) |
| Leucine [µmol/L] | -0.039 (-0.241, 0.164) | -0.061 (-0.259, 0.137) | -.067 (-.268, 0.134) | -.067 (-.268, 0.134) | -.059 (-.263, 0.146) | -.059 (-.263, 0.144) |
| Valine [µmol/L] | -0.075 (-0.287, 0.136) | -0.095 (-0.304, 0.113) | -.094 (-.306, 0.117) | -.083 (-.294, 0.129) | -.069 (-.284, 0.146) | -.061 (-.275, 0.152) |
| **Aromatic amino acids** |  |  |  |  |  |  |
| Phenylalanine [µmol/L] | 0.224 (-0.002, 0.451) | 0.204 (-0.017, 0.426) | 0.155 (-.069, 0.379) | 0.137 (-.087, 0.360) | 0.113 (-.113, 0.339) | 0.102 (-.122, 0.325) |
| Tyrosine [µmol/L] | -0.034 (-0.251, 0.182) | -0.056 (-0.271, 0.159) | -.055 (-.273, 0.164) | -.055 (-.275, 0.165) | -.055 (-.278, 0.168) | -.035 (-.256, 0.185) |
| **Ketone bodies** |  |  |  |  |  |  |
| Acetoacetate [µmol/L] | -0.196 (-0.428, 0.037) | -0.190 (-0.424, 0.044) | -.235 (-.473, 0.0029) | -.252 (-.491, -.012) | -.264 (-.506, -.022) | -.257 (-.498, -.015) |
| Acetate [µmol/L] | -0.083 (-0.303, 0.137) | -0.087 (-0.309, 0.135) | -.098 (-.322, 0.126) | -.107 (-.332, 0.118) | -.119 (-.347, 0.108) | -.118 (-.345, 0.109) |
| beta-hydroxybutyrate [µmol/L] | -0.172 (-0.417, 0.073) | -0.152 (-0.398, 0.094) | -.210 (-.458, 0.038) | -.235 (-.485, 0.015) | -.229 (-.482, 0.023) | -.238 (-.490, 0.014) |
| **Sphingolipids** |  |  |  |  |  |  |
| Sphingomyelin | -0.065 (-0.299, 0.169) | -0.059 (-0.294, 0.176) | -.137 (-.374, 0.100) | -.173 (-.409, 0.064) | -.171 (-.413, 0.070) | -.168 (-.409, 0.074) |
| Total choline | -0.062 (-0.294, 0.171) | -0.053 (-0.287, 0.180) | -.120 (-.357, 0.116) | -.155 (-.392, 0.082) | -.166 (-.407, 0.075) | -.161 (-.402, 0.079) |
| **Glycolysis related metabolites** |  |  |  |  |  |  |
| Citrate [µmol/L] | 0.139 (-0.084, 0.361) | 0.161 (-0.063, 0.385) | 0.143 (-.085, 0.370) | 0.117 (-.111, 0.346) | 0.096 (-.135, 0.328) | 0.107 (-.123, 0.336) |
| Glucose [mmol/L] | -0.207 (-0.441, 0.028) | -0.207 (-0.443, 0.029) | -.258 (-.497, -.019) | -.249 (-.488, -.012) | -.252 (-.494, -.0096) | -.235 (-.476, 0.0056) |
| Glycerol [mmol/L] | 0.214 (-0.014, 0.441) | 0.215 (-0.013, 0.444) | 0.228 (-.0032, 0.460) | 0.229 (-.0036, 0.462) | 0.226 (-.010, 0.463) | 0.246 (0.010, 0.482) |
| Lactate [mmol/L] | 0.0068 (-0.218, 0.231) | -0.0039 (-0.228, 0.220) | 0.016 (-.211, 0.243) | 0.0085 (-.221, 0.238) | 0.00084 (-.232, 0.233) | -.004 (-.232, 0.224) |
| Pyruvate [mmol/L] | 0.020 (-0.203, 0.244) | 0.023 (-0.201, 0.246) | 0.018 (-.209, 0.245) | 0.013 (-.216, 0.241) | 0.028 (-.204, 0.259) | 0.033 (-.199, 0.265) |
| **Fatty acids** |  |  |  |  |  |  |
| Total fatty acids [mmol/L] | 0.023 (-0.206, 0.252) | 0.023 (-0.205, 0.250) | -.041 (-.271, 0.189) | -.0749 (-.305, 0.157) | -.076 (-.311, 0.159) | -.073 (-.308, 0.162) |
| Monounsaturated fatty acids [mmol/L] | 0.082 (-0.138, 0.303) | 0.075 (-0.142, 0.292) | 0.020 (-.199, 0.240) | -.013 (-.234, 0.207) | -.012 (-.236, 0.211) | -.0093 (-.233, 0.214) |
| Saturated fatty acids [mmol/L] | 0.044 (-0.187, 0.276) | 0.048 (-0.183, 0.278) | -.025 (-.259, 0.208) | -.058 (-.292, 0.176) | -.064 (-.302, 0.174) | -.061 (-.298, 0.177) |
| **Inflammation** |  |  |  |  |  |  |
| Alpha-1-acid glycoprotein [mmol/L] | -0.095 (-0.317, 0.126) | -0.111 (-0.327, 0.106) | -.156 (-.375, 0.063) | -.177 (-.395, 0.041) | -.176 (-.398, 0.045) | -.169 (-.389, 0.052) |
| **Fluid balance** |  |  |  |  |  |  |
| Creatinine [µmol/L] | -0.067 (-0.213, 0.079) | -0.064 (-0.211, 0.083) | -.048 (-.197, 0.101) | -.053 (-.203, 0.097) | -.062 (-.215, 0.090) | -.056 (-.208, 0.097) |
| Albumin [standardized concentration units] | -0.316 (-0.524, -0.109) | -0.311 (-0.519, -0.102) | -.361 (-.572, -.149) | -.359 (-.569, -.149) | -.362 (-.575, -.148) | -.355 (-.566, -.144) |

**Pesticide exposure month**

**Table S22. Multivariable regression analyses of pesticide exposure in months with total lipoprotein subclasses with standardised metabolomic biomarkers in men**

| **Pesticide exposure month, β (95% CI), Males** | | | | | | |
| --- | --- | --- | --- | --- | --- | --- |
| **Total lipoprotein** | **Unadjusted** | **+ BMI** | **+ SEP** | **+ season of pesticide use** | **+ alcohol consumption** | **+ latitude of residence** |
| XXL_VLDL_P | 0.084 (-0.145, 0.313) | 0.121 (-0.102, 0.344) | 0.110 (-.118, 0.339) | 0.119 (-.111, 0.349) | 0.124 (-.108, 0.357) | 0.125 (-.108, 0.357) |
| XL_VLDL_P | 0.002 (-0.227, 0.231) | 0.029 (-0.195, 0.254) | 0.016 (-.214, 0.245) | 0.012 (-.219, 0.244) | 0.013 (-.221, 0.247) | 0.016 (-.218, 0.249) |
| L_VLDL_P | 0.025 (-0.199, 0.248) | 0.047 (-0.171, 0.266) | 0.074 (-.149, 0.297) | 0.069 (-.155, 0.293) | 0.070 (-.156, 0.297) | 0.071 (-.156, 0.297) |
| M_VLDL_P | 0.025 (-0.189, 0.241) | 0.054 (-0.155, 0.263) | 0.072 (-.142, 0.286) | 0.065 (-.151, 0.281) | 0.063 (-.155, 0.281) | 0.064 (-.153, 0.282) |
| S_VLDL_P | 0.002 (-0.199, 0.204) | 0.036 (-0.160, 0.232) | 0.043 (-.158, 0.244) | 0.035 (-.168, 0.237) | 0.031 (-.173, 0.236) | 0.033 (-.172, 0.237) |
| XS_VLDL_P | -0.019 (-0.233, 0.196) | 0.018 (-0.193, 0.229) | -.022 (-.238, 0.195) | -.037 (-.255, 0.180) | -.036 (-.255, 0.184) | -.034 (-.252, 0.184) |
| IDL_P | 0.069 (-0.137, 0.276) | 0.078 (-0.130, 0.285) | 0.093 (-.122, 0.307) | 0.094 (-.122, 0.310) | 0.106 (-.112, 0.324) | 0.105 (-.114, 0.323) |
| L_LDL_P | 0.050 (-0.172, 0.272) | 0.076 (-0.147, 0.298) | 0.0049 (-.223, 0.233) | -.011 (-.239, 0.217) | -.0079 (-.238, 0.222) | -.0047 (-.233, 0.223) |
| M_LDL_P | 0.041 (-0.178, 0.260) | 0.063 (-0.156, 0.282) | 0.00063 (-.224, 0.225) | -.015 (-.239, 0.210) | -.013 (-.239, 0.214) | -.0096 (-.234, 0.215) |
| S_LDL_P | 0.049 (-0.171, 0.269) | 0.068 (-0.152, 0.289) | 0.0098 (-.215, 0.235) | -.0046 (-.231, 0.221) | -.00056 (-.228, 0.227) | 0.0025 (-.224, 0.228) |
| XL_HDL_P | 0.107 (-0.109, 0.325) | 0.086 (-0.128, 0.299) | 0.049 (-.171, 0.268) | 0.062 (-.158, 0.283) | 0.087 (-.136, 0.309) | 0.085 (-.137, 0.308) |
| L_HDL_P | 0.214 (-0.010, 0.437) | 0.1800 (-0.038, 0.399) | 0.167 (-.057, 0.389) | 0.156 (-.069, 0.380) | 0.186 (-.039, 0.411) | 0.189 (-.035, 0.414) |
| M_HDL_P | 0.067 (-0.149, 0.283) | 0.044 (-0.174, 0.261) | 0.074 (-.148, 0.297) | 0.053 (-.170, 0.277) | 0.081 (-.140, 0.303) | 0.086 (-.132, 0.305) |
| S_HDL_P | -0.004 (-0.223, 0.216) | -0.020 (-0.243, 0.202) | -.019 (-.246, 0.208) | -.041 (-.268, 0.186) | -.028 (-.253, 0.198) | -.023 (-.245, 0.200) |

**Table S23. Multivariable regression analyses of pesticide exposure in months with total lipoprotein subclasses with standardised metabolomic biomarkers in women**

| **Pesticide exposure month, β (95% CI), Females** | | | | | | |
| --- | --- | --- | --- | --- | --- | --- |
| **Total lipoprotein** | **Unadjusted** | **+ BMI** | **+ SEP** | **+ season of pesticide use** | **+ alcohol consumption** | **+ latitude of residence** |
| XXL_VLDL_P | 0.211 (-0.048, 0.471) | 0.191 (-0.060, 0.442) | 0.189 (-.065, 0.444) | 0.179 (-.078, 0.437) | 0.186 (-.072, 0.443) | 0.189 (-.067, 0.447) |
| XL_VLDL_P | 0.038 (-0.238, 0.314) | 0.019 (-0.250, 0.287) | 0.010 (-.262, 0.283) | -.012 (-.288, 0.263) | -.016 (-.291, 0.259) | -.013 (-.287, 0.262) |
| L_VLDL_P | 0.120 (-0.154, 0.395) | 0.108 (-0.158, 0.374) | 0.115 (-.155, 0.385) | 0.096 (-.177, 0.369) | 0.097 (-.177, 0.370) | 0.101 (-.172, 0.374) |
| M_VLDL_P | 0.185 (-0.068, 0.438) | 0.164 (-0.079, 0.407) | 0.147 (-.100, 0.395) | 0.131 (-.118, 0.381) | 0.135 (-.114, 0.385) | 0.141 (-.108, 0.389) |
| S_VLDL_P | 0.213 (-0.056, 0.481) | 0.198 (-0.061, 0.457) | 0.161 (-.101, 0.424) | 0.125 (-.139, 0.389) | 0.129 (-.134, 0.393) | 0.129 (-.135, 0.393) |
| XS_VLDL_P | 0.267 (-0.011, 0.545) | 0.258 (-0.015, 0.531) | 0.193 (-.084, 0.469) | 0.157 (-.121, 0.435) | 0.169 (-.108, 0.446) | 0.167 (-.110, 0.444) |
| IDL_P | -0.037 (-0.361, 0.287) | -0.011 (-0.339, 0.316) | 0.011 (-.322, 0.345) | 0.013 (-.324, 0.351) | 0.012 (-.324, 0.347) | 0.013 (-.322, 0.349) |
| L_LDL_P | 0.229 (-0.049, 0.508) | 0.220 (-0.058, 0.499) | 0.145 (-.136, 0.426) | 0.105 (-.177, 0.386) | 0.116 (-.165, 0.396) | 0.113 (-.167, 0.394) |
| M_LDL_P | 0.241 (-0.039, 0.519) | 0.231 (-0.047, 0.509) | 0.161 (-.120, 0.442) | 0.121 (-.159, 0.403) | 0.131 (-.149, 0.411) | 0.128 (-.152, 0.408) |
| S_LDL_P | 0.251 (-0.029, 0.531) | 0.242 (-0.037, 0.521) | 0.176 (-.106, 0.458) | 0.136 (-.147, 0.419) | 0.143 (-.139, 0.425) | 0.140 (-.142, 0.422) |
| XL_HDL_P | -0.024 (-0.269, 0.221) | 0.013 (-0.228, 0.255) | -.0089 (-.255, 0.237) | -.047 (-.295, 0.200) | -.049 (-.297, 0.198) | -.055 (-.299, 0.189) |
| L_HDL_P | -0.273 (-0.517, -0.028) | -0.236 (-0.475, 0.003) | -.266 (-.508, -.024) | -.306 (-.551, -.061) | -.318 (-.563, -.074) | -.318 (-.563, -.074) |
| M_HDL_P | -0.308 (-0.589, -0.026) | -0.302 (-0.587, -0.018) | -.331 (-.620, -.043) | -.3541 (-.646, -.062) | -.378 (-.669, -.087) | -.372 (-.661, -.083) |
| S_HDL_P | -0.235 (-0.529, 0.059) | -0.238 (-0.536, 0.059) | -.290 (-.591, 0.010) | -.319 (-.622, -.017) | -.340 (-.643, -.0374) | -.340 (-.641, -.040) |

**Table S24. Multivariable regression analyses of pesticide exposure in months with triglyceride subclasses with standardised metabolomic biomarkers in men**

| **Pesticide exposure month, β (95% CI), Males** | | | | | | |
| --- | --- | --- | --- | --- | --- | --- |
| **Triglycerides** | **Unadjusted** | **+ BMI** | **+ SEP** | **+ season of pesticide use** | **+ alcohol consumption** | **+ latitude of residence** |
| XXL_VLDL_TG | 0.080 (-0.146, 0.306) | 0.115 (-0.105, 0.335) | 0.105 (-.119, 0.331) | 0.116 (-.111, 0.342) | 0.122 (-.107, 0.351) | 0.122 (-.107, 0.351) |
| XL_VLDL_TG | -0.025 (-0.247, 0.197) | -0.003 (-0.221, 0.215) | -.011 (-.234, 0.212) | -.017 (-.241, 0.208) | -.017 (-.244, 0.210) | -.014 (-.241, 0.213) |
| L_VLDL_TG | 0.027 (-0.193, 0.246) | 0.044 (-0.171, 0.259) | 0.079 (-.141, 0.298) | 0.073 (-.148, 0.293) | 0.074 (-.148, 0.297) | 0.074 (-.148, 0.297) |
| M_VLDL_TG | 0.011 (-0.196, 0.217) | 0.032 (-0.169, 0.233) | 0.059 (-.147, 0.265) | 0.052 (-.156, 0.259) | 0.049 (-.159, 0.258) | 0.050 (-.158, 0.259) |
| S_VLDL_TG | 0.009 (-0.188, 0.204) | 0.035 (-0.155, 0.226) | 0.057 (-.138, 0.253) | 0.050 (-.147, 0.247) | 0.045 (-.153, 0.244) | 0.046 (-.152, 0.245) |
| XS_VLDL_TG | -0.0004 (-0.201, 0.201) | 0.028 (-0.168, 0.223) | 0.029 (-.171, 0.229) | 0.016 (-.185, 0.218) | 0.012 (-.192, 0.216) | 0.014 (-.189, 0.216) |
| VLDL_TG | 0.019 (-0.194, 0.231) | 0.043 (-0.163, 0.249) | 0.064 (-.147, 0.274) | 0.057 (-.156, 0.269) | 0.055 (-.159, 0.269) | 0.056 (-.158, 0.270) |
| IDL_TG | 0.012 (-0.196, 0.219) | 0.034 (-0.169, 0.238) | 0.0088 (-.200, 0.218) | -.0069 (-.216, 0.202) | -.0083 (-.219, 0.203) | -.0063 (-.215, 0.203) |
| L_LDL_TG | 0.044 (-0.167, 0.255) | 0.061 (-.148, 0.269) | 0.018 (-.195, 0.232) | 0.0039 (-.209, 0.217) | 0.004 (-.211, 0.219) | 0.0062 (-.206, 0.218) |
| M_LDL_TG | 0.063 (-0.149, 0.275) | 0.074 (-0.136, 0.284) | 0.032 (-.183, 0.247) | 0.020 (-.195, 0.236) | 0.019 (-.198, 0.236) | 0.021 (-.193, 0.235) |
| S_LDL_TG | 0.060 (-0.149, 0.270) | 0.076 (-0.131, 0.283) | 0.049 (-.162, 0.261) | 0.038 (-.174, 0.250) | 0.037 (-.177, 0.251) | 0.039 (-.173, 0.251) |
| LDL_TG | 0.051 (-0.160, 0.263) | 0.066 (-0.143, 0.275) | 0.026 (-.188, 0.239) | 0.013 (-.201, 0.227) | 0.013 (-.203, 0.229) | 0.015 (-.198, 0.228) |
| XL_HDL_TG | 0.235 (0.015, 0.456) | 0.262 (0.039, 0.484) | 0.194 (-.033, 0.421) | 0.188 (-.041, 0.416) | 0.189 (-.042, 0.419) | 0.191 (-.038, 0.420) |
| L_HDL_TG | 0.225 (-0.004, 0.454) | 0.224 (-0.008, 0.456) | 0.192 (-.045, 0.429) | 0.179 (-.059, 0.416) | 0.183 (-.056, 0.422) | 0.193 (-.043, 0.429) |
| M_HDL_TG | 0.048 (-0.169, 0.265) | 0.074 (-0.140, 0.289) | 0.075 (-.144, 0.295) | 0.073 (-.149, 0.295) | 0.077 (-.146, 0.299) | 0.079 (-.142, 0.302) |
| S_HDL_TG | -0.005 (-0.200, 0.190) | 0.017 (-0.173, 0.206) | -.012 (-.206, 0.181) | -.0087 (-.203, 0.186) | -.018 (-.214, 0.178) | -.018 (-.212, 0.177) |
| HDL_TG | 0.136 (-0.079, 0.351) | 0.159 (-0.056, 0.374) | 0.127 (-.092, 0.347) | 0.120 (-.101, 0.341) | 0.12 (-.101, 0.344) | 0.124 (-.097, 0.345) |
| Serum_TG | 0.036 (-0.185, 0.257) | 0.063 (-0.151, 0.277) | 0.071 (-.148, 0.290) | 0.062 (-.159, 0.283) | 0.062 (-.161, 0.285) | 0.064 (-.158, 0.287) |

**Table S25. Multivariable regression analyses of pesticide exposure in months with triglyceride subclasses with standardised metabolomic biomarkers in women**

| **Pesticide exposure month, β (95% CI), Females** | | | | | | |
| --- | --- | --- | --- | --- | --- | --- |
| **Triglycerides** | **Unadjusted** | **+ BMI** | **+ SEP** | **+ season of pesticide use** | **+ alcohol consumption** | **+ latitude of residence** |
| XXL_VLDL_TG | 0.219 (-0.039, 0.478) | 0.197 (-0.052, 0.447) | 0.198 (-.055, 0.452) | 0.192 (-.064, 0.448) | 0.197 (-.059, 0.453) | 0.201 (-.055, 0.457) |
| XL_VLDL_TG | 0.030 (-0.252, 0.312) | 0.009 (-0.267, 0.286) | 0.0048 (-.275, 0.285) | -.019 (302, 0.263) | -.023 (-.305, 0.259) | -.020 (-.301, 0.261) |
| L_VLDL_TG | 0.114 (-.163, 0.391) | 0.100 (-0.169, 0.369) | 0.111 (-.162, 0.385) | 0.093 (-.184, 0.369) | 0.093 (-.184, 0.371) | 0.098 (-.178, 0.375) |
| M_VLDL_TG | 0.168 (-0.096, 0.431) | 0.146 (-0.109, 0.401) | 0.139 (-.119, 0.398) | 0.129 (-.132, 0.389) | 0.129 (-.132, 0.391) | 0.136 (-.124, 0.397) |
| S_VLDL_TG | 0.233 (-0.052, 0.518) | 0.221 (-0.056, 0.497) | 0.197 (-.085, 0.478) | 0.167 (-.116, 0.451) | 0.166 (-.118, 0.449) | 0.166 (-.118, 0.449) |
| XS_VLDL_TG | 0.238 (-0.052, 0.528) | 0.237 (-0.046, 0.519) | 0.188 (-.099, 0.475) | 0.142 (-.147, 0.430) | 0.142 (-.147, 0.430) | 0.139 (-.149, 0.427) |
| VLDL_TG | 0.193 (-0.071, 0.457) | 0.174 (-0.081, 0.428) | 0.158 (-.100, 0.417) | 0.136 (-.124, 0.397) | 0.136 (-.125, 0.396) | 0.140 (-.119, 0.400) |
| IDL_TG | 0.279 (-0.013, 0.572) | 0.286 (-0.002, 0.574) | 0.220 (-.072, 0.512) | 0.165 (-.129, 0.458) | 0.165 (-.128, 0.459) | 0.159 (-.133, 0.452) |
| L_LDL_TG | 0.252 (-0.044, 0.548) | 0.261 (-0.033, 0.554) | 0.187 (-.111, 0.484) | 0.127 (-.171, 0.426) | 0.126 (-.173, 0.425) | 0.119 (-.179, 0.417) |
| M_LDL_TG | 0.251 (-0.039, 0.542) | 0.256 (-0.034, 0.545) | 0.194 (-.099, 0.487) | 0.139 (-.155, 0.435) | 0.135 (-.159, 0.429) | 0.127 (-.166, 0.421) |
| S_LDL_TG | 0.244 (-0.047, 0.536) | 0.241 (-0.047, 0.529) | 0.187 (-.105, 0.479) | 0.136 (-.157, 0.429) | 0.131 (-.162, 0.423) | 0.125 (-.167, 0.418) |
| LDL_TG | 0.249 (-0.045, 0.542) | 0.254 (-0.037, 0.545) | 0.184 (-.111, 0.479) | 0.127 (-.169, 0.423) | 0.124 (-.172, 0.419) | 0.117 (-.178, 0.412) |
| XL_HDL_TG | 0.009 (-0.275, 0.295) | 0.042 (-0.244, 0.328) | -.022 (-.312, 0.269) | -.074 (-.367, 0.219) | -.067 (-.359, 0.225) | -.068 (-.361, 0.224) |
| L_HDL_TG | -0.094 (-0.371, 0.184) | -0.060 (-0.339, 0.219) | -.127 (-.411, 0.156) | -.179 (-.464, 0.105) | -.181 (-.466, 0.104) | -.185 (-.469, 0.099) |
| M_HDL_TG | 0.118 (-0.162, 0.398) | 0.107 (-0.172, 0.385) | 0.061 (-.222, 0.343) | 0.023 (-.262, 0.307) | 0.019 (-.267, 0.304) | 0.019 (-.266, 0.305) |
| S_HDL_TG | 0.271 (-0.029, 0.571) | 0.268 (-0.027, 0.563) | 0.217 (-.083, 0.517) | 0.167 (-.134, 0.469) | 0.169 (-.134, 0.471) | 0.160 (-.141, 0.462) |
| HDL_TG | 0.060 (-0.234, 0.354) | 0.075 (-0.220, 0.369) | 0.0043 (-.294, 0.303) | -.058 (-.358, 0.242) | -.059 (-.359, 0.242) | -.062 (-.362, 0.238) |
| Serum_TG | 0.209 (-0.052, 0.472) | 0.200 (-0.053, 0.454) | 0.165 (-.092, 0.422) | 0.127 (-.132, 0.385) | 0.126 (-.133, 0.385) | 0.127 (-.132, 0.386) |

**Table S26. Multivariable regression analyses of pesticide exposure in months with phospholipid subclasses with standardised metabolomic biomarkers in men**

| **Pesticide exposure month, β (95% CI), Males** | | | | | | |
| --- | --- | --- | --- | --- | --- | --- |
| **Phospholipids** | **Unadjusted** | **+ BMI** | **+ SEP** | **+ season of pesticide use** | **+ alcohol consumption** | **+ latitude of residence** |
| XXL_VLDL_PL | 0.075 (-0.144, 0.293) | 0.106 (-0.109, 0.321) | 0.105 (-.115, 0.325) | 0.106 (-.115, 0.328) | 0.114 (-.109, 0.338) | 0.116 (-.108, 0.340) |
| XL_VLDL_PL | 0.066 (-0.160, 0.293) | 0.094 (-0.129, 0.317) | 0.076 (-.152, 0.304) | 0.073 (-.157, 0.303) | 0.079 (-.154, 0.311) | 0.082 (-.150, 0.315) |
| L_VLDL_PL | 0.064 (-0.157, 0.286) | 0.089 (-0.128, 0.306) | 0.112 (-.109, 0.333) | 0.107 (-.116, 0.329) | 0.109 (-.115, 0.335) | 0.110 (-.115, 0.335) |
| M_VLDL_PL | 0.033 (-0.186, 0.251) | 0.064 (-0.148, 0.277) | 0.076 (-.142, 0.293) | 0.068 (-.152, 0.287) | 0.066 (-.155, 0.287) | 0.068 (-.153, 0.289) |
| S_VLDL_PL | -0.016 (-0.221, 0.189) | 0.018 (-0.182, 0.218) | 0.028 (-.176, 0.233) | 0.020 (-.186, 0.227) | 0.019 (-.189, 0.227) | 0.019 (-.188, 0.227) |
| XS_VLDL_PL | -0.022 (-0.243, 0.199) | 0.011 (-0.209, 0.231) | -.035 (-.261, 0.191) | -.054 (-.280, 0.172) | -.052 (-.280, 0.177) | -.049 (-.276, 0.178) |
| IDL_PL | 0.029 (-0.201, 0.259) | 0.056 (-0.175, 0.287) | -.023 (-.259, 0.213) | -.041 (-.277, 0.196) | -.037 (-.275, 0.202) | -.034 (-.270, 0.203) |
| L_LDL_PL | 0.057 (-0.168, 0.282) | 0.081 (-0.145, 0.307) | 0.0093 (-.222, 0.240) | -.0089 (-.240, 0.223) | -.0026 (-.236, 0.231) | 0.00091 (-.230, 0.232) |
| M_LDL_PL | 0.081 (-0.146, 0.308) | 0.105 (-0.122, 0.331) | 0.041 (-.191, 0.272) | 0.025 (-.207, 0.257) | 0.034 (-.200, 0.268) | 0.037 (-.195, 0.269) |
| S_LDL_PL | 0.101 (-0.122, 0.324) | 0.118 (-0.105, 0.341) | 0.058 (-.169, 0.286) | 0.044 (-.185, 0.272) | 0.057 (-.173, 0.287) | 0.060 (-.168, 0.288) |
| XL_HDL_PL | 0.006 (-0.231, 0.243) | -0.023 (-0.252, 0.205) | -.038 (-.273, 0.197) | -.034 (-.270, 0.203) | -.017 (-.255, 0.220) | -.018 (-.256, 0.219) |
| L_HDL_PL | 0.182 (-0.044, 0.408) | 0.147 (-0.074, 0.369) | 0.141 (-.085, 0.368) | 0.129 (-.099, 0.357) | 0.160 (-.068, 0.389) | 0.164 (-.064, 0.391) |
| M_HDL_PL | 0.065 (-0.151, 0.282) | 0.041 (-0.177, 0.258) | 0.066 (-.157, 0.289) | 0.045 (-.178, 0.269) | 0.076 (-.145, 0.297) | 0.081 (-.137, 0.299) |
| S_HDL_PL | 0.014 (-0.196, 0.224) | -0.008 (-0.221, 0.204) | 0.020 (-.197, 0.237) | 0.000019 (-.218, 0.218) | 0.020 (-.195, 0.235) | 0.025 (-.187, 0.238) |

**Table S27. Multivariable regression analyses of pesticide exposure in months with phospholipid subclasses with standardised metabolomic biomarkers in women**

| **Pesticide exposure month, β (95% CI), Females** | | | | |  |  |
| --- | --- | --- | --- | --- | --- | --- |
| **Phospholipids** | **Unadjusted** | **+ BMI** | **+ SEP** | **+ season of pesticide use** | **+ alcohol consumption** | **+ latitude of residence** |
| XXL_VLDL_PL | 0.092 (-0.175, 0.359) | 0.074 (-0.188, 0.336) | 0.076 (-.189, 0.342) | 0.061 (-.208, 0.329) | 0.062 (-.208, 0.331) | 0.068 (-.201, 0.337) |
| XL_VLDL_PL | 0.058 (-0.226, 0.342) | 0.036 (-0.242, 0.315) | 0.018 (-.263, 0.299) | -.0054 (-.289, 0.279) | -.0073 (-.292, 0.278) | -.0047 (-.289, 0.279) |
| L_VLDL_PL | 0.058 (-0.218, 0.334) | 0.045 (-0.222, 0.312) | 0.042 (-.229, 0.313) | 0.017 (-.258, 0.291) | 0.018 (-.257, 0.293) | 0.022 (-.253, 0.296) |
| M_VLDL_PL | 0.193 (-0.064, 0.449) | 0.172 (-0.075, 0.419) | 0.148 (-.104, 0.399) | 0.126 (-.127, 0.379) | 0.130 (-.123, 0.383) | 0.135 (-.118, 0.387) |
| S_VLDL_PL | 0.215 (-0.068, 0.497) | 0.202 (-0.072, 0.477) | 0.164 (-.114, 0.443) | 0.123 (-.157, 0.403) | 0.124 (-.155, 0.404) | 0.122 (-.157, 0.402) |
| XS_VLDL_PL | 0.269 (-0.012, 0.549) | 0.261 (-0.018, 0.539) | 0.193 (-.089, 0.474) | 0.159 (-.124, 0.442) | 0.170 (-.112, 0.452) | 0.169 (-.113, 0.451) |
| IDL_PL | 0.212 (-0.069, 0.493) | 0.204 (-0.077, 0.485) | 0.126 (-.158, 0.409) | 0.086 (-.199, 0.371) | 0.099 (-.184, 0.383) | 0.098 (-.185, 0.382) |
| L_LDL_PL | 0.181 (-0.094, 0.457) | 0.172 (-0.103, 0.448) | 0.097 (-.181, 0.375) | 0.062 (-.217, 0.340) | 0.071 (-.207, 0.349) | 0.070 (-.207, 0.348) |
| M_LDL_PL | 0.218 (-0.061, 0.498) | 0.212 (-0.066, 0.489) | 0.133 (-.147, 0.414) | 0.092 (-.189, 0.373) | 0.099 (-.182, 0.379) | 0.096 (-.184, 0.376) |
| S_LDL_PL | 0.216 (-0.062, 0.494) | 0.214 (-0.063, 0.490) | 0.139 (-.139, 0.419) | 0.094 (-.186, 0.375) | 0.095 (-.186, 0.376) | 0.092 (-.188, 0.373) |
| XL_HDL_PL | -0.038 (-0.240, 0.164) | -0.006 (-0.203, 0.191) | -.019 (-.220, 0.180) | -.048 (-.249, 0.155) | -.051 (-.253, 0.151) | -.054 (-.254, 0.147) |
| L_HDL_PL | -0.293 (-0.539, -0.046) | -0.259 (-0.501, -0.019) | -.288 (-.533, -.043) | -.326 (-.574, -.078) | -.340 (-.587, -.093) | -.339 (-.586, -.091) |
| M_HDL_PL | -0.312 (-0.599, -0.025) | -0.303 (-0.591, -0.014) | -.336 (-.629, -.043) | -.369 (-.665, -.073) | -.393 (-.689, -.098) | -.388 (-.682, -.094) |
| S_HDL_PL | -0.481 (-0.767, -0.194) | -0.484 (-0.774, -0.194) | -.500 (-.795, -.206) | -.509 (-.807, -.213) | -.536 (-.832, -.239) | -.532 (-.826, -.238) |

**Table S28. Multivariable regression analyses of pesticide exposure in months with cholesterol esters subclasses with standardised metabolomic biomarkers in men**

| **Pesticide exposure month, β (95% CI), Males** | | | | | | |
| --- | --- | --- | --- | --- | --- | --- |
| **Cholesterol esters** | **Unadjusted** | **+ BMI** | **+ SEP** | **+ season of pesticide use** | **+ alcohol consumption** | **+ latitude of residence** |
| XXL_VLDL_CE | 0.091 (-0.119, 0.301) | 0.135 (-0.072, 0.341) | 0.126 (-.085, 0.338) | 0.135 (-.078, 0.348) | 0.131 (-.085, 0.346) | 0.129 (-.086, 0.345) |
| XL_VLDL_CE | -0.023 (-0.241, 0.194) | 0.019 (-0.195, 0.233) | -.0096 (-.229, 0.209) | -.0024 (-.223, 0.218) | -.011 (-.234, 0.212) | -.011 (-.234, 0.212) |
| L_VLDL_CE | -0.007 (-0.228, 0.213) | 0.031 (-0.187, 0.249) | 0.059 (-.164, 0.282) | 0.064 (-.159, 0.286) | 0.059 (-.166, 0.283) | 0.059 (-.166, 0.283) |
| M_VLDL_CE | 0.052 (-0.170, 0.274) | 0.098 (-0.119, 0.315) | 0.086 (-.137, 0.308) | 0.082 (-.142, 0.306) | 0.078 (-.148, 0.304) | 0.079 (-.147, 0.305) |
| S_VLDL_CE | -0.004 (-0.211, 0.203) | 0.029 (-0.175, 0.234) | -.0063 (-.216, 0.203) | -.014 (-.225, 0.197) | -.017 (-.230, 0.196) | -.016 (-.228, 0.196) |
| XS_VLDL_CE | -0.041 (-0.261, 0.178) | -0.006 (-0.225, 0.213) | -.073 (-.297, 0.151) | -.082 (-.308, 0.143) | -.077 (-.304, 0.149) | -.077 (-.302, 0.149) |
| IDL_CE | 0.041 (-0.179, 0.261) | 0.071 (-0.149, 0.291) | -.0074 (-.233, 0.218) | -.021 (-.248, 0.205) | -.013 (-.241, 0.215) | -.010 (-.237, 0.216) |
| L_LDL_CE | 0.044 (-0.177, 0.265) | 0.070 (-0.151, 0.292) | -.00040 (-.227, 0.226) | -.015 (-.243, 0.212) | -.013 (-.242, 0.216) | -.0099 (-.237, 0.217) |
| M_LDL_CE | 0.015 (-0.204, 0.233) | 0.034 (-0.186, 0.254) | -.027 (-.252, 0.198) | -.041 (-.267, 0.185) | -.043 (-.271, 0.185) | -.039 (-.267, 0.187) |
| S_LDL_CE | 0.006 (-0.219, 0.231) | 0.022 (-0.205, 0.249) | -.038 (-.270, 0.195) | -.051 (-.285, 0.183) | -.052 (-.288, 0.184) | -.049 (-.284, 0.185) |
| XL_HDL_CE | 0.118 (-0.107, 0.343) | 0.102 (-0.122, 0.327) | 0.059 (-.171, 0.289) | 0.086 (-.146, 0.319) | 0.109 (-.126, 0.344) | 0.107 (-.126, 0.341) |
| L_HDL_CE | 0.224 (-0.007, 0.455) | 0.189 (-0.035, 0.414) | 0.174 (-.056, 0.403) | 0.165 (-.066, 0.396) | 0.195 (-.038, 0.428) | 0.198 (-.035, 0.429) |
| M_HDL_CE | 0.057 (-0.163, 0.277) | 0.029 (-0.191, 0.249) | 0.067 (-.158, 0.292) | 0.046 (-.179, 0.272) | 0.072 (-.153, 0.296) | 0.076 (-.145, 0.298) |
| S_HDL_CE | -0.099 (-0.343, 0.144) | -0.114 (-0.361, 0.133) | -.145 (-.398, 0.108) | -.163 (-.417, 0.091) | -.166 (-.422, 0.091) | -.162 (-.417, 0.093) |
| EstC | 0.088 (-0.137, 0.313) | 0.104 (-0.123, 0.331) | 0.049 (-.183, 0.280) | 0.035 (-.197, 0.268) | 0.051 (-.183, 0.286) | 0.056 (-.176, 0.288) |

**Table S29. Multivariable regression analyses of pesticide exposure in months with cholesterol esters subclasses with standardised metabolomic biomarkers in women**

| **Pesticide exposure month, β (95% CI), Females** | | | | | | |
| --- | --- | --- | --- | --- | --- | --- |
| **Cholesterol esters** | **Unadjusted** | **+ BMI** | **+ SEP** | **+ season of pesticide use** | **+ alcohol consumption** | **+ latitude of residence** |
| XXL_VLDL_CE | 0.297 (0.009, 0.585) | 0.279 (-0.004, 0.562) | 0.263 (-.024, 0.551) | 0.251 (-.039, 0.542) | 0.269 (-.021, 0.559) | 0.272 (-.018, 0.561) |
| XL_VLDL_CE | 0.033 (-0.263, 0.329) | 0.015 (-0.277, 0.308) | -.00035 (-.295, 0.294) | -.018 (-.316, 0.279) | -.016 (-.311, 0.279) | -.014 (-.309, 0.280) |
| L_VLDL_CE | 0.137 (-0.152, 0.427) | 0.124 (-0.159, 0.408) | 0.119 (-.169, 0.408) | 0.104 (-.187, 0.396) | 0.113 (-.179, 0.405) | 0.112 (-.180, 0.404) |
| M_VLDL_CE | 0.270 (0.008, 0.533) | 0.250 (-0.006, 0.506) | 0.209 (-.049, 0.46 9) | 0.187 (-.075, 0.449) | 0.202 (-.059, 0.464) | 0.205 (-.056, 0.466) |
| S_VLDL_CE | 0.191 (-0.083, 0.465) | 0.172 (-0.096, 0.441) | 0.117 (-.155, 0.389) | 0.083 (-.189, 0.357) | 0.099 (-.172, 0.371) | 0.097 (-.175, 0.369) |
| XS_VLDL_CE | 0.238 (-0.030, 0.507) | 0.224 (-0.043, 0.491) | 0.167 (-.103, 0.438) | 0.154 (-.119, 0.427) | 0.174 (-.097, 0.445) | 0.171 (-.099, 0.442) |
| IDL_CE | 0.217 (-0.057, 0.491) | 0.207 (-0.067, 0.480) | 0.136 (-.140, 0.413) | 0.107 (-.171, 0.385) | 0.122 (-.155, 0.398) | 0.121 (-.156, 0.398) |
| L_LDL_CE | 0.231 (-0.051, 0.513) | 0.218 (-0.063, 0.500) | 0.148 (-.136, 0.433) | 0.116 (-.169, 0.401) | 0.129 (-.156, 0.413) | 0.127 (-.157, 0.411) |
| M_LDL_CE | 0.232 (-0.049, 0.512) | 0.218 (-0.063, 0.499) | 0.159 (-.125, 0.444) | 0.131 (-.154, 0.416) | 0.143 (-.139, 0.426) | 0.142 (-.140, 0.425) |
| S_LDL_CE | 0.237 (-0.037, 0.511) | 0.226 (-0.049, 0.500) | 0.173 (-.104, 0.451) | 0.145 (-.133, 0.424) | 0.157 (-.119, 0.434) | 0.156 (-.121, 0.433) |
| XL_HDL_CE | 0.039 (-0.228, 0.306) | 0.067 (-0.201, 0.335) | 0.046 (-.227, 0.318) | 0.010 (-.265, 0.285) | 0.011 (-.265, 0.286) | 0.00043 (-.269, 0.270) |
| L_HDL_CE | -0.247 (-0.474, -0.021) | -0.211 (-0.429, 0.009) | -.231 (-.454, -.0085) | -.266 (-.491, -.041) | -.277 (-.502, -.053) | -.278 (-.503, -.053) |
| M_HDL_CE | -0.327 (-0.602, -0.052) | -0.325 (-0.601, -0.048) | -.336 (-.617, -.055) | -.337 (-.620, -.053) | -.360 (-.643, -.077) | -.354 (-.634, -.073) |
| S_HDL_CE | 0.011 (-0.244, 0.265) | 0.003 (-0.253, 0.259) | -.051 (-.309, 0.208) | -.076 (-.335, 0.182) | -.084 (-.343, 0.176) | -.087 (-.345, 0.171) |
| EstC | 0.132 (-0.146, 0.409) | 0.133 (-0.146, 0.412) | 0.062 (-.220, 0.344) | 0.016 (-.266, 0.299) | 0.019 (-.264, 0.303) | 0.018 (-.266, 0.301) |

**Table S30. Multivariable regression analyses of pesticide exposure in months with free cholesterol subclasses with standardised metabolomic biomarkers in men**

| **Pesticide exposure month, β (95% CI), Males** | | | | | | |
| --- | --- | --- | --- | --- | --- | --- |
| **Free cholesterol** | **Unadjusted** | **+ BMI** | **+ SEP** | **+ season of pesticide use** | **+ alcohol consumption** | **+ latitude of residence** |
| XXL_VLDL_FC | 0.081 (-0.138, 0.301) | 0.109 (-0.105, 0.325) | 0.098 (-.123, 0.318) | 0.099 (-.123, 0.321) | 0.101 (-.123, 0.326) | 0.104 (-.120, 0.328) |
| XL_VLDL_FC | 0.084 (-0.140, 0.309) | 0.117 (-0.103, 0.338) | 0.091 (-.135, 0.317) | 0.091 (-.137, 0.319) | 0.094 (-.137, 0.325) | 0.097 (-.134, 0.327) |
| L_VLDL_FC | -0.004 (-0.219, 0.211) | 0.018 (-0.193, 0.229) | 0.038 (-.178, 0.254) | 0.032 (-.186, 0.249) | 0.032 (-.188, 0.252) | 0.033 (-.187, 0.253) |
| M_VLDL_FC | 0.019 (-0.194, 0.234) | 0.048 (-0.159, 0.256) | 0.060 (-.153, 0.274) | 0.052 (-.163, 0.267) | 0.049 (-.168, 0.266) | 0.051 (-.166, 0.268) |
| S_VLDL_FC | -0.008 (-0.209, 0.192) | 0.024 (-0.172, 0.220) | 0.024 (-.177, 0.226) | 0.014 (-.189, 0.217) | 0.012 (-.192, 0.217) | 0.014 (-.190, 0.218) |
| XS_VLDL_FC | -0.053 (-0.274, 0.168) | -0.020 (-0.241, 0.200) | -.063 (-.289, 0.163) | -.084 (-.311, 0.144) | -.085 (-.315, 0.144) | -.083 (-.311, 0.144) |
| IDL_FC | 0.011 (-0.221, 0.244) | 0.036 (-0.199, 0.271) | -.041 (-.281, 0.199) | -.059 (-.299, 0.183) | -.056 (-.299, 0.187) | -.053 (-.295, 0.188) |
| L_LDL_FC | 0.036 (-0.192, 0.265) | 0.058 (-0.172, 0.289) | -.026 (-.261, 0.209) | -.042 (-.279, 0.194) | -.039 (-.278, 0.198) | -.037 (-.273, 0.199) |
| M_LDL_FC | 0.064 (-0.155, 0.284) | 0.085 (-0.136, 0.306) | 0.010 (-.215, 0.236) | -.0047 (-.231, 0.221) | 0.00058 (-.227, 0.228) | 0.0036 (-.222, 0.229) |
| S_LDL_FC | 0.078 (-0.146, 0.302) | 0.098 (-0.128, 0.323) | 0.023 (-.207, 0.253) | 0.0094 (-.221, 0.240) | 0.017 (-.216, 0.249) | 0.019 (-.211, 0.249) |
| XL_HDL_FC | 0.110 (-0.128, 0.348) | 0.095 (-0.138, 0.328) | 0.048 (-.192, 0.287) | 0.057 (-.184, 0.298) | 0.081 (-.163, 0.325) | 0.079 (-.164, 0.322) |
| L_HDL_FC | 0.165 (-0.075, 0.406) | 0.131 (-0.102, 0.364) | 0.111 (-.127, 0.349) | 0.103 (-.137, 0.342) | 0.130 (-.112, 0.372) | 0.133 (-.109, 0.374) |
| M_HDL_FC | 0.035 (-0.191, 0.261) | 0.010 (-0.216, 0.237) | 0.036 (-.195, 0.268) | 0.014 (-.218, 0.247) | 0.040 (-.191, 0.272) | 0.045(-.184, 0.274) |
| S_HDL_FC | -0.003 (-0.227, 0.222) | -0.022 (-0.249, 0.205) | -.019 (-.251, 0.213) | -.042 (-.275, 0.190) | -.025 (-.256, 0.206) | -.020 (-.249, 0.208) |
| Free cholesterol | 0.062 (-0.166, 0.289) | 0.079 (-0.150, 0.309) | 0.010 (-.225, 0.245) | -.012 (-.248, 0.224) | -.0026 (-.241, 0.235) | 0.0019 (-.234, 0.238) |

**Table S31. Multivariable regression analyses of pesticide exposure in months with free cholesterol subclasses with standardised metabolomic biomarkers in women**

| **Pesticide exposure month, β (95% CI), females** | | | | | | |
| --- | --- | --- | --- | --- | --- | --- |
| **Free cholesterol** | **Unadjusted** | **+ BMI** | **+ SEP** | **+ season of pesticide use** | **+ alcohol consumption** | **+ latitude of residence** |
| XXL_VLDL_FC | 0.111 (-0.160, 0.383) | 0.096 (-0.169, 0.362) | 0.084 (-.186, 0.353) | 0.064 (-.209, 0.337) | 0.066 (-.207, 0.339) | 0.072 (-.201, 0.345) |
| XL_VLDL_FC | 0.034 (-0.248, 0.316) | 0.014 (-0.262, 0.291) | -.0082 (-.285, 0.268) | -.030 (-.310, 0.249) | -.029 (-.309, 0.251) | -.027 (-.308, 0.253) |
| L_VLDL_FC | 0.017 (-0.272, 0.306) | 0.006 (-0.275, 0.288) | 0.00086 (-.285, 0.287) | -.028 (-.318, 0.261) | -.028 (-.318, 0.262) | -.024 (-.313, 0.266) |
| M_VLDL_FC | 0.174 (-0.091, 0.438) | 0.156 (-0.100, 0.411) | 0.128 (-.131, 0.388) | 0.101 (-.161, 0.363) | 0.104 (-.158, 0.366) | 0.108 (-.153, 0.369) |
| S_VLDL_FC | 0.175 (-0.105, 0.454) | 0.165 (-0.107, 0.436) | 0.118 (-.158, 0.394) | 0.075 (-.202, 0.352) | 0.078 (-.199, 0.354) | 0.076 (-.201, 0.352) |
| XS_VLDL_FC | 0.213 (-0.062, 0.487) | 0.206 (-0.066, 0.479) | 0.145 (-.132, 0.421) | 0.112 (-.166, 0.390) | 0.125 (-.153, 0.402) | 0.123 (-.155, 0.399) |
| IDL_FC | 0.215 (-0.062, 0.491) | 0.210 (-0.067, 0.487) | 0.137 (-.144, 0.417) | 0.100 (-.181, 0.382) | 0.114 (-.166, 0.394) | 0.113 (-.167, 0.393) |
| L_LDL_FC | 0.214 (-0.060, 0.489) | 0.208 (-0.067, 0.483) | 0.133 (-.145, 0.411) | 0.096 (-.183, 0.374) | 0.108 (-.169, 0.386) | 0.106 (-.171, 0.383) |
| M_LDL_FC | 0.229 (-0.044, 0.501) | 0.224 (-0.048, 0.496) | 0.148 (-.127, 0.422) | 0.108 (-.166, 0.383) | 0.117 (-.157, 0.391) | 0.113 (-.162, 0.387) |
| S_LDL_FC | 0.253 (-0.026, 0.531) | 0.249 (-0.029, 0.526) | 0.173 (-.107, 0.454) | 0.134 (-.147, 0.415) | 0.139 (-.142, 0.421) | 0.134 (-.146, 0.415) |
| XL_HDL_FC | 0.064 (-0.178, 0.306) | 0.094 (-0.146, 0.335) | 0.077 (-.168, 0.321) | 0.045 (-.201, 0.292) | 0.047 (-.200, 0.293) | 0.043 (-.201, 0.289) |
| L_HDL_FC | -0.207 (-0.425, 0.011) | -0.171 (-0.382, 0.039) | -.188 (-.402, 0.026) | -.219 (-.435, -.0026) | -.229 (-.445, -.014) | -.229 (-.445, -.014) |
| M_HDL_FC | -0.279 (-0.552, -0.008) | -0.271 (-0.544, 0.002) | -.304 (-.58, -.027) | -.331 (-.611, -.051) | -.354 (-.633, -.074) | -.349 (-.627, -.071) |
| S_HDL_FC | -0.471 (-0.770, -0.172) | -0.466 (-0.768, -0.164) | -.511 (-.817, -.204) | -.554 (-.863, -.246) | -.576 (-.885, -.267) | -.576 (-.883, -.269) |
| Free cholesterol | 0.129 (-0.157, 0.416) | 0.132 (-0.155, 0.419) | 0.053 (-.237, 0.343) | 0.0079 (-.284, 0.299) | 0.015 (-.277, 0.306) | 0.014 (-.277, 0.306) |

**Table S32. Multivariable regression analyses of pesticide exposure in months with total lipid subclasses with standardised metabolomic biomarkers in men**

| **Pesticide exposure month, β (95% CI), Males** | | | | | | |
| --- | --- | --- | --- | --- | --- | --- |
| **Total lipids** | **Unadjusted** | **+ BMI** | **+ SEP** | **+ season of pesticide use** | **+ alcohol consumption** | **+ latitude of residence** |
| XXL_VLDL_L | 0.083 (-0.144, 0.311) | 0.121 (-0.101, 0.343) | 0.109 (-.118, 0.337) | 0.118 (-.110, 0.347) | 0.123 (-.108, 0.354) | 0.123 (-.108, 0.354) |
| XL_VLDL_L | 0.011 (-0.218, 0.240) | 0.040 (-0.185, 0.265) | 0.025 (-.205, 0.255) | 0.022 (-.209, 0.254) | 0.023 (-.211, 0.257) | 0.026 (-.208, 0.260) |
| L_VLDL_L | 0.024 (-0.197, 0.245) | 0.048 (-0.169, 0.264) | 0.073 (-.148, 0.294) | 0.068 (-.154, 0.291) | 0.069 (-.155, 0.294) | 0.069 (-.155, 0.294) |
| M_VLDL_L | 0.027 (-0.189, 0.243) | 0.057 (-0.153, 0.267) | 0.073 (-.142, 0.288) | 0.066 (-.1503, 0.283) | 0.064 (-.155, 0.283) | 0.065 (-.153, 0.284) |
| S_VLDL_L | 0.0007 (-0.201, 0.203) | 0.034 (-0.162, 0.231) | 0.038 (-.163, 0.239) | 0.029 (-.173, 0.233) | 0.027 (-.178, 0.232) | 0.028 (-.177, 0.233) |
| XS_VLDL_L | -0.023 (-0.235, 0.189) | 0.014 (-0.196, 0.223) | -.029 (-.244, 0.186) | -.045 (-.261, 0.171) | -.043 (-.261, 0.175) | -.041 (-.258, 0.175) |
| IDL_L | 0.036 (-0.186, 0.257) | 0.064 (-0.158, 0.285) | -.011 (-.238, 0.216) | -.027 (-.255, 0.200) | -.022 (-.251, 0.207) | -.019 (-.246, 0.209) |
| L_LDL_L | 0.048 (-0.175, 0.270) | 0.073 (-0.150, 0.296) | 0.001 (-.227, 0.229) | -.015 (-.244, 0.214) | -.012 (-.242, 0.219) | -.0087 (-.237, 0.219) |
| M_LDL_L | 0.041 (-0.181, 0.264) | 0.063 (-0.159, 0.286) | -.00084 (-.229, 0.227) | -.016 (-.245, 0.212) | -.015 (-.245, 0.216) | -.011 (-.239, 0.217) |
| S_LDL_L | 0.048 (-0.170, 0.266) | 0.067 (-0.152, 0.285) | 0.0067 (-.217, 0.230) | -.0075 (-.232, 0.217) | -.0034 (-.229, 0.2223) | -.00043 (-.225, 0.224) |
| XL_HDL_L | 0.106 (-0.115, 0.327) | 0.084 (-0.133, 0.302) | 0.046 (-.177, 0.269) | 0.060 (-.164, 0.285) | 0.085 (-.142, 0.312) | 0.084 (-.143, 0.309) |
| L_HDL_L | 0.212 (-0.013, 0.437) | 0.178 (-0.042, 0.398) | 0.165 (-.059, 0.389) | 0.154 (-.072, 0.379) | 0.184 (-.043, 0.411) | 0.188 (-.038, 0.414) |
| M_HDL_L | 0.064 (-0.152, 0.279) | 0.039 (-0.177, 0.256) | 0.070 (-.151, 0.292) | 0.049 (-.173, 0.272) | 0.077 (-.143, 0.298) | 0.082 (-.135, 0.299) |
| S_HDL_L | -0.007 (-0.231, 0.216) | -0.025 (-0.252, 0.201) | -.026 (-.257, 0.205) | -.048 (-.278, 0.183) | -.034 (-.264, 0.196) | -.029 (-.256, 0.198) |

**Table S33. Multivariable regression analyses of pesticide exposure in months with total lipid subclasses with standardised metabolomic biomarkers in women**

| **Pesticide exposure month, β (95% CI), Females** | | | | | | |
| --- | --- | --- | --- | --- | --- | --- |
| **Total lipids** | **Unadjusted** | **+ BMI** | **+ SEP** | **+ season of pesticide use** | **+ alcohol consumption** | **+ latitude of residence** |
| XXL_VLDL_L | 0.206 (-0.053, 0.464) | 0.186 (-0.064, 0.436) | 0.183 (-.071, 0.437) | 0.172 (-.084, 0.429) | 0.178 (-.078, 0.435) | 0.183 (-.074, 0.439) |
| XL_VLDL_L | 0.038 (-0.237, 0.313) | 0.018 (-0.249, 0.286) | 0.0085 (-.263, 0.280) | -.014 (-.288, 0.260) | -.017 (-.291, 0.257) | -.014 (-.287, 0.259) |
| L_VLDL_L | 0.119 (-0.153, 0.391) | 0.107 (-0.156, 0.370) | 0.113 (-.155, 0.380) | 0.093 (-.178, 0.363) | 0.094 (-.177, 0.365) | 0.098 (-.173, 0.368) |
| M_VLDL_L | 0.189 (-0.064, 0.443) | 0.168 (-0.076, 0.412) | 0.149 (-.098, 0.397) | 0.132 (-.118, 0.382) | 0.136 (-.114, 0.386) | 0.141 (-.108, 0.391) |
| S_VLDL_L | 0.209 (-0.061, 0.478) | 0.194 (-0.066, 0.454) | 0.155 (-.109, 0.419) | 0.118 (-.147, 0.383) | 0.123 (-.141, 0.388) | 0.122 (-.143, 0.387) |
| XS_VLDL_L | 0.258 (-0.015, 0.531) | 0.249 (-0.019, 0.518) | 0.185 (-.087, 0.458) | 0.151 (-.123, 0.425) | 0.164 (-.109, 0.437) | 0.162 (-.111, 0.435) |
| IDL_L | 0.229 (-0.045, 0.502) | 0.222 (-0.051, 0.494) | 0.147 (-.129, 0.422) | 0.109 (-.168, 0.385) | 0.122 (-.154, 0.397) | 0.120 (-.155, 0.395) |
| L_LDL_L | 0.227 (-0.051, 0.506) | 0.218 (-0.059, 0.496) | 0.143 (-.137, 0.424) | 0.105 (-.177, 0.386) | 0.116 (-.164, 0.397) | 0.114 (-.166, 0.394) |
| M_LDL_L | 0.242 (-0.042, 0.525) | 0.231 (-0.051, 0.514) | 0.161 (-.125, 0.446) | 0.122 (-.163, 0.408) | 0.132 (-.153, 0.417) | 0.129 (-.155, 0.414) |
| S_LDL_L | 0.249 (-0.029, 0.526) | 0.240 (-0.036, 0.516) | 0.175 (-.105, 0.454) | 0.136 (-.144, 0.416) | 0.144 (-.136, 0.423) | 0.141 (-.138, 0.420) |
| XL_HDL_L | -0.016 (-0.263, 0.231) | 0.021 (-0.223, 0.265) | -.00052 (-.249, 0.248) | -.039 (-.289, 0.212) | -.040 (-.290, 0.209) | -.046 (-.293, 0.201) |
| L_HDL_L | -0.269 (-0.513, -0.027) | -0.233 (-0.470, 0.004) | -.262 (-.502, -.021) | -.301 (-.544, -.058) | -.313 (-.556, -.071) | -.313 (-.556, -.070) |
| M_HDL_L | -0.308 (-0.587, -0.029) | -0.303 (-0.584, -0.022) | -.331 (-.616, -.046) | -.352 (-.640, -.064) | -.376 (-.663, -.088) | -.370 (-.656, -.085) |
| S_HDL_L | -0.247 (-0.544, 0.051) | -0.249 (-0.549, 0.051) | -.303 (-.606, 0.0013) | -.332 (-.637, -.027) | -.353 (-.659, -.047) | -.353 (-.656, -.050) |

**Table S34. Multivariable regression analyses of pesticide exposure in months with total cholesterol subclasses with standardised metabolomic biomarkers in men**

| **Pesticide exposure month, β (95% CI), Males** | | | | | | |
| --- | --- | --- | --- | --- | --- | --- |
| **Total cholesterol** | **Unadjusted** | **+ BMI** | **+ SEP** | **+ season of pesticide use** | **+ alcohol consumption** | **+ latitude of residence** |
| XXL_VLDL_C | 0.084 (-0.134, 0.302) | 0.125 (-0.089, 0.339) | 0.110 (-.109, 0.329) | 0.117 (-.104, 0.338) | 0.115 (-.109, 0.338) | 0.115 (-.108, 0.338) |
| XL_VLDL_C | 0.039 (-0.186, 0.263) | 0.079 (-0.141, 0.299) | 0.050 (-.175, 0.275) | 0.054 (-.173, 0.281) | 0.051 (-.178, 0.281) | 0.053 (-.177, 0.282) |
| L_VLDL_C | 0.006 (-0.214, 0.227) | 0.041 (-0.176, 0.257) | 0.069 (-.152, 0.290) | 0.069 (-.154, 0.291) | 0.067 (-.158, 0.292) | 0.067 (-.157, 0.292) |
| M_VLDL_C | 0.042 (-0.177, 0.262) | 0.082 (-0.130, 0.295) | 0.081 (-.137, 0.299) | 0.076 (-.145, 0.296) | 0.073 (-.149, 0.295) | 0.074 (-.148, 0.296) |
| S_VLDL_C | -0.004 (-0.207, 0.199) | 0.029 (-0.169, 0.229) | 0.0067 (-.198, 0.211) | -.002 (-.208, 0.204) | -.005 (-.213, 0.204) | -.003 (-.210, 0.204) |
| XS_VLDL_C | -0.047 (-0.270, 0.177) | -0.011 (-0.234, 0.212) | -.073 (-.301, 0.155) | -.086 (-.316, 0.143) | -.083 (-.314, 0.148) | -.082 (-.312, 0.148) |
| IDL_C | 0.035 (-0.189, 0.259) | 0.063 (-0.162, 0.289) | -.015 (-.246, 0.215) | -.031 (-.262, 0.201) | -.024 (-.257, 0.209) | -.021 (-.252, 0.210) |
| L_LDL_C | 0.043 (-0.180, 0.266) | 0.068 (-0.156, 0.293) | -.0059 (-.235, 0.224) | -.021 (-.252, 0.209) | -.019 (-.251, 0.213) | -.016 (-.246, 0.215) |
| M_LDL_C | 0.023 (-0.195, 0.241) | 0.043 (-0.176, 0.262) | -.021 (-.245, 0.203) | -.036 (-.261, 0.189) | -.036 (-.263, 0.190) | -.033 (-.258, 0.192) |
| S_LDL_C | 0.019 (-0.199, 0.237) | 0.036 (-0.183, 0.256) | -.027 (-.251, 0.198) | -.040 (-.266, 0.185) | -.039 (-.267, 0.188) | -.037 (-.263, 0.189) |
| LDL_C | 0.030 (-0.187, 0.248) | 0.053 (-0.166, 0.272) | -.016 (-.239, 0.208) | -.031 (-.255, 0.194) | -.029 (-.256, 0.197) | -.027 (-.251, 0.198) |
| XL_HDL_C | 0.116 (-0.109, 0.342) | 0.101 (-0.123, 0.325) | 0.055 (-.175, 0.285) | 0.077 (-.155, 0.309) | 0.101 (-.134, 0.335) | 0.099 (-.134, 0.332) |
| L_HDL_C | 0.218 (-0.015, 0.450) | 0.183 (-0.043, 0.409) | 0.167 (-.064, 0.398) | 0.158 (-.074, 0.391) | 0.188 (-.046, 0.422) | 0.191 (-.043, 0.424) |
| M_HDL_C | 0.053 (-0.168, 0.273) | 0.025 (-0.195, 0.246) | 0.062 (-.164, 0.287) | 0.040 (-.186, 0.267) | 0.066 (-.159, 0.291) | 0.071 (-.151, 0.293) |
| S_HDL_C | -0.052 (-0.285, 0.180) | -0.068 (-0.304, 0.168) | -.092 (-.333, 0.149) | -.113 (-.354, 0.123) | -.109 (-.352, 0.133) | -.105 (-.346, 0.135) |
| HDL_C_ | 0.113 (-0.098, 0.324) | 0.081 (-0.126, 0.288) | 0.072 (-.139, 0.284) | 0.059 (-.154, 0.272) | 0.089 (-.124, 0.301) | 0.092 (-.120, 0.304) |
| Serum cholesterol | 0.089 (-0.139, 0.319) | 0.108 (-0.124, 0.339) | 0.048 (-.189, 0.284) | 0.031 (-.207, 0.268) | 0.045 (-.194, 0.284) | 0.049 (-.188, 0.286) |
| Remnant cholesterol | 0.038 (-0.179, 0.255) | 0.077 (-0.136, 0.290) | 0.029 (-.189, 0.247) | 0.017 (-.202, 0.237) | 0.022 (-.200, 0.244) | 0.024 (-.197, 0.245) |

**Table S35. Multivariable regression analyses of pesticide exposure in months with total cholesterol subclasses with standardised metabolomic biomarkers in women**

| **Pesticide exposure month, β (95% CI), Females** | | | | | | |
| --- | --- | --- | --- | --- | --- | --- |
| **Total cholesterol** | **Unadjusted** | **+ BMI** | **+ SEP** | **+ season of pesticide use** | **+ alcohol consumption** | **+ latitude of residence** |
| XXL_VLDL_C | 0.225 (-0.049, 0.499) | 0.209 (-0.059, 0.477) | 0.195 (-.077, 0.466) | 0.178 (-.096, 0.453) | 0.191 (-.084, 0.465) | 0.195 (-.079, 0.469) |
| XL_VLDL_C | 0.042 (-0.238, 0.323) | 0.024 (-0.249, 0.298) | 0.0069 (-.268, 0.282) | -.013 (-.291, 0.266) | -.011 (-.289, 0.267) | -.0091 (-.287, 0.268) |
| L_VLDL_C | 0.076 (-0.204, 0.355) | 0.065 (-0.208, 0.337) | 0.059 (-.218, 0.336) | 0.037 (-.243, 0.317) | 0.041 (-.239, 0.321) | 0.043 (-.237, 0.323) |
| M_VLDL_C | 0.234 (-0.024, 0.493) | 0.214 (-0.036, 0.465) | 0.179 (-.075, 0.433) | 0.155 (-.102, 0.411) | 0.164 (-.092, 0.421) | 0.168 (-.088, 0.424) |
| S_VLDL_C | 0.188 (-0.083, 0.459) | 0.172 (-0.093, 0.436) | 0.119 (-.149, 0.387) | 0.082 (-.188, 0.351) | 0.093 (-.175, 0.362) | 0.091 (-.178, 0.359) |
| XS_VLDL_C | 0.239 (-0.036, 0.514) | 0.228 (-0.045, 0.501) | 0.167 (-.110, 0.443) | 0.146 (-.133, 0.425) | 0.164 (-.113, 0.441) | 0.161 (-.116, 0.438) |
| IDL_C | 0.222 (-0.057, 0.500) | 0.212 (-0.066, 0.491) | 0.140 (-.142, 0.422) | 0.108 (-.175, 0.391) | 0.123 (-.159, 0.404) | 0.122 (-.159, 0.403) |
| L_LDL_C | 0.228 (-0.053, 0.509) | 0.217 (-0.064, 0.498) | 0.145 (-.139, 0.429) | 0.111 (-.174, 0.396) | 0.124 (-.159, 0.408) | 0.123 (-.161, 0.406) |
| M_LDL_C | 0.234 (-0.048, 0.516) | 0.222 (-0.059, 0.504) | 0.158 (-.126, 0.443) | 0.126 (-.159, 0.412) | 0.138 (-.146, 0.422) | 0.137 (-.147, 0.419) |
| S_LDL_C | 0.247 (-0.030, 0.524) | 0.236 (-0.041, 0.513) | 0.178 (-.103, 0.458) | 0.146 (-.135, 0.427) | 0.157 (-.123, 0.437) | 0.155 (-.125, 0.435) |
| LDL_C | 0.231 (-0.046, 0.509) | 0.219 (-0.057, 0.497) | 0.154 (-.127, 0.434) | 0.121 (-.159, 0.402) | 0.133 (-.147, 0.413) | 0.131 (-.148, 0.411) |
| XL_HDL_C | 0.043 (-0.214, 0.301) | 0.073 (-0.184, 0.331) | 0.052 (-.209, 0.314) | 0.017 (-.248, 0.281) | 0.017 (-.247, 0.282) | 0.0089 (-.251, 0.269) |
| L_HDL_C | -0.241 (-0.468, -0.013) | -0.204 (-0.424, 0.017) | -.224 (-.448, -.00019) | -.258 (-.485, -.032) | -.269 (-.495, -.044) | -.270 (-.496, -.045) |
| M_HDL_C | -0.319 (-0.591, -0.047) | -0.315 (-0.588, -0.042) | -.332 (-.609, -.054) | -.339 (-.619, -.058) | -.362 (-.641, -.082) | -.356 (-.633, -.078) |
| S_HDL_C | -0.069 (-0.341, 0.203) | -0.074 (-0.347, 0.200) | -.133 (-.409, 0.143) | -.164 (-.441, 0.112) | -.176 (-.453, 0.102) | -.179 (-.454, 0.097) |
| HDL_C_ | -0.247 (-0.505, 0.011) | -0.216 (-0.471, 0.038) | -.252 (-.511, 0.0059) | -.297 (-.558, -.036) | -.314 (-.575, -.054) | -.31 (-.576, -.054) |
| Serum cholesterol | 0.135 (-0.151, 0.422) | 0.136 (-0.151, 0.424) | 0.061 (-.229, 0.352) | 0.014 (-.278, 0.306) | 0.018 (-.274, 0.310) | 0.017 (-.276, 0.309) |
| Remnant cholesterol | 0.243 (-0.025, 0.511) | 0.228 (-0.035, 0.492) | 0.166 (-.100, 0.433) | 0.135 (-.133, 0.403) | 0.150 (-.117, 0.417) | 0.149 (-.117, 0.417) |

**Table S36. Multivariable regression analyses of pesticide exposure in months (PEM) with apolipoproteins, aminoacids, sphingomyelins, glucose metabolism, fatty acids, inflammation, and fluid balance with standardised metabolomic biomarkers in men**

| **Pesticide exposure month, β (95% CI), Males** | | | | | | |
| --- | --- | --- | --- | --- | --- | --- |
|  | **Unadjusted** | **+ BMI** | **+ SEP** | **+ season of pesticide use** | **+ alcohol consumption** | **+ latitude of residence** |
| **Apolipoproteins** |  |  |  |  |  |  |
| Apolipoprotein A1 [g/L] | 0.139 (-0.069, 0.346) | 0.121 (-0.088, 0.329) | 0.116 (-.097, 0.329) | 0.097 (-.117, 0.310) | 0.125 (-.089, 0.338) | 0.129 (-.082, 0.340) |
| Apolipoprotein B [g/L] | 0.053 (-0.165, 0.271) | 0.089 (-0.125, 0.304) | 0.047 (-.172, 0.266) | 0.033 (-.187, 0.254) | 0.038 (-.185, 0.261) | 0.041 (-.181, 0.263) |
| Ratio of Apo B/Apo A1 | -0.041 (-0.247, 0.165) | 0.006 (-0.191, 0.203) | -.032 (-.234, 0.169) | -.032 (-.235, 0.172) | -.046 (-.250, 0.159) | -.046 (-.250, 0.158) |
| **Amino acids** |  |  |  |  |  |  |
| Alanine [µmol/L] | -0.065 (-0.289, 0.159) | -0.055 (-0.279, 0.169) | 0.033 (-.197, 0.262) | 0.0025 (-.228, 0.232) | 0.002 (-.229, 0.234) | 0.0046 (-.226, 0.235) |
| Glutamine [µmol/L] | -0.097 (-0.332, 0.137) | -0.102 (-0.338, 0.134) | -.159 (-.402, 0.082) | -.051 (-.290, 0.189) | -.050 (-.291, 0.190) | -.048 (-.287, 0.192) |
| Glycine [µmol/L] | -0.009 (-0.199, 0.183) | 0.003 (-0.190, 0.196) | 0.022 (-.175, 0.219) | -.015 (-.213, 0.183) | -.044 (-.243, 0.155) | -.043 (-.238, 0.151) |
| Histidine [µmol/L] | 0.064 (-0.183, 0.312) | 0.059 (-0.189, 0.309) | 0.079 (-.176, 0.335) | 0.137 (-.120, 0.394) | 0.135 (-.123, 0.393) | 0.133 (-.124, 0.391) |
| **Branched-chain amino acids** |  |  |  |  |  |  |
| Isoleucine [µmol/L] | -0.012 (-0.219, 0.196) | 0.013 (-0.191, 0.217) | 0.047 (-.161, 0.256) | 0.025 (-.185, 0.235) | 0.029 (-.182, 0.242) | 0.033 (-.178, 0.244) |
| Leucine [µmol/L] | 0.021 (-0.183, 0.224) | 0.016 (-0.185, 0.217) | 0.060 (-.145, 0.265) | 0.035 (-.171, 0.242) | 0.043 (-.166, 0.252) | 0.046 (-.160, 0.253) |
| Valine [µmol/L] | -0.002 (-0.199, 0.196) | -0.007 (-0.205, 0.190) | 0.034 (-.168, 0.235) | 0.017 (-.186, 0.219) | 0.012 (-.192, 0.217) | 0.016 (-.187, 0.219) |
| **Aromatic amino acids** |  |  |  |  |  |  |
| Phenylalanine [µmol/L] | 0.096 (-0.117, 0.309) | 0.101 (-0.107, 0.309) | 0.074 (-.139, 0.287) | 0.055 (-.158, 0.267) | 0.032 (-.181, 0.245) | 0.035 (-.172, 0.243) |
| Tyrosine [µmol/L] | -0.257 (-0.475, -0.039) | -.233 (-0.450, -0.016) | -.201 (-.423, 0.021) | -.121 (-.343, 0.101) | -.119 (-.344, 0.105) | -.117 (-.341, 0.107) |
| **Ketone bodies** |  |  |  |  |  |  |
| Acetoacetate [µmol/L] | 0.039 (-0.181, 0.259) | 0.006 (-0.216, 0.228) | -.029 (-.257, 0.197) | -.032 (-.261, 0.197) | -.020 (-.249, 0.209) | -.018 (-.248, 0.211) |
| Acetate [µmol/L] | 0.092 (-0.131, 0.315) | 0.112 (-0.113, 0.337) | 0.156 (-.074, 0.386) | 0.130 (-.101, 0.361) | 0.139 (-.094, 0.373) | 0.141 (-.092, 0.374) |
| beta-hydroxybutyrate [µmol/L] | 0.098 (-0.115, 0.311) | 0.057 (-0.158, 0.272) | 0.023 (-.196, 0.242) | 0.021 (-.199, 0.241) | 0.036 (-.184, 0.256) | 0.038 (-.181, 0.256) |
| **Sphingolipids** |  |  |  |  |  |  |
| Sphingomyelin | 0.113 (-0.118, 0.343) | 0.113 (-0.120, 0.347) | 0.071 (-.168, 0.309) | 0.052 (-.186, 0.291) | 0.067 (-.173, 0.308) | 0.073 (-.165, 0.311) |
| Total choline | 0.106 (-0.108, 0.319) | 0.110 (-0.106, 0.327) | 0.082 (-.139, 0.304) | 0.068 (-.154, 0.291) | 0.093 (-.129, 0.316) | 0.098 (-.123, 0.319) |
| **Glycolysis related metabolites** |  |  |  |  |  |  |
| Citrate [µmol/L] | 0.037 (-0.194, 0.267) | 0.019 (-0.214, 0.252) | 0.016 (-.223, 0.254) | 0.0033 (-.235, 0.242) | -.002 (-.242, 0.238) | 0.0014 (-.237, 0.239) |
| Glucose [mmol/L] | 0.027 (-0.197, 0.252) | 0.033 (-0.195, 0.259) | 0.0084 (-.224, 0.240) | -.019 (-.251, 0.212) | 0.0054 (-.228, 0.238) | 0.0093 (-.222, 0.241) |
| Glycerol [mmol/L] | -0.210 (-0.444, 0.023) | -0.205 (-0.441, 0.032) | -.237 (-.478, 0.0048) | -.207 (-.449, 0.036) | -.224 (-.469, 0.021) | -.224 (-.468, 0.020) |
| Lactate [mmol/L] | -0.279 (-0.502, -0.056) | -0.276 (-0.501, -0.050) | -.202 (-.432, 0.028) | -.222 (-.453, 0.010) | -.222 (-.455, 0.011) | -.217 (-.446, 0.012) |
| Pyruvate [mmol/L] | 0.077 (-0.151, 0.304) | 0.087 (-0.143, 0.318) | 0.028 (-.206, 0.263) | 0.049 (-.186, 0.285) | 0.047 (-.191, 0.285) | 0.045 (-.193, 0.282) |
| **Fatty acids** |  |  |  |  |  |  |
| Total fatty acids [mmol/L] | 0.071 (-0.152, 0.294) | 0.085 (-0.137, 0.308) | 0.076 (-.151, 0.304) | 0.063 (-.166, 0.292) | 0.082 (-.148, 0.312) | 0.086 (-.143, 0.315) |
| Monounsaturated fatty acids [mmol/L] | 0.032 (-0.188, 0.251) | 0.044 (-0.174, 0.261) | 0.054 (-.168, 0.277) | 0.041 (-.183, 0.265) | 0.059 (-.165, 0.283) | 0.062 (-.161, 0.285) |
| Saturated fatty acids [mmol/L] | 0.112 (-0.109, 0.332) | 0.126 (-0.094, 0.346) | 0.080 (-.145, 0.305) | 0.067 (-.159, 0.293) | 0.079 (-.148, 0.306) | 0.084 (-.142, 0.309) |
| **Inflammation** |  |  |  |  |  |  |
| Alpha-1-acid glycoprotein [mmol/L] | -0.024 (-0.254, 0.206) | -0.025 (-0.253, 0.203) | -.0025 (-.234, 0.231) | -.037 (-.270, 0.197) | -.046 (-.282, 0.190) | -.042 (-.276, 0.192) |
| **Fluid balance** |  |  |  |  |  |  |
| Creatinine [µmol/L] | -0.240 (-0.537, 0.056) | -0.246 (-0.545, 0.053) | -.187 (-.493, 0.119) | -.113 (-.419, 0.194) | -.117 (-.427, 0.194) | -.119 (-.429, 0.192) |
| Albumin [standardized concentration units] | 0.070 (-0.179, 0.319) | 0.068 (-0.185, 0.320) | 0.052 (-.207, 0.311) | 0.024 (-.236, 0.284) | 0.028 (-.234, 0.291) | 0.033 (-.226, 0.293) |

**Table S37. Multivariable regression analyses of pesticide exposure in months (PEM) with apolipoproteins, aminoacids, sphingomyelins, glucose metabolism, fatty acids, inflammation, and fluid balance with standardised metabolomic biomarkers in women**

| **Pesticide exposure month, β (95% CI), Females** | | | | | | |
| --- | --- | --- | --- | --- | --- | --- |
|  | **Unadjusted** | **+ BMI** | **+ SEP** | **+ season of pesticide use** | **+ alcohol consumption** | **+ latitude of residence** |
| **Apolipoproteins** |  |  |  |  |  |  |
| Apolipoprotein A1 [g/L] | -0.229 (-0.513, 0.056) | -0.204 (-0.489, 0.080) | -.256 (-.545, 0.033) | -.308 (-.599, -.017) | -.324 (-.615, -.032) | -.323 (-.614, -.031) |
| Apolipoprotein B [g/L] | 0.259 (-0.007, 0.524) | 0.245 (-0.015, 0.505) | 0.183 (-.081, 0.446) | 0.149 (-.115, 0.414) | 0.162 (-.102, 0.425) | 0.162 (-.102, 0.426) |
| Ratio of Apo B/Apo A1 | 0.399 (0.143, 0.654) | 0.368 (0.123, 0.614) | 0.344 (0.095, 0.593) | 0.346 (0.094, 0.597) | 0.368 (0.119, 0.617) | 0.367 (0.118, 0.616) |
| **Amino acids** |  |  |  |  |  |  |
| Alanine [µmol/L] | 0.110 (-0.165, 0.386) | 0.130 (-0.145, 0.405) | 0.156 (-.123, 0.436) | 0.125 (-.156, 0.406) | 0.116 (-.167, 0.398) | 0.112 (-.169, 0.394) |
| Glutamine [µmol/L] | -0.198 (-0.466, 0.069) | -0.175 (-0.445, 0.096) | -.196 (-.471, 0.079) | -.189 (-.465, 0.088) | -.179 (-.456, 0.099) | -.174 (-.447, 0.099) |
| Glycine [µmol/L] | -0.205 (-0.525, 0.115) | -0.207 (-0.529, 0.116) | -.276 (-.602, 0.051) | -.269 (-.597, 0.059) | -.261 (-.591, 0.068) | -.267 (-.594, 0.059) |
| Histidine [µmol/L] | 0.005 (-0.269, 0.279) | 0.009 (-0.266, 0.286) | 0.049 (-.229, 0.328) | 0.042 (-.241, 0.324) | 0.037 (-.247, 0.320) | 0.040 (-.243, 0.323) |
| **Branched-chain amino acids** |  |  |  |  |  |  |
| Isoleucine [µmol/L] | 0.077 (-0.172, 0.326) | 0.067 (-0.176, 0.309) | 0.064 (-.183, 0.311) | 0.081 (-.168, 0.331) | 0.082 (-.168, 0.332) | 0.084 (-.165, 0.333) |
| Leucine [µmol/L] | 0.013 (-0.238, 0.263) | -0.009 (-0.255, 0.237) | -.0088 (-.258, 0.241) | -.0091 (-.260, 0.242) | -.017 (-.268, 0.235) | -.020 (-.270, 0.230) |
| Valine [µmol/L] | -0.089 (-0.351, 0.173) | -0.108 (-0.367, 0.151) | -.107 (-.369, 0.156) | -.089 (-.354, 0.174) | -.094 (-.359, 0.172) | -.094 (-.357, 0.168) |
| **Aromatic amino acids** |  |  |  |  |  |  |
| Phenylalanine [µmol/L] | 0.311 (0.031, 0.592) | 0.292 (0.017, 0.567) | 0.249 (-.029, 0.528) | 0.220 (-.058, 0.499) | 0.216 (-.062, 0.495) | 0.206 (-.068, 0.481) |
| Tyrosine [µmol/L] | 0.172 (-0.096, 0.441) | 0.152 (-0.116, 0.419) | 0.165 (-.106, 0.437) | 0.169 (-.106, 0.443) | 0.154 (-.120, 0.429) | 0.159 (-.112, 0.431) |
| **Ketone bodies** |  |  |  |  |  |  |
| Acetoacetate [µmol/L] | -0.066 (-0.352, 0.219) | -0.056 (-0.345, 0.233) | -.100 (-.394, 0.194) | -.123 (-.420, 0.174) | -.139 (-.436, 0.156) | -.139 (-.434, 0.156) |
| Acetate [µmol/L] | -0.018 (-0.291, 0.254) | -.023 (-0.298, 0.253) | -.022 (-.299, 0.256) | -.035 (-.315, 0.245) | -.037 (-.318, 0.243) | -.039 (-.318, 0.241) |
| beta-hydroxybutyrate [µmol/L] | -0.007 (-0.310, 0.296) | 0.027 (-0.279, 0.333) | -.031 (-.339, 0.278) | -.065 (-.377, 0.246) | -.074 (-.385, 0.236) | -.081 (-.390, 0.229) |
| **Sphingolipids** |  |  |  |  |  |  |
| Sphingomyelin | 0.006 (-0.284, 0.296) | 0.014 (-0.277, 0.306) | -.066 (-.361, 0.229) | -.123 (-.419, 0.173) | -.123 (-.421, 0.174) | -.123 (-.420, 0.174) |
| Total choline | -0.026 (-0.314, 0.262) | -0.015 (-0.305, 0.275) | -.085 (-.378, 0.209) | -.140 (-.436, 0.155) | -.149 (-.446, 0.148) | -.148 (-.445, 0.148) |
| **Glycolysis related metabolites** |  |  |  |  |  |  |
| Citrate [µmol/L] | 0.298 (0.023, 0.573) | 0.334 (0.056, 0.612) | 0.314 (0.032, 0.596) | 0.278 (-.0068, 0.563) | 0.297 (0.013, 0.581) | 0.298 (0.016, 0.579) |
| Glucose [mmol/L] | -0.210 (-0.499, 0.079) | -0.209 (-0.501, 0.083) | -.254 (-.549, 0.041) | -.242 (-.536, 0.053) | -.246 (-.542, 0.051) | -.241 (-.535, 0.053) |
| Glycerol [mmol/L] | 0.168 (-0.114, 0.449) | 0.164 (-0.119, 0.448) | 0.168 (-.120, 0.457) | 0.169 (-.122, 0.461) | 0.173 (-.119, 0.466) | 0.183 (-.108, 0.474) |
| Lactate [mmol/L] | 0.119 (-0.159, 0.396) | 0.109 (-0.168, 0.388) | 0.149 (-.133, 0.432) | 0.141 (-.144, 0.427) | 0.132 (-.154, 0.418) | 0.123 (-.156, 0.403) |
| Pyruvate [mmol/L] | 0.074 (-0.203, 0.349) | 0.073 (-0.205, 0.350) | 0.065 (-.218, 0.347) | 0.057 (-.227, 0.342) | 0.066 (-.219, 0.352) | 0.070 (-.215, 0.355) |
| **Fatty acids** |  |  |  |  |  |  |
| Total fatty acids [mmol/L] | 0.089 (-0.195, 0.372) | 0.092 (-0.190, 0.375) | 0.029 (-.257, 0.316) | -.023 (-.311, 0.265) | -.029 (-.318, 0.259) | -.029 (-.318, 0.260) |
| Monounsaturated fatty acids [mmol/L] | 0.157 (-0.116, 0.429) | 0.154 (-0.115, 0.424) | 0.101 (-.173, 0.374) | 0.049 (-.226, 0.324) | 0.035 (-.239, 0.311) | 0.036 (-.239, 0.311) |
| Saturated fatty acids [mmol/L] | 0.088 (-0.199, 0.374) | 0.095 (-0.191, 0.382) | 0.021 (-.269, 0.311) | -.032 (-.323, 0.260) | -.035 (-.327, 0.258) | -.034 (-.327, 0.258) |
| **Inflammation** |  |  |  |  |  |  |
| Alpha-1-acid glycoprotein [mmol/L] | -0.057 (-0.331, 0.217) | -0.069 (-0.338, 0.199) | -.107 (-.379, 0.165) | -.139 (-.412, 0.132) | -.140 (-.413, 0.132) | -.140 (-.411, 0.131) |
| **Fluid balance** |  |  |  |  |  |  |
| Creatinine [µmol/L] | -0.033 (-0.214, 0.147) | -0.029 (-0.211, 0.154) | -.015 (-.200, 0.171) | -.023 (-.209, 0.164) | -.025 (-.212, 0.163) | -.023 (-.210, 0.164) |
| Albumin [standardized concentration units] | -0.224 (-0.481, 0.033) | -0.215 (-0.474, 0.044) | -.255 (-.517, 0.0082) | -.251 (-.512, 0.011) | -.256 (-.519, 0.0068) | -.258 (-.518, 0.0021) |

**Pesticide exposure in years**

**Table S38. Multivariable regression analyses of pesticide exposure in years with total lipoprotein subclasses with standardised metabolomic biomarkers in men**

| **Pesticide exposure years, β (95% CI), Males** | | | | | | |
| --- | --- | --- | --- | --- | --- | --- |
| **Total lipoprotein** | **Unadjusted** | **+ BMI** | **+ SEP** | **+ season of pesticide use** | **+ alcohol consumption** | **+ latitude of residence** |
| XXL_VLDL_P | 0.037 (-0.167, 0.240) | 0.025 (-0.171, 0.221) | -.003 (-.211, 0.204) | 0.001 (-.207, 0.209) | -.003 (-.214, 0.207) | -.005 (-.216, 0.206) |
| XL_VLDL_P | -0.025 (-0.226, 0.176) | -0.034 (-0.229, 0.161) | -.051 (-.257, 0.154) | -.049 (-.255, 0.157) | -.044 (-.253, 0.165) | -.045 (-.254, 0.164) |
| L_VLDL_P | -0.103 (-0.298, 0.092) | -0.107 (-0.296, 0.081) | -.092 (-.292, 0.108) | -.096 (-.296, 0.104) | -.082 (-.285, 0.119) | -.079 (-.282, 0.124) |
| M_VLDL_P | -0.039 (-0.231, 0.153) | -0.049 (-0.233, 0.136) | -.036 (-.231, 0.159) | -.037 (-.233, 0.159) | -.032 (-.230, 0.166) | -.034 (-.233, 0.165) |
| S_VLDL_P | -0.039 (-0.219, 0.141) | -0.049 (-0.222, 0.124) | -.047 (-.231, 0.136) | -.053 (-.237, 0.131) | -.052 (-.239, 0.134) | -.069 (-.256, 0.117) |
| XS_VLDL_P | 0.0004 (-0.191, 0.191) | -0.009 (-0.194, 0.177) | -.057 (-.254, 0.139) | -.067 (-.264, 0.129) | -.071 (-.271, 0.129) | -.104 (-.302, 0.095) |
| IDL_P | -0.052 (-0.240, 0.137) | -0.053 (-0.241, 0.136) | -.032 (-.233, 0.168) | -.022 (-.223, 0.179) | -.049 (-.253, 0.155) | -.038 (-.242, 0.167) |
| L_LDL_P | 0.038 (-0.160, 0.236) | 0.031 (-0.165, 0.228) | -.058 (-.266, 0.149) | -.074 (-.282, 0.133) | -.068 (-.278, 0.142) | -.103 (-.311, 0.106) |
| M_LDL_P | 0.018 (-0.178, 0.214) | 0.011 (-0.183, 0.204) | -.071 (-.276, 0.134) | -.088 (-.293, 0.116) | -.078 (-.285, 0.129) | -.112 (-.317, 0.093) |
| S_LDL_P | -0.001 (-0.198, 0.195) | -0.008 (-0.203, 0.186) | -.091 (-.297, 0.115) | -.108 (-.313, 0.097) | -.095 (-.303, 0.112) | -.128 (-.335, 0.078) |
| XL_HDL_P | 0.019 (-0.175, 0.215) | 0.030 (-0.159, 0.220) | -.022 (-.223, 0.179) | -.019 (-.221, 0.182) | -.030 (-.235, 0.174) | -.040 (-.245, 0.164) |
| L_HDL_P | -0.061 (-0.256, 0.134) | -0.045 (-0.233, 0.143) | -.101 (-.302, 0.099) | -.111 (-.311, 0.089) | -.106 (-.307, 0.096) | -.114 (-.315, 0.087) |
| M_HDL_P | -0.124 (-0.317, 0.069) | -0.119 (-0.311, 0.073) | -.129 (-.332, 0.074) | -.143 (-.346, 0.059) | -.119 (-.321, 0.083) | -.117 (-.316, 0.083) |
| S_HDL_P | -0.055 (-0.250, 0.141) | -0.056 (-0.252, 0.140) | -.107 (-.314, 0.099) | -.128 (-.334, 0.077) | -.098 (-.304, 0.107) | -.118 (-.321, 0.085) |

**Table S39. Multivariable regression analyses of pesticide exposure in years with total lipoprotein subclasses with standardised metabolomic biomarkers in women**

| **Pesticide exposure years, β (95% CI), Females** | | | | | | |
| --- | --- | --- | --- | --- | --- | --- |
| **Total lipoprotein** | **Unadjusted** | **+ BMI** | **+ SEP** | **+ season of pesticide use** | **+ alcohol consumption** | **+ latitude of residence** |
| XXL_VLDL_P | 0.119 (-0.246, 0.485) | 0.094 (-0.255, 0.444) | 0.068 (-.284, 0.419) | 0.075 (-.277, 0.427) | 0.097 (-.263, 0.456) | 0.109 (-.249, 0.469) |
| XL_VLDL_P | 0.124 (-0.278, 0.525) | 0.106 (-0.280, 0.493) | 0.061 (-.328, 0.451) | 0.056 (-.334, 0.445) | 0.094 (-.305, 0.493) | 0.126 (-.272, 0.524) |
| L_VLDL_P | -0.147 (-0.514, 0.220) | -0.149 (-0.499, 0.203) | -.164 (-.518, 0.189) | -.168 (-.521, 0.186) | -.051 (-.420, 0.319) | -.024 (-.393, 0.345) |
| M_VLDL_P | 0.086 (-0.266, 0.438) | 0.072 (-0.264, 0.408) | 0.041 (-.297, 0.379) | 0.036 (-.301, 0.373) | 0.138 (-.213, 0.489) | 0.164 (-.186, 0.515) |
| S_VLDL_P | 0.241 (-0.133, 0.615) | 0.227 (-0.129, 0.584) | 0.181 (-.178, 0.540) | 0.170 (-.187, 0.528) | 0.267 (-.105, 0.638) | 0.264 (-.108, 0.635) |
| XS_VLDL_P | 0.357 (-0.030, 0.744) | 0.348 (-0.028, 0.725) | 0.274 (-.104, 0.652) | 0.260 (-.116, 0.636) | 0.333 (-.057, 0.724) | 0.322 (-.068, 0.713) |
| IDL_P | -0.066 (-0.509, 0.376) | -0.056 (-0.498, 0.385) | -.059 (-.505, 0.386) | -.050 (-.496, 0.396) | -.097 (-.560, 0.367) | -.093 (-.557, 0.370) |
| L_LDL_P | 0.339 (-0.049, 0.728) | 0.337 (-0.047, 0.720) | 0.243 (-.141, 0.627) | 0.220 (-.160, 0.601) | 0.316 (-.079, 0.711) | 0.307 (-.088, 0.702) |
| M_LDL_P | 0.341 (-0.047, 0.730) | 0.338 (-0.045, 0.722) | 0.250 (-.133, 0.634) | 0.226 (-.154, 0.606) | 0.350 (-.044, 0.745) | 0.340 (-.054, 0.735) |
| S_LDL_P | 0.341 (-0.050, 0.731) | 0.337 (-0.047, 0.723) | 0.254 (-.132, 0.639) | 0.231 (-.152, 0.613) | 0.362 (-.0355, 0.759) | 0.352 (-.046, 0.749) |
| XL_HDL_P | 0.134 (-0.207, 0.475) | 0.149 (-0.184, 0.483) | 0.133 (-.203, 0.468) | 0.142 (-.194, 0.477) | 0.067 (-.282, 0.416) | 0.038 (-.307, 0.383) |
| L_HDL_P | 0.016 (-0.325, 0.357) | 0.035 (-0.295, 0.364) | 0.016 (-.315, 0.348) | 0.014 (-.318, 0.346) | -.062 (-.406, 0.283) | -.060 (-.406, 0.285) |
| M_HDL_P | -0.079 (-0.473, 0.314) | -0.072 (-0.464, 0.321) | -.088 (-.483, 0.307) | -.106 (-.501, 0.289) | -.129 (-.539, 0.282) | -.099 (-.507, 0.309) |
| S_HDL_P | -0.054 (-0.464, 0.356) | -0.054 (-0.464, 0.356) | -.108 (-.519, 0.303) | -.137 (-.546, 0.272) | -.110 (-.537, 0.316) | -.108 (-.531, 0.315) |

**Table S40. Multivariable regression analyses of pesticide exposure in years with triglyceride subclasses with standardised metabolomic biomarkers in men**

| **Pesticide exposure years, β (95% CI), Males** | | | | | | |
| --- | --- | --- | --- | --- | --- | --- |
| **Triglycerides** | **Unadjusted** | **+ BMI** | **+ SEP** | **+ season of pesticide use** | **+ alcohol consumption** | **+ latitude of residence** |
| XXL_VLDL_TG | 0.034 (-0.167, 0.234) | 0.021 (-0.172, 0.215) | -.009 (-.214, 0.195) | -.005 (-.209, 0.199) | -.008 (-.216, 0.199) | -.009 (-.217, 0.198) |
| XL_VLDL_TG | -0.029 (-0.224, 0.165) | -0.037 (-.226, 0.152) | -.047 (-.247, 0.152) | -.047 (-.246, 0.153) | -.036 (-.239, 0.166) | -.034 (-.236, 0.169) |
| L_VLDL_TG | -0.141 (-0.333, 0.050) | -0.145 (-0.330, 0.040) | -.125 (-.321, 0.072) | -.129 (-.325, 0.068) | -.111 (-.309, 0.088) | -.105 (-.304, 0.094) |
| M_VLDL_TG | -0.062 (-0.246, 0.123) | -0.070 (-0.248, 0.107) | -.050 (-.238, 0.138) | -.050 (-.239, 0.137) | -.043 (-.233, 0.148) | -.040 (-.231, 0.151) |
| S_VLDL_TG | -0.064 (-0.239, 0.112) | -0.073 (-0.241, 0.096) | -.057 (-.235, 0.122) | -.059 (-.239, 0.119) | -.056 (-.237, 0.126) | -.065 (-.247, 0.117) |
| XS_VLDL_TG | -0.055 (-0.234, 0.125) | -0.064 (-0.236, 0.109) | -.076 (-.259, 0.107) | -.086 (-.269, 0.097) | -.081 (-.266, 0.105) | -.107 (-.292, 0.077) |
| VLDL_TG | -0.053 (-0.242, 0.137) | -0.062 (-0.244, 0.119) | -.051 (-.244, 0.141) | -.053 (-.246, 0.140) | -.046 (-.241, 0.149) | -.048 (-.244, 0.148) |
| IDL_TG | -0.015 (-0.201, 0.169) | -0.024 (-0.204, 0.156) | -.072 (-.263, 0.118) | -.088 (-.278, 0.102) | -.083 (-.275, 0.109) | -.124 (-.314, 0.067) |
| L_LDL_TG | 0.037 (-0.151, 0.226) | 0.029 (-0.154, 0.213) | -.041 (-.236, 0.153) | -.059 (-.253, 0.134) | -.054 (-.251, 0.142) | -.101 (-.295, 0.092) |
| M_LDL_TG | 0.055 (-0.134, 0.244) | 0.047 (-0.138, 0.233) | -.022 (-.218, 0.175) | -.041 (-.236, 0.154) | -.033 (-.231, 0.164) | -.079 (-.274, 0.116) |
| S_LDL_TG | 0.022 (-0.166, 0.209) | 0.013 (-0.169, 0.195) | -.039 (-.232, 0.154) | -.054 (-.247, 0.138) | -.045 (-.239, 0.150) | -.081 (-.274, 0.113) |
| LDL_TG | 0.042 (-0.146, 0.231) | 0.034 (-0.150, 0.218) | -.033 (-.228, 0.162) | -.052 (-.246, 0.142) | -.044 (-.241, 0.152) | -.089 (-.283, 0.105) |
| XL_HDL_TG | 0.161 (-0.037, 0.358) | 0.155 (-0.042, 0.352) | 0.083 (-.125, 0.291) | 0.081 (-.128, 0.289) | 0.075 (-.136, 0.286) | 0.041 (-.169, 0.250) |
| L_HDL_TG | 0.105 (-0.094, 0.304) | 0.108 (-0.091, 0.307) | 0.055 (-.157, 0.267) | 0.037 (-.174, 0.248) | 0.035 (-.178, 0.249) | 0.00019 (-.211, 0.212) |
| M_HDL_TG | 0.048 (-0.146, 0.242) | 0.042 (-0.148, 0.231) | 0.041 (-.159, 0.242) | 0.036 (-.165, 0.238) | 0.035 (-.169, 0.239) | 0.019 (-.184, 0.223) |
| S_HDL_TG | 0.088 (-0.088, 0.264) | 0.079 (-0.089, 0.247) | 0.034 (-.144, 0.212) | 0.026 (-.152, 0.204) | 0.029 (-.151, 0.209) | -.001 (-.180, 0.178) |
| HDL_TG | 0.087 (-0.105, 0.279) | 0.081 (-0.108, 0.270) | 0.034 (-.166, 0.235) | 0.022 (-.178, 0.222) | 0.022 (-.180, 0.225) | -.011 (-.212, 0.190) |
| Serum_TG | -0.038 (-0.235, 0.159) | -0.048 (-0.237, 0.140) | -.054 (-.254, 0.146) | -.061 (-.261, 0.139) | -.053 (-.256, 0.150) | -.067 (-.269, 0.137) |

**Table S41. Multivariable regression analyses of pesticide exposure in years with triglyceride subclasses with standardised metabolomic biomarkers in women**

| **Pesticide exposure years, β (95% CI), Females** | | | | | | |
| --- | --- | --- | --- | --- | --- | --- |
| **Triglycerides** | **Unadjusted** | **+ BMI** | **+ SEP** | **+ season of pesticide use** | **+ alcohol consumption** | **+ latitude of residence** |
| XXL_VLDL_TG | 0.103 (-0.261, 0.467) | 0.078 (-0.269, 0.426) | 0.054 (-.297, 0.404) | 0.062 (-.289, 0.412) | 0.088 (-.270, 0.446) | 0.100 (-.258, 0.458) |
| XL_VLDL_TG | 0.124 (-0.286, 0.534) | 0.107 (-0.290, 0.505) | 0.072 (-.328, 0.472) | 0.064 (-.336, 0.463) | 0.099 (-.309, 0.508) | 0.133 (-.275, 0.541) |
| L_VLDL_TG | -0.184 (-0.554, 0.186) | -0.186 (-0.542, 0.169) | -.197 (-.556, 0.161) | -.203 (-.561, 0.155) | -.088 (-.463, 0.287) | -.055 (-.429, 0.319) |
| M_VLDL_TG | 0.031 (-0.336, 0.398) | 0.017 (-0.335, 0.369) | -.0023 (-.356, 0.352) | -.0048 (-.358, 0.348) | 0.118 (-.250, 0.486) | 0.149 (-.218, 0.517) |
| S_VLDL_TG | 0.228 (-0.169, 0.625) | 0.214 (-0.168, 0.596) | 0.185 (-.199, 0.569) | 0.182 (-.201, 0.565) | 0.272 (-.127, 0.671) | 0.273 (-.126, 0.672) |
| XS_VLDL_TG | 0.377 (-0.027, 0.781) | 0.365 (-0.025, 0.754) | 0.314 (-.078, 0.706) | 0.305 (-.085, 0.695) | 0.348 (-.058, 0.754) | 0.335 (-.071, 0.740) |
| VLDL_TG | 0.139 (-0.229, 0.507) | 0.124 (-0.227, 0.475) | 0.099 (-.254, 0.452) | 0.097 (-.256, 0.449) | 0.183 (-.184, 0.549) | 0.205 (-.161, 0.572) |
| IDL_TG | 0.499 (0.092, 0.905) | 0.492 (0.095, 0.889) | 0.426 (0.027, 0.825) | 0.415 (0.019, 0.812) | 0.415 (0.002, 0.827) | 0.388 (-.024, 0.800) |
| L_LDL_TG | 0.514 (0.102, 0.926) | 0.510 (0.105, 0.915) | 0.437 (0.031, 0.842) | 0.426 (0.022, 0.829) | 0.411 (-.0091, 0.831) | 0.379 (-.040, 0.799) |
| M_LDL_TG | 0.515 (0.110, 0.919) | 0.511 (0.112, 0.909) | 0.454 (0.054, 0.855) | 0.444 (0.046, 0.842) | 0.439 (0.025, 0.854) | 0.407 (-.0066, 0.821) |
| S_LDL_TG | 0.469 (0.065, 0.875) | 0.463 (0.066, 0.859) | 0.412 (0.014, 0.810) | 0.401 (0.005, 0.798) | 0.418 (0.0057, 0.829) | 0.393 (-.019, 0.805) |
| LDL_TG | 0.515 (0.106, 0.923) | 0.510 (0.109, 0.911) | 0.442 (0.039, 0.844) | 0.431 (0.031, 0.831) | 0.424 (0.0078, 0.839) | 0.393 (-.023, 0.808) |
| XL_HDL_TG | 0.197 (-0.199, 0.593) | 0.205 (-0.190, 0.599) | 0.128 (-.269, 0.524) | 0.128 (-.268, 0.524) | 0.026 (-.386, 0.438) | 0.019 (-.392, 0.432) |
| L_HDL_TG | 0.092 (-0.295, 0.479) | 0.103 (-0.282, 0.488) | 0.027 (-.360, 0.413) | 0.0097 (-.375, 0.395) | -.093 (-.495, 0.309) | -.111 (-.512, 0.290) |
| M_HDL_TG | 0.252 (-0.137, 0.641) | 0.244 (-0.139, 0.628) | 0.193 (-.193, 0.578) | 0.180 (-.204, 0.565) | 0.147 (-.255, 0.549) | 0.154 (-.248, 0.556) |
| S_HDL_TG | 0.417 (-0.00041, 0.834) | 0.406 (-0.00043, 0.813) | 0.346 (-.063, 0.754) | 0.338 (-.069, 0.746) | 0.319 (-.107, 0.744) | 0.282 (-.142, 0.707) |
| HDL_TG | 0.279 (-0.130, 0.688) | 0.278 (-0.128, 0.685) | 0.197 (-.211, 0.604) | 0.181 (-.224, 0.586) | 0.098 (-.326, 0.521) | 0.083 (-.340, 0.506) |
| Serum_TG | 0.271 (-0.094, 0.635) | 0.258 (-0.091, 0.607) | 0.214 (-.136, 0.566) | 0.207 (-.142, 0.557) | 0.241 (-.123, 0.605) | 0.249 (-.116, 0.613) |

**Table S42. Multivariable regression analyses of pesticide exposure in years with phospholipid subclasses with standardised metabolomic biomarkers in men**

| **Pesticide exposure years, β (95% CI), Males** | | | | | | |
| --- | --- | --- | --- | --- | --- | --- |
| **Phospholipids** | **Unadjusted** | **+ BMI** | **+ SEP** | **+ season of pesticide use** | **+ alcohol consumption** | **+ latitude of residence** |
| XXL_VLDL_PL | -0.009 (-0.203, 0.185) | -0.019 (-0.208, 0.169) | -.039 (-.239, 0.160) | -.037 (-.237, 0.163) | -.036 (-.239, 0.166) | -.037 (-.241, 0.166) |
| XL_VLDL_PL | 0.006 (-0.193, 0.204) | -0.003 (-0.196, 0.190) | -.025 (-.229, 0.179) | -.022 (-.227, 0.182) | -.021 (-.229, 0.187) | -.027 (-.235, 0.180) |
| L_VLDL_PL | -0.072 (-0.266, 0.121) | -0.077 (-0.264, 0.110) | -.066 (-.265, 0.132) | -.071 (-.269, 0.127) | -.059 (-.259, 0.142) | -.058 (-.259, 0.143) |
| M_VLDL_PL | -0.028 (-0.223, 0.167) | -0.038 (-0.225, 0.149) | -.032 (-.231, 0.166) | -.035 (-.234, 0.164) | -.031 (-.233, 0.171) | -.037 (-.239, 0.165) |
| S_VLDL_PL | -0.049 (-0.233, 0.133) | -0.059 (-0.235, 0.117) | -.056 (-.243, 0.130) | -.063 (-.250, 0.124) | -.062 (-.251, 0.128) | -.079 (-.269, 0.109) |
| XS_VLDL_PL | -0.007 (-0.205, 0.189) | -0.015 (-0.209, 0.179) | -.071 (-.277, 0.135) | -.085 (-.290, 0.121) | -.084 (-.292, 0.124) | -.116 (-.323, 0.091) |
| IDL_PL | 0.056 (-0.149, 0.261) | 0.050 (-0.153, 0.254) | -.041 (-.256, 0.175) | -.056 (-.271, 0.159) | -.054 (-.271, 0.164) | -.088 (-.304, 0.128) |
| L_LDL_PL | 0.037 (-0.164, 0.238) | 0.031 (-0.168, 0.229) | -.061 (-.272, 0.149) | -.077 (-.287, 0.133) | -.070 (-.283, 0.142) | -.103 (-.314, 0.109) |
| M_LDL_PL | 0.022 (-0.181, 0.225) | 0.014 (-0.185, 0.214) | -.082 (-.294, 0.129) | -.100 (-.311, 0.110) | -.089 (-.303, 0.124) | -.125 (-.336, 0.087) |
| S_LDL_PL | 0.006 (-0.193, 0.205) | -0.002 (-0.198, 0.195) | -.101 (-.308, 0.107) | -.117 (-.324, 0.090) | -.102 (-.312, 0.107) | -.136 (-.344, 0.072) |
| XL_HDL_PL | 0.062 (-0.151, 0.275) | 0.074 (-0.129, 0.277) | 0.069 (-.147, 0.284) | 0.071 (-.145, 0.287) | 0.061 (-.156, 0.279) | 0.058 (-.159, 0.276) |
| L_HDL_PL | -0.085 (-0.282, 0.113) | -0.069 (-0.260, 0.122) | -.118 (-.321, 0.086) | -.128 (-.331, 0.075) | -.120 (-.325, 0.085) | -.125 (-.329, 0.079) |
| M_HDL_PL | -0.117 (-0.310, 0.075) | -0.113 (-0.305, 0.079) | -.132 (-.336, 0.071) | -.147 (-.349, 0.056) | -.121 (-.323, 0.080) | -.120 (-.319, 0.079) |
| S_HDL_PL | -0.092 (-0.279, 0.095) | -0.089 (-0.277, 0.098) | -.114 (-.312, 0.083) | -.127 (-.325, 0.070) | -.102 (-.298, 0.094) | -.104 (-.298, 0.090) |

**Table S43. Multivariable regression analyses of pesticide exposure in years with phospholipid subclasses with standardised metabolomic biomarkers in women**

| **Pesticide exposure years, β (95% CI), Females** | | | | | | |
| --- | --- | --- | --- | --- | --- | --- |
| **Phospholipids** | **Unadjusted** | **+ BMI** | **+ SEP** | **+ season of pesticide use** | **+ alcohol consumption** | **+ latitude of residence** |
| XXL_VLDL_PL | 0.131 (-0.245, 0.507) | 0.110 (-0.254, 0.474) | 0.103 (-.265, 0.470) | 0.107 (-.261, 0.475) | 0.114 (-.262, 0.490) | 0.141 (-.236, 0.517) |
| XL_VLDL_PL | 0.099 (-0.314, 0.513) | 0.083 (-0.317, 0.484) | 0.027 (-.375, 0.429) | 0.020 (-.382, 0.422) | 0.039 (-.374, 0.453) | 0.066 (-.347, 0.479) |
| L_VLDL_PL | -0.082 (-0.451, 0.286) | -0.084 (-0.437, 0.269) | -.105 (-.461, 0.250) | -.111 (-.467, 0.244) | -.011 (-.382, 0.360) | 0.012 (-.359, 0.383) |
| M_VLDL_PL | 0.132 (-0.226, 0.489) | 0.118 (-0.223, 0.459) | 0.077 (-.266, 0.421) | 0.070 (-.272, 0.412) | 0.161 (-.195, 0.518) | 0.183 (-.173, 0.539) |
| S_VLDL_PL | 0.309 (-0.084, 0.703) | 0.296 (-0.082, 0.674) | 0.254 (-.127, 0.634) | 0.243 (-.135, 0.621) | 0.323 (-.071, 0.716) | 0.315 (-.079, 0.709) |
| XS_VLDL_PL | 0.366 (-0.025, 0.756) | 0.360 (-0.023, 0.744) | 0.282 (-.102, 0.667) | 0.264 (-.119, 0.646) | 0.338 (-.059, 0.735) | 0.334 (-.063, 0.731) |
| IDL_PL | 0.331 (-0.061, 0.723) | 0.329 (-0.059, 0.717) | 0.233 (-.155, 0.621) | 0.211 (-.174, 0.597) | 0.276 (-.123, 0.676) | 0.272 (-.128, 0.671) |
| L_LDL_PL | 0.282 (-0.102, 0.666) | 0.280 (-0.099, 0.659) | 0.185 (-.195, 0.565) | 0.161 (-.216, 0.538) | 0.251 (-.140, 0.643) | 0.248 (-.143, 0.639) |
| M_LDL_PL | 0.314 (-0.076, 0.703) | 0.309 (-0.073, 0.693) | 0.208 (-.175, 0.591) | 0.183 (-.196, 0.563) | 0.288 (-.108, 0.683) | 0.276 (-.119, 0.671) |
| S_LDL_PL | 0.336 (-0.051, 0.723) | 0.334 (-0.048, 0.715) | 0.239 (-.143, 0.621) | 0.218 (-.162, 0.597) | 0.322 (-.074, 0.718) | 0.312 (-.084, 0.708) |
| XL_HDL_PL | 0.101 (-0.179, 0.383) | 0.116 (-0.156, 0.388) | 0.107 (-.166, 0.381) | 0.114 (-.160, 0.387) | 0.052 (-.232, 0.336) | 0.038 (-.245, 0.321) |
| L_HDL_PL | 0.017 (-0.326, 0.361) | 0.036 (-0.298, 0.369) | 0.022 (-.313, 0.357) | 0.019 (-.317, 0.354) | -.049 (-.398, 0.299) | -.042 (-.391, 0.307) |
| M_HDL_PL | -0.046 (-0.445, 0.354) | -0.037 (-0.436, 0.362) | -.056 (-.457, 0.345) | -.073 (-.474, 0.328) | -.103 (-.519, 0.314) | -.075 (-.490, 0.339) |
| S_HDL_PL | -0.445 (-0.844, -0.045) | -0.443 (-0.844, -0.043) | -.444 (-.846, -.041) | -.465 (-.867, -.063) | -.507 (-.925, -.089) | -.488 (-.903, -.073) |

**Table S44. Multivariable regression analyses of pesticide exposure in years with cholesterol esters subclasses with standardised metabolomic biomarkers in men**

| **Pesticide exposure years, β (95% CI), Males** | | | | | | |
| --- | --- | --- | --- | --- | --- | --- |
| **Cholesterol esters** | **Unadjusted** | **+ BMI** | **+ SEP** | **+ season of pesticide use** | **+ alcohol consumption** | **+ latitude of residence** |
| XXL_VLDL_CE | 0.114 (-0.072, 0.301) | 0.104 (-0.077, 0.285) | 0.113 (-.079, 0.305) | 0.118 (-.073, 0.310) | 0.107 (-.088, 0.301) | 0.099 (-.095, 0.295) |
| XL_VLDL_CE | 0.028 (-0.163, 0.218) | 0.019 (-0.167, 0.204) | 0.0035 (-.192, 0.199) | 0.0098 (-.186, 0.206) | 0.0019 (-.197, 0.201) | -.004 (-.204, 0.195) |
| L_VLDL_CE | -0.032 (-0.225, 0.160) | -0.037 (-0.225, 0.151) | 0.0015 (-.198, 0.201) | 0.0016 (-.196, 0.199) | -.0046 (-.205, 0.196) | -.007 (-.208, 0.194) |
| M_VLDL_CE | 0.045 (-0.153, 0.243) | 0.035 (-0.156, 0.226) | 0.029 (-.174, 0.231) | 0.029 (-.174, 0.232) | 0.020 (-.186, 0.226) | 0.005 (-.201, 0.211) |
| S_VLDL_CE | 0.049 (-0.136, 0.234) | 0.041 (-0.139, 0.221) | 0.0096 (-.181, 0.201) | 0.0027 (-.189, 0.194) | -.007 (-.201, 0.187) | -.033 (-.227, 0.159) |
| XS_VLDL_CE | 0.089 (-0.107, 0.285) | 0.082 (-0.111, 0.274) | 0.017 (-.187, 0.221) | 0.015 (-.189, 0.219) | -.002 (-.208, 0.204) | -.028 (-.234, 0.177) |
| IDL_CE | 0.064 (-0.133, 0.260) | 0.057 (-0.137, 0.252) | -.035 (-.241, 0.171) | -.047 (-.253, 0.158) | -.047 (-.255, 0.160) | -.078 (-.284, 0.129) |
| L_LDL_CE | 0.035 (-0.163, 0.232) | 0.028 (-0.167, 0.224) | -.058 (-.265, 0.149) | -.073 (-.279, 0.134) | -.066 (-.275, 0.143) | -.097 (-.305, 0.111) |
| M_LDL_CE | 0.0093 (-0.186, 0.205) | 0.0036 (-0.191, 0.198) | -.069 (-.275, 0.137) | -.084 (-.290, 0.121) | -.075 (-.284, 0.133) | -.103 (-.310, 0.105) |
| S_LDL_CE | -0.007 (-0.209, 0.195) | -0.012 (-0.213, 0.189) | -.087 (-.300, 0.126) | -.102 (-.315, 0.111) | -.092 (-.308, 0.124) | -.118 (-.333, 0.097) |
| XL_HDL_CE | 0.006 (-0.196, 0.208) | 0.010 (-0.189, 0.209) | -.062 (-.273, 0.149) | -.055 (-.267, 0.156) | -.065 (-.279, 0.149) | -.082 (-.296, 0.132) |
| L_HDL_CE | -0.035 (-0.237, 0.166) | -0.018 (-0.212, 0.175) | -.076 (-.282, 0.131) | -.082 (-.288, 0.124) | -.079 (-.288, 0.129) | -.086 (-.294, 0.122) |
| M_HDL_CE | -0.123 (-0.319, 0.073) | -0.116 (-0.310, 0.078) | -.111 (-.316, 0.094) | -.122 (-.327, 0.083) | -.098 (-.302, 0.107) | -.087 (-.289, 0.115) |
| S_HDL_CE | -0.0005 (-0.218, 0.217) | -0.002 (-0.219, 0.216) | -.057 (-.288, 0.175) | -.078 (-.308, 0.152) | -.053 (-.286, 0.181) | -.080 (-.313, 0.152) |
| EstC | -0.020 (-0.219, 0.179) | -0.025 (-0.224, 0.174) | -.107 (-.317, 0.104) | -.123 (-.332, 0.087) | -.115 (-.328, 0.097) | -.147 (-.358, 0.064) |

**Table S45. Multivariable regression analyses of pesticide exposure in years with cholesterol esters subclasses with standardised metabolomic biomarkers in women**

| **Pesticide exposure years, β (95% CI), Females** | | | | | | |
| --- | --- | --- | --- | --- | --- | --- |
| **Cholesterol esters** | **Unadjusted** | **+ BMI** | **+ SEP** | **+ season of pesticide use** | **+ alcohol consumption** | **+ latitude of residence** |
| XXL_VLDL_CE | 0.207 (-0.198, 0.613) | 0.185 (-0.209, 0.579) | 0.141 (-.256, 0.538) | 0.151 (-.247, 0.548) | 0.154 (-.252, 0.559) | 0.159 (-.246, 0.564) |
| XL_VLDL_CE | 0.196 (-0.236, 0.627) | 0.179 (-0.241, 0.599) | 0.131 (-.291, 0.552) | 0.131 (-.289, 0.551) | 0.172 (-.257, 0.601) | 0.185 (-.243, 0.612) |
| L_VLDL_CE | -0.016 (-0.403, 0.371) | -0.017 (-0.393, 0.358) | -.044 (-.422, 0.335) | -.038 (-.416, 0.341) | 0.069 (-.325, 0.464) | 0.063 (-.332, 0.458) |
| M_VLDL_CE | 0.229 (-0.137, 0.594) | 0.217 (-0.136, 0.569) | 0.156 (-.198, 0.510) | 0.149 (-.204, 0.504) | 0.211 (-.158, 0.579) | 0.225 (-.144, 0.593) |
| S_VLDL_CE | 0.214 (-0.169, 0.596) | 0.202 (-0.168, 0.573) | 0.131 (-.241, 0.503) | 0.113 (-.257, 0.482) | 0.225 (-.158, 0.608) | 0.213 (-.170, 0.596) |
| XS_VLDL_CE | 0.249 (-0.125, 0.624) | 0.241 (-0.127, 0.609) | 0.174 (-.195, 0.544) | 0.165 (-.204, 0.535) | 0.236 (-.145, 0.618) | 0.224 (-.158, 0.606) |
| IDL_CE | 0.265 (-0.117, 0.647) | 0.261 (-0.116, 0.639) | 0.170 (-.208, 0.548) | 0.149 (-.226, 0.526) | 0.221 (-.168, 0.611) | 0.218 (-.172, 0.608) |
| L_LDL_CE | 0.294 (-0.099, 0.687) | 0.291 (-0.098, 0.679) | 0.201 (-.188, 0.591) | 0.178 (-.208, 0.564) | 0.308 (-.092, 0.708) | 0.304 (-.097, 0.704) |
| M_LDL_CE | 0.264 (-0.127, 0.656) | 0.262 (-0.126, 0.649) | 0.187 (-.201, 0.576) | 0.162 (-.224, 0.547) | 0.343 (-.056, 0.741) | 0.340 (-.058, 0.739) |
| S_LDL_CE | 0.251 (-0.131, 0.633) | 0.249 (-0.129, 0.627) | 0.182 (-.197, 0.562) | 0.158 (-.219, 0.534) | 0.335 (-.055, 0.725) | 0.332 (-.058, 0.722) |
| XL_HDL_CE | 0.140 (-0.231, 0.512) | 0.150 (-0.219, 0.520) | 0.134 (-.238, 0.506) | 0.142 (-.230, 0.514) | 0.089 (-.299, 0.478) | 0.038 (-.343, 0.418) |
| L_HDL_CE | -0.0025 (-0.318, 0.313) | 0.016 (-0.287, 0.318) | 0.0063 (-.299, 0.311) | 0.0069 (-.298, 0.312) | -.064 (-.380, 0.253) | -.066 (-.383, 0.251) |
| M_HDL_CE | -0.181 (-0.564, 0.203) | -0.173 (-0.555, 0.207) | -.172 (-.556, 0.212) | -.187 (-.571, 0.197) | -.195 (-.594, 0.204) | -.162 (-.559, 0.234) |
| S_HDL_CE | 0.150 (-0.204, 0.505) | 0.151 (-0.203, 0.504) | 0.083 (-.271, 0.436) | 0.052 (-.297, 0.402) | 0.173 (-.193, 0.538) | 0.161 (-.202, 0.524) |
| EstC | 0.278 (-0.109, 0.665) | 0.279 (-0.106, 0.663) | 0.192 (-.193, 0.577) | 0.169 (-.213, 0.552) | 0.237 (-.162, 0.636) | 0.231 (-.168, 0.631) |

**Table S46. Multivariable regression analyses of pesticide exposure in years with free cholesterol subclasses with standardised metabolomic biomarkers in men**

| **Pesticide exposure years, β (95% CI), Males** | | | | | | |
| --- | --- | --- | --- | --- | --- | --- |
| **Free cholesterol** | **Unadjusted** | **+ BMI** | **+ SEP** | **+ season of pesticide use** | **+ alcohol consumption** | **+ latitude of residence** |
| XXL_VLDL_FC | 0.053 (-0.142, 0.247) | 0.042 (-0.147, 0.231) | 0.016 (-.184, 0.216) | 0.019 (-.180, 0.220) | 0.019 (-.184, 0.222) | 0.013 (-.191, 0.216) |
| XL_VLDL_FC | 0.019 (-0.179, 0.216) | 0.0095 (-0.182, 0.201) | -.020 (-.223, 0.182) | -.019 (-.221, 0.184) | -.022 (-.228, 0.184) | -.033 (-.239, 0.173) |
| L_VLDL_FC | -0.048 (-0.236, 0.139) | -0.052 (-0.234, 0.130) | -.035 (-.228, 0.159) | -.038 (-.231, 0.156) | -.029 (-.226, 0.167) | -.029 (-.227, 0.167) |
| M_VLDL_FC | -0.016 (-0.207, 0.175) | -0.025 (-0.209, 0.158) | -.015 (-.209, 0.179) | -.017 (-.212, 0.179) | -.012 (-.210, 0.1856) | -.018 (-.216, 0.180) |
| S_VLDL_FC | -0.031 (-0.209, 0.148) | -0.039 (-0.212, 0.134) | -.046 (-.229, 0.138) | -.054 (-.238, 0.129) | -.052 (-.239, 0.134) | -.073 (-.259, 0.113) |
| XS_VLDL_FC | -0.0046 (-0.201, 0.192) | -0.011 (-0.205, 0.183) | -.059 (-.266, 0.147) | -.070 (-.276, 0.136) | -.072 (-.280, 0.137) | -.104 (-.312, 0.104) |
| IDL_FC | 0.046 (-0.162, 0.254) | 0.042 (-0.166, 0.249) | -.044 (-.264, 0.175) | -.058 (-.277, 0.161) | -.057 (-.278, 0.165) | -.089 (-.309, 0.131) |
| L_LDL_FC | 0.059 (-0.145, 0.263) | 0.054 (-0.149, 0.258) | -.043 (-.259, 0.172) | -.059 (-.273, 0.156) | -.053 (-.270, 0.164) | -.087 (-.303, 0.129) |
| M_LDL_FC | 0.037 (-0.159, 0.233) | 0.031 (-0.164, 0.226) | -.071 (-.277, 0.135) | -.088 (-.293, 0.118) | -.078 (-.286, 0.129) | -.115 (-.321, 0.091) |
| S_LDL_FC | 0.028 (-0.172, 0.228) | 0.022 (-0.177, 0.220) | -.084 (-.294, 0.126) | -.099 (-.309, 0.110) | -.088 (-.299, 0.124) | -.126 (-.336, 0.085) |
| XL_HDL_FC | 0.040 (-0.173, 0.254) | 0.045 (-0.162, 0.252) | -.016 (-.235, 0.204) | -.0087 (-.228, 0.211) | -.019 (-.242, 0.204) | -.017 (-.240, 0.206) |
| L_HDL_FC | -0.044 (-0.255, 0.166) | -0.026 (-0.227, 0.176) | -.074 (-.289, 0.139) | -.079 (-.294, 0.135) | -.076 (-.293, 0.141) | -.083 (-.300, 0.134) |
| M_HDL_FC | -0.130 (-0.332, 0.072) | -0.124 (-0.324, 0.076) | -.137 (-.349, 0.075) | -.153 (-.364, 0.059) | -.128 (-.339, 0.083) | -.126 (-.335, 0.083) |
| S_HDL_FC | -0.057 (-0.257, 0.143) | -0.056 (-0.256, 0.144) | -.107 (-.319, 0.104) | -.126 (-.337, 0.084) | -.101 (-.311, 0.109) | -.119 (-.328, 0.089) |
| Free cholesterol | 0.00021 (-0.202, 0.202) | -0.004 (-0.206, 0.198) | -.097 (-.311, 0.116) | -.112 (-.325, 0.101) | -.105 (-.319, 0.111) | -.137 (-.351, 0.077) |

**Table S47. Multivariable regression analyses of pesticide exposure in years with free cholesterol subclasses with standardised metabolomic biomarkers in women**

| **Pesticide exposure years, β (95% CI), Females** | | | | | | |
| --- | --- | --- | --- | --- | --- | --- |
| **Free cholesterol** | **Unadjusted** | **+ BMI** | **+ SEP** | **+ season of pesticide use** | **+ alcohol consumption** | **+ latitude of residence** |
| XXL_VLDL_FC | 0.164 (-0.218, 0.545) | 0.142 (-0.227, 0.511) | 0.113 (-.259, 0.486) | 0.118 (-.254, 0.491) | 0.108 (-.273, 0.489) | 0.132 (-.249, 0.513) |
| XL_VLDL_FC | 0.144 (-0.266, 0.554) | 0.128 (-0.269, 0.526) | 0.064 (-.332, 0.459) | 0.058 (-.337, 0.454) | 0.079 (-.327, 0.487) | 0.097 (-.310, 0.504) |
| L_VLDL_FC | -0.068 (-.454, 0.318) | -0.069 (-0.442, 0.303) | -.088 (-.464, 0.287) | -.096 (-.471, 0.279) | -.016 (-.407, 0.376) | 0.009 (-.383, 0.401) |
| M_VLDL_FC | 0.153 (-0.216, 0.521) | 0.139 (-0.213, 0.492) | 0.100 (-.255, 0.455) | 0.092 (-.263, 0.446) | 0.163 (-.206, 0.531) | 0.186 (-.183, 0.554) |
| S_VLDL_FC | 0.288 (-0.101, 0.677) | 0.276 (-0.098, 0.651) | 0.224 (-.153, 0.600) | 0.209 (-.166, 0.583) | 0.294 (-.095, 0.683) | 0.286 (-.103, 0.676) |
| XS_VLDL_FC | 0.332 (-0.050, 0.714) | 0.327 (-0.049, 0.703) | 0.256 (-.122, 0.633) | 0.239 (-.137, 0.615) | 0.318 (-.0728, 0.708) | 0.309 (-.082, 0.699) |
| IDL_FC | 0.323 (-0.062, 0.708) | 0.323 (-0.059, 0.705) | 0.234 (-.149, 0.617) | 0.214 (-.167, 0.595) | 0.281 (-.114, 0.676) | 0.278 (-.117, 0.672) |
| L_LDL_FC | 0.314 (-0.068, 0.696) | 0.313 (-0.066, 0.693) | 0.219 (-.160, 0.598) | 0.197 (-.179, 0.573) | 0.286 (-.105, 0.676) | 0.276 (-.114, 0.667) |
| M_LDL_FC | 0.309 (-0.069, 0.689) | 0.308 (-0.067, 0.683) | 0.208 (-.167, 0.582) | 0.184 (-.187, 0.556) | 0.292 (-.094, 0.678) | 0.274 (-.112, 0.659) |
| S_LDL_FC | 0.322 (-0.066, 0.709) | 0.319 (-0.063, 0.702) | 0.221 (-.162, 0.603) | 0.198 (-.182, 0.578) | 0.319 (-.077, 0.715) | 0.295 (-.100, 0.691) |
| XL_HDL_FC | 0.145 (-0.193, 0.482) | 0.159 (-0.172, 0.490) | 0.141 (-.193, 0.475) | 0.152 (-.182, 0.485) | 0.091 (-.256, 0.439) | 0.074 (-.270, 0.419) |
| L_HDL_FC | 0.070 (-0.234, 0.375) | 0.089 (-0.201, 0.379) | 0.085 (-.208, 0.377) | 0.087 (-.206, 0.379) | 0.033 (-.270, 0.337) | 0.033 (-.271, 0.337) |
| M_HDL_FC | -0.022 (-0.401, 0.356) | -0.013 (-0.389, 0.364) | -.032 (-.411, 0.347) | -.049 (-.428, 0.330) | -.064 (-.458, 0.330) | -.039 (-.431, 0.354) |
| S_HDL_FC | -0.349 (-0.767, 0.068) | -0.345 (-0.762, 0.073) | -.381 (-.801, 0.038) | -.407 (-.825, 0.010) | -.444 (-.880, -.0084) | -.442 (-.876, -.0087) |
| Free cholesterol | 0.332 (-0.067, 0.730) | 0.333 (-0.063, 0.728) | 0.236 (-.159, 0.633) | 0.219 (-.176, 0.613) | 0.263 (-.148, 0.674) | 0.262 (-.149, 0.673) |

**Table S48. Multivariable regression analyses of pesticide exposure in years with total lipid subclasses with standardised metabolomic biomarkers in men**

| **Pesticide exposure years, β (95% CI), Males** | | | | | | |
| --- | --- | --- | --- | --- | --- | --- |
| **Total lipids** | **Unadjusted** | **+ BMI** | **+ SEP** | **+ season of pesticide use** | **+ alcohol consumption** | **+ latitude of residence** |
| XXL_VLDL_L | 0.039 (-0.164, 0.241) | 0.026 (-0.168, 0.221) | -.00062 (-.207, 0.206) | 0.0036 (-.203, 0.209) | -.00059 (-.209, 0.209) | -.0033 (-.213, 0.206) |
| XL_VLDL_L | -0.021 (-0.223, 0.179) | -0.030 (-0.225, 0.164) | -.049 (-.255, 0.156) | -.047 (-.253, 0.159) | -.043 (-.252, 0.166) | -.046 (-.255, 0.164) |
| L_VLDL_L | -0.097 (-0.291, 0.097) | -0.101 (-0.288, 0.086) | -.087 (-.285, 0.112) | -.089 (-.288, 0.108) | -.078 (-.279, 0.123) | -.075 (-.276, 0.126) |
| M_VLDL_L | -0.034 (-0.226, 0.159) | -0.043 (-0.228, 0.142) | -.032 (-.228, 0.165) | -.033 (-.229, 0.164) | -.029 (-.228, 0.171) | -.032 (-.231, 0.168) |
| S_VLDL_L | -0.034 (-0.214, 0.147) | -0.043 (-0.216, 0.129) | -.044 (-.228, 0.139) | -.051 (-.235, 0.133) | -.051 (-.237, 0.136) | -.069 (-.255, 0.118) |
| XS_VLDL_L | 0.007 (-0.182, 0.196) | -0.001 (-0.186, 0.183) | -.052 (-.248, 0.144) | -.062 (-.258, 0.134) | -.066 (-.265, 0.132) | -.098 (-.295, 0.099) |
| IDL_L | 0.054 (-0.143, 0.251) | 0.048 (-0.148, 0.243) | -.039 (-.247, 0.167) | -.054 (-.260, 0.153) | -.052 (-.261, 0.157) | -.086 (-.293, 0.122) |
| L_LDL_L | 0.039 (-0.159, 0.239) | 0.033 (-0.163, 0.230) | -.056 (-.265, 0.152) | -.072 (-.280, 0.135) | -.066 (-.276, 0.144) | -.100 (-.309, 0.109) |
| M_LDL_L | 0.018 (-0.181, 0.217) | 0.011 (-0.186, 0.208) | -.073 (-.282, 0.135) | -.090 (-.298, 0.117) | -.079 (-.290, 0.130) | -.114 (-.323, 0.095) |
| S_LDL_L | 0.00095 (-0.194, 0.196) | -0.0058 (-0.199, 0.187) | -.089 (-.294, 0.115) | -.106 (-.309, 0.098) | -.093 (-.299, 0.113) | -.125 (-.330, 0.079) |
| XL_HDL_L | 0.020 (-0.178, 0.219) | 0.030 (-0.163, 0.223) | -.024 (-.228, 0.181) | -.021 (-.226, 0.184) | -.031 (-.239, 0.176) | -.041 (-.249, 0.167) |
| L_HDL_L | -0.061 (-0.258, 0.135) | -0.045 (-0.234, 0.144) | -.100 (-.302, 0.101) | -.109 (-.311, 0.092) | -.105 (-.308, 0.098) | -.112 (-.315, 0.090) |
| M_HDL_L | -0.125 (-0.317, 0.067) | -0.119 (-0.311, 0.071) | -.129 (-.332, 0.073) | -.143 (-.345, 0.059) | -.119 (-.319, 0.082) | -.115 (-.314, 0.083) |
| S_HDL_L | -0.055 (-0.254, 0.145) | -0.055 (-0.255, 0.144) | -.108 (-.318, 0.103) | -.129 (-.339, 0.079) | -.099 (-.308, 0.111) | -.119 (-.326, 0.088) |

**Table S49. Multivariable regression analyses of pesticide exposure in years with total lipid subclasses with standardised metabolomic biomarkers in women**

| **Pesticide exposure years, β (95% CI), Females** | | | | | | |
| --- | --- | --- | --- | --- | --- | --- |
| **Total lipids** | **Unadjusted** | **+ BMI** | **+ SEP** | **+ season of pesticide use** | **+ alcohol consumption** | **+ latitude of residence** |
| XXL_VLDL_L | 0.122 (-0.242, 0.486) | 0.098 (-0.250, 0.446) | 0.070 (-.280, 0.421) | 0.077 (-.273, 0.428) | 0.096 (-.262, 0.455) | 0.109 (-.249, 0.468) |
| XL_VLDL_L | 0.126 (-0.274, 0.526) | 0.109 (-0.276, 0.494) | 0.061 (-.327, 0.449) | 0.056 (-.332, 0.444) | 0.094 (-.304, 0.491) | 0.124 (-.273, 0.521) |
| L_VLDL_L | -0.138 (-0.501, 0.225) | -0.139 (-0.488, 0.208) | -.157 (-.507, 0.194) | -.159 (-.510, 0.190) | -.046 (-.411, 0.319) | -.021 (-.387, 0.345) |
| M_VLDL_L | 0.100 (-0.252, 0.453) | 0.087 (-0.249, 0.423) | 0.052 (-.286, 0.391) | 0.047 (-.290, 0.385) | 0.144 (-.207, 0.496) | 0.169 (-.181, 0.521) |
| S_VLDL_L | 0.245 (-0.129, 0.620) | 0.232 (-0.126, 0.589) | 0.183 (-.177, 0.544) | 0.171 (-.187, 0.529) | 0.268 (-.104, 0.641) | 0.264 (-.109, 0.637) |
| XS_VLDL_L | 0.346 (-0.034, 0.727) | 0.338 (-0.033, 0.709) | 0.264 (-.108, 0.636) | 0.251 (-.120, 0.621) | 0.324 (-.060, 0.709) | 0.314 (-.071, 0.699) |
| IDL_L | 0.328 (-0.053, 0.709) | 0.325 (-0.051, 0.701) | 0.233 (-.144, 0.609) | 0.213 (-.161, 0.587) | 0.274 (-.114, 0.662) | 0.267 (-.121, 0.655) |
| L_LDL_L | 0.328 (-0.060, 0.716) | 0.325 (-0.058, 0.708) | 0.231 (-.152, 0.615) | 0.209 (-.172, 0.589) | 0.308 (-.087, 0.703) | 0.300 (-.095, 0.695) |
| M_LDL_L | 0.336 (-0.059, 0.731) | 0.333 (-0.056, 0.722) | 0.243 (-.147, 0.633) | 0.218 (-.168, 0.605) | 0.349 (-.052, 0.749) | 0.339 (-.061, 0.739) |
| S_LDL_L | 0.331 (-0.056, 0.717) | 0.328 (-0.053, 0.709) | 0.245 (-.137, 0.626) | 0.222 (-.157, 0.599) | 0.354 (-.039, 0.747) | 0.344 (-.049, 0.737) |
| XL_HDL_L | 0.137 (-0.207, 0.481) | 0.153 (-0.184, 0.489) | 0.136 (-.203, 0.475) | 0.145 (-.194, 0.484) | 0.071 (-.281, 0.424) | 0.042 (-.307, 0.390) |
| L_HDL_L | 0.019 (-0.320, 0.358) | 0.038 (-0.289, 0.365) | 0.021 (-.308, 0.349) | 0.019 (-.310, 0.349) | -.055 (-.397, 0.288) | -.054 (-.396, 0.289) |
| M_HDL_L | -0.084 (-0.472, 0.304) | -0.076 (-0.464, 0.311) | -.091 (-.481, 0.298) | -.109 (-.499, 0.281) | -.130 (-.535, 0.275) | -.100 (-.503, 0.303) |
| S_HDL_L | -0.063 (-0.477, 0.351) | -0.063 (-0.477, 0.352) | -.117 (-.533, 0.298) | -.148 (-.561, 0.265) | -.118 (-.549, 0.313) | -.116 (-.543, 0.312) |

**Table S50. Multivariable regression analyses of pesticide exposure in years with total cholesterol subclasses with standardised metabolomic biomarkers in men**

| **Pesticide exposure years, β (95% CI), Males** | | | | | | |
| --- | --- | --- | --- | --- | --- | --- |
| **Total cholesterol** | **Unadjusted** | **+ BMI** | **+ SEP** | **+ season of pesticide use** | **+ alcohol consumption** | **+ latitude of residence** |
| XXL_VLDL_C | 0.089 (-0.105, 0.282) | 0.078 (-0.110, 0.265) | 0.067 (-.132, 0.265) | 0.072 (-.127, 0.271) | 0.063 (-.139, 0.265) | 0.056 (-.147, 0.258) |
| XL_VLDL_C | 0.016 (-0.181, 0.213) | 0.0069 (-0.184, 0.198) | -.018 (-.219, 0.184) | -.014 (-.215, 0.188) | -.019 (-.224, 0.186) | -.028 (-.234, 0.177) |
| L_VLDL_C | -0.056 (-0.249, 0.136) | -0.061 (-0.248, 0.126) | -.030 (-.228, 0.168) | -.031 (-.229, 0.167) | -.031 (-.232, 0.169) | -.034 (-.235, 0.167) |
| M_VLDL_C | 0.015 (-0.180, 0.211) | 0.0053 (-0.182, 0.193) | 0.0062 (-.193, 0.205) | 0.0053 (-.194, 0.205) | 0.0019 (-.201, 0.204) | -.0089 (-.212, 0.194) |
| S_VLDL_C | 0.019 (-0.161, 0.201) | 0.011 (-0.165, 0.187) | -.011 (-.198, 0.175) | -.019 (-.206, 0.168) | -.025 (-.214, 0.164) | -.049 (-.239, 0.139) |
| XS_VLDL_C | 0.059 (-0.140, 0.258) | 0.052 (-0.144, 0.248) | -.0095 (-.217, 0.198) | -.015 (-.223, 0.193) | -.027 (-.238, 0.183) | -.057 (-.266, 0.153) |
| IDL_C | 0.057 (-0.143, 0.258) | 0.051 (-0.147, 0.250) | -.040 (-.251, 0.169) | -.053 (-.264, 0.157) | -.053 (-.265, 0.159) | -.084 (-.296, 0.127) |
| L_LDL_C | 0.041 (-0.159, 0.240) | 0.034 (-0.164, 0.232) | -.055 (-.265, 0.155) | -.070 (-.279, 0.139) | -.064 (-.276, 0.148) | -.096 (-.306, 0.115) |
| M_LDL_C | 0.014 (-0.181, 0.209) | 0.0083 (-0.185, 0.202) | -.071 (-.276, 0.134) | -.086 (-.291, 0.118) | -.077 (-.284, 0.130) | -.107 (-.313, 0.099) |
| S_LDL_C | -0.00064 (-0.196, 0.194) | -0.0062 (-0.200, 0.188) | -.086 (-.292, 0.119) | -.101 (-.306, 0.104) | -.090 (-.298, 0.118) | -.119 (-.325, 0.088) |
| LDL_C | 0.024 (-0.171, 0.219) | 0.018 (-0.175, 0.211) | -.066 (-.270, 0.139) | -.081 (-.285, 0.124) | -.072 (-.279, 0.134) | -.103 (-.308, 0.103) |
| XL_HDL_C | 0.013 (-0.189, 0.216) | 0.018 (-0.181, 0.216) | -.054 (-.265, 0.157) | -.047 (-.259, 0.164) | -.058 (-.272, 0.157) | -.069 (-.284, 0.144) |
| L_HDL_C | -0.037 (-0.240, 0.166) | -0.019 (-0.214, 0.175) | -.075 (-.283, 0.132) | -.081 (-.289, 0.126) | -.079 (-.288, 0.131) | -.085 (-.295, 0.124) |
| M_HDL_C | -0.127 (-0.324, 0.069) | -0.120 (-0.315, 0.074) | -.118 (-.324, 0.087) | -.131 (-.336, 0.075) | -.106 (-.311, 0.099) | -.097 (-.299, 0.106) |
| S_HDL_C | -0.016 (-0.223, 0.192) | -0.017 (-0.224, 0.191) | -.074 (-.294, 0.146) | -.098 (-.317, 0.121) | -.069 (-.290, 0.151) | -.097 (-.316, 0.123) |
| HDL_C_ | -0.059 (-0.248, 0.129) | -0.050 (-0.233, 0.133) | -.096 (-.290, 0.098) | -.109 (-.302, 0.084) | -.097 (-.291, 0.098) | -.106 (-.299, 0.088) |
| Serum C | 0.00072 (-0.204, 0.206) | -0.004 (-0.208, 0.200) | -.090 (-.306, 0.126) | -.107 (-.322, 0.109) | -.099 (-.317, 0.119) | -.132 (-.348, 0.085) |
| Remnant C | 0.032 (-0.162, 0.225) | 0.022 (-0.166, 0.210) | -.035 (-.234, 0.164) | -.044 (-.243, 0.155) | -.048 (-.250, 0.154) | -.075 (-.276, 0.126) |

**Table S51. Multivariable regression analyses of pesticide exposure in years with total cholesterol subclasses with standardised metabolomic biomarkers in women**

| **Pesticide exposure years, β (95% CI), Females** | | | | | | |
| --- | --- | --- | --- | --- | --- | --- |
| **Total cholesterol** | **Unadjusted** | **+ BMI** | **+ SEP** | **+ season of pesticide use** | **+ alcohol consumption** | **+ latitude of residence** |
| XXL_VLDL_C | 0.185 (-0.201, 0.571) | 0.162 (-0.210, 0.535) | 0.121 (-.254, 0.497) | 0.129 (-.247, 0.504) | 0.127 (-.257, 0.510) | 0.139 (-.244, 0.523) |
| XL_VLDL_C | 0.166 (-0.242, 0.574) | 0.149 (-0.245, 0.543) | 0.090 (-.304, 0.484) | 0.087 (-.306, 0.481) | 0.121 (-.282, 0.524) | 0.137 (-.266, 0.539) |
| L_VLDL_C | -0.079 (-0.453, 0.295) | -0.081 (-0.441, 0.279) | -.106 (-.469, 0.256) | -.106 (-.469, 0.257) | 0.0046 (-.374, 0.383) | 0.014 (-.365, 0.393) |
| M_VLDL_C | 0.194 (-0.166, 0.554) | 0.181 (-0.164, 0.527) | 0.128 (-.219, 0.476) | 0.122 (-.225, 0.468) | 0.189 (-.172, 0.549) | 0.206 (-.155, 0.567) |
| S_VLDL_C | 0.238 (-0.140, 0.617) | 0.226 (-0.138, 0.591) | 0.161 (-.206, 0.527) | 0.144 (-.221, 0.508) | 0.248 (-.130, 0.626) | 0.237 (-.141, 0.616) |
| XS_VLDL_C | 0.289 (-0.094, 0.672) | 0.281 (-0.095, 0.658) | 0.209 (-.169, 0.587) | 0.198 (-.179, 0.575) | 0.275 (-.116, 0.665) | 0.263 (-.128, 0.654) |
| IDL_C | 0.288 (-0.100, 0.676) | 0.285 (-0.099, 0.669) | 0.193 (-.192, 0.578) | 0.173 (-.210, 0.555) | 0.244 (-.153, 0.641) | 0.241 (-.156, 0.638) |
| L_LDL_C | 0.299 (-0.092, 0.692) | 0.297 (-0.091, 0.685) | 0.206 (-.183, 0.594) | 0.183 (-.203, 0.568) | 0.302 (-.098, 0.701) | 0.296 (-.103, 0.696) |
| M_LDL_C | 0.293 (-0.099, 0.686) | 0.291 (-0.098, 0.679) | 0.208 (-.181, 0.597) | 0.183 (-.203, 0.569) | 0.339 (-.060, 0.739) | 0.334 (-.066, 0.733) |
| S_LDL_C | 0.284 (-0.102, 0.670) | 0.281 (-0.101, 0.664) | 0.205 (-.178, 0.589) | 0.181 (-.199, 0.562) | 0.341 (-.054, 0.735) | 0.333 (-.062, 0.727) |
| LDL_C | 0.295 (-0.092, 0.681) | 0.292 (-0.090, 0.674) | 0.207 (-.176, 0.589) | 0.183 (-.197, 0.563) | 0.319 (-.075, 0.713) | 0.313 (-.081, 0.707) |
| XL_HDL_C | 0.141 (-0.217, 0.500) | 0.153 (-0.202, 0.509) | 0.136 (-.222, 0.493) | 0.145 (-.213, 0.502) | 0.088 (-.285, 0.461) | 0.045 (-.321, 0.411) |
| L_HDL_C | 0.016 (-0.301, 0.334) | 0.035 (-0.269, 0.339) | 0.026 (-.279, 0.332) | 0.027 (-.279, 0.334) | -.041 (-.359, 0.277) | -.043 (-.361, 0.276) |
| M_HDL_C | -0.145 (-0.524, 0.234) | -0.137 (-0.514, 0.240) | -.140 (-.519, 0.239) | -.156 (-.536, 0.224) | -.165 (-.559, 0.229) | -.134 (-.526, 0.258) |
| S_HDL_C | 0.107 (-0.272, 0.485) | 0.108 (-0.269, 0.485) | 0.036 (-.342, 0.413) | 0.0038 (-.370, 0.378) | 0.100 (-.291, 0.491) | 0.090 (-.298, 0.479) |
| HDL_C_ | 0.016 (-0.343, 0.376) | 0.033 (-0.319, 0.384) | 0.0046 (-.349, 0.358) | -.004 (-.358, 0.349) | -.045 (-.413, 0.323) | -.045 (-.414, 0.323) |
| Serum C | 0.301 (-0.098, 0.701) | 0.303 (-0.094, 0.699) | 0.209 (-.188, 0.607) | 0.188 (-.207, 0.582) | 0.254 (-.158, 0.665) | 0.249 (-.163, 0.661) |
| Remnant C | 0.274 (-0.099, 0.647) | 0.265 (-0.099, 0.628) | 0.183 (-.181, 0.548) | 0.168 (-.195, 0.530) | 0.253 (-.123, 0.629) | 0.252 (-.124, 0.629) |

**Table S52. Multivariable regression analyses of pesticide exposure in years (PEY) with apolipoproteins, aminoacids, sphingomyelins, glucose metabolism, fatty acids, inflammation, and fluid balance with standardised metabolomic biomarkers in men**

| **Pesticide exposure years, β (95% CI), Males** | | | | | | |
| --- | --- | --- | --- | --- | --- | --- |
|  | **Unadjusted** | **+ BMI** | **+ SEP** | **+ season of pesticide use** | **+ alcohol consumption** | **+ latitude of residence** |
| **Apolipoproteins** |  |  |  |  |  |  |
| Apolipoprotein A1 [g/L] | -0.087 (-0.272, 0.098) | -0.083 (-0.267, 0.101) | -.128 (-.323, 0.067) | -.145 (-.339, 0.049) | -.129 (-.324, 0.065) | -.144 (-.337, 0.049) |
| Apolipoprotein B [g/L] | -0.007 (-0.202, 0.188) | -0.017 (-0.206, 0.172) | -.076 (-.277, 0.124) | -.089 (-.289, 0.112) | -.086 (-.289, 0.117) | -.112 (-.314, 0.091) |
| Ratio of Apo B/Apo A1 | 0.048 (-0.136, 0.233) | 0.036 (-0.137, 0.209) | 0.0084 (-.176, 0.193) | 0.008 (-.177, 0.193) | 0.0006 (-.186, 0.187) | -.015 (-.201, 0.171) |
| **Amino acids** |  |  |  |  |  |  |
| Alanine [µmol/L] | -0.181 (-0.381, 0.019) | -0.186 (-0.384, 0.012) | -.076 (-.285, 0.134) | -.088 (-.297, 0.121) | -.083 (-.295, 0.128) | -.109 (-.319, 0.102) |
| Glutamine [µmol/L] | -0.002 (-0.211, 0.207) | 0.0021 (-0.206, 0.210) | -.088 (-.309, 0.133) | -.109 (-.327, 0.108) | -.089 (-.308, 0.129) | -.086 (-.305, 0.132) |
| Glycine [µmol/L] | -0.072 (-0.246, 0.101) | -0.069 (-0.242, 0.104) | -.087 (-.269, 0.095) | -.098 (-.279, 0.083) | -.116 (-.299, 0.066) | -.162 (-.341, 0.017) |
| Histidine [µmol/L] | 0.143 (-0.076, 0.362) | 0.139 (-0.079, 0.358) | 0.175 (-.056, 0.407) | 0.179 (-.053, 0.410) | 0.167 (-.066, 0.400) | 0.184 (-.049, 0.417) |
| **Branched-chain amino acids** |  |  |  |  |  |  |
| Isoleucine [µmol/L] | -0.212 (-0.397, -0.026) | -0.222 (-0.401, -0.042) | -.189 (-.379, 0.0004) | -.195 (-.386, -.0051) | -.190 (-.383, 0.0024) | -.208 (-.400, -.016) |
| Leucine [µmol/L] | -0.179 (-0.361, 0.0016) | -0.189 (-0.365, -0.012) | -.149 (-.336, 0.038) | -.163 (-.349, 0.024) | -.159 (-.349, 0.030) | -.188 (-.377, 0.00048) |
| Valine [µmol/L] | -0.259 (-0.435, -0.083) | -0.266 (-0.439, -0.092) | -.229 (-.413, -.046) | -.241 (-.424, -.058) | -.234 (-.419, -.048) | -.259 (-.443, -.074) |
| **Aromatic amino acids** |  |  |  |  |  |  |
| Phenylalanine [µmol/L] | 0.127 (-0.063, 0.318) | 0.118 (-0.066, 0.301) | 0.059 (-.136, 0.253) | 0.034 (-.158, 0.227) | 0.029 (-.165, 0.224) | -.029 (-.219, 0.160) |
| Tyrosine [µmol/L] | -0.028 (-0.221, 0.166) | -0.034 (-0.224, 0.156) | -.00089 (-.202, 0.201) | -.0067 (-.207, 0.193) | 0.00037 (-.203, 0.204) | -.0072 (-.210, 0.196) |
| **Ketone bodies** |  |  |  |  |  |  |
| Acetoacetate [µmol/L] | -0.089 (-0.285, 0.108) | -0.084 (-0.280, 0.112) | -.165 (-.372, 0.043) | -.171 (-.379, 0.037) | -.153 (-.363, 0.056) | -.158 (-.367, 0.052) |
| Acetate [µmol/L] | 0.179 (-0.023, 0.381) | 0.182 (-0.019, 0.383) | 0.260 (0.047, 0.473) | 0.257 (0.044, 0.469) | 0.276 (0.060, 0.492) | 0.259 (0.043, 0.476) |
| beta-hydroxybutyrate [µmol/L] | -0.057 (-0.247, 0.134) | -0.054 (-0.243, 0.136) | -.153 (-.353, 0.047) | -.166 (-.366, 0.033) | -.161 (-.361, 0.039) | -.187 (-.387, 0.012) |
| **Sphingolipids** |  |  |  |  |  |  |
| Sphingomyelin | -0.072 (-0.276, 0.133) | -0.074 (-0.279, 0.130) | -.166 (-.383, 0.051) | -.187 (-.402, 0.029) | -.180 (-.398, 0.038) | -.211 (-.427, 0.0049) |
| Total choline | -0.069 (-0.259, 0.120) | -0.071 (-0.261, 0.119) | -.138 (-.339, 0.063) | -.154 (-.354, 0.046) | -.142 (-.343, 0.059) | -.167 (-.367, 0.033) |
| **Glycolysis related metabolites** |  |  |  |  |  |  |
| Citrate [µmol/L] | 0.049 (-0.156, 0.255) | 0.050 (-0.155, 0.256) | 0.00035 (-.217, 0.218) | -.023 (-.239, 0.193) | 0.0031 (-.216, 0.222) | -.025 (-.243, 0.192) |
| Glucose [mmol/L] | -0.001 (-0.200, 0.198) | -0.0025 (-0.202, 0.196) | -.072 (-.282, 0.138) | -.096 (-.304, 0.113) | -.094 (-.304, 0.117) | -.111 (-.321, 0.099) |
| Glycerol [mmol/L] | 0.055 (-0.157, 0.267) | 0.056 (-0.155, 0.268) | 0.107 (-.117, 0.332) | 0.131 (-.093, 0.354) | 0.137 (-.089, 0.364) | 0.157 (-.069, 0.383) |
| Lactate [mmol/L] | -0.199 (-0.398, 0.00052) | -0.202 (-0.401, -0.003) | -.112 (-.323, 0.098) | -.115 (-.326, 0.095) | -.108 (-.320, 0.105) | -.156 (-.365, 0.053) |
| Pyruvate [mmol/L] | 0.085 (-0.118, 0.288) | 0.086 (-0.117, 0.289) | 0.053 (-.161, 0.267) | 0.067 (-.146, 0.281) | 0.049 (-.167, 0.266) | 0.056 (-.161, 0.273) |
| **Fatty acids** |  |  |  |  |  |  |
| Total fatty acids [mmol/L] | -0.099 (-0.297, 0.099) | -0.106 (-0.301, 0.089) | -.145 (-.351, 0.062) | -.158 (-.364, 0.048) | -.146 (-.354, 0.062) | -.169 (-.377, 0.037) |
| Monounsaturated fatty acids [mmol/L] | -0.129 (-0.324, 0.066) | -0.136 (-0.327, 0.054) | -.152 (-.353, 0.049) | -.159 (-.361, 0.042) | -.150 (-.353, 0.052) | -.167 (-.369, 0.035) |
| Saturated fatty acids [mmol/L] | 0.019 (-0.176, 0.215) | 0.013 (-0.179, 0.205) | -.067 (-.271, 0.137) | -.079 (-.283, 0.124) | -.060 (-.266, 0.145) | -.088 (-.292, 0.116) |
| **Inflammation** |  |  |  |  |  |  |
| Alpha-1-acid glycoprotein [mmol/L] | -0.124 (-0.329, 0.081) | -0.132 (-0.333, 0.069) | -.139 (-.352, 0.074) | -.158 (-.369, 0.054) | -.161 (-.376, 0.054) | -.185 (-.399, 0.029) |
| **Fluid balance** |  |  |  |  |  |  |
| Creatinine [µmol/L] | 0.085 (-0.178, 0.348) | 0.079 (-0.183, 0.341) | 0.170 (-.107, 0.447) | 0.169 (-.107, 0.445) | 0.173 (-.107, 0.454) | 0.188 (-.093, 0.469) |
| Albumin [standardized concentration units] | -0.107 (-0.329, 0.115) | -0.106 (-0.329, 0.116) | -.162 (-.399, 0.074) | -.184 (-.419, 0.051) | -.172 (-.411, 0.066) | -.197 (-.433, 0.040) |

**Table S53. Multivariable regression analyses of pesticide exposure in years (PEY) with apolipoproteins, aminoacids, sphingomyelins, glucose metabolism, fatty acids, inflammation, and fluid balance with standardised metabolomic biomarkers in women**

| **Pesticide exposure years, β (95% CI), Females** | | | | | | |
| --- | --- | --- | --- | --- | --- | --- |
|  | **Unadjusted** | **+ BMI** | **+ SEP** | **+ season of pesticide use** | **+ alcohol consumption** | **+ latitude of residence** |
| **Apolipoproteins** |  |  |  |  |  |  |
| Apolipoprotein A1 [g/L] | 0.048 (-0.348, 0.445) | 0.062 (-0.331, 0.454) | 0.010 (-.384, 0.405) | -.0053 (-.399, 0.389) | -.033 (-.444, 0.379) | -.026 (-.437, 0.386) |
| Apolipoprotein B [g/L] | 0.278 (-0.092, 0.648) | 0.270 (-0.089, 0.629) | 0.186 (-.174, 0.546) | 0.169 (-.188, 0.527) | 0.270 (-.101, 0.642) | 0.272 (-.099, 0.644) |
| Ratio of Apo B/Apo A1 | 0.239 (-0.118, 0.595) | 0.222 (-0.117, 0.561) | 0.176 (-.165, 0.517) | 0.170 (-.170, 0.511) | 0.288 (-.063, 0.639) | 0.285 (-.066, 0.637) |
| **Amino acids** |  |  |  |  |  |  |
| Alanine [µmol/L] | 0.312 (-0.071, 0.696) | 0.307 (-0.072, 0.686) | 0.299 (-.082, 0.679) | 0.289 (-.091, 0.669) | 0.316 (-.081, 0.714) | 0.303 (-.093, 0.699) |
| Glutamine [µmol/L] | -0.162 (-0.542, 0.219) | -0.158 (-0.539, 0.222) | -.196 (-.579, 0.187) | -.213 (-.594, 0.169) | -.214 (-.612, 0.185) | -.189 (-.583, 0.204) |
| Glycine [µmol/L] | -0.051 (-0.501, 0.399) | -0.0544 (-0.505, 0.396) | -.151 (-.602, 0.299) | -.169 (-.618, 0.279) | -.093 (-.564, 0.377) | -.128 (-.595, 0.338) |
| Histidine [µmol/L] | -0.399 (-0.781, -0.0163) | -0.398 (-0.779, -0.018) | -.317 (-.698, 0.064) | -.314 (-.696, 0.068) | -.392 (-.791, 0.0071) | -.377 (-.776, 0.021) |
| **Branched-chain amino acids** |  |  |  |  |  |  |
| Isoleucine [µmol/L] | -0.164 (-0.511, 0.184) | -0.179 (-0.514, 0.156) | -.188 (-.525, 0.149) | -.196 (-.533, 0.141) | -.122 (-.475, 0.230) | -.109 (-.460, 0.241) |
| Leucine [µmol/L] | 0.028 (-0.321, 0.377) | 0.014 (-0.326, 0.353) | 0.0028 (-.338, 0.344) | -.014 (-.354, 0.325) | 0.028 (-.327, 0.383) | 0.014 (-.339, 0.367) |
| Valine [µmol/L] | -0.031 (-0.396, 0.334) | -0.046 (-0.403, 0.311) | -.037 (-.396, 0.321) | -.058 (-.415, 0.299) | -.008 (-.382, 0.366) | -.0089 (-.379, 0.361) |
| **Aromatic amino acids** |  |  |  |  |  |  |
| Phenylalanine [µmol/L] | 0.357 (-0.034, 0.748) | 0.345 (-0.035, 0.725) | 0.276 (-.104, 0.657) | 0.249 (-.128, 0.627) | 0.197 (-.196, 0.589) | 0.151 (-.236, 0.539) |
| Tyrosine [µmol/L] | -0.311 (-0.685, 0.062) | -0.322 (-0.691, 0.048) | -.346 (-.717, 0.025) | -.355 (-.726, 0.016) | -.355 (-.742, 0.031) | -.329 (-.713, 0.053) |
| **Ketone bodies** |  |  |  |  |  |  |
| Acetoacetate [µmol/L] | -0.354 (-0.759, 0.052) | -0.354 (-0.759, 0.052) | -.404 (-.814, 0.0050) | -.404 (-.814, 0.0053) | -.429 (-.855, -.0023) | -.425 (-.850, -.00039) |
| Acetate [µmol/L] | 0.024 (-0.355, 0.404) | 0.025 (-0.356, 0.405) | -.0029 (-.383, 0.377) | -.019 (-.399, 0.360) | -.049 (-.444, 0.346) | -.055 (-.449, 0.339) |
| beta-hydroxybutyrate [µmol/L] | -0.359 (-0.782, 0.063) | -0.359 (-0.781, 0.063) | -.421 (-.842, 0.0009) | -.433 (-.855, -.0117) | -.423 (-.861, 0.015) | -.452 (-.889, -.015) |
| **Sphingolipids** |  |  |  |  |  |  |
| Sphingomyelin | 0.197 (-0.207, 0.601) | 0.202 (-0.201, 0.604) | 0.114 (-.289, 0.517) | 0.090 (-.309, 0.491) | 0.115 (-.303, 0.534) | 0.117 (-.302, 0.536) |
| Total choline | 0.259 (-0.142, 0.661) | 0.265 (-0.135, 0.665) | 0.191 (-.209, 0.593) | 0.174 (-.226, 0.574) | 0.185 (-.233, 0.603) | 0.190 (-.228, 0.608) |
| **Glycolysis related metabolites** |  |  |  |  |  |  |
| Citrate [µmol/L] | 0.152 (-0.232, 0.535) | 0.155 (-0.229, 0.538) | 0.150 (-.236, 0.536) | 0.138 (-.247, 0.524) | 0.042 (-.359, 0.443) | 0.047 (-.350, 0.445) |
| Glucose [mmol/L] | -0.092 (-0.502, 0.318) | -0.094 (-0.504, 0.317) | -.159 (-.571, 0.252) | -.194 (-.600, 0.213) | -.188 (-.615, 0.238) | -.166 (-.589, 0.258) |
| Glycerol [mmol/L] | 0.251 (-0.141, 0.644) | 0.257 (-0.134, 0.649) | 0.286 (-.107, 0.679) | 0.298 (-.094, 0.691) | 0.293 (-.118, 0.704) | 0.341 (-.069, 0.751) |
| Lactate [mmol/L] | -0.115 (-0.502, 0.271) | -0.122 (-0.506, 0.262) | -.113 (-.499, 0.272) | -.123 (-.509, 0.264) | -.139 (-.542, 0.263) | -.176 (-.571, 0.218) |
| Pyruvate [mmol/L] | -0.104 (-0.489, 0.281) | -0.096 (-0.479, 0.287) | -.101 (-.486, 0.285) | -.085 (-.469, 0.300) | -.064 (-.466, 0.338) | -.046 (-.448, 0.356) |
| **Fatty acids** |  |  |  |  |  |  |
| Total fatty acids [mmol/L] | 0.295 (-0.099, 0.689) | 0.293 (-0.097, 0.683) | 0.221 (-.169, 0.612) | 0.207 (-.182, 0.596) | 0.236 (-.171, 0.643) | 0.240 (-.167, 0.647) |
| Monounsaturated fatty acids [mmol/L] | 0.272 (-0.108, 0.652) | 0.265 (-0.106, 0.636) | 0.205 (-.168, 0.578) | 0.197 (-.175, 0.569) | 0.243 (-.144, 0.630) | 0.246 (-.142, 0.633) |
| Saturated fatty acids [mmol/L] | 0.367 (-0.032, 0.766) | 0.367 (-0.028, 0.761) | 0.285 (-.110, 0.681) | 0.272 (-.122, 0.666) | 0.288 (-.123, 0.699) | 0.292 (-.119, 0.704) |
| **Inflammation** |  |  |  |  |  |  |
| Alpha-1-acid glycoprotein [mmol/L] | 0.019 (-0.363, 0.400) | 0.0065 (-0.365, 0.378) | -.047 (-.419, 0.324) | -.076 (-.443, 0.292) | -.065 (-.449, 0.319) | -.062 (-.444, 0.321) |
| **Fluid balance** |  |  |  |  |  |  |
| Creatinine [µmol/L] | -0.074 (-0.325, 0.178) | -0.074 (-0.325, 0.178) | -.048 (-.301, 0.205) | -.059 (-.312, 0.194) | -.083 (-.348, 0.181) | -.073 (-.338, 0.191) |
| Albumin [standardized concentration units] | -0.341 (-0.699, 0.017) | -0.339 (-0.697, 0.019) | -.407 (-.766, -.048) | -.441 (-.795, -.087) | -.449 (-.819, -.078) | -.453 (-.819, -.087) |

**Specific pesticides use (PEU)**

**Table S54. Multivariable regression analyses of specific pesticides use with total lipoprotein subclasses with standardised metabolomic biomarkers in men**

| **Specific pesticides, β (95% CI), Males** | | | | | | |
| --- | --- | --- | --- | --- | --- | --- |
| **Total lipoprotein** | **Unadjusted** | **+ BMI** | **+ SEP** | **+ season of pesticide use** | **+ alcohol consumption** | **+ latitude of residence** |
| XXL_VLDL_P | -0.094 (-0.337, 0.149) | -0.015 (-0.251, 0.221) | -.021 (-.262, 0.219) | -.019 (-.259, 0.221) | -.0029 (-.247, 0.241) | -.0041 (-.249, 0.240) |
| XL_VLDL_P | -0.134 (-0.376, 0.107) | -0.072 (-0.308, 0.164) | -.077 (-.318, 0.163) | -.076 (-.317, 0.164) | -.053 (-.297, 0.192) | -.049 (-.294, 0.195) |
| L_VLDL_P | -0.098 (-0.335, 0.139) | -0.038 (-0.269, 0.193) | -.010 (-.246, 0.225) | -.012 (-.247, 0.223) | 0.019 (-.220, 0.258) | 0.025 (-.215, 0.264) |
| M_VLDL_P | -0.072 (-0.303, 0.159) | -0.007 (-0.230, 0.216) | 0.010 (-.217, 0.238) | 0.010 (-.218, 0.238) | 0.023 (-.209, 0.255) | 0.027 (-.205, 0.258) |
| S_VLDL_P | -0.122 (-0.339, 0.095) | -0.054 (-0.264, 0.155) | -.050 (-.264, 0.164) | -.052 (-.266, 0.162) | -.046 (-.264, 0.172) | -.054 (-.272, 0.164) |
| XS_VLDL_P | -0.135 (-0.365, 0.095) | -0.074 (-0.299, 0.152) | -.109 (-.338, 0.121) | -.112 (-.342, 0.117) | -.119 (-.353, 0.113) | -.137 (-.369, 0.095) |
| IDL_P | 0.047 (-0.182, 0.276) | 0.053 (-0.179, 0.284) | 0.070 (-.166, 0.307) | 0.078 (-.159, 0.314) | 0.103 (-.138, 0.344) | 0.111 (-.130, 0.352) |
| L_LDL_P | -0.059 (-0.298, 0.180) | -0.016 (-0.254, 0.223) | -.079 (-.322, 0.163) | -.086 (-.328, 0.155) | -.102 (-.347, 0.143) | -.117 (-.359, 0.126) |
| M_LDL_P | -0.051 (-0.287, 0.184) | -0.0095 (-0.244, 0.225) | -.067 (-.306, 0.171) | -.074 (-.312, 0.164) | -.090 (-.332, 0.151) | -.104 (-.343, 0.136) |
| S_LDL_P | -0.064 (-0.301, 0.173) | -0.024 (-0.259, 0.212) | -.080 (-.320, 0.159) | -.087 (-.326, 0.152) | -.098 (-.341, 0.144) | -.111 (-.352, 0.129) |
| XL_HDL_P | 0.063 (-0.173, 0.299) | 0.013 (-0.218, 0.243) | -.018 (-.253, 0.217) | -.017 (-.252, 0.218) | 0.012 (-.228, 0.251) | -.00087 (-.239, 0.238) |
| L_HDL_P | -0.015 (-0.250, 0.221) | -0.076 (-0.305, 0.153) | -.101 (-.334, 0.132) | -.106 (-.339, 0.127) | -.063 (-.299, 0.172) | -.057 (-.292, 0.178) |
| M_HDL_P | -0.200 (-0.432, 0.032) | -0.224 (-0.457, 0.0086) | -.219 (-.457, 0.017) | -.226 (-.462, 0.010) | -.175 (-.411, 0.061) | -.155 (-.388, 0.077) |
| S_HDL_P | -0.188 (-0.424, 0.047) | -0.179 (-0.417, 0.058) | -.204 (-.444, 0.037) | -.213 (-.452, 0.027) | -.179 (-.419, 0.059) | -.177 (-.414, 0.060) |

**Table S55. Multivariable regression analyses of specific pesticides use with total lipoprotein subclasses with standardised metabolomic biomarkers in women**

| **Specific pesticides, β (95% CI), Females** | | | | |  |  |
| --- | --- | --- | --- | --- | --- | --- |
| **Total lipoprotein** | **Unadjusted** | **+ BMI** | **+ SEP** | **+ season of pesticide use** | **+ alcohol consumption** | **+ latitude of residence** |
| XXL_VLDL_P | 0.333 (0.059, 0.607) | 0.269 (0.0075, 0.532) | 0.26 (-.0091, 0.519) | 0.243 (-.024, 0.511) | 0.271 (0.00091, 0.542) | 0.276 (0.0062, 0.546) |
| XL_VLDL_P | 0.232 (-0.059, 0.524) | 0.165 (-0.117, 0.447) | 0.139 (-.145, 0.423) | 0.118 (-.169, 0.405) | 0.138 (-.152, 0.429) | 0.159 (-.130, 0.449) |
| L_VLDL_P | 0.269 (-0.027, 0.566) | 0.194 (-0.090, 0.479) | 0.192 (-.095, 0.479) | 0.173 (-.118, 0.463) | 0.211 (-.084, 0.506) | 0.228 (-.067, 0.523) |
| M_VLDL_P | 0.286 (0.014, 0.557) | 0.209 (-0.051, 0.468) | 0.182 (-.079, 0.443) | 0.168 (-.096, 0.432) | 0.198 (-.069, 0.466) | 0.217 (-.049, 0.484) |
| S_VLDL_P | 0.324 (0.036, 0.612) | 0.246 (-0.029, 0.521) | 0.203 (-.075, 0.481) | 0.169 (-.110, 0.449) | 0.185 (-.098, 0.467) | 0.184 (-.099, 0.467) |
| XS_VLDL_P | 0.343 (0.045, 0.642) | 0.285 (-0.0057, 0.576) | 0.215 (-.078, 0.508) | 0.184 (-.111, 0.479) | 0.197 (-.099, 0.495) | 0.192 (-.106, 0.489) |
| IDL_P | 0.020 (-0.324, 0.364) | 0.042 (-0.302, 0.386) | 0.042 (-.305, 0.389) | 0.038 (-.313, 0.390) | 0.054 (-.300, 0.408) | 0.055 (-.299, 0.409) |
| L_LDL_P | 0.224 (-0.076, 0.524) | 0.189 (-0.106, 0.486) | 0.099 (-.198, 0.396) | 0.068 (-.231, 0.366) | 0.096 (-.205, 0.396) | 0.092 (-.209, 0.393) |
| M_LDL_P | 0.226 (-0.074, 0.526) | 0.189 (-0.107, 0.486) | 0.103 (-.194, 0.400) | 0.073 (-.225, 0.371) | 0.104 (-.196, 0.405) | 0.100 (-.199, 0.400) |
| S_LDL_P | 0.226 (-0.075, 0.528) | 0.189 (-0.108, 0.489) | 0.107 (-.192, 0.406) | 0.075 (-.224, 0.375) | 0.107 (-.196, 0.409) | 0.102 (-.200, 0.405) |
| XL_HDL_P | 0.0017 (-0.262, 0.265) | 0.060 (-0.197, 0.318) | 0.046 (-.214, 0.306) | -.00013 (-.263, 0.263) | -.016 (-.282, 0.249) | -.041 (-.304, 0.222) |
| L_HDL_P | -0.333 (-0.595, -0.069) | -0.260 (-0.514, -0.0059) | -.282 (-.539, -.026) | -.327 (-.586, -.067) | -.365 (-.628, -.103) | -.365 (-.628, -.103) |
| M_HDL_P | -0.401 (-0.704, -0.098) | -0.371 (-0.674, -0.068) | -.395 (-.701, -.089) | -.415 (-.724, -.106) | -.461 (-.773, -.149) | -.437 (-.747, -.127) |
| S_HDL_P | -0.317 (-0.633, -0.00038) | -0.325 (-0.641, -0.0087) | -.387 (-.705, -.069) | -.408 (-.728, -.088) | -.440 (-.764, -.116) | -.432 (-.754, -.111) |

**Table S56. Multivariable regression analyses of specific pesticides use with triglyceride subclasses with standardised metabolomic biomarkers in men**

| **Specific pesticides, β (95% CI), Males** | | | | | | |
| --- | --- | --- | --- | --- | --- | --- |
| **Triglycerides** | **Unadjusted** | **+ BMI** | **+ SEP** | **+ season of pesticide use** | **+ alcohol consumption** | **+ latitude of residence** |
| XXL_VLDL_TG | -0.096 (-0.336, 0.144) | -0.019 (-0.252, 0.214) | -.025 (-.262, 0.211) | -.023 (-.259, 0.213) | -.0050 (-.245, 0.235) | -.0059 (-.247, 0.235) |
| XL_VLDL_TG | -0.145 (-0.379, 0.089) | -0.089 (-0.318, 0.140) | -.090 (-.324, 0.143) | -.089 (-.323, 0.143) | -.061 (-.299, 0.176) | -.056 (-.293, 0.182) |
| L_VLDL_TG | -0.095 (-0.328, 0.138) | -0.041 (-0.268, 0.187) | -.0069 (-.239, 0.225) | -.0085 (-.239, 0.223) | 0.027 (-.208, 0.262) | 0.035 (-.200, 0.269) |
| M_VLDL_TG | -0.073 (-0.295, 0.149) | -0.015 (-0.230, 0.199) | 0.0087 (-.210, 0.228) | 0.0087 (-.210, 0.228) | 0.025 (-.198, 0.248) | 0.032 (-.191, 0.255) |
| S_VLDL_TG | -0.093 (-0.305, 0.118) | -0.034 (-0.238, 0.171) | -.016 (-.224, 0.192) | -.017 (-.226, 0.191) | -.0057 (-.218, 0.206) | -.0087 (-.221, 0.203) |
| XS_VLDL_TG | -0.122 (-0.338, 0.094) | -0.060 (-0.269, 0.149) | -.062 (-.275, 0.151) | -.066 (-.279, 0.147) | -.057 (-.274, 0.159) | -.071 (-.286, 0.145) |
| VLDL_TG | -0.093 (-0.321, 0.135) | -0.029 (-0.249, 0.191) | -.011 (-.236, 0.213) | -.012 (-.237, 0.213) | 0.0065 (-.222, 0.235) | 0.0091 (-.219, 0.238) |
| IDL_TG | -0.103 (-0.325, 0.120) | -0.048 (-0.266, 0.169) | -.074 (-.296, 0.148) | -.080 (-.301, 0.141) | -.073 (-.298, 0.152) | -.096 (-.318, 0.127) |
| L_LDL_TG | -0.085 (-0.311, 0.142) | -0.032 (-0.255, 0.191) | -.075 (-.301, 0.152) | -.082 (-.307, 0.143) | -.076 (-.305, 0.153) | -.102 (-.328, 0.124) |
| M_LDL_TG | -0.043 (-0.271, 0.184) | 0.005 (-0.219, 0.229) | -.039 (-.267, 0.190) | -.047 (-.274, 0.181) | -.041 (-.272, 0.189) | -.067 (-.294, 0.161) |
| S_LDL_TG | -0.087 (-0.313, 0.138) | -0.029 (-0.251, 0.191) | -.061 (-.285, 0.164) | -.067 (-.291, 0.157) | -.059 (-.286, 0.169) | -.078 (-.304, 0.148) |
| LDL_TG | -0.078 (-0.305, 0.149) | -0.025 (-0.248, 0.198) | -.066 (-.293, 0.161) | -.074 (-.299, 0.152) | -.067 (-.297, 0.162) | -.092 (-.318, 0.135) |
| XL_HDL_TG | 0.137 (-0.101, 0.376) | 0.163 (-0.076, 0.403) | 0.115 (-.128, 0.358) | 0.114 (-.129, 0.357) | 0.114 (-.133, 0.361) | 0.096 (-.149, 0.341) |
| L_HDL_TG | 0.076 (-0.164, 0.316) | 0.072 (-0.169, 0.315) | 0.041 (-.205, 0.288) | 0.032 (-.213, 0.277) | 0.040 (-.209, 0.289) | 0.035 (-.211, 0.282) |
| M_HDL_TG | -0.060 (-0.294, 0.174) | 0.0089 (-0.221, 0.239) | 0.0086 (-.226, 0.243) | 0.0061 (-.228, 0.241) | 0.029 (-.208, 0.267) | 0.028 (-.209, 0.265) |
| S_HDL_TG | 0.0079 (-0.204, 0.219) | 0.078 (-0.126, 0.282) | 0.050 (-.157, 0.257) | 0.047 (-.161, 0.254) | 0.052 (-.158, 0.263) | 0.032 (-.177, 0.241) |
| HDL_TG | 0.027 (-0.204, 0.258) | 0.080 (-0.149, 0.309) | 0.051 (-.182, 0.285) | 0.046 (-.187, 0.279) | 0.061 (-.176, 0.298) | 0.047 (-.188, 0.282) |
| Serum_TG | -0.103 (-0.341, 0.134) | -0.034 (-0.263, 0.195) | -.029 (-.262, 0.204) | -.031 (-.264, 0.202) | -.011 (-.248, 0.226) | -.015 (-.252, 0.222) |

**Table S57. Multivariable regression analyses of specific pesticides use with triglyceride subclasses with standardised metabolomic biomarkers in women**

| **Specific pesticides, β (95% CI), Females** | | | | | | |
| --- | --- | --- | --- | --- | --- | --- |
| **Triglycerides** | **Unadjusted** | **+ BMI** | **+ SEP** | **+ season of pesticide use** | **+ alcohol consumption** | **+ latitude of residence** |
| XXL_VLDL_TG | 0.331 (0.058, 0.604) | 0.268 (0.0069, 0.529) | 0.255 (-.0081, 0.518) | 0.246 (-.019, 0.512) | 0.275 (0.0056, 0.544) | 0.279 (0.010, 0.548) |
| XL_VLDL_TG | 0.175 (-0.123, 0.474) | 0.111 (-0.179, 0.400) | 0.091 (-.201, 0.382) | 0.066 (-.228, 0.361) | 0.081 (-.217, 0.379) | 0.104 (-.193, 0.401) |
| L_VLDL_TG | 0.215 (-0.085, 0.514) | 0.141 (-0.147, 0.429) | 0.143 (-.148, 0.433) | 0.124 (-.169, 0.419) | 0.167 (-.133, 0.466) | 0.188 (-.111, 0.486) |
| M_VLDL_TG | 0.253 (-0.030, 0.535) | 0.177 (-0.095, 0.449) | 0.162 (-.112, 0.436) | 0.151 (-.125, 0.427) | 0.187 (-.093, 0.467) | 0.209 (-.070, 0.489) |
| S_VLDL_TG | 0.335 (0.029, 0.640) | 0.257 (-0.038, 0.551) | 0.230 (-.068, 0.528) | 0.199 (-.100, 0.499) | 0.206 (-.097, 0.509) | 0.209 (-.095, 0.512) |
| XS_VLDL_TG | 0.338 (0.026, 0.649) | 0.267 (-0.034, 0.568) | 0.217 (-.087, 0.521) | 0.171 (-.134, 0.477) | 0.159 (-.149, 0.468) | 0.153 (-.156, 0.462) |
| VLDL_TG | 0.292 (0.0087, 0.576) | 0.211 (-0.059, 0.482) | 0.189 (-.084, 0.463) | 0.167 (-.109, 0.443) | 0.181 (-.098, 0.459) | 0.197 (-.082, 0.476) |
| IDL_TG | 0.338 (0.025, 0.652) | 0.288 (-0.019, 0.595) | 0.223 (-.086, 0.532) | 0.169 (-.142, 0.479) | 0.144 (-.170, 0.458) | 0.128 (-.186, 0.442) |
| L_LDL_TG | 0.294 (-0.024, 0.612) | 0.254 (-0.059, 0.567) | 0.179 (-.135, 0.495) | 0.122 (-.195, 0.438) | 0.092 (-.228, 0.412) | 0.073 (-.247, 0.392) |
| M_LDL_TG | 0.284 (-0.029, 0.596) | 0.246 (-0.062, 0.554) | 0.187 (-.123, 0.498) | 0.135 (-.178, 0.447) | 0.104 (-.212, 0.419) | 0.084 (-.232, 0.399) |
| S_LDL_TG | 0.289 (-0.024, 0.602) | 0.238 (-0.068, 0.545) | 0.185 (-.124, 0.494) | 0.136 (-.175, 0.447) | 0.111 (-.203, 0.425) | 0.095 (-.218, 0.409) |
| LDL_TG | 0.292 (-0.023, 0.608) | 0.251 (-0.059, 0.561) | 0.181 (-.131, 0.493) | 0.126 (-.188, 0.439) | 0.096 (-.221, 0.413) | 0.077 (-.239, 0.393) |
| XL_HDL_TG | 0.053 (-0.253, 0.358) | 0.067 (-0.238, 0.372) | -.0044 (-.312, 0.303) | -.061 (-.371, 0.249) | -.077 (-.390, 0.236) | -.082 (-.396, 0.231) |
| L_HDL_TG | -0.199 (-0.498, 0.099) | -0.168 (-0.465, 0.129) | -.243 (-.542, 0.056) | -.295 (-.596, 0.0068) | -.328 (-.633, -.022) | -.337 (-.642, -.032) |
| M_HDL_TG | 0.183 (-0.118, 0.483) | 0.136 (-0.160, 0.432) | 0.087 (-.211, 0.386) | 0.051 (-.251, 0.352) | 0.026 (-.281, 0.332) | 0.033 (-.273, 0.339) |
| S_HDL_TG | 0.325 (0.0033, 0.647) | 0.262 (-0.052, 0.576) | 0.203 (-.114, 0.520) | 0.151 (-.168, 0.471) | 0.131 (-.193, 0.456) | 0.109 (-.215, 0.432) |
| HDL_TG | 0.077 (-0.239, 0.393) | 0.056 (-0.258, 0.369) | -.023 (-.339, 0.293) | -.085 (-.403, 0.232) | -.116 (-.438, 0.206) | -.124 (-.446, 0.198) |
| Serum_TG | 0.297 (0.016, 0.578) | 0.224 (-0.045, 0.494) | 0.183 (-.089, 0.455) | 0.145 (-.129, 0.419) | 0.141 (-.137, 0.418) | 0.147 (-.130, 0.425) |

**Table S58. Multivariable regression analyses of specific pesticides use with phospholipid subclasses with standardised metabolomic biomarkers in men**

| **Specific pesticides, β (95% CI), Males** | | | | | | |
| --- | --- | --- | --- | --- | --- | --- |
| **Phospholipids** | **Unadjusted** | **+ BMI** | **+ SEP** | **+ season of pesticide use** | **+ alcohol consumption** | **+ latitude of residence** |
| XXL_VLDL_PL | -0.139 (-0.371, 0.094) | -0.069 (-0.297, 0.158) | -.069 (-.301, 0.162) | -.068 (-.299, 0.164) | -.045 (-.281, 0.189) | -.043 (-.278, 0.192) |
| XL_VLDL_PL | -0.108 (-0.347, 0.131) | -0.044 (-0.278, 0.190) | -.054 (-.293, 0.184) | -.053 (-.292, 0.186) | -.032 (-.275, 0.211) | -.032 (-.275, 0.212) |
| L_VLDL_PL | -0.073 (-0.309, 0.162) | -0.012 (-0.241, 0.217) | 0.011 (-.222, 0.245) | 0.0094 (-.224, 0.243) | 0.038 (-.199, 0.275) | 0.042 (-.195, 0.279) |
| M_VLDL_PL | -0.077 (-0.312, 0.159) | -0.009 (-0.236, 0.218) | 0.0019 (-.229, 0.233) | 0.0012 (-.230, 0.233) | 0.012 (-.224, 0.248) | 0.014 (-.222, 0.249) |
| S_VLDL_PL | -0.146 (-0.366, 0.074) | -0.078 (-0.292, 0.135) | -.074 (-.291, 0.144) | -.076 (-.294, 0.142) | -.064 (-.286, 0.157) | -.074 (-.295, 0.147) |
| XS_VLDL_PL | -0.123 (-0.360, 0.114) | -0.075 (-0.311, 0.160) | -.114 (-.354, 0.125) | -.119 (-.359, 0.119) | -.132 (-.376, 0.111) | -.147 (-.388, 0.095) |
| IDL_PL | -0.060 (-0.307, 0.187) | -0.021 (-0.268, 0.226) | -.089 (-.339, 0.162) | -.095 (-.345, 0.155) | -.115 (-.369, 0.138) | -.129 (-.381, 0.123) |
| L_LDL_PL | -0.061 (-0.303, 0.180) | -0.022 (-0.264, 0.219) | -.088 (-.334, 0.157) | -.095 (-.339, 0.150) | -.108 (-.356, 0.140) | -.119 (-.366, 0.127) |
| M_LDL_PL | -0.079 (-0.323, 0.165) | -0.033 (-0.275, 0.209) | -.097 (-.343, 0.149) | -.104 (-.349, 0.141) | -.109 (-.358, 0.139) | -.122 (-.369, 0.125) |
| S_LDL_PL | -0.068 (-0.308, 0.171) | -0.029 (-0.268, 0.209) | -.093 (-.335, 0.149) | -.099 (-.341, 0.142) | -.095 (-.340, 0.149) | -.107 (-.349, 0.136) |
| XL_HDL_PL | 0.107 (-0.149, 0.364) | 0.047 (-0.200, 0.294) | 0.041 (-.210, 0.293) | 0.042 (-.210, 0.294) | 0.064 (-.191, 0.319) | 0.057 (-.198, 0.311) |
| L_HDL_PL | -0.046 (-0.284, 0.193) | -0.104 (-0.337, 0.128) | -.124 (-.361, 0.112) | -.129 (-.366, 0.107) | -.084 (-.322, 0.155) | -.073 (-.311, 0.165) |
| M_HDL_PL | -0.211 (-0.443, 0.022) | -0.235 (-0.468, -0.0018) | -.236 (-.473, 0.00043) | -.243 (-.479, -.0065) | -.187 (-.423, 0.048) | -.169 (-.402, 0.063) |
| S_HDL_PL | -0.221 (-0.446, 0.0039) | -0.227 (-0.454, -0.0003) | -.229 (-.459, 0.00025) | -.235 (-.465, -.0056) | -.187 (-.416, 0.041) | -.172 (-.398, 0.054) |

**Table S59. Multivariable regression analyses of specific pesticides use with phospholipid subclasses with standardised metabolomic biomarkers in women**

| **Specific pesticides, β (95% CI), Females** | | | | | | |
| --- | --- | --- | --- | --- | --- | --- |
| **Phospholipids** | **Unadjusted** | **+ BMI** | **+ SEP** | **+ season of pesticide use** | **+ alcohol consumption** | **+ latitude of residence** |
| XXL_VLDL_PL | 0.228 (-0.054, 0.510) | 0.174 (-0.099, 0.448) | 0.173 (-.103, 0.449) | 0.157 (-.122, 0.436) | 0.165 (-.118, 0.448) | 0.179 (-.103, 0.463) |
| XL_VLDL_PL | 0.246 (-0.055, 0.546) | 0.181 (-0.111, 0.472) | 0.147 (-.146, 0.439) | 0.127 (-.169, 0.423) | 0.142 (-.159, 0.444) | 0.161 (-.140, 0.461) |
| L_VLDL_PL | 0.279 (-0.019, 0.576) | 0.204 (-0.082, 0.489) | 0.194 (-.094, 0.482) | 0.172 (-.119, 0.464) | 0.203 (-.094, 0.499) | 0.218 (-.079, 0.514) |
| M_VLDL_PL | 0.297 (0.021, 0.573) | 0.219 (-0.044, 0.482) | 0.183 (-.082, 0.449) | 0.165 (-.103, 0.433) | 0.189 (-.082, 0.461) | 0.206 (-.065, 0.477) |
| S_VLDL_PL | 0.331 (0.027, 0.634) | 0.255 (-0.037, 0.547) | 0.213 (-.081, 0.508) | 0.174 (-.123, 0.470) | 0.178 (-.122, 0.477) | 0.174 (-.126, 0.473) |
| XS_VLDL_PL | 0.303 (0.0016, 0.604) | 0.259 (-0.037, 0.555) | 0.184 (-.114, 0.482) | 0.158 (-.141, 0.458) | 0.172 (-.131, 0.474) | 0.171 (-.131, 0.473) |
| IDL_PL | 0.211 (-0.092, 0.513) | 0.182 (-0.118, 0.481) | 0.089 (-.212, 0.389) | 0.058 (-.244, 0.359) | 0.081 (-.223, 0.385) | 0.080 (-.224, 0.384) |
| L_LDL_PL | 0.168 (-0.129, 0.464) | 0.137 (-0.156, 0.431) | 0.045 (-.249, 0.339) | 0.019 (-.277, 0.314) | 0.048 (-.250, 0.346) | 0.048 (-.249, 0.346) |
| M_LDL_PL | 0.204 (-0.097, 0.504) | 0.165 (-0.131, 0.461) | 0.065 (-.232, 0.362) | 0.033 (-.265, 0.331) | 0.063 (-.238, 0.364) | 0.058 (-.242, 0.359) |
| S_LDL_PL | 0.186 (-0.113, 0.485) | 0.153 (-0.142, 0.448) | 0.059 (-.237, 0.355) | 0.019 (-.278, 0.317) | 0.042 (-.259, 0.343) | 0.037 (-.264, 0.339) |
| XL_HDL_PL | -0.030 (-0.247, 0.187) | 0.028 (-0.182, 0.238) | 0.021 (-.191, 0.233) | -.012 (-.227, 0.202) | -.029 (-.245, 0.187) | -.042 (-.257, 0.173) |
| L_HDL_PL | -0.361 (-0.625, -0.096) | -0.290 (-0.547, -0.033) | -.309 (-.568, -.049) | -.351 (-.614, -.089) | -.392 (-.657, -.127) | -.387 (-.653, -.122) |
| M_HDL_PL | -0.399 (-0.707, -0.091) | -0.366 (-0.674, -0.059) | -.394 (-.704, -.084) | -.425 (-.738, -.111) | -.475 (-.792, -.157) | -.453 (-.768, -.138) |
| S_HDL_PL | -0.601 (-0.909, -0.293) | -0.593 (-0.901, -0.284) | -.602 (-.913, -.291) | -.608 (-.922, -.293) | -.662 (-.979, -.344) | -.643 (-.959, -.328) |

**Table S60. Multivariable regression analyses of specific pesticides use with cholesterol esters subclasses with standardised metabolomic biomarkers in men**

| **Specific pesticides, β (95% CI), Males** | | | | | | |
| --- | --- | --- | --- | --- | --- | --- |
| **Cholesterol esters** | **Unadjusted** | **+ BMI** | **+ SEP** | **+ season of pesticide use** | **+ alcohol consumption** | **+ latitude of residence** |
| XXL_VLDL_CE | 0.019 (-0.204, 0.242) | 0.089 (-0.129, 0.308) | 0.091 (-.131, 0.313) | 0.094 (-.128, 0.316) | 0.084 (-.142, 0.310) | 0.078 (-.148, 0.304) |
| XL_VLDL_CE | -0.052 (-0.282, 0.178) | 0.012 (-0.213, 0.237) | 0.00068 (-.228, 0.229) | 0.0034 (-.225, 0.232) | 0.00049 (-.233, 0.234) | -.0043 (-.238, 0.229) |
| L_VLDL_CE | -0.048 (-0.282, 0.186) | 0.012 (-0.218, 0.243) | 0.049 (-.186, 0.284) | 0.048 (-.185, 0.282) | 0.051 (-.186, 0.287) | 0.048 (-.189, 0.285) |
| M_VLDL_CE | -0.015 (-0.254, 0.224) | 0.058 (-0.174, 0.289) | 0.055 (-.181, 0.291) | 0.055 (-.181, 0.292) | 0.047 (-.194, 0.287) | 0.040 (-.200, 0.280) |
| S_VLDL_CE | -0.104 (-0.326, 0.119) | -0.039 (-0.258, 0.179) | -.069 (-.292, 0.153) | -.072 (-.294, 0.151) | -.086 (-.313, 0.140) | -.101 (-.326, 0.125) |
| XS_VLDL_CE | -0.097 (-0.332, 0.139) | -0.043 (-0.277, 0.191) | -.099 (-.336, 0.139) | -.099 (-.337, 0.139) | -.117 (-.358, 0.124) | -.134 (-.374, 0.106) |
| IDL_CE | -0.076 (-0.312, 0.161) | -0.033 (-0.269, 0.203) | -.101 (-.341, 0.139) | -.106 (-.345, 0.134) | -.122 (-.365, 0.120) | -.134 (-.375, 0.107) |
| L_LDL_CE | -0.051 (-0.288, 0.187) | -0.0093 (-0.247, 0.228) | -.073 (-.314, 0.169) | -.079 (-.319, 0.162) | -.099 (-.343, 0.145) | -.111 (-.353, 0.132) |
| M_LDL_CE | -0.033 (-0.268, 0.203) | 0.0019 (-0.234, 0.238) | -.052 (-.292, 0.188) | -.058 (-.297, 0.181) | -.082 (-.325, 0.162) | -.091 (-.333, 0.151) |
| S_LDL_CE | -0.048 (-0.291, 0.195) | -0.019 (-0.263, 0.225) | -.070 (-.319, 0.178) | -.076 (-.324, 0.172) | -.098 (-.351, 0.154) | -.108 (-.359, 0.143) |
| XL_HDL_CE | 0.055 (-0.189, 0.299) | 0.025 (-0.217, 0.267) | -.016 (-.262, 0.231) | -.013 (-.259, 0.234) | 0.012 (-.239, 0.264) | -.0077 (-.258, 0.242) |
| L_HDL_CE | 0.026 (-0.217, 0.269) | -0.040 (-0.275, 0.195) | -.066 (-.306, 0.173) | -.069 (-.309, 0.169) | -.029 (-.272, 0.214) | -.025 (-.268, 0.218) |
| M_HDL_CE | -0.155 (-0.391, 0.081) | -0.194 (-0.429, 0.041) | -.182 (-.421, 0.057) | -.187 (-.426, 0.052) | -.143 (-.382, 0.097) | -.119 (-.355, 0.117) |
| S_HDL_CE | -0.069 (-0.331, 0.193) | -0.066 (-0.331, 0.198) | -.099 (-.369, 0.169) | -.108 (-.377, 0.159) | -.113 (-.385, 0.159) | -.121 (-.392, 0.151) |
| EstC | -0.139 (-0.379, 0.100) | -0.110 (-0.352, 0.131) | -.166 (-.411, 0.079) | -.173 (-.417, 0.072) | -.169 (-.417, 0.079) | -.179 (-.425, 0.067) |

**Table S61. Multivariable regression analyses of specific pesticides use with cholesterol esters subclasses with standardised metabolomic biomarkers in women**

| **Specific pesticides, β (95% CI), Females** | | | | | | |
| --- | --- | --- | --- | --- | --- | --- |
| **Cholesterol esters** | **Unadjusted** | **+ BMI** | **+ SEP** | **+ season of pesticide use** | **+ alcohol consumption** | **+ latitude of residence** |
| XXL_VLDL_CE | 0.432 (0.129, 0.736) | 0.375 (0.080, 0.671) | 0.349 (0.051, 0.647) | 0.334 (0.033, 0.636) | 0.373 (0.068, 0.677) | 0.372 (0.068, 0.676) |
| XL_VLDL_CE | 0.377 (0.064, 0.691) | 0.315 (0.0087, 0.620) | 0.288 (-.0189, 0.595) | 0.275 (-.035, 0.585) | 0.313 (0.0013, 0.625) | 0.318 (0.0068, 0.629) |
| L_VLDL_CE | 0.448 (0.136, 0.760) | 0.379 (0.075, 0.682) | 0.367 (0.061, 0.674) | 0.355 (0.044, 0.665) | 0.390 (0.076, 0.705) | 0.385 (0.070, 0.699) |
| M_VLDL_CE | 0.407 (0.126, 0.689) | 0.338 (0.066, 0.611) | 0.286 (0.012, 0.560) | 0.268 (-.0095, 0.545) | 0.301 (0.021, 0.582) | 0.311 (0.031, 0.592) |
| S_VLDL_CE | 0.301 (0.0059, 0.595) | 0.235 (-0.051, 0.521) | 0.169 (-.119, 0.457) | 0.144 (-.146, 0.433) | 0.179 (-.112, 0.471) | 0.172 (-.119, 0.464) |
| XS_VLDL_CE | 0.308 (0.019, 0.597) | 0.260 (-0.024, 0.545) | 0.201 (-.086, 0.487) | 0.192 (-.097, 0.482) | 0.222 (-.068, 0.513) | 0.213 (-.077, 0.504) |
| IDL_CE | 0.221 (-0.074, 0.515) | 0.187 (-0.105, 0.478) | 0.101 (-.192, 0.394) | 0.079 (-.215, 0.374) | 0.111 (-.186, 0.408) | 0.111 (-.186, 0.408) |
| L_LDL_CE | 0.221 (-0.083, 0.524) | 0.186 (-0.114, 0.486) | 0.099 (-.202, 0.401) | 0.077 (-.226, 0.379) | 0.119 (-.186, 0.423) | 0.118 (-.187, 0.423) |
| M_LDL_CE | 0.209 (-0.093, 0.511) | 0.178 (-0.121, 0.478) | 0.106 (-.195, 0.407) | 0.088 (-.214, 0.389) | 0.133 (-.171, 0.436) | 0.134 (-.169, 0.437) |
| S_LDL_CE | 0.203 (-0.092, 0.497) | 0.174 (-0.118, 0.466) | 0.109 (-.186, 0.403) | 0.091 (-.204, 0.386) | 0.136 (-.161, 0.433) | 0.136 (-.161, 0.433) |
| XL_HDL_CE | 0.083 (-0.204, 0.369) | 0.116 (-0.169, 0.402) | 0.104 (-.185, 0.392) | 0.063 (-.229, 0.354) | 0.056 (-.240, 0.351) | 0.013 (-.277, 0.302) |
| L_HDL_CE | -0.298 (-0.542, -0.055) | -0.225 (-0.459, 0.0081) | -.238 (-.474, -.0021) | -.278 (-.516, -.039) | -.312 (-.553, -.071) | -.314 (-.555, -.074) |
| M_HDL_CE | -0.451 (-0.747, -0.156) | -0.416 (-0.710, -0.122) | -.423 (-.719, -.126) | -.419 (-.719, -.119) | -.458 (-.761, -.155) | -.432 (-.733, -.131) |
| S_HDL_CE | -0.043 (-0.316, 0.231) | -0.055 (-0.328, 0.218) | -.128 (-.401, 0.146) | -.141 (-.415, 0.133) | -.126 (-.404, 0.152) | -.129 (-.406, 0.147) |
| EstC | 0.106 (-0.193, 0.405) | 0.090 (-0.207, 0.387) | 0.0046 (-.294, 0.303) | -.033 (-.333, 0.266) | -.016 (-.320, 0.287) | -.019 (-.323, 0.285) |

**Table S62. Multivariable regression analyses of specific pesticides use with free cholesterol subclasses with standardised metabolomic biomarkers in men**

| **Specific pesticides, β (95% CI), Males** | | | | | | |
| --- | --- | --- | --- | --- | --- | --- |
| **Free cholesterol** | **Unadjusted** | **+ BMI** | **+ SEP** | **+ season of pesticide use** | **+ alcohol consumption** | **+ latitude of residence** |
| XXL_VLDL_FC | -0.086 (-0.319, 0.147) | -0.016 (-0.244, 0.211) | -.025 (-.256, 0.207) | -.022 (-.254, 0.209) | -.0063 (-.242, 0.229) | -.0068 (-.243, 0.229) |
| XL_VLDL_FC | -0.096 (-0.333, 0.141) | -0.028 (-0.259, 0.204) | -.047 (-.283, 0.189) | -.046 (-.283, 0.191) | -.032 (-.274, 0.209) | -.036 (-.277, 0.205) |
| L_VLDL_FC | -0.093 (-0.321, 0.135) | -0.036 (-0.259, 0.188) | -.011 (-.239, 0.217) | -.012 (-.239, 0.216) | 0.011 (-.222, 0.243) | 0.014 (-.218, 0.246) |
| M_VLDL_FC | -0.076 (-0.306, 0.154) | -0.012 (-0.235, 0.210) | -.000093 (-.227, 0.227) | -.00051 (-.228, 0.227) | 0.0097 (-.222, 0.241) | 0.011 (-.219, 0.242) |
| S_VLDL_FC | -0.138 (-0.354, 0.077) | -0.075 (-0.284, 0.135) | -.078 (-.292, 0.136) | -.081 (-.295, 0.133) | -.074 (-.292, 0.144) | -.084 (-.302, 0.133) |
| XS_VLDL_FC | -0.104 (-0.340, 0.133) | -0.060 (-0.296, 0.176) | -.096 (-.336, 0.144) | -.099 (-.339, 0.139) | -.115 (-.359, 0.129) | -.132 (-.3745, 0.111) |
| IDL_FC | -0.051 (-0.301, 0.199) | -.021 (-0.273, 0.230) | -.084 (-.339, 0.172) | -.089 (-.344, 0.166) | -.114 (-.373, 0.144) | -.128 (-.385, 0.129) |
| L_LDL_FC | -0.029 (-0.274, 0.217) | 0.0021 (-0.245, 0.249) | -.070 (-.321, 0.180) | -.076 (-.326, 0.173) | -.099 (-.353, 0.154) | -.113 (-.365, 0.138) |
| M_LDL_FC | -0.035 (-0.271, 0.201) | -0.001 (-0.238, 0.235) | -.072 (-.312, 0.168) | -.079 (-.318, 0.160) | -.094 (-.337, 0.149) | -.109 (-.350, 0.131) |
| S_LDL_FC | -0.032 (-0.273, 0.209) | 0.00035 (-0.241, 0.241) | -.073 (-.317, 0.172) | -.079 (-.323, 0.165) | -.090 (-.338, 0.158) | -.107 (-.353, 0.138) |
| XL_HDL_FC | 0.077 (-0.181, 0.335) | 0.036 (-0.216, 0.287) | -.0069 (-.263, 0.249) | -.0039 (-.260, 0.252) | 0.019 (-.242, 0.281) | 0.013 (-.248, 0.274) |
| L_HDL_FC | 0.033 (-0.221, 0.287) | -0.039 (-0.283, 0.206) | -.066 (-.315, 0.183) | -.069 (-.318, 0.180) | -.033 (-.286, 0.221) | -.030 (-.284, 0.223) |
| M_HDL_FC | -0.197 (-0.439, 0.046) | -0.231 (-0.473, 0.012) | -.230 (-.477, 0.016) | -.237 (-.483, 0.0089) | -.192 (-.439, 0.055) | -.174 (-.418, 0.069) |
| S_HDL_FC | -0.198 (-0.439, 0.043) | -0.203 (-0.446, 0.040) | -.227 (-.474, 0.019) | -.235 (-.480, 0.010) | -.195 (-.441, 0.051) | -.191 (-.434, 0.053) |
| Free cholesterol | -0.103 (-0.347, 0.139) | -0.077 (-0.322, 0.167) | -.140 (-.389, 0.108) | -.146 (-.394, 0.1018) | -.152 (-.404, 0.099) | -.163 (-.412, 0.087) |

**Table S63. Multivariable regression analyses of specific pesticides use with free cholesterol subclasses with standardised metabolomic biomarkers in women**

| **Specific pesticides, β (95% CI), Females** | | | | | | |
| --- | --- | --- | --- | --- | --- | --- |
| **Free cholesterol** | **Unadjusted** | **+ BMI** | **+ SEP** | **+ season of pesticide use** | **+ alcohol consumption** | **+ latitude of residence** |
| XXL_VLDL_FC | 0.239 (-0.048, 0.525) | 0.182 (-0.095, 0.459) | 0.164 (-.115, 0.444) | 0.142 (-.1409, 0.425) | 0.143 (-.144, 0.430) | 0.156 (-.131, 0.443) |
| XL_VLDL_FC | 0.256 (-0.042, 0.554) | 0.193 (-0.096, 0.483) | 0.154 (-.134, 0.442) | 0.137 (-.155, 0.429) | 0.159 (-.137, 0.455) | 0.169 (-.127, 0.466) |
| L_VLDL_FC | 0.251 (-0.061, 0.563) | 0.178 (-0.124, 0.479) | 0.170 (-.134, 0.474) | 0.144 (-.164, 0.452) | 0.161 (-.151, 0.474) | 0.177 (-.136, 0.489) |
| M_VLDL_FC | 0.278 (-0.0061, 0.562) | 0.202 (-0.071, 0.474) | 0.167 (-.108, 0.441) | 0.142 (-.136, 0.419) | 0.157 (-.124, 0.438) | 0.174 (-.107, 0.454) |
| S_VLDL_FC | 0.287 (-0.013, 0.587) | 0.215 (-0.074, 0.504) | 0.164 (-.128, 0.455) | 0.125 (-.169, 0.418) | 0.133 (-.164, 0.429) | 0.129 (-.167, 0.426) |
| XS_VLDL_FC | 0.266 (-0.029, 0.561) | 0.225 (-0.066, 0.515) | 0.157 (-.136, 0.449) | 0.131 (-.163, 0.426) | 0.148 (-.149, 0.445) | 0.143 (-.154, 0.441) |
| IDL_FC | 0.205 (-0.093, 0.502) | 0.184 (-0.112, 0.479) | 0.098 (-.199, 0.395) | 0.069 (-.229, 0.368) | 0.093 (-.207, 0.394) | 0.093 (-.208, 0.393) |
| L_LDL_FC | 0.197 (-0.098, 0.492) | 0.174 (-0.119, 0.467) | 0.082 (-.212, 0.376) | 0.054 (-.241, 0.349) | 0.086 (-.211, 0.383) | 0.082 (-.216, 0.379) |
| M_LDL_FC | 0.203 (-0.090, 0.495) | 0.173 (-0.117, 0.462) | 0.075 (-.215, 0.365) | 0.044 (-.247, 0.335) | 0.079 (-.215, 0.373) | 0.069 (-.225, 0.363) |
| S_LDL_FC | 0.218 (-0.081, 0.518) | 0.185 (-0.111, 0.481) | 0.088 (-.208, 0.385) | 0.057 (-.241, 0.355) | 0.092 (-.209, 0.393) | 0.078 (-.223, 0.379) |
| XL_HDL_FC | 0.071 (-0.189, 0.331) | 0.124 (-0.132, 0.379) | 0.109 (-.149, 0.368) | 0.070 (-.191, 0.332) | 0.063 (-.201, 0.328) | 0.046 (-.216, 0.308) |
| L_HDL_FC | -0.246 (-0.479, -0.011) | -0.172 (-0.396, 0.052) | -.179 (-.406, 0.047) | -.215 (-.445, 0.014) | -.245 (-.476, -.014) | -.247 (-.479, -.0162) |
| M_HDL_FC | -0.377 (-0.669, -0.085) | -0.341 (-0.632, -0.051) | -.368 (-.661, -.075) | -.393 (-.689, -.096) | -.437 (-.736, -.137) | -.416 (-.714, -.118) |
| S_HDL_FC | -0.576 (-0.897, -0.254) | -0.565 (-0.887, -0.244) | -.611 (-.935, -.287) | -.652 (-.978, -.325) | -.697 (-1.028, -.366) | -.691 (-1.02, -.362) |
| Free cholesterol | 0.127 (-0.179, 0.436) | 0.109 (-0.196, 0.415) | 0.015 (-.292, 0.322) | -.025 (-.333, 0.284) | -.0082 (-.321, 0.305) | -.0067 (-.319, 0.306) |

**Table S64. Multivariable regression analyses of specific pesticides use with total lipid subclasses with standardised metabolomic biomarkers in men**

| **Specific pesticides, β (95% CI), Males** | | | | | | |
| --- | --- | --- | --- | --- | --- | --- |
| **Total lipids** | **Unadjusted** | **+ BMI** | **+ SEP** | **+ season of pesticide use** | **+ alcohol consumption** | **+ latitude of residence** |
| XXL_VLDL_L | -0.093 (-0.334, 0.149) | -0.014 (-0.249, 0.221) | -.020 (-.259, 0.218) | -.018 (-.257, 0.220) | -.0026 (-.245, 0.240) | -.0039 (-.247, 0.239) |
| XL_VLDL_L | -0.129 (-0.371, 0.113) | -0.065 (-0.301, 0.171) | -.072 (-.313, 0.168) | -.071 (-.312, 0.169) | -.049 (-.294, 0.196) | -.046 (-.291, 0.199) |
| L_VLDL_L | -0.096 (-0.331, 0.139) | -0.036 (-0.265, 0.193) | -.0095 (-.243, 0.224) | -.011 (-.244, 0.222) | 0.018 (-.219, 0.255) | 0.023 (-.214, 0.261) |
| M_VLDL_L | -0.071 (-0.303, 0.161) | -0.005 (-0.229, 0.219) | 0.011 (-.218, 0.239) | 0.011 (-.218, 0.239) | 0.022 (-.210, 0.255) | 0.025 (-.207, 0.258) |
| S_VLDL_L | -0.125 (-0.342, 0.092) | -0.057 (-0.267, 0.153) | -.056 (-.269, 0.158) | -.058 (-.272, 0.156) | -.053 (-.271, 0.165) | -.062 (-.279, 0.156) |
| XS_VLDL_L | -0.128 (-0.356, 0.099) | -0.070 (-0.295, 0.154) | -.108 (-.336, 0.120) | -.111 (-.339, 0.117) | -.121 (-.352, 0.111) | -.137 (-.368, 0.093) |
| IDL_L | -0.068 (-0.306, 0.169) | -0.027 (-0.264, 0.211) | -.092 (-.332, 0.149) | -.097 (-.337, 0.143) | -.113 (-.357, 0.130) | -.127 (-.369, 0.115) |
| L_LDL_L | -0.054 (-0.294, 0.185) | -0.013 (-0.252, 0.226) | -.078 (-.320, 0.165) | -.084 (-.326, 0.158) | -.102 (-.347, 0.144) | -.115 (-.359, 0.128) |
| M_LDL_L | -0.049 (-0.288, 0.191) | -0.008 (-0.246, 0.231) | -.067 (-.309, 0.176) | -.074 (-.316, 0.168) | -.091 (-.337, 0.154) | -.105 (-.349, 0.139) |
| S_LDL_L | -0.058 (-0.293, 0.176) | -0.020 (-0.254, 0.214) | -.077 (-.315, 0.161) | -.084 (-.321, 0.153) | -.096 (-.337, 0.145) | -.109 (-.348, 0.131) |
| XL_HDL_L | 0.064 (-0.176, 0.303) | 0.013 (-0.221, 0.248) | -.018 (-.257, 0.221) | -.017 (-.256, 0.222) | 0.011 (-.232, 0.255) | -.001 (-.244, 0.242) |
| L_HDL_L | -0.010 (-0.248, 0.227) | -0.074 (-0.304, 0.156) | -.098 (-.333, 0.136) | -.103 (-.337, 0.131) | -.060 (-.297, 0.177) | -.054 (-.290, 0.182) |
| M_HDL_L | -0.196 (-0.428, 0.035) | -0.223 (-0.455, 0.009) | -.218 (-.454, 0.017) | -.224 (-.459, 0.011) | -.174 (-.409, 0.061) | -.154 (-.386, 0.077) |
| S_HDL_L | -0.188 (-0.428, 0.052) | -0.182 (-0.424, 0.059) | -.208 (-.453, 0.037) | -.217 (-.461, 0.027) | -.184 (-.428, 0.061) | -.180 (-.422, 0.061) |

**Table S65. Multivariable regression analyses of specific pesticides use with total lipid subclasses with standardised metabolomic biomarkers in women**

| **Specific pesticides, β (95% CI), Females** | | | | | | |
| --- | --- | --- | --- | --- | --- | --- |
| **Total lipids** | **Unadjusted** | **+ BMI** | **+ SEP** | **+ season of pesticide use** | **+ alcohol consumption** | **+ latitude of residence** |
| XXL_VLDL_L | 0.328 (0.055, 0.601) | 0.265 (0.0043, 0.527) | 0.250 (-.013, 0.513) | 0.237 (-.029, 0.504) | 0.264 (-.005, 0.533) | 0.269 (0.00021, 0.538) |
| XL_VLDL_L | 0.239 (-0.052, 0.530) | 0.172 (-0.108, 0.453) | 0.145 (-.138, 0.428) | 0.124 (-.162, 0.410) | 0.146 (-.144, 0.435) | 0.166 (-.123, 0.455) |
| L_VLDL_L | 0.277 (-0.017, 0.570) | 0.202 (-0.079, 0.483) | 0.198 (-.086, 0.483) | 0.179 (-.109, 0.467) | 0.215 (-.077, 0.507) | 0.231 (-.061, 0.523) |
| M_VLDL_L | 0.293 (0.021, 0.565) | 0.216 (-0.044, 0.475) | 0.187 (-.075, 0.449) | 0.171 (-.093, 0.436) | 0.200 (-.067, 0.468) | 0.219 (-.048, 0.486) |
| S_VLDL_L | 0.320 (0.031, 0.609) | 0.243 (-0.034, 0.519) | 0.197 (-.082, 0.476) | 0.164 (-.117, 0.445) | 0.179 (-.104, 0.463) | 0.178 (-.106, 0.462) |
| XS_VLDL_L | 0.331 (0.037, 0.624) | 0.276 (-0.011, 0.562) | 0.206 (-.082, 0.495) | 0.178 (-.112, 0.469) | 0.194 (-.099, 0.486) | 0.188 (-.105, 0.481) |
| IDL_L | 0.235 (-0.059, 0.529) | 0.202 (-0.088, 0.493) | 0.114 (-.177, 0.406) | 0.084 (-.209, 0.377) | 0.105 (-.190, 0.400) | 0.103 (-.192, 0.398) |
| L_LDL_L | 0.221 (-0.079, 0.520) | 0.188 (-0.108, 0.484) | 0.097 (-.200, 0.394) | 0.067 (-.231, 0.365) | 0.098 (-.203, 0.398) | 0.095 (-.206, 0.395) |
| M_LDL_L | 0.225 (-0.079, 0.529) | 0.189 (-0.111, 0.490) | 0.101 (-.201, 0.403) | 0.073 (-.229, 0.375) | 0.107 (-.198, 0.412) | 0.103 (-.202, 0.408) |
| S_LDL_L | 0.221 (-0.077, 0.519) | 0.185 (-0.109, 0.479) | 0.103 (-.192, 0.399) | 0.073 (-.223, 0.369) | 0.107 (-.192, 0.407) | 0.102 (-.197, 0.401) |
| XL_HDL_L | 0.0097 (-0.256, 0.275) | 0.068 (-0.192, 0.328) | 0.054 (-.209, 0.316) | 0.0083 (-.257, 0.274) | -.0069 (-.275, 0.261) | -.033 (-.298, 0.233) |
| L_HDL_L | -0.329 (-0.590, -0.068) | -0.256 (-0.508, -0.003) | -.276 (-.531, -.021) | -.319 (-.578, -.062) | -.358 (-.618, -.098) | -.358 (-.619, -.097) |
| M_HDL_L | -0.405 (-0.705, -0.106) | -0.374 (-0.673, -0.075) | -.397 (-.699, -.096) | -.415 (-.720, -.110) | -.461 (-.769, -.153) | -.437 (-.743, -.131) |
| S_HDL_L | -0.333 (-0.653, -0.014) | -0.339 (-0.659, -0.020) | -.402 (-.724, -.081) | -.424 (-.747, -.100) | -.454 (-.782, -.127) | -.447 (-.772, -.122) |

**Table S66. Multivariable regression analyses of specific pesticides use with total cholesterol subclasses with standardised metabolomic biomarkers in men**

| **Specific pesticides, β (95% CI), Males** | | | | | | |
| --- | --- | --- | --- | --- | --- | --- |
| **Total cholesterol** | **Unadjusted** | **+ BMI** | **+ SEP** | **+ season of pesticide use** | **+ alcohol consumption** | **+ latitude of residence** |
| XXL_VLDL_C | -0.037 (-0.268, 0.195) | 0.039 (-0.188, 0.265) | 0.033 (-.197, 0.263) | 0.036 (-.194, 0.266) | 0.036 (-.198, 0.271) | 0.032 (-.202, 0.267) |
| XL_VLDL_C | -0.077 (-0.314, 0.159) | -0.007 (-0.238, 0.225) | -.023 (-.259, 0.212) | -.021 (-.257, 0.214) | -.015 (-.256, 0.225) | -.020 (-.260, 0.220) |
| L_VLDL_C | -0.076 (-0.311, 0.158) | -0.012 (-0.241, 0.217) | 0.023 (-.211, 0.256) | 0.022 (-.211, 0.256) | 0.036 (-.202, 0.273) | 0.035 (-.202, 0.273) |
| M_VLDL_C | -0.045 (-0.280, 0.191) | 0.026 (-0.201, 0.254) | 0.029 (-.202, 0.262) | 0.029 (-.203, 0.262) | 0.031 (-.206, 0.267) | 0.028 (-.208, 0.264) |
| S_VLDL_C | -0.119 (-0.337, 0.099) | -0.054 (-0.267, 0.159) | -.074 (-.291, 0.144) | -.077 (-.294, 0.141) | -.084 (-.305, 0.138) | -.097 (-.317, 0.124) |
| XS_VLDL_C | -0.104 (-0.344, 0.136) | -0.051 (-0.289, 0.187) | -.103 (-.345, 0.139) | -.105 (-.347, 0.137) | -.122 (-.368, 0.123) | -.139 (-.384, 0.105) |
| IDL_C | -0.072 (-0.313, 0.169) | -0.032 (-0.273, 0.209) | -.099 (-.345, 0.145) | -.105 (-.349, 0.139) | -.124 (-.371, 0.124) | -.136 (-.383, 0.110) |
| L_LDL_C | -0.048 (-0.288, 0.193) | -0.008 (-0.249, 0.232) | -.074 (-.319, 0.169) | -.080 (-.324, 0.163) | -.102 (-.349, 0.146) | -.114 (-.359, 0.132) |
| M_LDL_C | -0.036 (-0.271, 0.199) | -0.0001 (-0.235, 0.235) | -.058 (-.297, 0.181) | -.064 (-.302, 0.174) | -.086 (-.328, 0.156) | -.098 (-.338, 0.143) |
| S_LDL_C | -0.046 (-0.281, 0.189) | -0.014 (-0.249, 0.221) | -.071 (-.310, 0.169) | -.077 (-.315, 0.162) | -.096 (-.339, 0.146) | -.107 (-.349, 0.134) |
| LDL_C | -0.043 (-0.278, 0.191) | -0.007 (-0.241, 0.228) | -.068 (-.307, 0.170) | -.074 (-.312, 0.164) | -.095 (-.336, 0.146) | -.107 (-.347, 0.133) |
| XL_HDL_C | 0.053 (-0.192, 0.297) | 0.018 (-0.223, 0.259) | -.024 (-.270, 0.222) | -.021 (-.267, 0.225) | 0.0043 (-.247, 0.255) | -.012 (-.262, 0.238) |
| L_HDL_C | 0.031 (-0.214, 0.276) | -0.038 (-0.274, 0.199) | -.064 (-.305, 0.177) | -.067 (-.308, 0.174) | -.027 (-.272, 0.217) | -.023 (-.268, 0.221) |
| M_HDL_C | -0.166 (-0.403, 0.071) | -0.204 (-0.440, 0.032) | -.194 (-.434, 0.046) | -.199 (-.439, 0.040) | -.155 (-.395, 0.085) | -.132 (-.368, 0.105) |
| S_HDL_C | -0.107 (-0.357, 0.143) | -0.105 (-0.358, 0.147) | -.142 (-.398, 0.115) | -.152 (-.406, 0.103) | -.145 (-.403, 0.112) | -.151 (-.406, 0.105) |
| HDL_C_ | -0.096 (-0.323, 0.132) | -0.154 (-0.376, 0.068) | -.178 (-.404, 0.048) | -.184 (-.409, 0.042) | -.143 (-.369, 0.084) | -.139 (-.365, 0.087) |
| Serum C | -0.111 (-0.358, 0.135) | -0.082 (-0.330, 0.166) | -.141 (-.393, 0.111) | -.148 (-.399, 0.103) | -.147 (-.401, 0.108) | -.157 (-.409, 0.096) |
| Remnant C | -0.105 (-0.338, 0.128) | -0.041 (-0.269, 0.187) | -.083 (-.315, 0.149) | -.086 (-.318, 0.146) | -.095 (-.331, 0.141) | -.107 (-.342, 0.127) |

**Table S67. Multivariable regression analyses of specific pesticides use with total cholesterol subclasses with standardised metabolomic biomarkers in women**

| **Specific pesticides, β (95% CI), Females** | | | | | | |
| --- | --- | --- | --- | --- | --- | --- |
| **Total cholesterol** | **Unadjusted** | **+ BMI** | **+ SEP** | **+ season of pesticide use** | **+ alcohol consumption** | **+ latitude of residence** |
| XXL_VLDL_C | 0.368 (0.078, 0.657) | 0.309 (0.029, 0.588) | 0.284 (0.0019, 0.565) | 0.266 (-.019, 0.551) | 0.291 (0.0028, 0.579) | 0.295 (0.0074, 0.583) |
| XL_VLDL_C | 0.314 (0.017, 0.611) | 0.248 (-.0391, 0.535) | 0.214 (-.073, 0.501) | 0.198 (-.092, 0.489) | 0.231 (-.063, 0.525) | 0.239 (-.054, 0.533) |
| L_VLDL_C | 0.379 (0.077, 0.682) | 0.306 (0.015, 0.597) | 0.295 (0.00056, 0.589) | 0.276 (-.022, 0.573) | 0.307 (0.0054, 0.609) | 0.313 (0.011, 0.615) |
| M_VLDL_C | 0.356 (0.078, 0.634) | 0.283 (0.017, 0.549) | 0.237 (-.032, 0.506) | 0.216 (-.055, 0.487) | 0.243 (-.032, 0.517) | 0.256 (-.019, 0.530) |
| S_VLDL_C | 0.296 (0.0039, 0.587) | 0.227 (-0.055, 0.508) | 0.165 (-.119, 0.449) | 0.134 (-.151, 0.419) | 0.161 (-.127, 0.449) | 0.155 (-.133, 0.443) |
| XS_VLDL_C | 0.308 (0.013, 0.604) | 0.261 (-0.030, 0.552) | 0.195 (-.098, 0.488) | 0.179 (-.116, 0.476) | 0.207 (-.090, 0.504) | 0.199 (-.098, 0.496) |
| IDL_C | 0.221 (-0.078, 0.521) | 0.190 (-0.107, 0.487) | 0.103 (-.195, 0.401) | 0.079 (-.221, 0.379) | 0.109 (-.193, 0.411) | 0.109 (-.193, 0.411) |
| L_LDL_C | 0.216 (-0.087, 0.518) | 0.183 (-0.116, 0.483) | 0.095 (-.206, 0.396) | 0.071 (-.232, 0.373) | 0.109 (-.194, 0.414) | 0.108 (-.196, 0.413) |
| M_LDL_C | 0.211 (-0.092, 0.514) | 0.179 (-0.121, 0.479) | 0.098 (-.203, 0.399) | 0.077 (-.225, 0.379) | 0.121 (-.184, 0.425) | 0.119 (-.185, 0.423) |
| S_LDL_C | 0.212 (-0.086, 0.510) | 0.180 (-0.115, 0.475) | 0.106 (-.191, 0.403) | 0.085 (-.213, 0.383) | 0.129 (-.171, 0.429) | 0.126 (-.175, 0.426) |
| LDL_C | 0.212 (-0.086, 0.510) | 0.180 (-0.115, 0.475) | 0.097 (-.199, 0.394) | 0.075 (-.223, 0.372) | 0.116 (-.184, 0.415) | 0.114 (-.186, 0.414) |
| XL_HDL_C | 0.077 (-0.200, 0.353) | 0.118 (-0.156, 0.392) | 0.104 (-.173, 0.381) | 0.061 (-.219, 0.341) | 0.054 (-.229, 0.338) | 0.018 (-.261, 0.297) |
| L_HDL_C | -0.291 (-0.535, -0.046) | -0.216 (-0.451, 0.018) | -.228 (-.465, 0.0086) | -.268 (-.508, -.028) | -.302 (-.543, -.059) | -.304 (-.546, -.062) |
| M_HDL_C | -0.438 (-0.729, -0.146) | -0.402 (-0.693, -0.111) | -.413 (-.707, -.119) | -.416 (-.713, -.119) | -.456 (-.756, -.157) | -.431 (-.729, -.133) |
| S_HDL_C | -0.136 (-0.428, 0.156) | -0.145 (-0.437, 0.146) | -.223 (-.516, 0.069) | -.243 (-.536, 0.049) | -.241 (-.538, 0.057) | -.242 (-.538, 0.053) |
| HDL_C_ | -0.334 (-0.611, -.057) | -0.272 (-0.543, -0.00065) | -.305 (-.578, -.032) | -.352 (-.629, -.076) | -.387 (-.667, -.108) | -.387 (-.667, -.108) |
| Serum C | 0.115 (-0.193, 0.424) | 0.099 (-0.208, 0.405) | 0.0068 (-.301, 0.314) | -.033 (-.342, 0.276) | -.016 (-.329, 0.298) | -.017 (-.330, 0.297) |
| Remnant C | 0.313 (0.025, 0.601) | 0.256 (-.025, 0.537) | 0.181 (-.102, 0.463) | 0.156 (-.128, 0.440) | 0.187 (-.099, 0.474) | 0.188 (-.098, 0.475) |

**Table S68. Multivariable regression analyses of specific pesticides use with apolipoproteins, aminoacids, sphingomyelins, glucose metabolism, fatty acids, inflammation, and fluid balance in men**

| **Specific pesticides, β (95% CI), Males** | | | | |  |  |
| --- | --- | --- | --- | --- | --- | --- |
|  | **Unadjusted** | **+ BMI** | **+ SEP** | **+ season of pesticide use** | **+ alcohol consumption** | **+ latitude of residence** |
| **Apolipoproteins** |  |  |  |  |  |  |
| Apolipoprotein A1 [g/L] | -0.139 (-0.362, 0.084) | -0.169 (-0.392, 0.054) | -.191 (-.417, 0.036) | -.198 (-.424, 0.028) | -.158 (-.385, 0.069) | -.153 (-.379, 0.072) |
| Apolipoprotein B [g/L] | -0.103 (-0.338, 0.131) | -0.042 (-0.272, 0.187) | -.081 (-.315, 0.152) | -.086 (-.319, 0.147) | -.092 (-.329, 0.145) | -.102 (-.338, 0.134) |
| Ratio of Apo B/Apo A1 | -0.007 (-0.229, 0.214) | 0.071 (-0.139, 0.282) | 0.048 (-.167, 0.262) | 0.048 (-.167, 0.263) | 0.016 (-.202, 0.233) | 0.0029 (-.214, 0.220) |
| **Amino acids** |  |  |  |  |  |  |
| Alanine [µmol/L] | -0.220 (-0.462, 0.021) | -0.181 (-0.421, 0.060) | -.089 (-.334, 0.154) | -.094 (-.337, 0.149) | -.090 (-.337, 0.157) | -.099 (-.346, 0.146) |
| Glutamine [µmol/L] | -0.064 (-0.316, 0.188) | -0.088 (-0.340, 0.165) | -.139 (-.396, 0.118) | -.149 (-.402, 0.103) | -.157 (-.412, 0.099) | -.146 (-.401, 0.109) |
| Glycine [µmol/L] | -0.082 (-0.286, 0.123) | -0.090 (-0.296, 0.115) | -.083 (-.292, 0.126) | -.087 (-.295, 0.121) | -.133 (-.343, 0.077) | -.152 (-.358, 0.054) |
| Histidine [µmol/L] | 0.138 (-0.125, 0.402) | 0.146 (-0.119, 0.410) | 0.177 (-.092, 0.447) | 0.178 (-.091, 0.447) | 0.164 (-.108, 0.436) | 0.171 (-.101, 0.443) |
| **Branched-chain amino acids** |  |  |  |  |  |  |
| Isoleucine [µmol/L] | -0.212 (-0.435, 0.012) | -0.154 (-0.372, 0.064) | -.126 (-.348, 0.095) | -.128 (-.349, 0.094) | -.125 (-.351, 0.099) | -.128 (-.353, 0.096) |
| Leucine [µmol/L] | -0.194 (-0.412, 0.025) | -0.154 (-0.369, 0.061) | -.123 (-.341, 0.095) | -.128 (-.346, 0.089) | -.126 (-.348, 0.096) | -.135 (-.355, 0.086) |
| Valine [µmol/L] | -0.234 (-0.446, -0.022) | -0.208 (-0.419, 0.0029) | -.176 (-.389, 0.038) | -.179 (-.394, 0.034) | -.201 (-.419, 0.016) | -.207 (-.423, 0.0083) |
| **Aromatic amino acids** |  |  |  |  |  |  |
| Phenylalanine [µmol/L] | 0.092 (-0.137, 0.321) | 0.143 (-0.079, 0.366) | 0.121 (-.105, 0.348) | 0.111 (-.113, 0.335) | 0.072 (-.155, 0.299) | 0.042 (-.179, 0.263) |
| Tyrosine [µmol/L] | -0.094 (-0.327, 0.139) | -0.061 (-0.291, 0.169) | -.025 (-.259, 0.209) | -.029 (-.262, 0.204) | -.035 (-.273, 0.202) | -.032 (-.269, 0.205) |
| **Ketone bodies** |  |  |  |  |  |  |
| Acetoacetate [µmol/L] | -0.209 (-0.446, 0.027) | -0.231 (-0.469, 0.0067) | -.273 (-.515, -.032) | -.277 (-.518, -.035) | -.249 (-.493, -.0044) | -.246 (-.489, -.0015) |
| Acetate [µmol/L] | 0.146 (-0.098, 0.389) | 0.141 (-0.103, 0.386) | 0.188 (-.061, 0.436) | 0.186 (-.062, 0.434) | 0.189 (-.064, 0.442) | 0.183 (-.069, 0.435) |
| beta-hydroxybutyrate [µmol/L] | -0.152 (-0.381, 0.077) | -0.174 (-0.404, 0.055) | -.216 (-.449, 0.016) | -.223 (-.455, 0.0099) | -.197 (-.430, 0.037) | -.209 (-.442, 0.023) |
| **Sphingolipids** |  |  |  |  |  |  |
| Sphingomyelin | -0.217 (-0.463, 0.029) | -0.208 (-0.457, 0.039) | -.259 (-.512, -.0068) | -.268 (-.519, -.017) | -.257 (-.511, -.0027) | -.263 (-.515, -.012) |
| Total choline | -0.197 (-0.426, 0.031) | -0.183 (-0.414, 0.047) | -.221 (-.455, 0.013) | -.228 (-.461, 0.0055) | -.198 (-.433, 0.037) | -.202 (-.435, 0.031) |
| **Glycolysis related metabolites** |  |  |  |  |  |  |
| Citrate [µmol/L] | 0.014 (-0.233, 0.262) | -0.013 (-0.263, 0.236) | -.025 (-.278, 0.228) | -.036 (-.287, 0.216) | -.062 (-.318, 0.193) | -.069 (-.323, 0.184) |
| Glucose [mmol/L] | -0.207 (-0.446, 0.033) | -.198 (-.439, 0.043) | -.231 (-.476, 0.014) | -.241 (-.484, 0.0022) | -.225 (-.471, 0.021) | -.224 (-.469, 0.020) |
| Glycerol [mmol/L] | 0.213 (-0.036, 0.462) | 0.189 (-0.062, 0.439) | 0.178 (-.076, 0.432) | 0.188 (-.066, 0.441) | 0.207 (-.050, 0.465) | 0.222 (-.035, 0.479) |
| Lactate [mmol/L] | -0.256 (-0.496, -0.016) | -0.241 (-0.482, 0.00021) | -.176 (-.421, 0.068) | -.178 (-.423, 0.068) | -.171 (-.419, 0.078) | -.188 (-.432, 0.056) |
| Pyruvate [mmol/L] | 0.125 (-0.119, 0.369) | 0.108 (-0.138, 0.354) | 0.054 (-.195, 0.304) | 0.059 (-.189, 0.309) | 0.043 (-.210, 0.296) | 0.040 (-.213, 0.293) |
| **Fatty acids** |  |  |  |  |  |  |
| Total fatty acids [mmol/L] | -0.221 (-0.459, 0.017) | -0.176 (-0.412, 0.061) | -.193 (-.433, 0.048) | -.198 (-.438, 0.042) | -.168 (-.412, 0.075) | -.173 (-.415, 0.069) |
| Monounsaturated fatty acids [mmol/L] | -0.271 (-0.506, -0.037) | -0.219 (-0.449, 0.012) | -.217 (-.451, 0.018) | -.219 (-.454, 0.015) | -.186 (-.423, 0.050) | -.189 (-.425, 0.046) |
| Saturated fatty acids [mmol/L] | -0.074 (-0.309, 0.162) | -0.028 (-0.262, 0.205) | -.077 (-.315, 0.160) | -.082 (-.319, 0.155) | -.056 (-.297, 0.184) | -.063 (-.302, 0.175) |
| **Inflammation** |  |  |  |  |  |  |
| Alpha-1-acid glycoprotein [mmol/L] | -0.305 (-0.551, -0.058) | -0.264 (-0.507, -0.019) | -.253 (-.501, -.0049) | -.260 (-.507, -.014) | -.259 (-.510, -.0084) | -.262 (-.511, -.013) |
| **Fluid balance** |  |  |  |  |  |  |
| Creatinine [µmol/L] | -0.097 (-0.414, 0.219) | -0.093 (-0.411, 0.224) | -.028 (-.351, 0.295) | -.031 (-.352, 0.291) | -.045 (-.373, 0.283) | -.041 (-.369, 0.287) |
| Albumin [standardized concentration units] | -0.157 (-0.425, 0.109) | -0.163 (-0.433, 0.107) | -.193 (-.468, 0.083) | -.202 (-.475, 0.072) | -.207 (-.486, 0.071) | -.207 (-.483, 0.069) |

**Table S69. Multivariable regression analyses of specific pesticides use with apolipoproteins, aminoacids, sphingomyelins, glucose metabolism, fatty acids, inflammation, and fluid balance in women**

| **Specific pesticides, β (95% CI), Females** | | | | | | |
| --- | --- | --- | --- | --- | --- | --- |
|  | **Unadjusted** | **+ BMI** | **+ SEP** | **+ season of pesticide use** | **+ alcohol consumption** | **+ latitude of residence** |
| **Apolipoproteins** |  |  |  |  |  |  |
| Apolipoprotein A1 [g/L] | -.307 (-.612, -.0015) | -.262 (-.565, 0.041) | -.319 (-.624, -.013) | -.370 (-.678, -.062) | -.399 (-.712, -.087) | -.393 (-.706, -.080) |
| Apolipoprotein B [g/L] | 0.298 (0.012, 0.583) | 0.242 (-.036, 0.519) | 0.162 (-.116, 0.441) | 0.136 (-.145, 0.416) | 0.170 (-.113, 0.453) | 0.173 (-.109, 0.456) |
| Ratio of Apo B/Apo A1 | 0.491 (0.216, 0.765) | 0.407 (0.146, 0.668) | 0.369 (0.105, 0.632) | 0.377 (0.111, 0.644) | 0.429 (0.163, 0.696) | 0.428 (0.161, 0.695) |
| **Amino acids** |  |  |  |  |  |  |
| Alanine [µmol/L] | 0.172 (-0.124, 0.468) | 0.134 (-0.159, 0.427) | 0.128 (-.167, 0.424) | 0.100 (-.198, 0.398) | 0.082 (-.221, 0.384) | 0.076 (-.225, 0.378) |
| Glutamine [µmol/L] | -0.146 (-0.434, 0.142) | -0.135 (-0.423, 0.153) | -.177 (-.468, 0.114) | -.157 (-.450, 0.136) | -.121 (-.418, 0.176) | -.096 (-.389, 0.197) |
| Glycine [µmol/L] | -0.210 (-0.559, 0.139) | -0.217 (-0.566, 0.131) | -.316 (-.667, 0.034) | -.303 (-.656, 0.050) | -.262 (-.621, 0.097) | -.283 (-.639, 0.073) |
| Histidine [µmol/L] | -0.019 (-0.314, 0.276) | -0.011 (-0.305, 0.283) | 0.071 (-.224, 0.367) | 0.064 (-.236, 0.363) | 0.044 (-.259, 0.348) | 0.052 (-.252, 0.356) |
| **Branched-chain amino acids** |  |  |  |  |  |  |
| Isoleucine [µmol/L] | 0.051 (-0.217, 0.319) | -0.020 (-0.279, 0.238) | -.027 (-.289, 0.234) | -.0035 (-.268, 0.261) | 0.051 (-.217, 0.319) | 0.064 (-.203, 0.331) |
| Leucine [µmol/L] | -0.010 (-0.279, 0.259) | -0.076 (-0.338, 0.186) | -.089 (-.354, 0.174) | -.082 (-.348, 0.184) | -.066 (-.336, 0.204) | -.071 (-.339, 0.198) |
| Valine [µmol/L] | -0.144 (-0.426, 0.137) | -0.206 (-0.482, 0.069) | -.201 (-.479, 0.076) | -.174 (-.453, 0.106) | -.147 (-.431, 0.138) | -.141 (-.422, 0.141) |
| **Aromatic amino acids** |  |  |  |  |  |  |
| Phenylalanine [µmol/L] | 0.310 (0.0085, 0.612) | 0.244 (-0.049, 0.538) | 0.175 (-.120, 0.469) | 0.156 (-.139, 0.452) | 0.118 (-.181, 0.417) | 0.094 (-.200, 0.389) |
| Tyrosine [µmol/L] | 0.090 (-0.198, 0.378) | 0.045 (-0.240, 0.330) | 0.026 (-.262, 0.313) | 0.032 (-.259, 0.323) | 0.012 (-.283, 0.306) | 0.036 (-.256, 0.327) |
| **Ketone bodies** |  |  |  |  |  |  |
| Acetoacetate [µmol/L] | -0.280 (-0.587, 0.027) | -0.270 (-0.578, 0.037) | -.318 (-.629, -.0074) | -.351 (-.666, -.037) | -.369 (-.687, -.052) | -.362 (-.678, -.045) |
| Acetate [µmol/L] | -0.215 (-0.507, 0.078) | -0.213 (-0.507, 0.080) | -.244 (-.538, 0.050) | -.256 (-.553, 0.041) | -.269 (-.570, 0.031) | -.270 (-.570, 0.029) |
| beta-hydroxybutyrate [µmol/L] | -0.205 (-0.531, 0.121) | -0.201 (-0.527, 0.125) | -.263 (-.589, 0.064) | -.304 (-.634, 0.027) | -.299 (-.632, 0.035) | -.315 (-.648, 0.017) |
| **Sphingolipids** |  |  |  |  |  |  |
| Sphingomyelin | -0.046 (-0.358, 0.266) | -0.047 (-0.358, 0.264) | -.137 (-.449, 0.175) | -.189 (-.503, 0.124) | **-**.187 (-.506, 0.131) | -.184 (-.503, 0.134) |
| Total choline | -0.049 (-0.359, 0.261) | -0.046 (-0.355, 0.263) | -.121 (-.432, 0.189) | -.175 (-.488, 0.138) | -.192 (-.510, 0.126) | -.188 (-.506, 0.131) |
| **Glycolysis related metabolites** |  |  |  |  |  |  |
| Citrate [µmol/L] | 0.109 (-0.186, 0.406) | 0.119 (-0.177, 0.416) | 0.117 (-.182, 0.416) | 0.078 (-.224, 0.379) | 0.082 (-.223, 0.388) | 0.092 (-.210, 0.395) |
| Glucose [mmol/L] | -.235 (-.545, 0.075) | -.247 (-.557, 0.064) | -.312 (-.624, 0.00029) | -.284 (-.596, 0.029) | -.278 (-.596, 0.039) | -.257 (-.572, 0.059) |
| Glycerol [mmol/L] | 0.240 (-0.057, 0.538) | 0.264 (-0.032, 0.561) | 0.301 (0.0015, 0.601) | 0.297 (-.0057, 0.599) | 0.291 (-.016, 0.598) | 0.326 (0.019, 0.632) |
| Lactate [mmol/L] | 0.071 (-0.227, 0.369) | 0.037 (-0.259, 0.333) | 0.046 (-.253, 0.345) | 0.038 (-.265, 0.341) | -.005 (-.312, 0.301) | -.020 (-.320, 0.280) |
| Pyruvate [mmol/L] | 0.018 (-0.279, 0.315) | 0.049 (-0.246, 0.345) | 0.048 (-.251, 0.346) | 0.031 (-.270, 0.333) | 0.072 (-.234, 0.378) | 0.081 (-.225, 0.387) |
| **Fatty acids** |  |  |  |  |  |  |
| Total fatty acids [mmol/L] | 0.109 (-0.195, 0.414) | 0.079 (-0.221, 0.381) | 0.0082 (-.295, 0.311) | -.043 (-.348, 0.262) | -.055 (-.365, 0.254) | -.051 (-.361, 0.258) |
| Monounsaturated fatty acids [mmol/L] | 0.200 (-0.093, 0.493) | 0.149 (-0.137, 0.437) | 0.091 (-.198, 0.380) | 0.037 (-.254, 0.329) | 0.015 (-.279, 0.309) | 0.018 (-.277, 0.313) |
| Saturated fatty acids [mmol/L] | 0.117 (-0.191, 0.425) | 0.093 (-0.212, 0.398) | 0.012 (-.295, 0.318) | -.039 (-.349, 0.269) | -.054 (-.367, 0.259) | -.049 (-.363, 0.264) |
| **Inflammation** |  |  |  |  |  |  |
| Alpha-1-acid glycoprotein [mmol/L] | -0.0075 (-0.302, 0.287) | -0.073 (-0.359, 0.213) | -.130 (-.418, 0.158) | -.154 (-.442, 0.134) | -.168 (-.460, 0.125) | -.161 (-.452, 0.130) |
| **Fluid balance** |  |  |  |  |  |  |
| Creatinine [µmol/L] | -0.012 (-0.205, 0.182) | -0.0087 (-0.203, 0.186) | 0.017 (-.179, 0.213) | 0.016 (-.182, 0.214) | 0.0067 (-.195, 0.208) | 0.015 (-.186, 0.216) |
| Albumin [standardized concentration units] | -0.336 (-0.612, -0.060) | -0.327 (-0.603, -0.051) | -.394 (-.672, -.117) | -.376 (-.653, -.099) | -.378 (-.660, -.096) | -.374 (-.653, -.095) |
